# ﻿DNA barcoding of the horsefly fauna (Diptera, Tabanidae) of Croatia with notes on the morphology and taxonomy of selected species from Chrysopsinae and Tabaninae

**DOI:** 10.3897/zookeys.1087.78707

**Published:** 2022-02-23

**Authors:** Stjepan Krčmar, Mladen Kučinić, Marco Pezzi, Branka Bruvo Mađarić

**Affiliations:** 1 Department of Biology, Josip Juraj Strossmayer University of Osijek, Cara Hadrijana 8/A, 31000 Osijek, Croatia Josip Juraj Strossmayer University of Osijek Osijek Croatia; 2 Department of Biology, Faculty of Science, University of Zagreb, 10000 Zagreb, Croatia University of Zagreb Zagreb Croatia; 3 Department of Life Sciences and Biotechnology, University of Ferrara, Via Luigi Borsari 46, 44121 Ferrara, Italy University of Ferrara Ferrara Italy; 4 Ruđer Bošković Institute, Bijenička cesta 54, 10000 Zagreb, Croatia Rudjer Bošković Institute Zagreb Croatia

**Keywords:** Barcode Index Number (BIN), cytochrome c oxidase gene subunit I (COI), Molecular Operational Taxonomic Unit (MOTU), species delimitation, vector species

## Abstract

In the Croatian fauna, horseflies (Tabanidae) are represented by 78 species belonging to two subfamilies, five tribes, and 10 genera. Identification of these species is based on morphological characteristics. In this study, 43 species of horseflies were analyzed. The highest number of species (19) belongs to the genus *Tabanus*, followed by the genera *Hybomitra* with seven species, *Haematopota* with six species, *Chrysops* with four species, *Atylotus* and *Philipomyia* with two species each, and the genera *Silvius*, *Dasyrhamphis*, and *Heptatoma* with one species each. The standard DNA barcoding region of the mitochondrial cytochrome c oxidase gene, subunit I (COI), was sequenced and compared to the Barcode of Life Database (BOLD). Our analyses confirmed our morphological identifications and added 16 new Barcode Index Numbers (BINs) for Tabanidae to BOLD. Potential problems in the systematics and taxonomy of this family are highlighted.

## ﻿Introduction

The Tabanidae comprise about 4400 species and include some of the largest biting flies, commonly called horseflies, deer flies, and clegs (Pape et al. 2011; Morita et al. 2016; Ježek et al. 2017). The females of horseflies are known worldwide as important mechanical vectors of viruses, bacteria, protozoa, and helminths that cause disease in wild and domestic animals (Foil 1989; Desquesnes and Dia 2004). Horseflies are important pests of livestock because of the blood loss and nuisance caused by their bites, so they have been associated with losses in livestock production (Baldacchino et al. 2014). The known human pathogens transmitted by horsefly species *Chrysopsdimidiatus* Wulp, 1885 and *C.silaceus* Austen, 1907 are *Loaloa* Cobbold, 1864 (Spirulida, Onchocercidae) in Africa (Chippaux et al. 2000; Cheke et al. 2003) and *Bacillusanthracis* Cohn, 1872 (Bacillales, Bacillaceae) (Chainey 1993). About 550 species of horseflies are known in the Palaearctic region (Chvála 1988), with 220 occurring in Europe (Pape et al. 2015).

Currently, the identification of horsefly species is mainly based on morphological features, but it requires a lot of experience and is extremely time-consuming. Some morphological features, i.e., colouration of the abdomen, antennae, maxillary palpi, and notopleural lobes, as well as the shape and colour of frontal calli (the lower callus, located at the lower part of the frons and the upper callus, often present on the middle of the frons), the width of the vertex, or post ocular margins, are considered important taxonomic characters for the identification of horseflies (Chvála et al. 1972). Distinguishing these morphological features can be difficult, leading to misidentifications. Therefore, many recent studies utilize DNA barcoding to verify/supplement their findings (Banerjee et al. 2015; Nitiyamatawat et al. 2017; Changbunjong et al. 2018; Mugasa et al. 2018; Morinière et al. 2019; Changbunjong et al. 2020; Mazumdar et al. 2021). DNA barcoding is established as a universal tool in biodiversity research, ensuring rapid and accurate species identification independent of the developmental stage (Hebert et al. 2003). In addition to species identification, DNA barcoding is also used to reveal genetic diversity and determine the presence of biotypes, and it can help to point out taxonomic ambiguities at species level and within species complexes (Votýpka et al. 2019).

The horsefly fauna is still poorly studied in the western and central Balkans. In Bosnia and Herzegovina, 62 species have been recorded (Mikuška et al. 2008), followed by Serbia with 45 species (Krčmar 2011; Aibulatov et al. 2012), Slovenia with 44 species (Krčmar and Bogdanović 2001), Montenegro with 42 species, North Macedonia with 40 species (Krčmar et al. 2002), and Kosovo with six species (Krčmar et al. 2002), while in Hungary 61 species have been recorded (Majer 2001).

In Croatia, 78 species classified in 10 genera and two subfamilies of horseflies have been recorded, many with differing requirements in habitat preference for larval and adult stages in the feeding area and annual periodicity (Krčmar et al. 2011). The subfamily Chrysopsinae comprises the genera *Silvius* Meigen, 1820 and *Chrysops* Meigen, 1803 with two and seven species, respectively. The majority of Croatian tabanid species belong to the subfamily Tabaninae, which is represented by eight genera. The genus *Tabanus* Linnaeus, 1758 includes the majority of species (30), followed by the genera *Hybomitra* Enderlein, 1922 with 17, *Haematopota* Meigen, 1803 with nine, *Atylotus* Osten-Sacken, 1876 with five, *Dasyrhamphis* Enderlein, 1922 with three, *Therioplectes* Zeller, 1842 and *Philipomyia* Olsufjev, 1964 with two species each, while for the genus *Heptatoma* Meigen, 1803 only one species is recorded (Krčmar et al. 2011). Molecular studies of horseflies in Croatia and surrounding countries are very limited (Biruš 2006), so this study represents the first comprehensive use of DNA barcoding for the identification and delimitation of species belonging to the Croatian, as well as regional, horsefly fauna.

## ﻿Materials and methods

**Figure 1.**
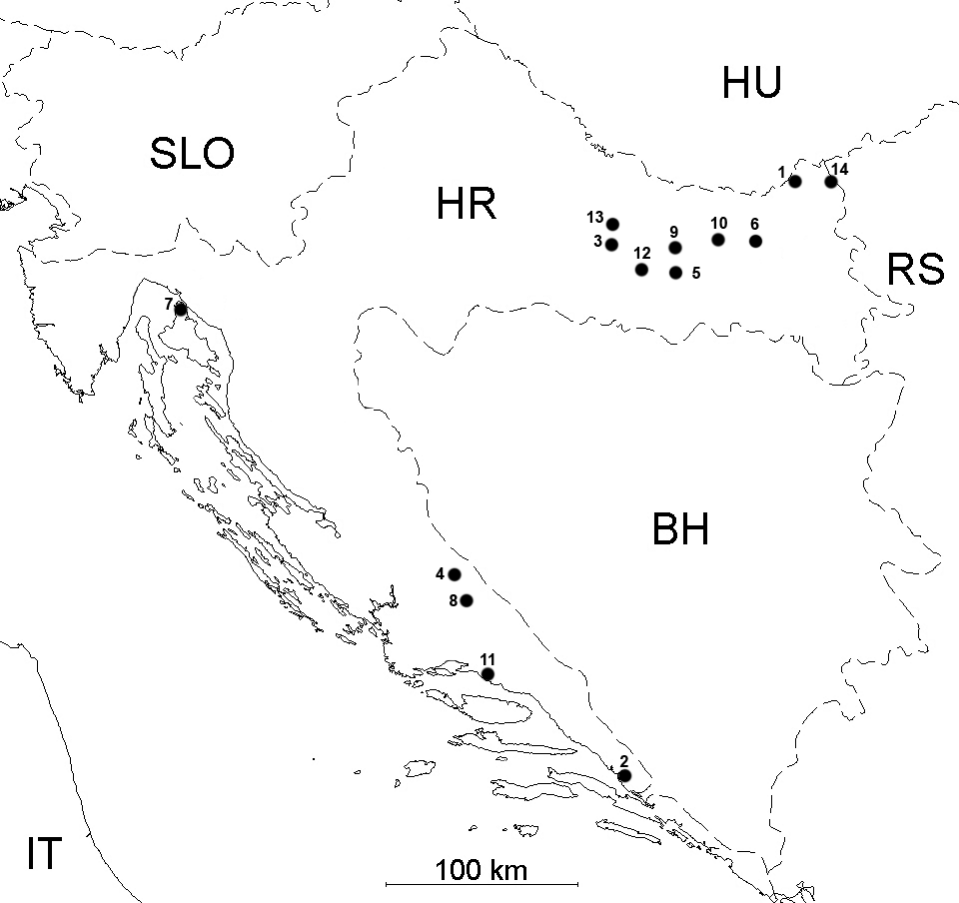
Sampling sites of horseflies (Diptera: Tabanidae) in Croatia: 1 – Branjina, 2 – Desne, 3 – Djedovica (Papuk Mountain), 4 – Donje Maovice, 5 – Kutjevo, 6 – Normanci, 7 – Njivice (Krk Island), 8 – Peruča, 9 – Petrov vrh (Papuk Mountain), 10 – Seona (Našice), 11 – Tugare, 12 – Velika, 13 – Voćin, 14 – Zmajevac. The details about localities can be found in BOLD project CROTA. Acronyms for the countries: HR: Croatia; SLO: Slovenia; HU: Hungary; RS: Republic of Serbia; BH: Bosnia and Herzegovina: IT: Italy.

Horseflies were collected on 14 localities (Fig. 1) during the summer months of 2015–2018 using Malaise traps (design by Townes 1972), Nzi traps according to the design of Mihok (2002), and canopy traps according to the design of Hribar et al. (1991). Identification, morphological description of analysed species, and nomenclature followed that of Chvála et al. (1972), Chvála (1988), Zeegers (2018), and Andreeva (2004). Several analysed species of *Tabanus*, *Hybomitra*, and *Haematopota* have a high morphological similarity. Morphological characteristics of females important for their identification are presented in Suppl. material 1 according to Chvála et al. (1972) and Zeegers (2018). All analysed species were identified using a stereomicroscope (Carl Zeiss, Jena, Germany) under magnification of 40×. The list of specimens analysed in this study is presented in Table 1. The horseflies were kept individually in 20 ml plastic tubes in 96% ethanol.

**Table 1.** List of analysed horsefly species from Croatian fauna.

Tribe	Species	locality/region	sample ID/voucher nr.	BOLD process ID nr.	BOLD hit (>98% identity)
Chrysopsini	*Chrysopscaecutiens*	Djedovica/CO	SK-4/CROBB288	CROTA004-20	*C.caecutiens*
	*Chrysopsparallelogrammus*	Zmajevac/CO	SK-3/CROBB287	CROTA003-20	*C.parallelogrammus* *
	*Chrysopsrelictus*	Zmajevac/CO	SK-1/CROBB285	CROTA001-20	*C.relictus* *
	*Chrysopsviduatus*	Zmajevac/CO	SK-2/CROBB286	CROTA002-20	*C.viduatus*
	*Silviusalpinus*	Velika/CO	SK-31/CROBB315	CROTA031-20	*S.alpinus* *
Diachlorini	***Dasyrhamphisumbrinus***	Njivice/ME	**SK-28**/**CROBB312**	CROTA028-20	-
	*Philipomyiaaprica*	Peruča/ME	SK-30/CROBB314	CROTA030-20	*T.bovinus* *
	*Philipomyiagraeca*	Desne/ME	SK-29/CROBB313	CROTA029-20	*T.bovinus* *
Haematopotini	***Haematopotagrandis***	Donje Maovice/ME	**SK-42**/**CROBB326**	CROTA042-20	-
	*Haematopotaitalica*	Zmajevac/CO	SK-35/CROBB319	CROTA035-20	*Ha.italica*
	***Haematopotapandazisi***	Branjina/CO	**SK-34**/**CROBB318**	CROTA034-20	-
	*Haematopotapluvialis*	Kutjevo/CO	SK-32/CROBB316	CROTA032-20	*Ha.pluvialis*
	*Haematopotascutellata*	Djedovica/CO	SK-36/CROBB320	CROTA036-20	*Ha.scutellata* *
	***Haematopotasubcylindrica***	Zmajevac/CO	**SK-33**/**CROBB317**	CROTA033-20	-
Heptatomini	*Heptatomapellucens*	Zmajevac/CO	SK-40/CROBB324	CROTA040-20	*He.pellucens* *
Tabanini	*Atylotusloewianus*	Branjina/CO	SK-38/CROBB322	CROTA038-20	*A.loewianus* *
	*Atylotusrusticus*	Peruča/ME	SK-37/CROBB321	CROTA037-20	*A.rusticus* *
	***Hybomitraacuminata***	Njivice/ME	**SK-22**/**CROBB306**	CROTA022-20	-
	*Hybomitrabimaculata*	Normanci/CO	SK-23/CROBB307	CROTA023-20	*H.bimaculata*
	***Hybomitrasolstitialis***	Zmajevac/CO	**SK-24**/**CROBB308**	CROTA024-20	-
	*Hybomitradistinguenda*	Djedovica/CO	SK-25/CROBB309	CROTA025-20	*H.distinguenda* *
	*Hybomitramuehlfeldi*	Zmajevac/CO	SK-26/CROBB310	CROTA026-20	*H.muehlfeldi* *
	***Hybomitrapilosa***	Seona/CO	**SK-27**/**CROBB311**	CROTA027-20	-
	*Hybomitraukrainica*	Zmajevac/CO	SK-39/CROBB323	CROTA039-20	*H.solstitialis*
	*Tabanusautumnalis*	Zmajevac/CO	SK-5/CROBB289	CROTA005-20	*T.autumnalis* *
	***Tabanusbifarius***	Njivice/ME	**SK-7/CROBB291**	CROTA007-20	-
	*Tabanusbovinus*	Zmajevac/CO	SK-8/CROBB292	CROTA008-20	*T.sudeticus*
	***Tabanusbriani***	Voćin/CO	**SK-45/ CROBB882**	CROTA045-21	-
	*Tabanusbromius*	Zmajevac/CO	SK-6/CROBB290	CROTA006-20	*T.bromius*
	*Tabanuscordiger*	Voćin/CO	SK-9/CROBB293	CROTA009-20	*T.cordiger* *
	***Tabanusdarimonti***	Desne/ME	**SK-10/CROBB294**	CROTA010-20	-
	***Tabanuseggeri***	Desne/ME	**SK-11/CROBB295**	CROTA011-20	-
	***Tabanusexclusus***	Tugare/ME	**SK-12/CROBB296**	CROTA012-20	-
	*Tabanusglaucopis*	Voćin/CO	SK-13/CROBB297	CROTA013-20	*T.glaucopis* *
	***Tabanuslunatus***	Tugare/ME	**SK-14/CROBB298**	CROTA014-20	-
	*Tabanusmaculicornis*	Normanci/CO	SK-15/CROBB299	CROTA015-20	*T.maculicornis*
	***Tabanusmiki***	Desne/ME	**SK-16/CROBB300**	CROTA016-20	-
	***Tabanusquatuornotatus***	Donje Maovice/ME	**SK-21**/**CROBB305**	CROTA021-20	-
	*Tabanusrupium*	Petrov vrh/CO	SK-17/CROBB301	CROTA017-20	*T.rupium*
	***Tabanusshannonellus***	Donje Maovice/ME	**SK-43**/**CROBB327**	CROTA043-20	-
	*Tabanusspodopterus*	Peruča/ME	SK-41/CROBB325	CROTA041-20	*T.spodopterus* *
	*Tabanussudeticus*	Zmajevac/CO	SK-18/CROBB302	CROTA018-20	*T.sudeticus* *
	*Tabanustergestinus*	Zmajevac/CO	SK-19/CROBB303	CROTA019-20	*T.tergestinus* *

Region: Continental = CO; Mediterranean = ME. Asterisks (*) denote hits with private records from BOLD database; entries constituting new BINs in BOLD are shown in bold font.

Genomic DNA was extracted from a single leg for each individual using the GenEluteTM Mammalian Genomic DNA Miniprep Kit (Sigma, St. Louis, MO, USA), following the protocol for rodent tail preparation with slight modifications (incubation in proteinase K overnight; DNA eluted in 100 µl of elution solution). Barcoded specimens are kept as vouchers in the Tabanidae collection of the Department of Biology of the Josip Juraj Strossmayer University of Osijek (listed in Table 1).

Standard barcoding region of mitochondrial cytochrome c oxidase I (COI) gene (Hebert et al. 2003) was successfully amplified for all horsefly specimens. Amplification mixtures included 1× DreamTaq reaction buffer with 2 mM MgCl_2_ (Thermo Scientific, Waltham, MA, USA), 0.2 mM dNTP mix (Qiagen, Hilden, Germany), 0.5 µM each primer (LCO1490 / HCO2198), 1.0 U DreamTaq polymerase (Thermo Scientific, Waltham, MA, USA) and 3 µl of DNA in 20 µl reaction volume. PCR products were enzymatically purified using the ExoI-rSAP system (NEB, Ipswich, MA, USA) following the manufacturer’s protocol and bidirectionally sequenced in Macrogen Europe Inc. (Amsterdam, the Netherlands) using amplification primers. Sequences were checked and edited in Geneious v. 8.1.4. (https://www.geneious.com) and subsequently deposited in NCBI GenBank and the Barcode of Life Database (BOLD) (NCBI GenBank acc. numbers MZ563329–MZ563371 and OM502029; BOLD ID numbers listed in Table 1; BOLD project CROTA). The BIN-RESL algorithm (Ratnasingham and Hebert 2013) assigned the new sequences to particular BINs in BOLD, corresponding to putative Molecular Operational Taxonomic Units (MOTUs).

The BOLD identification tool (http://www.boldsystems.org/index.php/IDS_OpenIdEngine; accessed on 2021-6-6) was used to compare DNA barcode sequences amplified from our samples with the public barcode data available in BOLD. The NCBI GenBank database was searched using the BLAST tool via MegaBlast algorithm (https://blast.ncbi.nlm.nih.gov/Blast.cgi; accessed on 2021-6-6). Publicly available COI sequences of conspecific and congeneric tabanid specimens (preferentially of the species confined to the Palaearctic region) were downloaded from BOLD and used in all subsequent analyses. As outgroups, three species from the family Rhagionidae were used: *Rhagiomaculatus* (De Geer, 1776), *Chrysopilusnubecula* (Fallén, 1814) and *Symphoromyiacrassicornis* (Panzer, 1806).

The COI sequences were analysed in the three following datasets: 1. tribe Chrysopsini; 2. tribes Haematopotini and Heptatomini united in a single dataset (because *Heptatoma* was long considered part of the tribe Haematopotini); 3. tribes Tabanini and Diachlorini united in a single dataset (because the genus *Philipomyia* was until recently considered part of the genus *Tabanus*). Multiple sequence alignments were conducted with MAFFT v. 7, using the “Auto” strategy (Katoh et al. 2019) (https://mafft.cbrc.jp/alignment/server/index.html). Final alignments are given in Suppl. material 2. Intraspecific and interspecific *p*-distances were calculated in MEGA v. 7.0.25 (Kumar et al. 2016). Neighbor-joining (NJ) trees based on the *p*-distance model were calculated in MEGA v. 7.0.25, and the robustness of the clades was assessed through 1000 bootstrap replicates. Maximum likelihood (ML) trees were constructed on PhyML 3.0 webserver (Guindon et al. 2010) (http://www.atgc-montpellier.fr/phyml), with automatic model selection by SMS (Smart Model Selection algorithm) (Lefort et al. 2017) determined through Akaike selection criterion, and with aLRT SH-like support (Anisimova and Gascuel 2006). The resulting trees were edited in FigTree v. 1.4.3. (http://tree.bio.ed.ac.uk/software/figtree).

Three species delimitation methods were employed to confirm the assignment of specimens to particular MOTUs. bPTP (Zhang et al. 2013) is a tree-based method using the Poisson tree processes model to infer putative species boundaries on a given phylogenetic input tree. As input for the bPTP (https://species.h-its.org/ptp/), the inferred PhyML trees were used; MCMC (Markov Chain Monte Carlo) analyses were run for 10^6^ generations, with a thinning of 200 and burn-in proportion of 0.1; species delimitation was inferred by maximum likelihood solution.

ABGD (Puillandre et al. 2012) and ASAP (Puillandre et al. 2021) are distance-based methods that use threshold values for differentiation between inter- and intraspecific divergences. Both methods indicate the presence of a “barcode gap”, thus clustering the samples into putative MOTUs within partitions. ASAP additionally ranks the partitions according to an ad-hoc score computed using the probabilities of groups to be panmictic species and the barcode gap widths (Puillandre et al. 2021). ABGD and ASAP were performed online (https://bioinfo.mnhn.fr/abi/public/abgd/abgdweb.html and https://bioinfo.mnhn.fr/abi/public/asap/asapweb.html, respectively) under default parameters, based on *p*-distances.

## ﻿Results

In this study, 43 of the 78 Croatian species of horseflies (55%), belonging to the subfamilies Chrysopsinae (genera *Chrysops* and *Silvius*) and Tabaninae (genera *Tabanus*, *Hybomitra*, *Atylotus*, *Haematopota*, *Dasyrhamphis*, *Philipomyia*, and *Heptatoma*), were morphologically identified and their identification was checked by DNA barcoding and species delimitation methods (Table 1). Most of the analysed species belong to the genus *Tabanus* (19 out of 30 recorded species), followed by *Hybomitra* (seven of 17), *Haematopota* (six of nine), *Chrysops* (four of seven), *Atylotus* (two of five), *Philipomyia* (two of two), *Silvius* (one of two), *Heptatoma* (one of one), and *Dasyrhamphis* (one of three species) (Table 1). No species for the genus *Therioplectes* (zero of two recorded) were analysed.

The results of the BOLD identification tool are shown in Table 1. Most of the newly sequenced records have species-level matches in BOLD, but among the matched sequences many are private and therefore not available for further analysis. Among our specimens, 16 have no species-level matches to either public or private data in BOLD and, thus, constitute new BINs (Barcode Index Numbers, roughly corresponding to MOTUs) in BOLD. These are *Dasyrhamphisumbrinus* (Meigen, 1820), *Haematopotagrandis* Meigen, 1820, *Ha.pandazisi* (Kröber, 1936), *Ha.subcylindrica* Pandellé 1883, *Hybomitraacuminata* (Loew, 1858), *H.pilosa* (Loew, 1858), *H.solstitialis* (Meigen, 1820) nec Lyneborg (1959), *Tabanusbifarius* Loew, 1858, *T.briani* Leclercq, 1962, *T.darimonti* Leclercq, 1964, *T.eggeri* Schiner, 1868, *T.exclusus* Pandellé, 1883, *T.lunatus* Fabricius, 1794, *T.miki* Brauer in Brauer & Bergenstamm, 1880, *T.quatuornotatus* Meigen, 1820, and *T.shannonellus* (Kröber, 1936). For 17 additional MOTUs, no public data are available in BOLD (as of July 2021), so these sequences represent the first public entries for the respective species. These are *Atylotusloewianus* (Villeneuve, 1920), *A.rusticus* (Linnaeus, 1767), *C.parallelogrammus* Zeller, 1842, *C.relictus* Meigen, 1820, *Silviusalpinus* (Scopoli, 1763), *Ha.scutellata* (Olsufjev, Moucha & Chvála, 1964), *Heptatomapellucens* (Fabricius, 1776), *H.distinguenda* (Verrall, 1909), *H.muehlfeldi* (Brauer in Brauer & Bergenstamm, 1880), *Philipomyiaaprica* (Meigen, 1820), *P.graeca* (Fabricius, 1794), *T.autumnalis* Linnaeus, 1761, *T.cordiger* Meigen, 1820, *T.glaucopis* Meigen, 1820, *T.spodopterus* Meigen, 1820, *T.sudeticus* Zeller, 1842, and *T.tergestinus* Egger, 1859. Three species of the tribe Diachlorini, sequenced here (*D.umbrinus*, *P.aprica*, and *P.graeca*) represent the first records for this tribe deposited in the BOLD database.

It must be emphasized that under the names of some of these species there are previous BOLD records with sequences that do not match our new entries (i.e., they are classified in separate BOLDBINs, e.g., for *H.solstitialis* and *Ha.pandazisi*), most likely due to misidentification or possible species complexes.

**Figure 2.**
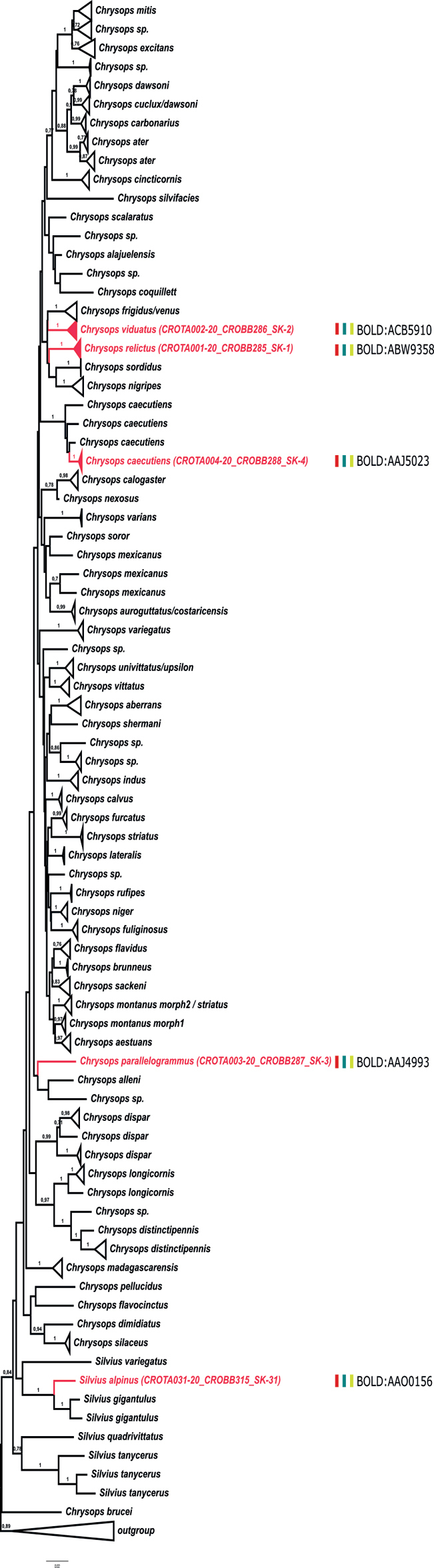
Maximum likelihood (ML) phylogenetic tree for the tribe Chrysopsini based on COI sequences of specimens sampled in this work and congeneric sequences from BOLD database of public records. The clades corresponding to MOTUs (as determined by species delimitation methods) are collapsed for simplicity; numbers on the nodes denote ML aLRT support (values lower than 0.70 are not shown). MOTUs containing sequences obtained in this study are marked in red; the results of the species delineation methods for the newly sequenced samples are presented as vertical bars beside the respective MOTU clades (bPTP in red; ABGD in green; ASAP in yellow; classification into BOLDBINs as assigned by BIN-RESL).

**Figure 3.**
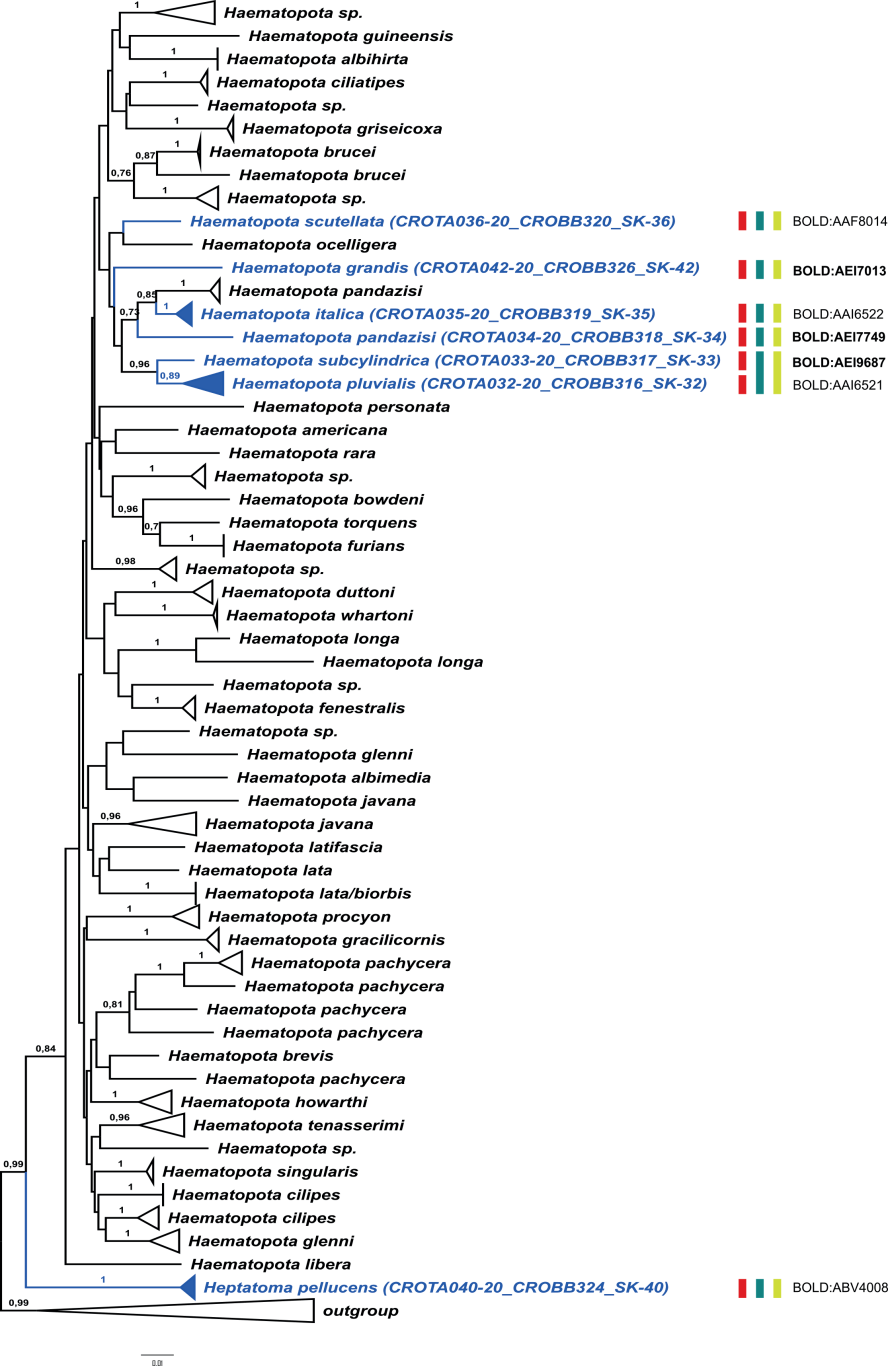
ML phylogenetic tree for the tribes Haematopotini and Heptatomini based on COI sequences of specimens sampled in this work and congeneric sequences from BOLD database of public records. The clades corresponding to MOTUs (as determined by species delimitation methods) are collapsed for simplicity; numbers on the nodes denote ML aLRT support (values lower than 0.70 are not shown). MOTUs containing sequences obtained in this study are marked in blue; the results of the species delineation methods for the newly sequenced samples are presented as vertical bars beside the respective MOTU clades (bPTP in red; ABGD in green; ASAP in yellow; classification into BOLDBINs as assigned by BIN-RESL, with newly established BINs marked in bold font).

**Figure 4.**
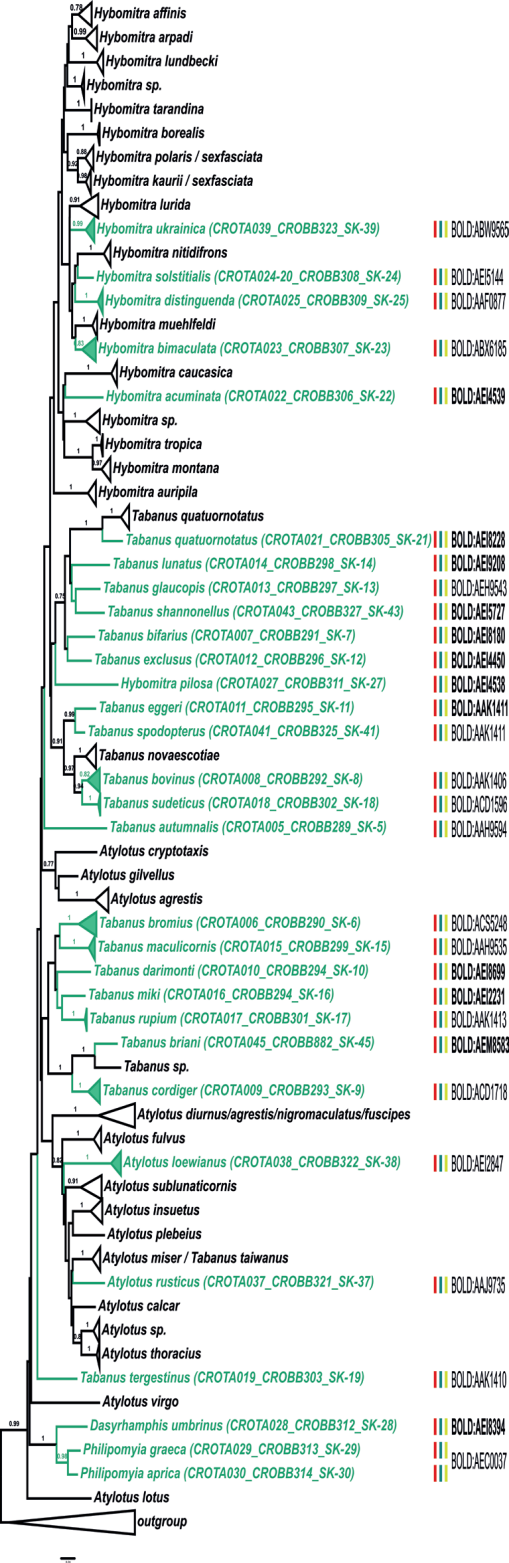
ML phylogenetic tree for the tribes Tabanini and Diachlorini based on COI sequences of specimens sampled in this work and congeneric sequences from BOLD database of public records. The clades corresponding to MOTUs (as determined by species delimitation methods) are collapsed for simplicity; numbers on the nodes denote ML aLRT support (values < 0.70 are not shown). MOTUs containing sequences obtained in this study are marked in green; the results of the species delineation methods for the newly sequenced samples are presented as vertical bars beside the respective MOTU clades (bPTP in red; ABGD in green; ASAP in yellow; classification into BOLDBINs as assigned by BIN-RESL, with newly established BINs marked in bold font).

On the other hand, several of our records match the data from BOLD, but these matched sequences were deposited under different species names. For example, our sample of *T.bovinus* Linnaeus, 1758 matches a public BOLD record deposited as *T.sudeticus*, while our sample of *T.sudeticus* matches a public BOLD record of *T.bovinus*; however, the private BOLD database contains high-score hit sequences stored under the matching names for both of our samples (*T.bovinus* and *T.sudeticus*). Our sample of *H.ukrainica* (Olsufjev, 1952) matches several samples deposited in BOLD under the name *H.solstitialis*. Another example is the case of species *P.aprica* and *P.graeca*, for which there are no high-score matches in the public BOLD database, but which have high-score matches with *T.bovinus* from private BOLD records. These cases most likely represent misidentifications of the specimens deposited in BOLD. The results of species delimitation methods for the three datasets are presented in Figs 2–4 and in Suppl. materials 3–5.

The bPTP, ABGD, and ASAP species delimitation methods yielded mostly concordant results, in turn in agreement with BIN-RESL classifications of our newly sequenced specimens (marked on Figs 2–4), with only a few exceptions (namely, for the species pairs *Ha.pluvialis* (Linnaeus, 1758) / *Ha.subcylindrica* and *P.aprica* / *P.graeca*). Most of our records are placed within conspecific MOTUs, except for those that represent new BOLD species entries and therefore form separate MOTUs / BINs (Figs 2–4; Suppl. materials 3–5). In addition, the exceptions are also our samples of *T.quatuornotatus* and *Ha.pandazisi*, which constitute MOTUs / BINs separate from other samples of these two species from BOLD. The MOTUs determined by species delimitation methods are highly supported in the phylogenetic trees, but the relationships between the MOTUs are largely unresolved and generally have much lower support values. In addition, there are cases of inconsistency between formal species designations and positioning within MOTUs. In all three datasets, some of the species are split into more than one MOTU (for example *A.agrestis* (Wiedemann, 1828), *C.caecutiens* (Linnaeus, 1758), *Ha.pandazisi*, *T.bromius* Linnaeus, 1758, *T.quatuornotatus*, etc.). In addition, several MOTUs contain samples that are designated by different names, for example a clade containing samples of *A.agrestis*, *A.diurnus* (Walker, 1850), and *A.nigromaculatus* Ricardo, 1900, or a clade composed of *T.taiwanus* Hayakawa & Takahasi, 1983 and *A.miser* (Szilády, 1915). These cases represent discordant BINs.

All genera within the tribes Chrysopsini and Tabanini appear to be paraphyletic, as well as the tribe Tabanini with respect to the tribe Diachlorini (although with low support).

The range of *p*-distances within MOTUs is 0–2.1%, while the range of those among MOTUs within the tribes is 1.7–14%. The highest intraspecific value of *p*-distances is observed for the species *T.bromius* (2.1%), while the lowest interspecific *p*-distances are recorded for the species pairs *Ha.pluvialis* / *Ha.subcylindrica* (1.8–2.7%) and *P.aprica* / *P.graeca* (1.7%).

## ﻿Discussion

Studies on vector ecology are crucial for understanding, predicting, and controlling insect-borne diseases. In that instance, national collections of DNA barcodes are particularly useful in cases of medically or veterinary, as well as economically important species. For example, in the recently published DNA barcode collection of German Diptera (Morinière et al. 2019), the authors emphasized the importance of monitoring vector species by metabarcoding. However, COI barcodes for only about 550 out of more than 4400 described tabanid species are presently available in BOLD, and relatively few molecular-level studies have been conducted on the European horsefly fauna (Morinière et al. 2019). Regarding the horsefly species from Croatia, no DNA barcoding data have been available in BOLD up to date.

The high degree of variability in the colouration of the frontal calli, antennae, notopleural lobes, legs, and abdomen in some horsefly species very often leads to confusion, making correct identification very difficult. In addition, when specimens are old and damaged or missing parts such as antennae, legs, palpi, or hairs on eyes, identification is even more difficult, and errors are quite common. Such misidentifications can also cause incorrect record entries into public databases such as NCBI and BOLD, as we observed in this study. The use of new literature data and DNA sequencing is therefore important for correct insect taxonomy and systematic studies, in order to avoid errors in species identification (Schmidt and Polaszek 2007).

Most European horsefly species belong to the tribe Tabanini, and their representatives are found in all terrestrial zoogeographical regions except those permanently covered with ice (Chvála et al. 1972; Trojan 2001). Within this tribe, the genus *Tabanus* in Europe comprises 4% of all known *Tabanus* species worldwide (Chvála et al. 1972), and it is represented in Croatian horsefly fauna by about 38% of all recorded species. The taxonomy of this genus is quite complex. It is divided into several groups of species according to their morphological characteristics; separation is based mainly on female morphology (Chvála et al. 1972; Suppl. material 1). Some species described as *Tabanus* in the older literature are now referred to other genera (Chvála et al. 1972; Andreeva 2004). For example, the tribe Diachlorini includes several European species of the genus *Philipomyia*, and some genera from the Neotropics have been synonymized with *Tabanus* (Chvála et al. 1972). These taxonomic changes often lead to confusion in tabanid nomenclature, especially among researchers who study medical significance or vector role of this family. A recent study (Werszko et al. 2020) reported vector horsefly species *T.distinguendus* Verrall, 1909 and *T.apricus* Meigen, 1820, although the valid name for *T.distinguendus* is *H.distinguenda* (Verrall, 1909) and that for *T.apricus* is *P.aprica*. Another study, reporting skin lesions caused by bites of *T.bovinus* in Bolivia (Veraldi and Esposito 2017), caught the attention of Dr Stephen M. Smith who reported that the skin damage could not have been caused by bites from *T.bovinus*, since the species does not belong to the Neotropical fauna (Smith 2018). Probably a very high morphological similarity between the Palaearctic species *T.bovinus* and some Neotropical species from the genus *Tabanus* was the reason for these errors. In the European fauna, *T.bovinus* and *T.sudeticus* are morphologically very similar, which often leads to erroneous identification, especially when performed by non-specialists.

In the present study, the COI barcoding region was used for species confirmation. The sequence data enabled us to unequivocally identify some of the horsefly species which are morphologically very similar, for instance, the already mentioned *T.bovinus* and *T.sudeticus*, but also some others, like *H.solstitialis* / *H.ukrainica* and *T.maculicornis* / *T.bromius* (mentioned in Suppl. material 1). In the Palaearctic region, identification of species from the genus *Hybomitra* is often difficult due to variability in colouration and uncertainty about structural features (Zeegers 2018; see Suppl. material 1). When the occurrence of *H.ukrainica* in the Croatian fauna was first reported (Krčmar and Majer 1994), a revision of the horsefly collections in Croatian Natural History Museum in Zagreb in 2001 revealed that this species had already been collected in Croatia in 1982, 1987, 1988, and 1989 (Krčmar and Mikuska 2001) but had been incorrectly identified as *H.solstitialis*, formerly called *H.ciureai* (Séguy, 1937). Since the 1950s, the name *H.solstitialis* has been misinterpreted in the European literature, although *H.ciureai* was originally described as a variety of *H.solstitialis* (Zeegers 2018). Despite well-illustrated morphological characters relevant for identification of *H.ukrainica* and *H.ciureai* (now *H.solstitialis*) (Mally 1985), mistakes are still possible due to high morphological similarity. Therefore, for the identification of these two species belonging to “*bimaculata*” group, molecular analyses are necessary. The results of our analyses confirm a significant genetic divergence in the standard COI barcoding region between these two species (*H.solstitialis* and *H.ukrainica*) and prove that DNA barcoding is informative enough for their unequivocal distinction. Moreover, our results reveal high levels of genetic variability within some of the currently recognized tabanid taxa, reflected through their splitting in multiple separate MOTUs. This finding is also consistent with previous studies (Morita et al. 2016; Morinière et al. 2019) and indicates the possibility of cryptic diversity or the existence of species complexes not yet recognized. In that context, it is interesting to note that our specimens of *T.quatuornotatus* and *Ha.pandazisi* constitute new BINs in BOLD, genetically diverged from other samples denoted with the same names. Other evident examples are *A.agrestis*, which appears in several clearly separated OTUs scattered in the phylogenetic tree, or several species of *Chrysops*, which appear in multiple OTUs each.

On the other hand, our data suggests high genetic similarity in the DNA barcoding region between some of the species, leading to incongruence between the results of the different species delimitation methods. For example, the range of *p*-distances for *Ha.pluvialis* and *Ha.subcylindrica* is 1.8–2.7%, and both the BIN-RESL algorithm and bPTP recognized them as separate MOTUs. In contrast, the ABGD and ASAP methods recovered these two species in a single MOTU (Fig. 3). *Ha.pluvialis* is very similar to *Ha.subcylindrica* so the identification and separation of these two species is sometimes very difficult on morphological basis (see Suppl. material 1). A similar situation is observed for *P.aprica* and *P.graeca*. These two species are mainly distinguished by the colouration of the abdomen and the shape and colouration of antennae. The inferred *p*-distances for these two species (1.7%) are below the standard threshold for BIN separation in BOLD and they have been classified within the same BIN; however, species delimitation methods bPTP, ABGD, and ASAP all recovered them in two separate MOTUs (Fig. 4). Besides a high degree of morphological similarity, the species within each of these two species pairs (*Ha.pluvialis* / *Ha.subcylindrica* and *P.aprica* / *P.graeca*) also share some ecological characteristics: they inhabit the same regions, occur at the same time, and their flight period is almost the same; however, *P.aprica* prefers mountainous habitats. Therefore, the high genetic similarity of the species within these two species pairs could also be indicative of their close evolutionary relationship. Other than in these two cases of high genetic similarity which caused the incongruence between various species-delimitation methods, other investigated MOTUs are clearly distinguished through non-overlapping low intraspecific and high interspecific variability, indicating the existence of a barcoding gap which is a prerequisite for efficient and reliable DNA barcoding (Hebert et al. 2003).

Analyses of various molecular markers have been used worldwide in the study of the taxonomy, phylogeography and phylogenetics of horseflies (Sofield et al. 1984; Wiegmann et al. 2000; Iranpour et al. 2004; Cywinska et al. 2010; Morita et al. 2016; Sanal Demirci et al. 2021); however, many phylogenetic aspects of this family still remain unclear (Morita et al. 2016; Votýpka et al. 2019). One of the most comprehensive studies of phylogenetic relationships within the family Tabanidae and its subfamilies and tribes was conducted using several genetic markers: mitochondrial COI, nuclear 28S rRNA, CAD (carbamoyl-phosphate synthetase 2, aspartate transcarbamylase and dihydroorotase), and AATS (alanyl-tRNA synthetase) (Morita et al. 2016). The authors reported that the classification of Tabanidae should be reconsidered based on the new data obtained by molecular techniques. Their main finding was the paraphyly of the Chrysopsinae, as already proposed by Mackerras (1954). However, the tribes Pangoniini and Tabanini could also be paraphyletic, although the support for this is weak. In addition, most genera within the tribes Chrysopsini and Tabanini appear to be paraphyletic. For example, the large genus *Tabanus*, which is one of the most diversified genera in Diptera, is most likely paraphyletic with respect to several other genera (Morita et al. 2016).

Although our analyses are based on a single mitochondrial locus, most of the results are consistent with previous studies (Morita et al. 2016; Changbunjong et al. 2018). Analysis of the tribes Tabanini and Diachlorini resulted in Diachlorini embedded within the Tabanini, with *A.lotus* Burton, 1978 positioned as basal to all other Tabanini and Diachlorini, suggesting the paraphyly of the tribe Tabanini. In addition, all genera within the tribes Chrysopsini and Tabanini—*Chrysops* and *Silvius* in Chrysopsinae, and *Hybomitra*, *Atylotus*, and *Tabanus* in Tabaninae—appear to be mutually paraphyletic. However, most of these results have low support, similar to previous studies, and further work is required to obtain useful data for correct classification.

Molecular markers for phylogenetic studies of horseflies in Croatian fauna were first used in 2006 (Biruš 2006). In that study, the phylogenetic relationships between several species of the horsefly genera *Philipomyia*, *Dasyrhamphis* (both from the tribe Diachlorini), and *Tabanus* (from the tribe Tabanini) were investigated using 16S rRNA and COI mitochondrial genes. The sequence-based phylogenetic data supported the recent morphology-based classification of two horsefly species, *P.aprica* and *P.graeca*, within the new genus *Philipomyia* (these two species were previously classified in the genus *Tabanus*) and the placement of this genus in the tribe Diachlorini rather than in the tribe Tabanini (Biruš 2006). Our results are consistent with these data: both species of *Philipomyia* are positioned in a highly supported monophyletic clade along with *D.umbrinus*.

Among all representatives of the subfamily Tabaninae, *He.pellucens* is the only species with a unique morphological feature, a totally bare subcostal vein (Trojan 1994). Moreover, the differences in larval forms between species of the genera *Heptatoma* and *Haematopota* confirm the necessary separation of the genus *Heptatoma* into a separate tribe (Andreeva 2004). The results of our analysis also support that conclusion, with *Heptatoma* being positioned basally to all species of Haematopotini, thus rendering the former tribe monophyletic.

## ﻿Conclusion

In this paper, 55% of the horsefly species known from Croatia were analysed by DNA barcoding, which proved to be an effective tool for their identification. The obtained data provide a basis for a reference library of Tabanidae DNA barcodes for the investigated region and contribute to BOLD through the addition of 16 new BINs. The presented results also indicate some inconsistencies in the taxonomy and systematics of horseflies, mostly in line with previous systematic studies. However, considering the shortcomings of a single-locus approach for systematic studies, a more comprehensive integrative approach covering a broader range of taxa and additional molecular markers is needed to address these issues.

## Supplementary materials

Supplementary material 1 Morphological characteristics of females of some horseflies from subfamily Tabaninae and Chrysopsinae Data type pdf file Explanation note Morphological characteristics of females of some horseflies from subfamily Tabaninae and Chrysopsinae, important for species identification (presented according to Chvála et al. (1972) and Zeegers (2018)). https://binary.pensoft.net/file/649953This dataset is made available under the Open Database License (http://opendatacommons.org/licenses/odbl/1.0/). The Open Database License (ODbL) is a license agreement intended to allow users to freely share, modify, and use this Dataset while maintaining this same freedom for others, provided that the original source and author(s) are credited.Author: Stjepan Krčmar

Supplementary material 2 COI multiple sequence alignments Data type docx file Explanation note MAFFT multiple alignments of COI nucleotide sequences (in fasta format) for the tree datasets: 1. Chrysopsini; 2. Haematopotini and Heptatomini; 3. Tabanini and Diachlorini. https://binary.pensoft.net/file/649954This dataset is made available under the Open Database License (http://opendatacommons.org/licenses/odbl/1.0/). The Open Database License (ODbL) is a license agreement intended to allow users to freely share, modify, and use this Dataset while maintaining this same freedom for others, provided that the original source and author(s) are credited.Author: Branka Bruvo Mađarić

Supplementary material 3 Figure S1. NJ tree for the tribe Chrysopsini Data type pdf file https://binary.pensoft.net/file/649955This dataset is made available under the Open Database License (http://opendatacommons.org/licenses/odbl/1.0/). The Open Database License (ODbL) is a license agreement intended to allow users to freely share, modify, and use this Dataset while maintaining this same freedom for others, provided that the original source and author(s) are credited.Author: Branka Bruvo Mađarić

Supplementary material 4 Figure S2. NJ tree for the tribes Haematopotini and Heptatomini Data type pdf file https://binary.pensoft.net/file/649956This dataset is made available under the Open Database License (http://opendatacommons.org/licenses/odbl/1.0/). The Open Database License (ODbL) is a license agreement intended to allow users to freely share, modify, and use this Dataset while maintaining this same freedom for others, provided that the original source and author(s) are credited.Author: Branka Bruvo Mađarić

Supplementary material 5 Figure S3. NJ tree for the tribes Tabanini and Diachlorini Data type pdf file https://binary.pensoft.net/file/649957This dataset is made available under the Open Database License (http://opendatacommons.org/licenses/odbl/1.0/). The Open Database License (ODbL) is a license agreement intended to allow users to freely share, modify, and use this Dataset while maintaining this same freedom for others, provided that the original source and author(s) are credited.Author: Branka Bruvo Mađarić
