# Supplementary material for: ﻿DNA barcoding of the horsefly fauna (Diptera, Tabanidae) of Croatia with notes on the morphology and taxonomy of selected species from Chrysopsinae and Tabaninae
Source: Zookeys. 2022 Feb 23;1087:141–61. doi: 10.3897/zookeys.1087.78707 (PMC8891235; doi:10.3897/zookeys.1087.78707)
Supplement: Supplementary material 1 — Morphological characteristics of females of some horseflies from subfamily Tabaninae and Chrysopsinae [file zookeys-1087-141-s001.pdf]

Morphological characteristics of females of some horseflies from subfamily Tabaninae and Chrysopsinae, important for species identification (presented according to Chvála et al. (1972) and Zeegers (2018))

Genus *Tabanus*

The genus *Tabanus* is represented in Croatian horsefly fauna by about 38% of all described species. This genus is divided into several groups of species according to their morphological characteristics; separation is based mainly on female morphology. In the "*glaucopis*" group of species, specimens of *Tabanus glaucopis* Meigen, 1820 and *T. shannonellus* Kröber, 1936 are rather similar, especially when the whitish scutellum and the enlarged median triangles on tergites 3 to 5 in *T. shannonellus* are not clearly distinct. Very high variability in colouration of subcallus and colouration and shape of frontal calli, as well as the abdominal colouration in *T. glaucopis,* sometimes cause misidentifications. The "*bromius*" group of species generally consists of medium-sized species (11 mm to 18 mm) from which *T. maculicornis* Zetterstedt, 1842 and *T. bromius* L., 1758 highly resemble each other. The main morphological characteristic for their correct identification is the width of the postocular margin on the vertex. The largest species from the genus *Tabanus* (25 mm to 27 mm) are classified in the "*bovinus*" group. In this group of species *T. bovinus* L., 1758 and *T. sudeticus* Zeller, 1842 are morphologically similar since both species are large and blackish brown. The main morphological features for distinguishing these two species are the typical yellow posterior border in all tergites and sternites, as well as the appearance and colouration of antennae and palpi. Other 13 analysed species from the genus *Tabanus* (Table 1) are clearly distinguishable based on their morphological characteristics.

Genus *Hybomitra*

In the Palaearctic region, identification of species from the genus *Hybomitra* is often difficult due to variability in colouration and uncertainty about structural features (Zeegers 2018). In different wet habitats in Croatia, five species of the genus, *Hybomitra bimaculata* (Macquart, 1826), *H. solstitialis* (Meigen, 1820) nec Lyneborg (1959), *H. distinguenda* (Verrall, 1909), *H. muehlfeldi* (Brauer *in* Brauer and Bergenstamm, 1880) and *H. ukrainica* (Olsufjev, 1952) are the most common ones. These species are morphologically very similar, and often their identification is almost impossible without a detailed analysis of the cerci and subgenital plate of the female genitalia. All five species belong to the "*bimaculata*" group of species. The other two analysed species from this genus, *H.*

*acuminata* and *Hybomitra pilosa*, are clearly distinguishable based on their morphological characteristics.

Genus *Haematopota*

*Haematopota pluvialis* (L., 1758), the most common species among all horseflies recorded in Europe, is very similar to *Ha. subcylindrica* Pandellé, 1883. Moreover, some of the *Haematopota* species are very variable and some of the described taxa represent only varieties of well-known and common species (Chvála et al. 1972). The Palaearctic species of the genus *Haematopota* are separated into the groups "*pluvialis*" and "*italica*". The main morphological feature that distinguishes *Ha. pluvialis* from other species in the "*pluvialis*" group is the shape of the first segment of antennae, always with a deep constriction before the tip, which gives an irregular shape to this segment. The first segments of the antennae often have a slight constriction before the tip also in *Ha. subcylindrica* and in *Ha. scutellata* (Olsufjev, Moucha & Chvála, 1964). However, a large whitish-grey patch on the scutellum in *Ha. scutellata* allows for secure identification and separation from closely related species. Concerning the great variability of *Ha. pluvialis* in shape and colouration of antennae, the identification and separation from *Ha. subcylindrica* is sometimes very difficult only on morphological basis. The other three analysed species from this genus (Table 1) are easily distinguished according to their morphological characteristics.

Other genera

The Croatian fauna of the genus *Chrysops* is represented by seven species, with a variable area of distribution. According to their morphological characteristics, these seven species are separated into the following groups: "*sepulcralis*", "*relictus*", "*rufipes*" and "*italicus*". Among the four species analysed in this study, *Chrysops caecutiens* (L., 1758) belongs to the "*sepulcralis*" group, while *C. relictus* Meigen, 1820, *C. parallelogrammus* Zeller, 1842 and *C. viduatus* (Fabricius, 1794) belong to the "*relictus*" group. Females of these species are distinguished mainly by the shape of the black patch on the yellowish second tergite. In this study, the two species of the genus *Atylotus* were clearly identified based on their morphological characteristics. *Atylotus loewianus* (Villeneuve, 1920) is a medium-sized species (13,5 mm to 16 mm) from golden yellow to yellowish-brown, while *A. rusticus* (L., 1767) is a greyish species. One female specimen was analysed from each of the genera *Silvius*, *Heptatoma*, and *Dasyrhamphis*. The genus *Silvius* occurs in Croatia with two species, of which *Silvius alpinus* (Scopoli, 1763) is widely distributed in different habitats. The genus *Heptatoma* is monotypic, with only one species known, *Heptatoma pellucens* (Fabricius, 1776)

distributed throughout most of Europe, while the genus *Dasyrhamphis* is represented by three species, among which only *Dasyrhamphis umbrinus* (Meigen, 1820) was analysed in this study.

## References


Chvála M, Lyneborg L, Moucha J (1972) The horse flies of Europe (Diptera, Tabanidae). Entomological Society of Copenhagen, Copenhagen.

Zeegers T (2018) A new synonymy in the horsefly genus *Hybomitra* (Diptera: Tabanidae). Nederlandse Faunistische Mededelingen 50: 89–92.
