# Supplementary material for: ﻿DNA barcoding of the horsefly fauna (Diptera, Tabanidae) of Croatia with notes on the morphology and taxonomy of selected species from Chrysopsinae and Tabaninae
Source: Zookeys. 2022 Feb 23;1087:141–61. doi: 10.3897/zookeys.1087.78707 (PMC8891235; doi:10.3897/zookeys.1087.78707)
Supplement: Supplementary material 2 — COI multiple sequence alignments [file zookeys-1087-141-s002.docx]

1. CHRYSOPSINI

>ACT022-07|Chrysops univittatus|COI-5P|KM285533

----------------------------------------ggaacatctttaagtatttt

aattcgtgctgaactaggtcacccaggagccttaattggtgatgatcaaatttataatgt

aattgtaactgctcatgcattcgttataattttctttatagtaatacctattataattgg

aggattcggaaattgattagttccattaatattaggagcacctgatatagcattccctcg

aataaataatataagtttttgactactacctccttcattaacccttctattagttagaag

tatagttgaaaatggagctggaactggttgaactgtataccccccattatcagctgctat

tgctcatggaggaggatcagtagatttagcaattttttctttacacttagccggaatttc

atcaattttaggagctgtaaattttattacaacagtaattaatatacgatcaacaggaat

tacatttgatcgaatacctttatttgtttgagctgttgttattactgctattcttctttt

attatctttacctgttttagctggrgcaattactatattattaacagatcgaaatttaaa

tacttctttttttgacccagctggaggaggagaccctattttataccaacatctatct

>ACT006-06|Chrysops univittatus|COI-5P|KM285536

----------------------------------------ggaacatctttaagtatttt

aattcgtgctgaactaggtcacccaggagccttaattggtgatgatcaaatttataatgt

aattgtaactgctcatgcattcgttataattttctttatagtaatacctattataattgg

aggattcggaaattgattagttccattaatattaggagcacctgatatagcattccctcg

aataaataatataagtttttgactactacctccttcattaacccttctattagttagaag

tatagttgaaaatggagctggaactggttgaactgtataccccccattatcagctgctat

tgctcatggaggaggatcagtagatttagcaattttttctttacacttagccggaatttc

atcaattttaggagctgtaaattttattacaacagtaattaatatacgatcaacaggaat

tacatttgatcgaatacctttatttgtttgagctgttgttattactgctattcttctttt

attatctttacctgttttagctggagcaattactatattattaacagatcgaaatttaaa

tacttctttttttgacccagctggaggaggagaccctattttataccaacatctatct

>ACT009-06|Chrysops univittatus|COI-5P|KM285535

----------------------------------------ggaacatctttaagtatttt

aattcgtgctgaactaggtcacccaggagccttaattggtgatgatcaaatttataatgt

aattgtaactgctcatgcattcgttataattttctttatagtaatacctattataattgg

aggattcggaaattgattagttccattaatattaggagcacctgatatagcattccctcg

aataaataatataagtttttgactactacctccttcattaacccttctattagttagaag

tatagttgaaaatggagctggaactggttgaactgtataccctccattatcagctgctat

tgctcatggaggaggatcagtagatttagcaattttttctttacacttagccggaatttc

atcaattttaggagctgtaaattttattacaacagtaattaatatacgatcaacaggaat

tacatttgatcgaatacctttatttgtttgagctgttgttattactgctattcttctttt

attatctttacctgttttagctggagcaattactatattattaacagatcgaaatttaaa

tacttctttttttgacccagctggaggaggagaccctattttataccaacatctatct

>MHTAB120-09|Chrysops univittatus|COI-5P|KM285538

----------------------------------------ggaacatctttaagtatttt

aattcgtgctgaactaggtcacccaggagccttaattggtgatgatcaaatttataatgt

aattgtaactgctcatgcattcgttataattttctttatagtaatacctattataattgg

aggatttggaaattgattagttccattaatattaggagcacctgatatagcattccctcg

aataaataatataagtttttgactactacctccttcattaacccttctattagttagaag

tataattgaaaatggagctggaactggttgaactgtataccccccattatcagctgctat

tgctcatggaggaggatcagtagatttagcaattttttctttacacttagccggaatttc

atcaattttaggagctgtaaattttattacaacagtaattaatatacgatcaacaggaat

tacatttgatcgaatacctttatttgtttgagctgttgttattactgctattcttctttt

attatctttacctgttttagctggagcaattactatattattaacagatcgaaatttaaa

tacttctttttttgacccagctggaggaggagaccctattttataccaacatctattc

>OPPQG340-17|Chrysops|COI-5P

aactctctattttattttcggagcatgagccggaataattggaacatctttaagtatttt

aattcgtgctgaactaggtcacccaggagccttaattggtgatgatcaaatttataatgt

aattgtaactgctcatgcattcgttataattttctttatagtaatacctattataattgg

aggattcggaaattgattagttccattaatattaggagcacctgatatagcattccctcg

aataaataatataagtttttgactactacctccttcattaacccttctattagttagaag

tatagttgaaaatggagctggaactggttgaactgtataccccccattatcagctgctat

tgctcatggaggaggatcagtagatttagcaattttttctttacacttagccggaatttc

atcaattttaggagctgtaaattttattacaacagtaattaatatacgatcaacaggaat

tacatttgatcgaatacctttatttgtttgagctgttgttattactgctattcttctttt

attatctttacctgttttagctggagcaattactatattattaacagatcgaaatttaaa

tacttctttttttgacccagctggaggaggagaccctattttataccaacatctattc

>ASDMT1098-11|Chrysops|COI-5P|MG169612

aactctctattttattttcggggcatgagccggaataattggaacatctttaagtatttt

aattcgtgctgaactaggtcacccaggagccttaattggtgatgatcaaatttataatgt

aattgtaactgctcatgcattcgttataattttctttatagtaatacctattataattgg

aggattcggaaattgattagttccattaatattaggagcacctgatatagcattccctcg

aataaataatataagtttttgactactacctccttcattaacccttctattagttagaag

tatagttgaaaatggggctggaactggttgaactgtataccctccattatcagctgctat

tgctcatggaggaggatcagtagatttagcaattttttctttacacttagccggaatttc

atcaattttaggagctgtaaattttattacaacagtaattaatatacgatcaacaggaat

tacatttgatcgaatacctttatttgtttgagctgttgttattactgctattcttctttt

attatctttacctgttttagctggggcaattactatattattaacagatcgaaatttaaa

tacttctttttttgacccagctggaggaggagaccctattttataccaacatctattc

>GBDP15883-15|Chrysops upsilon|COI-5P|KM243518

aactctctattttattttcggagcgtgagccggaataattggaacatctttaagtatttt

aattcgtgctgaactaggtcacccaggagccttaattggtgatgatcaaatttataatgt

aattgtaactgctcatgcattcgttataattttctttatagtaatacctattataattgg

aggattcggaaattgattagttccattaatattaggagcacctgatatagcattccctcg

aataaataatataagtttttgactactacctccttcattaacccttctattagttagaag

tatagttgaaaatggagctggaactggttgaactgtatatcccccattatcagctgctat

tgctcatggaggaggatcagtagatttagcaattttttctttacacttagctggaatttc

atcaattttaggggctgtaaattttattacaacagtaattaatatacgatcaacaggaat

tacatttgatcgaatacctttatttgtttgagctgttgttattactgctattcttctttt

attatctttacctgttttagctggagcaattactatattattaacagatcgaaatttaaa

tacttctttttttgatccagctggaggaggagaccctattttataccaacatctattc

>ACT027-07|Chrysops vittatus|COI-5P|KM285551

----------------------------------------ggaacatctttaagtatttt

aattcgtgctgaactaggtcacccaggagccttaattggtgatgatcaaatttataatgt

aattgtaactgctcatgcatttgttataattttctttatagtaatacctattataattgg

aggatttggaaattgattagttccattaatattaggagcacctgatatagcatttcctcg

aataaataatataagtttttgattactccccccttcattaactcttctattagttagaag

tatagttgaaaacggggctggaactggatgaactgtataccctccattatcagctgctat

tgctcatggaggaggatcagtagatttagcaattttttctttacatttagctggaatttc

atcaattttaggagctgtaaattttattacaacagtaattaatatacgatcaacaggaat

tacatttgaccgaatacctttatttgtttgagctgttgttattactgcaattctcctttt

attatctttacctgttttagctggagctattactatattattaacagatcgaaatttaaa

tacttctttctttgacccagctggaggaggagaccctattctataccaacatttatct

>ACT035-07|Chrysops vittatus|COI-5P|KM285555

----------------------------------------ggaacatctttaagtatttt

aattcgtgctgaactaggtcacccaggagccttaattggtgatgatcaaatttataatgt

aattgtaactgctcatgcatttgttataattttctttatagtaatacctattataattgg

aggatttggaaattgattagttccattaatattaggagcacctgatatagcatttcctcg

aataaataatataagtttttgattactccccccttcattaactcttttattagttagaag

tatagttgaaaacggggctggaactggatgaactgtataccctccattatcagctgctat

tgctcatggaggaggatcagtagatttagcaattttttctttacatttagctggaatttc

atcaattttaggagctgtaaattttattacaacagtaattaacatacgatcaacaggaat

tacatttgaccgaatacctttatttgtttgagctgttgttattactgcaattctcctttt

attatctttacctgttttagctggagctattactatattattaacagatcgaaatttaaa

tacttctttctttgacccagctggaggaggagaccctattttataccaacatttatct

>OPPEO6133-17|Chrysops vittatus|COI-5P

aactctctattttattttcggagcttgagctggaataattggaacatctttaagtatttt

aattcgtgctgaactaggtcacccaggagccttaattggtgatgatcaaatttataatgt

aattgtaactgctcatgcatttgttataattttctttatagtaatacctattataattgg

aggatttggaaattgattagttccattaatattaggagcacctgatatagcatttcctcg

aataaataatataagtttttgattactccccccttcattaactcttctattagttagaag

tatagttgaaaacggggctggaactggatgaactgtataccctccattatcagctgctat

tgctcatggaggaggatcagtagatttagcaattttttctttacatttagctggaatttc

atcaattttaggagctgtaaactttattacaacagtaattaatatacgatcaacaggaat

tacatttgaccgaatacctttatttgtttgagctgttgttattactgcaattctcctttt

attatctttacctgttttagctggagctattactatattattaacagatcgaaatttaaa

tacttctttctttgacccagctggaggaggagaccctattctataccaacatttattc

>BBDED136-10|Chrysops vittatus|COI-5P|HM883105

aactctctattttattttcggagcttgagctggaataattggaacatctttaagtatctt

aattcgtgctgaactaggtcacccaggagccttaattggtgatgatcaaatttataatgt

aattgtaactgctcatgcatttgttataattttctttatagtaatacctattataattgg

aggatttggaaattgattagttccattaatattaggagcacctgatatagcatttcctcg

aataaataatataagtttttgattactccccccttcattaactcttttattagttagaag

tatagttgaaaacggggctggaactggatgaactgtataccctccattatcagctgctat

tgctcatggaggaggatcagtagatttagcaattttttctttacatttagctggaatttc

atcaattttaggagctgtaaattttattacaacagtaattaacatacgatcaacaggaat

tacatttgaccgaatacctttatttgtttgagctgttgttattactgcaattctcctttt

attatctttacctgttttagctggagctattactatattattaacagatcgaaatttaaa

tacttctttctttgacccagctggaggaggagatcctattttataccaacatttattc

>CNROQ423-13|Chrysops vittatus|COI-5P|KR388640

aactctctattttattttcggagcttgagctggaataattggaacatctttaagtatttt

aattcgtgctgaactaggtcacccaggagccttaattggtgatgatcaaatttataatgt

aattgtaactgctcatgcatttgttataattttctttatagtaatacctattataattgg

aggatttggaaattgattagttccattaatattaggagcacctgatatagcatttcctcg

aataaataatataagtttttgattactccccccttcattaactcttctattagttagaag

tatagttgaaaacggagctggaactggatgaactgtataccctccattatcagctgctat

tgctcatggaggaggatcagtagatttagcaattttttctttacatttagctggaatttc

atcaattttaggagctgtaaattttattacaacagtaattaatatacgatcaacaggaat

tacatttgaccgaatacctttatttgtttgagctgttgttattactgcaattctcctttt

attatctttacctgttttagctggagctattactatattattaacagatcgaaatttaaa

t---------------------------------------------------------

>RRINV3861-15|Chrysops vittatus|COI-5P|KT707506

aactctctattttattttcggagcttgagctggaataattggaacatctttaagtatttt

aattcgtgctgaactaggtcacccaggagccttaattggtgatgatcaaatttataatgt

aattgtaactgctcatgcatttgttataattttctttatagtaatacctattataattgg

aggatttggaaattgattagttccattaatattaggagcacctgatatagcatttcctcg

aataaataatataagtttttgattactccccccttcattaactcttctattagttagaag

tatagttgaaaacggggctggaactggatgaactgtataccctccattatcagctgctat

tgctcatggaggaggatcagtagatttagcaattttttctttacatttagctggaatttc

atcaattttaggagctgtaaattttattacaacagtaattaatatacgatcaacaggaat

tacatttgaccgaatacctttatttgtttgagctgttgttattactgcaattctcctttt

attatctttacctgttttagcaggagctattactatattattaacagatcgaaatttaaa

tacttctttctttgacccagctggaggagg----------------------------

>CNKJF051-14|Chrysops vittatus|COI-5P|KR386873

-actctctattttattttcggagcttgagctggaataattggaacatctttaagaatttt

aattcgtgctgaactaggtcacccaggagccttaattggtgatgatcaaatttataatgt

aattgtaactgctcatgcatttgttataattttctttatagtaatacctattataattgg

aggatttggaaattgattagttccattaatattaggagcacctgatatagcattccctcg

aataaataatataagtttttgattactccccccttcattaactcttctattagttagaag

tatagttgaaaacggggctggaactggatgaactgtataccccccattatcagctgctat

tgctcatggaggagggtcagtagatttagcaattttttctttacatttagctggaatttc

atcaattttaggagctgtaaattttattacaacagtaattaatatacgatcaacaggaat

tacatttgaccgaatacctttatttgtttgagctgttgtcattactgcaattctcctttt

attatctttacctgttttagctggagctattactatattattaacagatcgaaatttaaa

t---------------------------------------------------------

>OPPEO6150-17|Chrysops aestuans|COI-5P

aactctctattttattttcggagcttgagctggaataattggaacatcattaagtatttt

aattcgtgctgaactaggtcatccaggagccttaattggtgacgatcaaatttataatgt

aattgtaactgctcatgcatttgttataattttctttatagtaatacctattataattgg

aggatttggaaattgattagttccattaatattaggagctcctgatatagcattccctcg

aataaataatataagtttttgattactacctccttcattaactcttctattagttagaag

tatagttgaaaatggagctggaactggatgaactgtatacccaccattatcagcagctat

tgctcatggaggaggatcagtagatttagcaattttttctttacatttagctggaatttc

atcaattttaggagctgtaaattttattacaacagtaattaatatacgatcaacaggaat

tacatttgatcgaataccattatttgtttgagctgttgttattactgctattcttctttt

attatccctacctgttttagctggagctattactatattattaacagatcgaaatttaaa

tacttctttctttgaccctgctggaggaggagacccaattttataccaacacttattc

>OPPEO6129-17|Chrysops aestuans|COI-5P

aactctctattttattttcggagcttgagctggaataattggaacatcattaagtatttt

aattcgtgctgaactaggtcatccaggagccttaattggtgacgatcaaatttataatgt

aattgtaactgctcatgcatttgttataattttctttatagtaatacctattataattgg

aggatttggaaattgattagttccattaatattaggagctcctgatatagcatttcctcg

aataaataatataagtttttgattactacctccttcattaactcttctattagttagaag

tatagttgaaaatggagctggaactggatgaactgtatacccaccattatcagcagctat

tgctcatggaggaggatcagtagatttagcaattttttctttacatttagctggaatttc

atcaattttaggagctgtaaattttattacaacagtaattaatatacgatcaacaggaat

tacatttgatcgaataccattatttgtttgagctgttgttattactgctattcttctttt

attatccctacctgttttagctggagctattactatattattaacagatcgaaatttaaa

tacttctttctttgatcctgctggaggaggagacccaattttataccaacacttattc

>OPPEO6128-17|Chrysops aestuans|COI-5P

aactctctattttattttcggagcttgagctggaataattggaacatcattaagtatttt

aattcgtgctgaactaggtcatccaggagccttaattggtgacgatcaaatttataatgt

aattgtaactgctcatgcatttgttataattttctttatagtaatacctattataattgg

aggatttggaaattgattagttccattaatattaggagctcctgatatagcattccctcg

aataaataatataagtttttgattactgcctccttcattaactcttctattagttagaag

tatagttgaaaatggagctggaactggatgaactgtatacccaccattatcagcagctat

tgctcatggaggaggatcagtagatttagcaattttttctttacatttagctggaatttc

atcaattttaggagctgtaaattttattacaacagtaattaatatacgatcaacaggaat

tacatttgatcgaataccattatttgtttgagctgttgttattactgctattcttctttt

attatctctacctgttttagctggagctattactatattattaacagatcgaaatttaaa

tacttctttctttgatcctgctggaggaggagacccaattttataccaacacttattc

>CNGRK502-13|Chrysops aestuans|COI-5P|KR468359

aactctctattttattttcggagcttgagctggaataattggaacatcattaagtatttt

aattcgtgctgaactaggtcatccaggagccttaattggtgacgatcaaatttataatgt

aattgtaactgctcatgcatttgttataattttctttatagtaatacctattataattgg

aggatttggaaattgattagttccattaatattaggagctcctgatatagcattccctcg

aataaataatataagtttttgattactacctccttcattaactcttctattagttagaag

tatagttgaaaatggagctggaactggatgaactgtatacccaccattatcagcagctat

tgctcatggaggaggatcagtagatttagcaattttttctttacatttagctggaatctc

atcaattttaggagctgtaaattttattacaacagtaattaatatacgatcaacaggaat

tacatttgatcgaatgccattatttgtttgagctgttgttattactgctattcttctttt

attatccctacctgttttagctggagctattactatattattaacagatcgaaatttaaa

tacttctttctttgatcctgctggaggaggagacccaattttataccaacacttattc

>ACT014-06|Chrysops montanus morph1|COI-5P|KM285519

----------------------------------------ggaacatcattaagtatttt

aattcgtgctgaactaggtcatccaggagcattaattggtgatgatcaaatttataatgt

aattgtaactgctcatgcatttgttataattttctttatagtaatacctattataattgg

aggatttggaaattgattagttccattaatattaggagctcctgatatagcatttcctcg

aataaataatataagtttttgattacttcctccttcattaactctcttattagttagaag

tatagttgaaaacggagctggaactggatgaactgtatacccaccattatcagcagctat

tgctcatggaggaggatcagtagatttagcaattttttctttacatttagctggaatttc

atcaattttaggagctgtaaattttattacaacagtaattaatatacgatcaacaggaat

tacatttgatcgaataccattatttgtttgagctgttgttattactgctattcttctttt

attatctctacctgttttagctggagctattactatattattaacagatcgaaatttaaa

tacttctttctttgatcctgctggaggaggagatccaattttatatcaacatttattc

>MHTAB123-09|Chrysops montanus morph1|COI-5P|KM285517

----------------------------------------ggaacatcattaagtatttt

aattcgtgctgaactaggtcatccaggagcattaattggtgatgatcaaatttataatgt

aattgtaactgctcatgcatttgttataattttctttatagtaatacctattataattgg

aggatttggaaattgattggttccattaatattaggagctcctgatatagcatttcctcg

aataaataatataagtttttgattacttcctccttcattaactctcttattagttagaag

tatagttgaaaacggagctggaactggatgaactgtatacccaccattatcagcagctat

tgctcatggaggaggatcagtagatttagcaattttttctttacatttagctggaatttc

atcaattttaggagctgtaaattttattacaacagtaattaatatacgatcaacaggaat

tacatttgatcgaataccattatttgtttgagctgttgttattactgctattcttctttt

attatctctacctgttttagctggagctattactatattattaacagatcgaaatttaaa

tacttctttctttgatcctgctggaggaggagatccaattttatatcaacatttattc

>ACT007-06|Chrysops montanus morph1|COI-5P|KM285513

----------------------------------------ggaacatcattaagtatttt

aattcgtgctgaactaggtcatccaggagcattaattggtgatgatcaaatttataatgt

aattgtaactgctcatgcatttgttataattttctttatagtaatacctattataattgg

aggatttggaaattgattagttccattaatattaggagctcctgatatagcatttcctcg

aataaataatataagtttttgattacttcctccttcattaactctcttattagttagaag

tatagttgaaaacggagctggaactggatgaactgtatacccaccattatcagcagctat

tgctcatggaggaggatcagtagatttagcaattttttctttacatttagctggaatttc

atcaattttaggagctgtaaattttattacaacagtaattaatatacgatcaacaggaat

tacatttgatcgaataccattatttgtttgagctgttgttattactgctattcttctttt

attatctctacctgttttagctggagctattactatattattaacagatcgaaatttaaa

tacttctttctttgatcctgccggaggaggagatccaattttatatcaacatttatct

>ACT040-07|Chrysops montanus morph1|COI-5P|KM285518

----------------------------------------ggaacatcattaagtatttt

aattcgtgctgaactaggtcatccaggagcattaattggtgatgatcaaatttataatgt

aattgtaactgctcatgcatttgttataattttctttatagtaatacctattataattgg

aggatttggaaattgattagttccattaatattaggagctcctgatatagcatttcctcg

aataaataatataagtttttgattacttcctccttcattaactctcttattagttagaag

tatagttgaaaacggagctggaactggatgaactgtatacccaccattatcagcagctat

tgctcatggaggaggatcagtagatttagcaattttttctttacatttagctggaatttc

atcaattttaggagctgtaaattttattacaacagtaattaatatacgatcaacaggaat

tacatttgatcgaataccattatttgtttgagctgttgttattactgctattcttctttt

attatctctacctgttttagctggagctattactataatattaacagatcgaaatttaaa

tacttctttctttgatcctgctggaggaggagatccaattttatatcaacatttattt

>ELPCG5529-17|Chrysops|COI-5P

aactctctattttattttcggggcttgagctggaataattggaacatcattaagtatttt

aattcgtgctgaactaggtcatccaggagcattaattggtgatgatcaaatttataatgt

aattgtaactgctcatgcatttgttataattttctttatagtaatacctattataattgg

aggatttggaaattgattagttccattaatattaggagctcctgatatagcatttcctcg

aataaataatataagtttttgattacttcctccttcattaactctcttattagttagaag

tatagttgaaaacggagctggaactggatgaactgtatacccaccattatcagcagctat

tgctcatggaggaggatcagtagatttagcaattttttctttacatttagctggaatttc

atcaattttaggagctgtaaattttattacaacagtaattaatatacgatcaacaggaat

tacatttgatcgaataccattatttgtttgagctgttgttattactgctattcttctttt

attatctctacctgttttagctggagctattactatattattaacagatcgaaatttaaa

tacttctttctttgatcctgctggaggaggagatccaattttatatcaacatttattc

>OPPEO6178-17|Chrysops|COI-5P

aactctctattttattttcggagcttgagctggaataattggaacatcattaagtatttt

aattcgtgctgaactaggtcatccaggagcattaattggtgatgatcaaatttataatgt

aattgtaactgctcatgcatttgttataattttctttatagtaatacctattataattgg

aggatttggaaattgattagttccattaatattaggagctcctgatatagcatttcctcg

aataaataatataagtttttgattacttcctccttcattaactctcttattagttagaag

tatagttgaaaacggagctggaactggatgaactgtatacccaccattatcagcagctat

tgctcatggaggaggatcagtagatttagcaattttttctttacatttagctggaatttc

atcaattttaggagctgtaaattttattacaacagtaattaatatacgatcaacaggaat

tacatttgatcgaataccattatttgtttgagctgttgttattactgctattcttctttt

attatctctacctgttttagctggagctattactatattattaacagatcgaaatttaaa

tacttctttctttgatcctgctggaggaggagatccaattttatatcaacatttattc

>CNTIF2597-15|Chrysops aestuans|COI-5P|MF837928

-actctctattttattttcggagcttgagctggaataattggaacatcattaagtatttt

aattcgtgctgaactaggtcatccaggagccttaattggtgacgatcaaatttataatgt

aattgtaactgctcatgcatttgttataattttctttatagtaatacctattataattgg

aggatttggaaattgattagttccattaatattaggagctcctgatatagcattccctcg

aataaataatataagtttttgattactacctccttcattaactcttctattagttagaag

tatagttgaaaatggagctggaactggatgaactgtatacccaccattatcagcagctat

tgctcatggaggaggatcagtagatttagcaattttttctttacatttagctggaatttc

atcaattttaggagctgtaaattttattacaacagtaattaatatacgatcaacaggaat

tacatttgatcgaataccattatttgtttgagctgttgttattactgctattcttctttt

attatccctacctgttttagctggagctattactatattattaaca--------------

----------------------------------------------------------

>CNGSE208-15|Chrysops aestuans|COI-5P|MF835350

-------------attttcggagcttgagctggaataattggaacatcattaagtatttt

aattcgtgctgaactaggtcatccaggagccttaattggtgacgatcaaatttataatgt

aattgtaactgctcatgcatttgttataattttctttatagtaatacctattataattgg

aggatttggaaattgattagttccattaatattaggagctcctgatatagcattccctcg

aataaataatataagtttttgattactacctccttcattaactcttctattagttagaag

tatagttgaaaatggagctggaactggatgaactgtatacccaccattatcagcagctat

tgctcatggaggaggatcagtagatttagcaattttttctttacatttagctggaatctc

atcaattttaggagctgtaaattttattacaacagtaattaatatacgatcaacaggaat

tacatttgatcgaatgccattatttgtttgagctgttgttattactgctattcttctttt

attatccctacctgttttagctggagctattactata-----------------------

----------------------------------------------------------

>CNGSE207-15|Chrysops aestuans|COI-5P|MF833710

-------------attttcggagcttgagctggaataattggaacatcattaagtatttt

aattcgtgctgaactaggtcatccaggagccttaattggtgacgatcaaatttataatgt

aattgtaactgctcatgcatttgttataattttctttatagtaatacctattataattgg

aggatttggaaattgattagttccattaatattaggagctcctgatatagcattccctcg

aataaataatataagtttttgattactacctccttcattaactcttttattagttagaag

tatagttgaaaatggagctggaactggatgaactgtatacccaccattatcagcagctat

tgctcatggaggaggatcagtagatttagcaattttttctttacatttagctggaatttc

atcaattttaggagctgtaaattttattacaacagtaattaatatacgatcaacaggaat

tacatttgatcgaataccattatttgtttgagctgttgttattactgctattcttctttt

attatccctacctgttttagctggagctattactatattattaacagat-----------

----------------------------------------------------------

>MHTAB119-09|Chrysops montanus morph2|COI-5P|KM285522

----------------------------------------ggaacatcattaagtatttt

aattcgtgctgaactaggtcacccaggagccttaattggtgatgatcaaatttataatgt

aattgtaactgctcacgcatttgttataattttctttatagtaatacctattataattgg

aggatttggaaattgattagttccattaatattaggagctcctgatatagcattccctcg

aataaataatataagtttttgattacttcctccttcattaactcttctattagttagaag

tatagttgaaaacggagctggaactggatgaactgtatacccaccattatcagcagctat

tgctcatggtggaggatcagtagatttagcaatcttttctttacatttagctggaatttc

atcaattttaggagctgtaaattttattactacagtaattaatatacgatcaacaggaat

tacatttgatcgaataccattatttgtttgagcagttgttattactgctattcttctttt

attatccctgcctgttttagctggagctattactatattattaacagatcgaaacttaaa

tacttctttctttgacccagctggaggaggagatccaattttataccaacatttattt

>ACT026-07|Chrysops montanus morph2|COI-5P|KM285521

----------------------------------------ggaacatcattaagtatttt

aattcgtgctgaactaggtcacccaggagccttaattggtgatgatcaaatttataatgt

aattgtaactgctcacgcatttgttataattttctttatagtaatacctattataattgg

aggatttggaaattgattagttccattaatattaggagctcctgatatagcattccctcg

aataaataatataagtttttgattacttcctccttcattaactcttctattagttagaag

tatagttgaaaacggagctggaactggatgaactgtatacccaccattatcagcagctat

tgctcatggtggaggatcagtagatttagcaatcttttctttacatttagctggaatttc

atcaattttaggagctgtaaattttattactacagtaattaatatacgatcaacaggaat

tacatttgatcgaataccattatttgtttgagcagttgttattactgctattcttctttt

attatccctacctgttttagctggagctattactatattattaacagatcgaaacttaaa

tacttctttctttgatccagctggaggaggagatccaattttatatcaacatttattt

>OPPEO6132-17|Chrysops|COI-5P

aactctctattttatttttggagcttgagccggaataattggaacatcattaagtatttt

aattcgtgctgaactaggtcacccaggagccttaattggtgatgatcaaatttataatgt

aattgtaactgctcacgcatttgttataattttctttatagtaatacctattataattgg

aggatttggaaattgattagttccattaatattaggagctcctgatatagcattccctcg

aataaataatataagtttttgattacttcctccttcattaactcttctattagttagaag

tatagttgaaaacggagctggaactggatgaactgtatacccaccattatcagcagctat

tgcccatggtggaggatcagtagatttagcaatcttttctttacatttagctggaatttc

atcaattttaggagctgtaaattttattactacagtaattaatatacgatcaacaggaat

tacatttgatcgaataccattatttgtttgagcagttgttattactgctattcatctttt

attatccctacctgttttagctggagctattactatattattaacagatcgaaacttaaa

tacttctttctttgacccagctggaggaggagatccaattttataccaacatttattt

>ASDIP574-15|Chrysops striatus|COI-5P

aactctctattttattttcggagcttgagccggaataattggaacatcattaagtatttt

aattcgtgctgaactaggtcacccaggagccttaattggtgatgatcaaatttataatgt

aattgtaactgctcatgcatttgttataattttctttatagtaatacctattataattgg

aggatttggaaattgattagttccattaatattaggagctcctgatatagcattccctcg

aataaataatataagtttttgattacttcctccttcattaactcttctattagttagaag

tatagttgaaaacggagctggaactggatgaantgtatacccaccattatcagcagctat

tgcncatggtggaggatcagtagatttagcaatcttttctttacatttagctggaatttc

atcaattttaggagctgtaaattttattactacagtaattaatatacgatcaacaggaat

tacatttgatcgaataccattatttgtttgagccgttgttattactgctattcntctttt

attatccctacctgttttagctggagctattactatattattaacagatcgaaatttaaa

tacttctttctttgacccagctggaggaggagatccaattttataccaacatttattt

>ASDIP573-15|Chrysops niger|COI-5P

aactctctattttattttcggagcttgagccggaataattggaacatcgttaagtatttt

aattcgtgctgaactaggtcacccaggagccttaattggtgatgatcaaatttataatgt

aattgtaactgctcatgcatttgttataattttctttatagtaatacctattataattgg

aggatttggaaattgattagttccattaatattaggagctcctgatatagctttccctcg

aataaataatataagtttttgattacttcctccttcattaactcttttgttagttagaag

tatagttgaaaatggagctggaactggatgaactgtatatcccccattatcagcagctat

tgctcatggcggaggatcagtagatttagcaattttttctttacatttagctggtatttc

ttcaattttaggggctgtaaattttattacaacagtaattaatatacgatcaacaggaat

tacatttgatcgaatacctttatttgtttgagctgttgttattactgctattcttctctt

attatctttacctgttttagctggagctattactatattattaacagatcgaaacttaaa

tacttcattttttgatccagctggagggggtgatccaattctataccaacatttattt

>CNGBL039-14|Chrysops niger|COI-5P|KR386366

aactctctattttattttcggagcttgagccggaataattggaacatcgttaagtatttt

aattcgtgctgaactaggtcacccaggagccttaattggtgatgatcaaatttataatgt

aattgtaactgctcatgcatttgttataattttctttatagtaatacctattataattgg

aggatttggaaattgattagttccattaatattaggagctcctgatatagctttccctcg

aataaataatataagtttttgattacttcctccttcattaactcttttgttagttagaag

tatagttgaaaatggagctggaactggatgaactgtatatcccccattatcagcagctat

tgctcatggtggaggatcagtagatttagcaattttttctttacatttagctggtatttc

ttcaattttaggggctgtaaattttattacaacagtaattaatatacgatcaacaggaat

tacatttgatcgaatacctttatttgtttgagctgttgttattactgctattcttctctt

attatctttacctgttttagctggagctattactatattattaacagatcgaaacttaaa

tacttcattttttgatccagctggagggggtgatccaattctataccaacatttattt

>CNPKF2772-14|Chrysops niger|COI-5P|KR391225

aactctctattttattttcggagcttgagccggaataattggaacatcgttaagtatttt

aattcgtgctgaactaggtcacccaggagccttaattggtgatgatcaaatttataatgt

aattgtaactgctcatgcatttgttataattttctttatagtaatacctattataattgg

aggatttggaaattgattagttccattaatattaggagctcctgatatagcattccctcg

aataaataatataagtttttgattacttcctccttcattaactcttttattagttagaag

tatagttgaaaatggagctggaactggatgaactgtatatcccccattatcagcagctat

tgctcatggtggaggatcagtagatttagcaattttttctttacatttagctggtatttc

ttcaattttaggggctgtaaattttattactacagtaattaatatacgatcaacaggaat

tacatttgatcgaatacctttatttgtttgagctgttgttattactgctattcttctttt

attatctttacctgttttagctggggctattactatattattaacagatcgaaacttaaa

tacttcattttttgatccagctggagggggtgatccaattctataccaacatttattt

>MHTAB153-09|Chrysops calvus|COI-5P|KM285442

----------------------------------------ggaacatcattaagtatttt

aattcgtgctgaactaggtcacccaggagccttaattggtgatgatcaaatttataatgt

aattgtaactgctcatgcatttgttataattttctttatagtaatacctattataattgg

gggattcggaaattgattagtcccattaatattaggagcccctgatatagcattccctcg

aataaataatataagtttttgattacttcctccttcattaactcttttattagttagaag

tatagttgaaaatggagctggaactggatgaactgtatatcccccattatcagcagctat

tgctcatggaggaggatcagtagatttagcaattttttctttacacttagctggtatttc

ttcaattttaggagctgtaaattttattacaacagtaattaatatacgatcaacaggaat

tacatttgatcgaatacctttatttgtttgagctgttgttattactgctattcttctttt

attatctttacctgttttagctggagctattactatattattaacagatcgaaatttaaa

tacttcattttttgatccagctggaggaggtgatccaattttataccaacatttattt

>SSKJB1058-14|Chrysops calvus|COI-5P|MF833772

-------------attttcggggcttgagccggaataattggaacatcattaagtatttt

aattcgtgctgaactaggtcacccaggagccttaattggtgatgatcaaatttataatgt

aattgtaactgctcatgcatttgttataattttctttatagtaatacctattataattgg

gggattcggaaattgattagtcccattaatattaggagcccctgatatagcattccctcg

aataaataatataagtttttgattacttcctccttcattaactcttttattagttagaag

tatagttgaaaatggagctggaactggatgaactgtatatcccccattatcagcagctat

tgctcatggagggggatcagtagatttagcaattttttctttacacttagctggtatttc

ttcaattttaggagctgtaaattttattacaacagtaattaatatacgatcaacaggaat

tacatttgatcgaatacctttatttgtttgagctgttgttattactgctattcttctttt

attatctttacctgttttagctggagctattactata-----------------------

----------------------------------------------------------

>SSKJB1105-14|Chrysops calvus|COI-5P|MF830178

-------------attttcggggcttgagccggaataattggaacatcattaagtatttt

aattcgtgctgaactaggtcacccaggagccttaattggtgatgatcaaatttataatgt

aattgtaactgctcatgcatttgttataattttctttatagtaatacctattataattgg

gggattcggaaattgattagtcccattaatattaggagcccctgatatagcattccctcg

aataaataatataagtttttgattacttcctccttcattaactcttttattagttagaag

tatagttgaaaatggagctggaactggatgaactgtatatcccccattatcagcagctat

tgctcatggagggggatcagtagatttagcaattttttctttacacttagctggtatttc

ttcaattttaggagctgtaaattttattacaacagtaattaatatacgatcaacaggaat

tacatttgatcgaatacctttatttgtttgagctgttgttattactgctattcttctttt

attatctttacctgttttagct--------------------------------------

----------------------------------------------------------

>SSKJB1033-14|Chrysops calvus|COI-5P|MF834136

-actctctattttattttcggggcttgagccggaataattggaacatcattaagtatttt

aattcgtgctgaactaggtcacccaggagccttaattggtgatgatcaaatttataatgt

aattgtaactgctcatgcatttgttataattttctttatagtaatacctattataattgg

gggattcggaaattgattagtcccattaatattaggagcccctgatatagcattccctcg

aataaataatataagtttttgattacttcctccttcattaactcttttattagttagaag

tatagttgaaaatggagctggaactggatgaactgtatatcccccattatcagcagctat

tgctcatggagggggatcagtagatttagcaattttttctttacacttagctggtatttc

ttcaattttaggagctgtaaattttattacaacagtaattaatatacgatcaacaggaat

tacatttgatcgaatacctttatttgtttgagctgttgttattactgctattcttctttt

attatctttacctgttttagctggagctattactata-----------------------

----------------------------------------------------------

>SSKJC2190-15|Chrysops calvus|COI-5P|MF835173

-actctctattttattttcggggcttgagccggaataattggaacatcattaagtatttt

aattcgtgctgaactaggtcacccaggagccttaattggtgatgatcaaatttataangt

aattgtaactgctcatgcatttgttataattttctttatagtaatacctattataattgg

gggattcggaaattgattagtcccattaatattaggagcccctgatatagcattccctcg

aataaataatataagtttttgattacttcctccttcattaactcttttattagttagaag

tatagttgaaaatggagctggaactggatgaactgtatatcccccattatcagcagctat

tgctcatggagggggatcagtagatttagcaattttttctttacacttagctggtatttc

ttcaattttaggagctgtaaattttattacaacagtaattaatatacgatcaacaggaat

tacatttgatcgaatacctttatttgtttgagctgttgttattactgctattcttctttt

attatctttacctgttttagctggagctattactatattattaacagatcgaaatttaaa

tacttca---------------------------------------------------

>BBDIV1015-12|Chrysops flavidus|COI-5P

aactctctattttattttcggagcttgagccggaataattggaacatcattaagtatttt

aattcgtgctgaactaggtcacccaggagccttaattggtgatgatcaaatttataatgt

aattgtaactgctcatgcatttgttataattttctttatagtaatacctattataattgg

aggatttggaaattgattagttccattaatattaggagctcctgatatagcatttcctcg

aataaataatataagtttttgattacttcctccttcattaactcttttattagttagaag

tatagttgaaaatggagctggaactggatgaactgtatatccaccattatcagcagctat

tgctcatggtgggggatcagtagatttagcaattttttctttacatttagctggaatttc

ttcaattttaggagctgtaaattttattacaacagttattaatatacgatcaacaggaat

tacctttgatcgaatacctttatttgtttgagcagttgtcattactgctattcttctttt

attatctttacctgttttagctggagctattactatattattaacagatcgaaatttaaa

tacttctttctttgacccagctggaggaggagatccaattttataccaacatttattt

>BBDIT226-11|Chrysops flavidus|COI-5P

aactctctattttattttcggagcttgagccggaataattggaacatcattaagtatttt

aattcgtgctgaactaggtcacccaggagccttaattggtgatgatcaaatttataatgt

aattgtaactgctcatgcatttgttataattttctttatagtaatacctattataatcgg

aggatttggaaattgattagttccattaatattaggagctcctgatatagcatttcctcg

aataaataatataagtttttgattacttcctccttcattaactcttttattagttagaag

tatagttgaaaatggagctggaactggatgaactgtatatccaccattatcagcagctat

tgctcatggtgggggatcagtagatttagcaattttttctttacatttagctggaatttc

ttcaattttaggagctgtaaattttattacaacagttattaatatacgatcaacaggaat

tacctttgatcgaatacctttatttgtttgagcagttgtcattactgctattcttctttt

attatctttacctgttttagctggagctattactatattattaacagatcgaaatttaaa

tacttctttctttgacccagctggaggaggagatcccattttataccaacatttattt

>BBDIV1523-12|Chrysops flavidus|COI-5P

aactctctattttattttcggagcttgagccggaataattggaacatcattaagtatttt

aattcgtgctgaactaggtcacccaggagccttaattggtgatgatcaaatttataatgt

aattgtaactgctcatgcatttgttataattttctttatagtaatacctattataattgg

aggatttggaaattgattagttccattaatattaggagctcctgatatagcattccctcg

aataaataatataagtttttgattacttcctccttcattaactcttttattagttagaag

tatagttgaaaatggggctggaactggatgaactgtatatccaccattatcagcagctat

tgctcatggtggaggatcagtagatttagcaattttttctttacatttagctggaatttc

ttcaattttaggagctgtaaattttattacaacagttattaatatacgatcaacaggaat

cacctttgatcgaatacctttatttgtttgagcagttgtcattactgctattcttctttt

attatctttacctgttttagctggagctattactatattattaacagatcgaaatttaaa

tacttctttctttgacccagctggaggag-----------------------------

>BBDIV1627-12|Chrysops flavidus|COI-5P

aactctctattttattttcggagcttgagccggaataattggaacatcattaagtatttt

aattcgtgctgaactaggtcanccaggagccttaattggtgatgatcaaatttataatgt

aattgtaactgctcatgcatttgttataattttctttatagtaatacctattataattgg

aggatttggaaattgattagttccattaatattaggagctcctgatatagcatttcctcg

aataaataatataagtttttgattacttcctccttcactaactcttttattagttagaag

tatagttgaaaatggagctggaactggatgaactgtatanccaccattatcagcagctat

tgctcatggtggaggatcagtagatttagcaattttttctttacatttagctggaatttc

ttcaattttaggagctgtaaattttattacaacagttattaatatacgatcaacaggaat

tacatttgatcgaatacctttatttgtttgagcagttgtcattactgctattcttctttt

attatctttacctgttttagctggagctattactatattattaacagatcgaaatttaaa

tacttctttctttga-------------------------------------------

>SSPAB238-13|Chrysops sackeni|COI-5P|KM912302

aactctctattttattttcggagcttgagccggaataattggaacatcactaagtatttt

aattcgtgctgaactaggtcacccaggagccttaattggtgatgatcaaatttataatgt

aattgtaactgctcatgcttttgttataattttctttatagtaatacctattataattgg

gggattcggaaattgattagttccattaatattaggagctcctgatatagcatttcctcg

aataaataatataagtttttgattacttcccccttcattaactcttttattagttagaag

tatagttgaaaacggggctggaactggatgaactgtatacccaccattatcagcagctat

tgctcatggaggaggatcagtagatttagcaattttttctttacatttagctggaatttc

ttcaattttaggggctgtaaattttattacaacagtaattaatatacgatcaacaggaat

tacatttgatcgaatacctttatttgtttgagctgttgttattactgctattcttctttt

attatctttacctgttttagccggagctattactatattattaacagatcgaaatttaaa

tacttctttctttgatccagctggaggaggagatccaattctatatcaacatttattt

>OPPHE216-17|Chrysops sackeni|COI-5P

aactctctattttattttcggggcttgagtcggaataattggaacatcactaagtatttt

aattcgtgctgaactaggtcacccaggagccttaattggtgatgatcaaatttataatgt

aattgtaactgctcatgcttttgttataattttctttatagtaatacctattataattgg

aggatttggaaattgattagttccattaatattaggagctcctgatatagcatttcctcg

aataaataatataagtttttgattacttcccccttcattaactcttttattagttacaag

tatagttgaaaatggggctggaactggatgaactgtatacccaccattatcagcagctat

tgctcatggaggaggatcggtagatttagcaattttttctttacacttagctggaatttc

ttcaattttaggagctgtaaattttattacaacagtaattaatatacgatcaacaggaat

tacatttgatcgaatacctttatttgtttgagctgttgttattactgctattcttctttt

attatctttacctgttttagctggagctattactatattattaacagatcgaaatttaaa

tacttctttctttgatccagctggaggaggagatccaattttatatcaacatttattt

>CNTID3969-15|Chrysops sackeni|COI-5P|MF831673

-actctctattttattttcggggcttgagccggaataattggaacatcactaagtatttt

aattcgtgctgaactaggtcacccaggagccttaattggtgatgatcaaatttataatgt

aattgtaactgctcatgcttttgttataattttctttatagtaatacctattataattgg

aggattcggaaattgattagttccattaatattaggagctcctgatatagcatttcctcg

aataaataatataagtttttgattacttcccccttcattaactcttttattagttagaag

tatagttgaaaatggggctggaactggatgaactgtatatccaccattatcagcagctat

tgctcatggaggaggatcagtagatttagcaattttttctttacacttagctggaatttc

ttcaattttaggagctgtaaattttattacaacagtaattaatatacgatcaacaggaat

tacatttgatcgaatacctttatttgtttgagctgttgttattactgctattcttctttt

attatctttacctgttttagctggagctattactatattattaacagat-----------

----------------------------------------------------------

>GBMND48683-21|Chrysops flavidus|COI-5P|MW532704

------------------------------------------------------------

------------------------------------------------------------

-------------------tttgttataattttctttatagtaatacctattataattgg

aggatttggaaattgattagttccattaatattaggagctcctgatatagcatttcctcg

aataaataatataagtttttgattacttcctccttcattaactcttttattagttagaag

tatagttgaaaatggggctggaactggatgaactgtatacccaccattatcagcagctat

tgctcatggtggaggatcagtagatttagcaattttttctttacatttagctggaatttc

ttcaattttaggagctgtaaattttattacaacagttattaatatacgatcaacaggaat

tacctttgatcgaatacctttatttgtttgagcagttgtcattactgctattcttctttt

attatctttacctgttttagccg-------------------------------------

----------------------------------------------------------

>GBMND48677-21|Chrysops flavidus|COI-5P|MW532710

------------------------------------------------------------

------------------------------------------------------------

-------------------tttgttataattttctttatagtaatacctattataattgg

aggatttggaaattgattagttccattaatattaggagctcctgatatagcatttcctcg

aataaataatataagtttttgattacttcctccttcattaactcttttattagttagaag

tatagttgaaaatggagctggaactggatgaactgtatatccaccattatcagcagctat

tgctcatggtggaggatcagtagatttagcaattttttctttacatttagctggaatttc

ttcaattttaggagctgtaaattttattacaacagttattaatatacgatcaacaggaat

tacctttgatcgaatacctttatttgtttgagcagttatcattactgctattcttctttt

attatctttacctgttttagctg-------------------------------------

----------------------------------------------------------

>BBDCQ894-10|Chrysops brunneus|COI-5P|JN291661

aactctctattttattttcggagcttgagccggaataattggaacatctctaagtatttt

aattcgtgctgaactaggtcatccaggagctttaattggtgatgatcaaatttataatgt

aattgtaactgctcatgcatttgttataattttctttatagtaatacctattataattgg

aggatttggaaattgattagttccattaatattaggagctcctgatatagcatttcctcg

aataaataatataagtttttgattacttcctccttcattaactcttttattagttagaag

tatagttgaaaatggagctggaactggatgaacagtatacccaccattatcagcagcaat

tgctcatggtggaggatcagtagatttagcaattttttctttacatttagctggaatttc

ttcaattttaggagctgtaaattttattacaacagtaattaatatacgatcaacaggaat

tacatttgatcgaatacctttatttgtttgatctgttgttattactgctattcttctttt

attatctttacctgttttagctggagctattactatattattaacagatcgaaatttaaa

tacttctttttttgatccagctggaggaggagatccaattttataccaacatttattc

>BBDCQ903-10|Chrysops brunneus|COI-5P|JN291669

aactctctattttattttcggagcttgagccggaataattggaacatctctaagtatttt

aattcgtgctgaactaggtcatccaggagctttaattggtgatgatcaaatttataatgt

aattgtaactgctcatgcatttgttataattttctttatagtaatacctattataattgg

aggatttggaaattgattagttccattaatattaggagctcctgatatagcatttcctcg

aataaataatataagtttttgattacttcctccttcattaactcttttattagttagaag

tatagttgaaaatggagttggaactggatgaacagtatacccaccattatcagcagcaat

tgctcatggtggaggatcagtagatttagcaattttttctttacatttagctggaatttc

ttcaattttaggagctgtaaattttattacaacagtaattaatatacgatcaacaggaat

tacatttgatcgaatacctttatttgtttgatctgttgttattactgctattcttctttt

attatctttacctgttttagctggagctattactatattattaacagatcgaaatttaaa

tacttctttttttgatccagctggaggaggagatccaattttataccaacatttattc

>ASDIP914-15|Chrysops lateralis|COI-5P

aactctctattttatttttggagcttgagccggaataattggaacatcattaagtatttt

aattcgtgctgaactaggtcacccaggagccttaattggtgatgatcaaatttataatgt

aattgtaactgctcatgcatttgttataattttctttatagtaatacctattataattgg

aggatttggaaattgactagttccattaatattaggagctcctgatatagcattccctcg

aataaataatataagtttttgattacttcctccttctttaactcttctattagttagaag

tatagttgaaaatggagctggaactggatgaactgtatacccaccattatcagcagctat

tgcacatggaggaggatcagtagatttagcaattttttctttacacttagctggaatttc

ttcaattttaggagctgtaaattttattacaacagtaattaatatacgatcaacaggaat

cacattcgaccgaatacctttatttgtttgagctgttgttattactgctattcttctttt

attatctttacctgttttagctggagctattactatactattaacagatcgaaatttaaa

tacatcattctttgatccagctggaggaggtgatccaattttatatcaacatttattt

>BBDEC386-09|Chrysops|COI-5P|HM412074

-------------atttttggagcttgagccggaataattggaacatcattaagtatttt

aattcgtgctgaactaggtcacccaggagccttaattggtgatgatcaaatttataatgt

aattgtaactgctcatgcatttgttataattttctttatagtaatacctattataattgg

aggatttggaaattgactagttccattaatattaggagctcctgatatagcattccctcg

aataaataatataagtttttgattacttcctccttctttaactcttctattagttagaag

tatagttgaaaatggagctggaactggatgaactgtatacccaccattatcagcagctat

tgcacatggaggaggatcagtagatttagcaattttttctttacacttagctggaatttc

ttcaattttaggagctgtaaattttattacaacagtaattaatatacgatcaacaggaat

cacattcgaccgaatacctttatttgtttgagctgttgttattactgctattcttctttt

attatctttacctgttttagctggagctattactatactattaacagatcgaaatttaaa

tacatcattctttgatccagctggaggaggtgatccaattttataccaacatttattt

>CNKJN075-14|Chrysops|COI-5P|KR394747

-actctctattttatttttggagcttgagccggaataattggaacatcattaagtatttt

aattcgtgctgaactaggtcacccaggagccttaattggtgatgatcaaatttataatgt

aattgtaactgctcatgcatttgttataattttctttatagtaatacctattataattgg

aggatttggaaattgactagttccattaatattaggagctcctgatatagcattccctcg

aataaataatataagtttttgattacttcctccttctttaactcttctattagttagaag

tatagttgaaaatggagctggaactggatgaactgtatacccaccattatcagcagctat

tgcacatggaggaggatcagtagatttagcaattttttctttacacttagctggaatttc

ttcaattttaggagctgtaaattttattacaacagtaattaatatacgatcaacaggaat

cacattcgaccgaatacctttatttgtttgagctgttgttattactgctattcttctttt

attatctttacctgttttagctggagctattactatactattaaca--------------

----------------------------------------------------------

>CNTIF2427-15|Chrysops|COI-5P|MF833041

aactctctattttatttttggagcttgagccggaataattggaacatcattaagtatttt

aattcgtgctgaactaggtcacccaggagccttaattggagatgatcaaatttataatgt

aattgtaactgctcatgcatttgttataattttctttatagtaatacctattataattgg

aggatttggaaattgattagtaccattaatattaggagctcctgatatagcattccctcg

aataaataatataagtttttgattacttcctccttcattaactcttctattagtcagaag

tatagttgaaaatggagctggaactggatgaactgtatacccaccattatcagcagctat

tgctcatggaggaggatcagtagatttagcaattttttctttacatttagctggaatttc

ttcaattttaggtgctgtaaattttattacaacagtaattaatatacgatcaacaggaat

tacatttgatcgaatacctttatttgtttgagctgttgttattactgctattcttctctt

attatctctacctgttttagctggtgctattactatactattaacagatcgaaatttaaa

tacttcattttttgacccagccggaggaggtgacccaattttatatcaacatttattt

>CNNHB271-14|Chrysops furcatus|COI-5P|KR495513

-actctctattttatttttggagcttgagccggaataattggaacatcattaagtatttt

aattcgtgctgaactaggtcacccaggagccttaattggtgatgatcaaatttataatgt

aattgtaactgctcatgcatttgttataattttctttatagtaatacctattataattgg

aggatttggaaattgattagttccattaatattaggagcacctgatatagcatttcctcg

aataaataatataagtttttgattactccctccttcattaacacttttattagttagaag

tatagttgaaaatggagctggaactggatgaactgtatacccacccttatcagctgctat

tgctcatggagggggctcagtagatttagcaattttttctttacacttagctggaatttc

ttcaattttaggagctgtaaattttattacaacagtaattaatatacgatcaacaggaat

tacattcgatcgaatacctttatttgtttgagctgttgttattactgctattcttctttt

attatctttacctgttttagctggagctattaccatattattaacagat-----------

----------------------------------------------------------

>CNMIG2397-14|Chrysops furcatus|COI-5P|KR392566

-actctctattttatttttggagcttgagccggaataattggaacatcattaagtatttt

aattcgtgctgaactaggtcacccaggagccttaattggtgatgatcaaatttataatgt

aattgtaactgctcatgcatttgttataattttctttatagtaatacctattataattgg

aggatttggaaattgattagttccattaatattaggagcacctgatatagcatttcctcg

aataaataatataagtttttgattacttcctccttcattaacacttttattagttagaag

tatagttgaaaatggagctggaactggatgaactgtatacccacccttatcagctgctat

tgctcatggagggggctcagtagatttagcaattttttctttacacttagctggaatttc

ttcaattttaggagctgtaaattttattacaacagtaattaatatacgatcaacaggaat

tacattcgatcgaatacctttatttgtttgagctgttgttattactgctattcttctttt

attatctttacctgttttagctggagctattaccatattattaacagatcga--------

----------------------------------------------------------

>SSGBB1007-14|Chrysops furcatus|COI-5P|KR522784

----ctctattttatttttggagcttgagccggaataattggaacatcattaagtatttt

aattcgtgctgaactaggtcacccaggagccttaattggtgatgatcaaatttataatgt

aattgtaactgctcatgcatttgttataattttctttatagtaatacctattataattgg

aggatttggaaattgattagttccattaatattaggagcacctgatatagcatttcctcg

aataaataatataagtttttgattacttcctccttcattaacacttttattagttagaag

tatagttgaaaatggagctggaactggatgaactgtatacccacccttatcagctgctat

tgctcatggagggggctcagtagatttagcaattttttctttacacttagctggaatttc

ttcaattttaggagctgtaaattttattacaacagtaattaatatacgatcaacaggaat

tacattcgatcgaatacctttatttgtttgagctgttgttattactgctattcttctttt

attatctttacctgttttagctggagctattaccatattattaacagat-----------

----------------------------------------------------------

>CNNHC044-14|Chrysops furcatus|COI-5P|KR979492

----------tttatttttggagcttgagccggaataattggaacatcattaagtatttt

aattcgtgctgaactagntcacccaggagccttaattggtgatgatcaaatttataatgt

aattgtaactgctcatgcatttgttataattttctttatagtaatacctattataattgg

aggatttggaaattgattagttccattaatattaggagcacctgatatagcatttcctcg

aataaataatataagtttttgattacttcctccttcattaacacttttattagttagaag

tatagttgaaaatggagctggaactggatgaactgtatacccacccttatcagctgctat

tgctcatggagggggctcagtagatttagcaattttttctttacacttagctggaatttc

ttcaattttaggagctgtaaattttattacaacagtaattaatatacgatcaacaggaat

tacattcgatcgaatacctttatttgtttgagctgttgttattactgctatt--------

------------------------------------------------------------

----------------------------------------------------------

>TTDFW509-08|Chrysops furcatus|COI-5P|KM570666

aactctctattttatttttggagcttgagccggaataattggaacatcattaagtatttt

aattcgtgctgaactaggtcacccaggagccttaattggtgatgatcaaatttataatgt

aattgtaactgctcatgcatttgttataattttctttatagtaatacctattataattgg

aggatttggaaattgattagttccattaatattaggagcacctgatatagcatttcctcg

aataaataatataagtttttgattacttcctccttcattaacacttttattagttagaag

tatagttgaaaatggagctggaactggatgaactgtatacccacccttatcagctgctat

tgctcatggagggggctcagtagatttagcaattttttctttacacttagctggaatttc

ttcaattttaggagctgtaaattttattacaacagtaattaatatacgatcaacaggaat

tacattcgatcgaatacctttatttgtttgagctgttgttattactgctattcttctttt

attatctttacctgttttagctggagctattaccatattattaacagatcgaaatttaaa

tacttcattctttgatccagctggaggaggagacccaattttataccaacatttattc

>MHTAB009-07|Chrysops furcatus|COI-5P|KM285499

aactctctattttatttttggagcttgagccggaataattggaacatcattaagtatttt

aattcgtgctgaactaggtcacccaggagccttaattggtgatgatcaaatttataatgt

aattgtaactgctcatgcatttgttataattttctttatagtaatacctattataattgg

aggatttggaaattgattagttccattaatattaggagcacctgatatagcatttcctcg

aataaataatataagtttttgattacttcctccttcattaacacttttattagttagaag

tatagttgaaaatggagctggaactggatgaactgtatacccacccttatcagctgctat

tgctcatggagggggctcagtagatttagcaattttttctttacacttagctggaatttc

ttcaattttaggagctgtaaattttattacaacagtaattaatatacgatcaacaggaat

tacattcgatcgaatacctttatttgtttgagctgttgttattactgctattcttctttt

attatctttacctgttttagctggagctattaccatattattaacagatcgaaatttaaa

tacttcattctttgacccagctggaggaggagacccaattttataccaacatttatt-

>CNEID3161-12|Chrysops furcatus|COI-5P|KR439721

aactctctattttatttttggagcttgagccggaataattggaacatcattaagtatttt

aattcgtgctgaactaggtcacccaggagccttaattggtgatgatcaaatttataatgt

aattgtaactgctcatgcatttgttataattttctttatagtaatacctattataattgg

aggatttggaaattgattagttccattaatattaggagcacctgatatagcatttcctcg

aataaataatataagtttttgattactccctccttcattaacacttttattagttagaag

tatagttgaaaatggagctggaactggatgaactgtatacccacccttatcagctgctat

tgctcatggagggggctcagtagatttagcaattttttctttacacttagctggaatttc

ttcaattttaggagctgtaaattttattacaacagtaattaatatacgatcaacaggaat

tacattcgatcgaatacctttatttgtttgagctgttgttattactgctattcttctttt

attatctttacctgttttagctggagctattaccatattattaacagatcgaaatttaaa

tacttcattctttgacccagc-------------------------------------

>MHTAB005-07|Chrysops furcatus|COI-5P|KM285496

--------------------------------ggataattggaacatcattaagtatttt

aattcgtgctgaactaggtcacccaggagccttaattggtgatgatcaaatttataatgt

aattgtaactgctcatgcatttgttataattttctttatagtaatacctattataattgg

aggatttggaaattgattagttccattaatattaggagcacctgatatagcatttcctcg

aataaataatataagtttttgattacttcctccttcattaacacttttattagttagaag

tatagttgaaaatggagctggaactggatgaactgtatacccacccttatcagctgctat

tgctcatggagggggctcagtagatttagcaattttttctttacacttagctggaatttc

ttcaattttaggagctgtaaattttattacaacagtaattaatatacgatcaacaggaat

tacattcgatcgaatacctttatttgtttgagctgttgttattactgctattcttctttt

attatctttacctgttttagctggagctattaccatattattaacagatcgaaatttaaa

tacttcattctttgacccagctggaggaggagac------------------------

>FIDIP2948-12|Chrysops rufipes|COI-5P|Pending (#7799)

aactctctattttatttttggagcttgagccggaataattggaacatccttaagtatttt

aattcgtgctgaactaggtcacccaggagccttaattggtgacgatcaaatttataatgt

aattgtaactgctcatgcatttgttataattttctttatagtaatacctattataattgg

aggatttggaaattgattagttccattaatattaggagctcctgatatagcatttcctcg

aataaataatataagtttttgattacttcctccttcattaactcttctattagttagaag

tatagttgaaaacggagctggaactggatgaactgtttatcccccattatcagcaactat

tgctcatggaggaggatcagttgatttagcaattttttctttacatttagccggaatttc

ttcaattttaggagctgtaaattttattactacagtaattaatatacgatcaacaggaat

cacatttgatcgaatacctttatttgtttgagctgttgttattactgctattcttctttt

attatctttacctgttttagctggagctattacaatattattaacagatcgaaatttaaa

tacttcattttttgacccagctggaggaggagacccaattttataccaacatttattt

>FIDIP2949-12|Chrysops rufipes|COI-5P|Pending (#7799)

aactctctattttatttttggagcttgagccggaataattggaacatccttaagtatttt

aattcgtgctgaactaggtcacccaggagccttaattggtgacgatcaaatttataatgt

aattgtaactgctcatgcatttgttataattttctttatagtaatacctattataattgg

aggatttggaaattgattagttccattaatattaggagctcctgatatagcatttcctcg

aataaataatataagtttttgattacttcctccttcattaactcttctattagttagaag

tatagttgaaaacggagctggaactggatgaactgtttatcccccattatcagcaactat

tgctcatggaggaggatcagttgatttagcaattttttctttacatttagccggaatttc

ttcaattttaggagctgtaaattttattactacagtaattaatatacgatcaacaggaat

cacatttgatcgaatacctttatttgtttgagctgttgttattactgctattcttctttt

attatctttacctgttttagctg-------------------------------------

----------------------------------------------------------

>OPPEQ110-17|Chrysops aberrans|COI-5P

aactctctattttattttcggagcttgagccggaataattggaacatcattaagtatttt

aattcgtgctgaactaggtcatccgggagccctgattggagacgatcaaatttataatgt

aattgtaactgctcatgcatttgttataattttctttatagttatacctattataattgg

gggatttggaaattgattagtcccattaatattaggagcacctgatatagcattccctcg

aataaataatataagtttttgattacttcccccttcattaactcttctattagttagaag

tatagttgaaaatggggctggaactggatgaactgtatacccaccattatcagcagctat

tgcacatggaggaggatcagtagatttagcaattttttctttacacttagctgggatttc

ttcaattttaggagctgtaaattttattacaacagtaattaatatacgatcaacaggaat

tacatttgatcgaatacctttatttgtttgagctgttgttattactgctattcttctttt

attatctttacctgttttagctggagcaattactatactattaacagatcgaaacttaaa

tacttctttctttgaccctgctgggggaggagacccaattttataccaacatttattt

>OPPQG322-17|Chrysops aberrans|COI-5P

aactctctattttattttcggagcttgagccggaataattggaacatcattaagtatttt

aattcgtgctgaactaggtcacccgggagccctgattggagacgatcaaatttataatgt

aattgtaactgctcatgcatttgttataattttctttatagttatacccattataattgg

gggatttggaaattgattagttccattaatattaggagcacctgatatagcattccctcg

aataaataatataagtttttgattacttcccccttcattaactcttctattagttagaag

tatagttgaaaatggggctggaactggatgaactgtatacccaccattatcagcagctat

tgcacatggaggaggatcagtagatttagcaattttttctttacacttagccgggatttc

ttcaattttaggggctgtaaattttattacaacagtaattaatatacgatcaacaggaat

tacatttgatcgaatacctttatttgtttgagctgttgttattactgctattcttctttt

attatctttacctgttttagctggagcaattactatactattaacagatcgaaatttaaa

tacttctttctttgaccctgctggaggaggagacccaattttataccaacatttattt

>ASDMT1097-11|Chrysops aberrans|COI-5P|MG169527

aactctctattttattttcggagcttgagccggaataattggaacatcattaagtatttt

aattcgtgctgaactaggtcacccgggagctctgattggagacgatcaaatttataatgt

aattgtaactgctcatgcatttgttataattttctttatagttatacctattataattgg

gggatttggaaattgattagtcccattaatattaggagcacctgatatagcattccctcg

aataaataatataagtttttgattacttcccccttcattaactcttctattagttagaag

tatagttgaaaatggggctggaactggatgaactgtatacccaccattatcagcagctat

tgcacatggaggaggatcagtagatttagcaattttttctttacatttagccggaatttc

ttcaattttaggggctgtaaattttattacaacagtaattaatatacgatcaacaggaat

tacatttgatcgaatacctttatttgtttgagctgttgttattactgctattcttctttt

attatctttacctgttttagctggagcaattactatactattaacagatcgaaatttaaa

tacttctttctttgaccctgctggaggaggagatccaattttataccaacatttattt

>CNSLP030-13|Chrysops aberrans|COI-5P|KP043552

aactctctattttattttcggagcttgagccggaataattggaacatcattaagtatttt

aattcgtgctgaactaggtcacccgggagccctgattggagacgatcaaatttataatgt

aattgtaactgctcatgcatttgttataattttctttatagttatacctattataattgg

gggatttggaaattgattagttccattaatattaggagcacctgatatagcattccctcg

aataaataatataagtttttgattacttcccccttcattaactcttctattagttagaag

tatagttgaaaatggggctggaactggatgaactgtatacccaccattatcagcagctat

tgcacatggaggaggatcagtagatttagcaattttttctttacacttagccgggatttc

ttcaattttaggggctgtaaattttattacaacagtaattaatatacgatcaacaggaat

tacatttgatcgaatacctttatttgtttgagctgttgttattactgctattcttctttt

attatctttacctgttttagctggagcaattactatactattaacagat-----------

----------------------------------------------------------

>ASDMT916-11|Chrysops aberrans|COI-5P|MG164869

aactctctattttattttcggagcttgagccggaataattggaacatcattaagtatttt

aattcgtgctgaactaggtcacccgggagccctgattggagatgatcaaatttataatgt

aattgtaactgctcatgcatttgttataattttctttatagttatacctattataattgg

aggatttggaaattgattagtcccattaatattaggagcacctgatatagcattccctcg

aataaataatataagtttttgattactccccccttcattaactcttctattagttagaag

tatagttgaaaatggggctggaactggatgaactgtatacccaccattatcagcagctat

tgcacatgggggaggatcagtagatttagcaattttttctttacacttagctgggatttc

ctcaattttaggagctgtaaattttattacaacagtaattaatatacgatcaacaggaat

tacatttgatcgaatacctttatttgtttgagctgttgttattactgctattcttctttt

attatctttacctgttttagctggagcaattactatactattaacagatcgaaacttaaa

tacttctttctttgaccctgctggaggaggagacccaattctataccaacatttattt

>OPPQG327-17|Chrysops aberrans|COI-5P

aactctctattttattttcggagcttgagccggaataattggaacatcattaagtatttt

aattcgtgctgaactaggtcatccgggagccctgattggagacgatcaaatttataatgt

aattgtaactgctcatgcatttgttataattttctttatagttatacctattataattgg

gggatttggaaattgattagtcccattaatattaggagcacctgatatagcattccctcg

aataaataatataagtttttgattacttcccccttcattaactcttctattagttagaag

tatagttgaaaatggggctggaactggatgaactgtataccccccattatcagcagctat

tgcacatggaggaggatcagtagatttagcaattttttctttacacttagccgggatttc

ttcaattttaggggctgtaaattttattacaacagtaattaatatacgatcaacaggaat

tacatttgatcgaatgcctttatttgtttgagctgttgttattactgctattcttctttt

attatctttacctgttttagctggagcaattactatactattaacagatcgaaatttaaa

tacttcattctttgaccctgctggaggaggagatccaattttataccaacatttattt

>CNTIF2599-15|Chrysops aberrans|COI-5P|MF833576

----------tttattttcggagcttgagccggaataattggaacatcattaagtatttt

aattcgtgctgaactaggtcacccgggagccctgattggagacgatcaaatttataatgt

aattgtaactgctcatgcatttgttataattttctttatagttatacctattataattgg

gggatttggaaattgattagttccattaatattaggagcacctgatatagcattccctcg

aataaataatataagtttttgattacttcccccttcattaactcttctattagttagaag

tatagttgaaaatggggctggaactggatgaactgtatacccaccattatcagcagctat

tgcacatggaggaggatcagtagatttagcaattttttctttacacttagccgggatttc

ttcaattttaggggctgtaaattttattacaacagtaattaatatacgatcaacaggaat

tacatttgatcgaatacctttatttgtttgagctgttgttattactgctattcttctttt

attatctttacctgttttagctgga-----------------------------------

----------------------------------------------------------

>BBDEC417-09|Chrysops shermani|COI-5P|HM435753

aactctctattttattttcggagcttgagccggaataattggaacatcattaagaattct

aattcgtgctgaactaggtcacccaggagccttaattggtgatgatcaaatttataatgt

aattgtaactgctcatgcatttgttataattttctttatagttatacctattataattgg

tggatttggaaattgattagtcccattaatgctaggagcccctgatatagcattcccccg

aataaataatataagtttttgattactccccccttcattaactcttttattagttagaag

tatagttgaaaacggggctggaactggatgaactgtatacccaccattatcagcagctat

tgctcatggaggaggatcagtagatttagcaattttttctttacacttagctggaatttc

ttcaattttaggagccgtaaattttattactacagttattaatatacgatcaacaggaat

tacatttgaccgaatacctttattcgtttgagctgttgttattactgctattcttctttt

attatctttacctgttttagctggagctattactatattattaacagatcgaaatttaaa

tacttcattctttgacccagctggaggaggagatccaattttatatcaacatctattt

>DIPUS244-10|Chrysops fuliginosus|COI-5P

aactctctattttatttttggagcttgagccggaataattggaacatcattaagtatttt

aattcgtgctgaactaggtcacccaggagccttaattggtgatgatcaaatttataatgt

aattgtaactgctcatgcatttgttataattttctttatagtaatacctattataattgg

aggatttggtaattgattagttccattaatacttggagctcctgatatagcatttcctcg

aataaataatataagtttttgattacttcctccttcattaactcttttattagttagaag

tatagttgaaaatggggctggaactggatgaactgtatatccaccattatcagctgctat

tgctcatggaggaggatcagtagacttagcaattttttctttacatttagctggtatttc

ttcaattttaggggctgtaaattttattacaacagtaattaatatacgatcaacaggaat

tacttttgatcgaataccattatttgtatgagctgttgttattactgctattcttctttt

attatctttacccgttttagctggagctattactatattattaactgatcgaaatttaaa

tacttcattttttgacccagctggaggaggtgatccaattttatatcaacatttattt

>DIPUS247-10|Chrysops fuliginosus|COI-5P

aactctctattttatttttggagcttgagccgggataattggaacatcattaagtatttt

aattcgtgctgaactaggtcacccaggagccttaattggtgatgatcaaatttataatgt

aattgtaactgctcatgcatttgttataattttctttatagtaatacctattataattgg

aggatttggtaattgattagttccattaatacttggagctcctgatatagcatttcctcg

aataaataatataagtttttgattacttcctccttcattaactcttttattagttagaag

tatagttgaaaatggggctggaactggatgaactgtatatccaccattatcagctgctat

tgctcatggaggaggatcagtagacttagcaattttttctttacatttagctggtatttc

ttcaattttaggagctgtaaattttattacaacagtaattaatatacgatcaacaggaat

tacctttgatcgaataccattatttgtatgagctgttgttattactgctattcttctttt

attatctttacccgttttagctggagctattactatattattaactgatcgaaatttaaa

tacttcattctttgacccagctggaggaggtgatccaattttatatcaacatttattt

>DIPUS248-10|Chrysops fuliginosus|COI-5P

aactctctattttatttttggagcttgagccggaataattggaacatcattaagtatttt

aattcgtgctgaactaggtcacccaggagccttaattggtgatgatcaaatttataatgt

aattgtaactgctcatgcatttgttataattttctttatagtaatacctattataattgg

aggatttggtaattgattagttccattaatacttggagctcctgatatagcatttcctcg

aataaataatataagtttttgattacttcctccttcattaactcttttattagttagaag

tatagttgaaaatggagctggaactggatgaactgtatatccaccattatcagctgctat

tgctcatggaggaggatcagtagatttagcaattttttctttacatttagctggtatttc

ttcaattttaggggctgtaaattttattacaacagtaattaatatacgatcaacaggaat

tacctttgaccgaataccattatttgtatgagctgttgttattactgctattcttctttt

attatctttacccgttttagctggagctattactatattattaactgatcgaaatttaaa

tacttcattttttgacccagctggaggaggtgatccaattttatatcaacatttattt

>BBDCP609-10|Chrysops|COI-5P|JF868971

aactctctattttattttcggggcttgagccggaataattggaacatcattaagtattct

aattcgtgctgaactaggtcacccaggagccttaattggtgatgatcaaatttataatgt

aattgtaactgctcatgcatttgttataattttctttatagtaatacctattataattgg

aggatttggaaattgattagttccattaatattaggagctcctgatatagcatttcctcg

aataaataatataagtttttgattacttccaccttcattaacccttctattagttagaag

tatagtcgaaaatggagctggaactggttgaactgtatacccaccattatctgcagctat

tgcccatggaggaggatcagtagatttagcaattttttctcttcatttagctggaatttc

atcaattttaggagctgtaaattttattacaacagtaattaatatacgatcaacaggaat

tacatttgatcgaatacctttatttgtttgagctgttgttattactgctattcttctttt

attatctttacctgttttagctggagctattacaatattattaacagatcgaaatttaaa

tacctcattctttgacccagcagggggaggagatccaattttataccaacatttattt

>ASIND3638-12|Chrysops scalaratus|COI-5P

aactctctattttattttcggagcttgagctggaataattggaacatcattaagtatttt

aattcgtgctgaattaggtcatccaggagctttaattggtgatgatcaaatttataatgt

aattgtaactgctcatgcatttgttataattttctttatagttatacctattataattgg

aggatttggaaattgactagttccattaatattaggagctcctgatatagcatttcctcg

tataaacaatataagtttttgattacttcccccttcattaacacttttattagttagtag

tatagtt-----------------------------------------------------

------------------------------------------------------------

------------------------------------------------------------

------------------------------------------------------------

------------------------------------------------------------

----------------------------------------------------------

>ASIND3609-12|Chrysops alajuelensis|COI-5P

aactctctattttattttcggggcttgagctggaataattggaacatctttaagtatttt

aattcgtgctgaattaggtcacccaggagctttaattggtgatgatcaaatttataatgt

aattgtaactgctcatgcatttgttataattttctttatagttatacctattataattgg

aggatttggaaattgattagttccattaatactaggagctcctgatatagcatttcctcg

aataaataatataagtt-------------------------------------------

------------------------------------------------------------

------------------------------------------------------------

------------------------------------------------------------

------------------------------------------------------------

------------------------------------------------------------

----------------------------------------------------------

>ASIND3631-12|Chrysops nexosus|COI-5P

aactctctattttattttcggagcttgagccggaataattggaacatctctaagtatttt

aattcgagctgaactaggtcacccaggagccttaattggtgatgatcaaatttataatgt

aattgtaactgctcatgcttttgttataattttctttatagttatacctattataattgg

aggatttggaaattgattagttccattaatattaggagctcctgatatagcattccctcg

aataaataatatgagtttttgattactccctccttcattaacacttttattagttagtag

tatagtt-----------------------------------------------------

------------------------------------------------------------

------------------------------------------------------------

------------------------------------------------------------

------------------------------------------------------------

----------------------------------------------------------

>ASIND3618-12|Chrysops calogaster|COI-5P

aactctctattttattttcggggcttgagccggaataattggaacatcactaagtatttt

aattcgagctgaactaggtcacccaggagccttaattggtgatgatcaaatttataatgt

aattgtaactgctcatgcttttgttataattttctttatagttatacctattataattgg

aggatttggaaattgattagttccattaatattaggagcccctgatatagcatttcctcg

aataaataatataagtttttgattacttcctccttcattaacacttttattagttagtag

tatagttgaaaatggagctggaactggatgaactgtatacccaccattatcagcagctat

cgcccatggaggaggttcagttgatttagcaattttttctttacatttagctggaatttc

ctcaattcttggagctgtaaattttattacaacagtaattaatatacgatcaacaggaat

tacatttgatcgaataccttnattngtttgagctgttgttattacagctattntactttt

attatctttacctgttttagctggagctattacaatactattaacngatcgaaatttaaa

tacttcattttttgacccagctggaggaggagatcctattttataccaacacttattt

>ASIND3619-12|Chrysops calogaster|COI-5P

aactctctattttattttcggggcttgagccggaataattggaacatcactaagtatttt

aattcgagctgaactaggtcacccaggagccttaattggtgatgatcaaatttataatgt

aattgtaactgctcatgcttttgttataattttctttatagttatacctattataattgg

aggatttggaaattgattagttccattaatattaggagcccctgatatagcatttcctcg

aataaataatataagtttttgattactccctccttcattaacacttttattagttagtag

tatagttgaaaatggagctggaactggatgaactgtatacccgccattatcagcagctat

cgcccatggaggaggttcagttgatttagcaattttttctttacatttagctggaatttc

ctcaattcttggggctgtaaactttattacaacagtaattaatatacgatcaacaggaat

tacatttgatcgaatacctttattcgtttgagctgttgttattacagctattttactttt

attatctttacctgttttagctggagctattacaatactattaacagatcgaaatttaaa

tacctcattttttgacccagctggaggaggagatcctattttataccaacacttattt

>ASIND3611-12|Chrysops auroguttatus|COI-5P

aactctctattttatttttggagcttgagccggaataattggaacatcattaagtatttt

aattcgagctgaattaggtcatccaggagctctaattggtgatgatcaaatttataatgt

aattgtaactgctcatgcatttgttataattttctttatagtaatacctattataattgg

gggatttggaaattgattagttcctttaatattaggagcccctgatatagcattcccacg

aataaataatataagtttttgattacttcctccttcattaacacttttattagtcagaag

tatagttgaaaatggagctggaactggatgaactgtatatcctcctttatcagctgctat

tgctcatggtggaggttcagttgatttagcaattttttctttacatttagctggaatttc

ttcaattttaggagctgtaaattttattacaacagtaattaatatacgatcaacaggaat

tacatttgatcgaataccattatttgtatgagctgttgttattacagcaattcttctttt

attatctttacctgttttagctggtgctattactatattattaacagatcgaaatttaaa

tacttcattctttgatccagctggaggaggagatccaattttataccaacatttattt

>ASIND4240-12|Chrysops costaricensis|COI-5P

aactctctattttatttttggagcttgagccggaataattggaacatcattaagtatttt

aattcgagctgaattaggtcatccaggagctctaattggtgatgatcaaatttataatgt

aattgtaactgctcatgcatttgttataattttctttatagtaatacctattataattgg

gggatttggaaattgattagttcctttaatattaggagcccctgatatagcattcccacg

aataaataatataagtttttgattacttcctccttcattaacacttttattagtcagaag

tatagttgaaaatggagctggaactggatgaactgtataccctcctttatcagctgctat

tgctcatggtggaggttcagttgatttagcaattttttctttacatttagctggaatttc

ttcaattttaggagctgtaaattttattacaacagtaattaatatacgatcaacaggaat

tacatttgatcgaataccattatttgtatgagctgttgttattacagcaattcttctttt

attatctttacctgttttagctggtgctattactatattattaacagatcgaaatttaaa

tacttcattctttgatccagctggaggaggagatccaattttataccaacatttattt

>ASIND3614-12|Chrysops auroguttatus|COI-5P

aactctctattttatttttggagcttgagccggaataattggaacatcattaagtatttt

aattcgagctgaattaggtcatccaggagctctaattggtgatgatcaaatttataatgt

aattgtaactgctcatgcatttgttataattttctttatagtaatacctattataattgg

gggatttggaaattgattagttcctttaatattaggagcccctgatatagcattcccacg

aataaataatataagtttttgattacttcctccttcattaacacttttattagtcagaag

tatagttgaaaatggagctggaactggatgaactgtatatcctcctttatcagctgctat

tgctcatggtggaggttcagttgatttagcaattttttctttacatttagctggaatttc

ttcaattttaggagctgtaaattttattacaacagtaattaatatacgatcaacaggaat

tacatttgatcgaataccattatttgtatgagctgttgttattacagcaattcttctttt

attatctttacctgttttagctggtgctattactatattattaacagatcgaaatttaaa

tacttcattctttgatccagctggaggaggagatccaattttatatcaacatttattt

>ASIND3613-12|Chrysops auroguttatus|COI-5P

aactctctattttatttttggagcttgagccggaataattggaacatcattaagtatttt

aattcgagctgaattaggtcatccaggagctctaattggtgatgatcaaatttataatgt

aattgtaactgctcatgcatttgttataattttctttatagtaatacctattataattgg

gggatttggaaattgattagttcctttaatattaggagcccctgatatagcattcccacg

aataaataatataagtttttgattacttcctccttcattaacacttttattagtcagaag

tatagtt-----------------------------------------------------

------------------------------------------------------------

------------------------------------------------------------

------------------------------------------------------------

------------------------------------------------------------

----------------------------------------------------------

>ASIND3643-12|Chrysops soror|COI-5P

aactctctattttatttttggagcttgagccggaataattggaacatctttaagtatttt

aattcgagctgaactaggtcacccaggagctctaattggtgatgatcaaatttataacgt

aattgtaactgctcatgcatttgttataattttctttatagttatacctattataattgg

aggatttggaaattgattagttcctttaatattaggagctcctgatatagcatttcctcg

aataaataatataagtttttgattacttcccccttctttaacccttttattagttagaag

tatagtt-----------------------------------------------------

------------------------------------------------------------

------------------------------------------------------------

------------------------------------------------------------

------------------------------------------------------------

----------------------------------------------------------

>ASIND3627-12|Chrysops mexicanus|COI-5P

aactctctattttattttcggagcttgagccggaataattggaacatcattaagtatttt

aattcgagctgaactaggtcacccaggagctttaattggtgatgatcaaatttataatgt

aattgtaactgctcatgcatttgttataattttctttatagttatacctattataattgg

aggatttggaaattgattagtacctttaatattaggggctcctgatatagcatttcctcg

aataaataatatgagtttttgattacttcccccttcattaacacttttattagttagtag

tatagttgaaaatggagctggaactggatgaacagtataccctccattatcagcagctat

tgctcatggaggaggttcagttgatttagcaattttttctttacatttagctggaatttc

ctcaattttaggagctgtaaattttattacaacagtaattaatatacgatctacaggaat

tacatttgatcgaatacctttatttgtttgagctgttgttattacagcaattcttctttt

attatcattacctgtattagctggagcaattactatactattaactgatcgaaatttaaa

tacttcattttttgatccagctggtggaggagatccaattttataccaacatttattt

>ASIND3630-12|Chrysops mexicanus|COI-5P

aactctctattttatttttggagcttgagccggaataattggaacatccttaagtatttt

aattcgagctgaactaggtcatccaggagctttaattggtgatgatcaaatttataatgt

aattgtaactgctcatgcatttgttataattttctttatagttatacctattataattgg

aggatttggaaattgacttgtaccattaatattaggagcccctgatatagcatttcctcg

aataaataatataagtttttgattacttcctccttcattaacacttttattagttagtag

tatagttgaaaatggagctggaactggatgaactgtatatcctccattatcagcaactat

tgctcatagaggaggttcagttgatctagcaattttttctttacatttagctggtatttc

ttcaattcttggggctgtaaactttattacaacagtaattaatatacgatctacaggaat

tacatttgatcgaatacctttatttgtatgagctgttgttattacagcaattcttctttt

attatcattacctgtattagctggagctattactatattattaactgatcgaaatttaaa

tacttcattttttgatccagcaggtggtggagatccaattttatatcaacatttattt

>CNTIC4014-15|Chrysops|COI-5P|MF836261

-------------attttcggagcttgagccggaataattggaacatcattaagtatttt

aattcgtgctgaactaggtcacccaggagctttaattggtgatgatcaaatttataatgt

aattgtaactgctcatgcatttgttataattttctttatagtaatacctattataattgg

gggatttggaaattgattagtcccattaatattaggagcccctgatatagcattccctcg

aataaataatataagtttctgattattacccccatcattaacccttctattagttagaag

tatagttgaaaatggagctggaactggatgaactgtatacccaccattatctgcagctat

tgcccatggaggaggatcagttgatttagcaattttttctttacatttagctggaatttc

ttctattttaggagctgtaaactttattacaacagtaattaatatacgatcaacaggaat

cacatttgatcgaatacctttatttgtttgagctgttgttattactgccattcttctttt

actatctttacctgttttagctggagctattactata-----------------------

----------------------------------------------------------

>CNRMD2011-12|Chrysops|COI-5P|KP042331

aactctctattttattttcggagcttgagccggaataattggaacatcattaagtatttt

aattcgtgctgaactaggtcacccaggagctttaattggtgatgatcaaatttataatgt

aattgtaactgctcatgcatttgttataattttctttatagtaatacctattataattgg

gggatttggaaattgattagtcccattaatattaggagcccctgatatagcattccctcg

aataaataatataagtttctgattattacccccatcattaacccttctattagttagaag

tatagttgaaaatggagctggaactggatgaactgtatacccaccattatctgcagctat

tgcccatggaggaggatcagttgatttagcaattttttctttacatttagctggaatttc

ttctattttaggagctgtaaactttattacaacagtaattaatatacgatcaacaggaat

cacatttgatcgaatacctttatttgtttgagctgttgttattactgccattcttctttt

actatctttacctgttttagctggagctattactatattattaacagatcgaaatttaaa

tacatccttctttgatcctgctggaggagg----------------------------

>OPPEC696-17|Chrysops|COI-5P

aactctctattttattttcggagcttgagccggaataattggaacatcattaagtatttt

aattcgtgctgaactaggtcacccaggagccttaattggtgatgatcaaatttataatgt

aattgtaactgctcatgcatttgttataattttctttatagtaatacctattataattgg

aggatttggaaattgattagtcccattaatattaggagcccctgatatagcattccctcg

aataaataatataagtttctgattattacccccatcattaacccttctattagttagaag

tatagttgaaaatggagctggaactggatgaactgtatacccaccattatctgcagctat

tgcccatggaggaggatcagttgatttagcaattttttctttacatttagccggaatttc

ttctattttaggagctgtaaactttattacaacagtaattaatatacgatcaacaggaat

cacatttgatcgaatacctttatttgtttgagctgttgttattactgccattcttctttt

actatctttacctgttttagctggagctattactatattattaacagatcgaaatttaaa

tacatccttctttgatcctgctggaggaggagatccaattttatatcaacatttattt

>JSDIQ799-10|Chrysops indus|COI-5P|JN302489

aactctctattttattttcggagcttgagccggaataattggaacatcattaagtatttt

aattcgtgctgaactaggtcacccaggagccttaattggtgatgatcaaatttataatgt

aattgtaactgctcatgcatttgttataattttctttatagtaatacctattataattgg

aggatttggaaattgattagtcccattaatattaggagcccctgatatagcattccctcg

aataaataatataagtttctgattattacccccatcattaacccttctattagttagaag

tatagttgaaaatggagctggaactggatgaactgtatacccaccattatctgcagctat

tgcccatggaggaggatcagttgatttagcaattttttctttacatttagctggaatttc

ttctattttaggagctgtaaactttattacaacagtaattaatatacgatcaacaggaat

cacatttgatcgaatacctttatttgtttgagctgttgttattactgccattcttctttt

actatctttacctgttttagctggagctattactatattattaacagatcgaaatttaaa

tacatccttctttgatcctgctggaggaggagatccaattttatatcaacatttattt

>TTDFW705-08|Chrysops indus|COI-5P|KM570604

aactctctattttattttcggagcttgagccggaataattggaacatcattaagtatttt

aattcgtgctgaactaggtcacccaggagccttaattggtgatgatcaaatttataatgt

aattgtaactgctcatgcatttgttataattttctttatagtaatacctattataattgg

aggatttggaaattgattagtcccattaatattaggagcccctgatatagcattccctcg

aataaataatataagtttctgattattacccccatcattaacccttctattagttagaag

tatagttgaaaatggagctggaactggatgaactgtatacccaccattatctgcagctat

tgctcatggaggaggatcagttgatttagcaattttttctttacatttagccggaatttc

ttctattttaggagctgtaaactttattacaacagtaattaatatacgatcaacaggaat

cacatttgatcgaatacctttatttgtttgagctgttgttattactgccattcttctttt

actatctttacctgttttagctggagctattactatattattaacagatcgaaatttaaa

tacatccttctttgaccctgctggaggaggagatccaattttatatcaacatttattt

>ACT080-07|Chrysops indus|COI-5P|KM285505

----------------------------------------ggaacatcattaagtatttt

aattcgtgctgaactaggtcacccaggagccttaattggtgatgatcaaatttataatgt

aattgtaactgctcatgcatttgttataattttctttatagtaatacctattataattgg

aggatttggaaattgattagtcccattaatattaggagcccctgatatagcattccctcg

aataaataatataagtttctgattattacccccatcattaacccttctattagttagaag

tatagttgaaaatggagctggaactggatgaactgtatacccaccattatctgcagctat

tgcccatggaggaggatcagttgatttagcaattttttctttacatttagccggaatttc

ttctattttaggagctgtaaactttattacaacagtaattaatatacgatcaacaggaat

cacatttgatcgaatacctttatttgtttgagctgttgttattactgccattcttctttt

actatctttacctgttttagctggagctattactatattattaacagatcgaaatttaaa

tacatccttctttgatcctgctggaggaggagatccaattttatatcaacatttattt

>ACT081-07|Chrysops indus|COI-5P|KM285504

----------------------------------------ggaacatcattaagtatttt

aattcgtgctgaactaggtcacccaggagccttaattggtgatgatcaaatttataatgt

aattgtaactgctcatgcatttgttataattttctttatagtaatacctatcataattgg

aggatttggaaattgattagtcccattaatattaggagcccctgatatagcattccctcg

aataaataatataagtttctgattattacccccatcattaacccttctattagttagaag

tatagttgaaaatggagctggaactggatgaactgtatacccaccattatctgcagctat

tgcccatggaggaggatcagttgatttagcaattttttctttacatttagctggaatttc

ttctattttaggagctgtaaactttattacaacagtaattaatatacgatcaacaggaat

cacatttgatcgaatacctttatttgtttgagctgttgttattactgccattcttctttt

actatctttacctgttttagctggagctattactatattattaacagatcgaaatttaaa

tacatccttctttgatcctgctggaggaggagatccaattttatatcaacatttattt

>JSDIQ839-10|Chrysops indus|COI-5P|KR655076

------------------------------------------------------------

--------------------------gagctttaattggtgatgatcaaatttataatgt

aattgtaactgctcatgcatttgttataattttctttatagtaatacctattataattgg

aggatttggaaattgattagtcccattaatattaggagcccctgatatagcattccctcg

aataaataatataagtttctgattattacccccatcattaacccttctattagttagaag

tatagttgaaaatggagctggaactggatgaactgtatacccaccattatctgcagctat

tgcccatggaggaggatcagttgatttagcaattttttctttacatttagccggaatttc

ttctattttaggagctgtaaactttattacaacagtaattaatatacgatcaacaggaat

cacatttgatcgaatacctttatttgtttgagctgttgttattactgccattcttctttt

actatctttacctgttttagctggagctattactatattattaacagatcgaaatttaaa

tacatccttctttgatcctgctggaggaggagacccaattttatatcaacatttattt

>SBGB012-03|Chrysops sp. SLB-2003|COI-5P|AY165640

-------------------ggagcttgagccggaataattggaacatcattaagtatttt

aattcgtgctgaactaggtcacccaggagctttaattggtgatgatcaaatttataatgt

aattgtaactgctcatgcatttgttataattttctttatagtaatacctattataattgg

gggatttggaaattgattagtcccattaatattaggagcccctgatatagcattccctcg

aataaataatataagtttctgattattacccccatcattaacccttctattagttagaag

tatagttgaaaatggagctggaactggatgaactgtatacccaccattatctgcagctat

tgcccatggaggaggatcagttgatttagcaattttttctttacatttagctggaatttc

ttctattttaggagctgtaaactttattacaacagtaattaatatacgatcaacaggaat

cacatttgatcgaatacctttatttgtttgagctgttgttattactgccattcttctttt

actatctttacctgttttagctggagctattactatattattaacagatcgaaatttaaa

tacatccttctttgatcctgctggaggaggagacccaatttta---------------

>ASIND4263-12|Chrysops sp.|COI-5P

aactctttactttattttcggagcttgagccggaataattggaacctcattaagtatttt

aattcgtgctgaattaggtcacccaggagctttaattggtgatgatcaaatttataatgt

aattgtaacagctcatgcatttgttataattttctttatagttatacctattataattgg

aggatttggaaattgactagttccattaatattaggagctcctgatatagcatttcctcg

aataaataatataagtttttgattactaccaccttcattaactcttttattagtcagaag

tatagttgaaaatggagctggaactggatgaactgtttatcccccattatcagctgctat

tgctcatggaggaggatcagttgatcttgcaattttttctttacatttagctggaatttc

atcaattttaggagctgtaaattttattacaacagtaattaatatacgatcaacaggaat

tacttttgatcgaataccattatttgtttgagctgtagttattacagctatccttttatt

attatcattacctgttttagctggagctattactatattattaactgatcgaaatttaaa

tacatctttttttgatccagctggaggaggagatccaattttatatcaacatttattt

>TTDFW266-08|Chrysops|COI-5P|KM569834

aactctctattttattttcggagcttgagctggaataattggaacatcgttaagtatttt

aattcgtgctgaactaggtcacccaggagctctaattggtgatgatcaaatttataatgt

aattgtaacagctcatgcatttgttataattttctttatagttatacctattataattgg

aggatttggaaattgattagtcccattaatattgggagcccctgatatagcattccctcg

aataaataatataagtttttgattacttcctccttcattaacccttttattagttagaag

tatagttgaaaatggagctggaactggatgaactgtataccccccattatcagctgctat

tgctcatggaggaggatcagttgatttagcaattttttccttacatttagctggaatttc

atcaattttaggagctgtaaattttattacaacagtaattaatatacgatcaacaggaat

tacatttgatcgaataccattatttgtttgagctgttgttattactgctattcttttatt

attatctttacctgttttagctggagctattaccatactattaactgatcgaaatttaaa

tacgtcattttttgacccggctggaggaggggatccaattttataccaacatttattt

>ASIND3622-12|Chrysops melaenus|COI-5P

aactctctattttatttttggagcttgagccggaataattggaacatcattaagtatttt

aattcgagctgaattaggtcatccaggagctttaattggagatgatcaaatttataatgt

aattgtaactgctcatgcatttgttataattttctttatagtaatacctattataattgg

aggatttggaaactgacttgtacctttaatattaggagcccctgatatagcatttccacg

aataaataatataagtttttgattacttcctccttctttaacccttttattagttagtag

aatagtt-----------------------------------------------------

------------------------------------------------------------

------------------------------------------------------------

------------------------------------------------------------

------------------------------------------------------------

----------------------------------------------------------

>ASDIP292-15|Chrysops striatus|COI-5P

aactctctattttattttcggagcttgagctggaataattggaacatcattaagtatttt

aattcgtgctgaactaggacaccctggagccttaattggtgatgatcaaatttataatgt

aattgtaactgctcatgcatttgttataattttctttatagtaatacctattataattgg

gggatttggaaattgattagttccactaatattaggtgcccctgatatagcattccctcg

aataaataatataagtttttgattacttcctccttcattaactcttctattagttagaag

tatagttgaaaatggggctggaactggatgaactgtatacccaccattatcagctgctat

tgctcatggtgggggatcagtagatttagcaattttttctttacacctagctggaatttc

ttcaattttaggggctgtaaattttattacaacagtaattaatatacgatcaactggaat

tacattcgatcgaatacctttatttgtttgagctgttgttattactgctattcttctctt

attatccttacctgttttagctggagccattactatacttttaacagatcgaaatttaaa

tacttccttctttgacccagctggaggaggagacccagtcttatatcaacatttattc

>ASDIP293-15|Chrysops striatus|COI-5P

----------------------------------ataattggaacatcattaagtatttt

aattcgtgctgaactaggacaccctggagccttaattggtgatgatcaaatttataatgt

aattgtaactgctcatgcatttgttataattttctttatagtaatacctattataattgg

gggatttggaaattgattagttccactaatattaggtgcccctgatatagcattccctcg

aataaataatataagtttttgattacttcctccttcattaactcttctattagttagaag

tatagttgaaaatggggctggaactggatgaactgtatacccaccattatcagctgctat

tgctcatggtgggggatcagtagatttagcaattttttctttacacctagctggaatttc

ttcaattttaggggctgtaaattttattacaacagtaattaatatacgatcaactggaat

tacattcgatcgaatacctttatttgtttgagctgttgttattactgctattcttctctt

attatccttacctgttttagctggagccattactatacttttaacagatcgaaatttaaa

tacttccttctttgacccagctggaggaggagacccagtcttatatcaacatttattc

>ACT011-06|Chrysops striatus|COI-5P|KM285526

----------------------------------------ggaacatcattaagtatttt

aattcgtgctgaactaggacaccctggagccttaattggtgatgatcaaatttataatgt

aattgtaactgctcatgcatttgttataattttctttatagtaatacctattataattgg

gggatttggaaattgattagttccactaatattaggtgcccctgatatagcattccctcg

aataaataatataagtttttgattacttcctccttcattaactcttctattagttagaag

tatagttgaaaatggggctggaactggatgaactgtatacccaccattatcagctgctat

tgctcatggtgggggatcagtagatttagcaattttttctttacacctagctggaatttc

ttcaattttaggggctgtaaattttattacaacagtaattaatatacgatcaactggaat

tacattcgatcgaatacctttatttgtttgagctgttgttattactgctattcttctctt

attatccttacctgttttagctggagccattactatacttttaacagatcgaaatttaaa

tacttccttctttgacccagctggaggaggagacccagtcttatatcaacatttattc

>ELPCG7681-17|Chrysops striatus|COI-5P

-actctctattttattttcggagcttgagctggaataattggaacatcattaagtatttt

aattcgtgctgaactaggacaccctggagccttaattggtgatgatcaaatttataatgt

aattgtaactgctcatgcatttgttataattttctttatagtaatacctattataattgg

gggatttggaaattgattagttccactaatattaggtgcccctgatatagcattccctcg

aataaataatataagtttttgattacttcctccttcattaactcttctattagttagaag

tatagttgaaaatggggctggaactggatgaactgtatacccaccattatcagctgctat

tgctcatggtgggggatcagtagatttagcaattttttctttacacctagctggaatttc

ttcaattttaggggctgtaaattttattacaacagtaattaatatacgatcaactggaat

tacattcgatcgaatacctttatttgtttgagctgttgttattactgctattcttctctt

attatccttacctgttttagctggagccattactatacttttaacagatcga--------

----------------------------------------------------------

>USDIP245-09|Chrysops|COI-5P|HM417163

aactctctattttattttcggagcttgagccggaataattggaacatcattaagtatttt

aattcgtgctgaactaggacacccaggagctttaattggggatgatcaaatttataatgt

aattgtaactgctcatgcatttgttataattttctttatagttataccaattataattgg

aggatttggaaattgattagttccattaatattaggagcccctgatatagcattccctcg

aataaataatataagtttttgattattacccccatcattaactcttcttttagttagaag

tatagttgaaaatggggctggaactggatgaactgtataccctcctttatcagcagctat

tgctcatggaggtggatcagtagatttagctattttctctttacacctagctggtatctc

ttcaattttaggtgctgtaaattttattacaactgtaattaatatacgatcaacaggaat

tacatttgatcgaatacctttatttgtttgagctgttgttattactgctattcttctttt

attatcattacctgttttagctggagctattactatattattaactgatcgaaatttaaa

tacttcattctttgatccagctggaggaggtgaccctattttatatcaacatttattt

>GMFRG1181-14|Chrysops|COI-5P

----------tttatttttggagcttgagccggaataattggaacatcattaagtatttt

aattcgtgctgaactaggacacccaggagctttaattggggatgatcaaatttataatgt

aattgtaactgctcatgcatttgttataattttctttatagtaatacctattataattgg

gggatttggaaattgattagttccattaatattaggagctcctgatatagcattccctcg

aataaataatataagtttttgattacttcccccttcattaactcttcttttagttagaag

tatagttgaaaatggggctggaactggatgaacagtatacccacctttatcagctgctat

tgctcatggaggaggatcagtagatctagcaattttttctttacacttagcaggtatttc

ttcaattctaggagctgtaaattttattacaactgtaattaatatacgatcaacaggaat

tacatttgatcgaatacctttatttgtttgagctgttgttattactgctattcttctttt

attatctctacctgttttagctggagctattactatactattaactgat-----------

----------------------------------------------------------

>GMFRG1180-14|Chrysops|COI-5P

-actctctattttatttttggagcttgagccggaataattggaacatcattaagtatttt

aattcgtgctgaactaggacacccaggagctttaattggggatgatcaaatttataatgt

aattgtaactgctcatgcatttgttataattttctttatagtaatacctattataattgg

aggatttggaaattgattagttccattaatattaggggctcctgatatagcattccctcg

aataaataatataagtttttgattacttcccccttcattaactcttcttttagttagaag

tatagttgaaaatggggctggaactggatgaacagtatacccacccttatcagctgctat

tgctcatggaggaggatcagtagatctagcgattttttctttacacttagcaggtatttc

ttcaattctaggagctgtaaattttattacaactgtaattaatatacgatcaacaggaat

tacatttgatcgaatacctttatttgtttgagctgttgttattactgctattcttctttt

attatctctacctgttttagctggagctattactatactattaactgat-----------

----------------------------------------------------------

>ASIND4261-12|Chrysops sp.|COI-5P

aacactttactttatttttggagcttgggccggaataattggaacatcattaagtatttt

aattcgagctgaactaggtcacccaggagctttaattggtgatgaccaaatttataatgt

aattgtaactgctcatgcttttgttataattttctttatagtaatacctattataattgg

aggatttggaaattgattagtacctttaatattaggagctcctgatatagcatttcctcg

aataaataatataagtttttgattactccctccttcattaacacttttattagttagtag

tatagttgaaaatggggctggaactggatgaacagtttaccctccattatctgctgctat

tgcacatggaggaggatcagttgatttagcaattttttctttacatttagctggaatttc

ttcaattttaggagctgtaaattttattacaactgttattaatatacgatctactggaat

tacatttgatcgtatacctctatttgtttgagctgttgttattacagcaatccttttatt

attatcattacctgttttagctggagcaattacaatattattaactgatcgaaatttaaa

tacttcattttttgatccagcaggaggaggtgatccaattttataccaacatcttttc

>ASIND3648-12|Chrysops varians|COI-5P

aacactttactttatttttggagcttgagccggaataattggaacatcattaagtatttt

aattcgagctgaactaggtcacccaggagctttaattggtgatgaccaaatttataatgt

aattgtaactgctcatgcttttgttataattttctttatagtaatacctattataattgg

aggatttggaaattgattagtacctttaatattaggagctcctgatatagcatttcctcg

aataaataatataagtttttgattactccctccttcattaacacttttattagttagtag

tatagttgaaaatggggctggaactggatgaacagtttaccctccattatctgctgctat

tgcacatggaggaggatcagttgatttagcaattttttctttacatttagctggaatttc

ttcaattttaggagctgtaaattttattacaactgttattaatatacgatctactggaat

tacatttgatcgtatacctctatttgtttgagctgttgttattacagcaatccttttatt

attatcattacctgttttagctggagcaattacaatattattaactgatcgaaatttaaa

tacttcattttttgatccagcaggaggaggtgatccaattttataccaacatcttttc

>FIDIP214-11|Chrysops caecutiens|COI-5P|Pending (#7798)

aacactttactttattttcggagcttgagccggaataattggaacatcattaagtatttt

aattcgtgctgaactaggtcacccaggagccttaattggtgatgatcaaatttataatgt

aattgtaactgctcatgcatttgttataattttctttatagtaatacctatcataattgg

agggtttggaaattgattagtgcctttaatattaggagcccctgatatagcattccctcg

aataaataatataagtttctgattactccctccttcattaacccttttattagttagaag

tatagttgaaaatggagcaggaactggttgaactgtataccctcctttatcagctgctat

tgcccatggaggaggatcagttgatttagcaattttttctttacatttagctgggatttc

atcaattttaggtgctgtaaattttattacaacagtaattaatatacgatcaacaggaat

tacatttgatcgaatacctttatttgtttgagctgtagttattacagcaattttactttt

attatctttaccagttttagcaggtgctattactatacttttaacagatcgaaatttaaa

tacatcatttttcgacccagctggaggaggagatcctattttataccaacacttattt

>GMGRD3604-13|Chrysops caecutiens|COI-5P

aacactttactttattttcggagcttgagccggaataattggaacatcattaagtatttt

aattcgtgctgaattaggtcatccaggagccttaattggtgatgatcaaatttataatgt

aattgtaactgctcatgcatttgttataattttctttatagtaatacctattataattgg

agggtttggaaattgattagttcctttaatattaggagcccctgatatagcattccctcg

aataaataatataagtttctgattactccctccttcattaacccttttattagttagaag

tatagttgaaaatggagcagggactggttgaactgtatacccccctttatcagctgctat

tgctcatggaggaggatcagttgatttagcaattttttctttacatttagctggaatttc

atcaattttaggtgctgtaaattttattacgacagtaattaatatacgatcaacaggaat

tacatttgatcgaatacctttatttgtttgagctgtagttattacagcaattttactttt

attatctttaccagttttagcaggtgcaattactatacttttaacagatcggaatctaaa

tacatcatttttcgacccagctggaggaggagaccctattttataccaacacttattt

>CROBB288_SK-4_Chrysops caecutiens

aacactttactttattttcggagcttgagccggaataattggaacatcattaagtatttt

aattcgtgctgaattaggtcatccaggagccttaattggtgatgatcaaatttataatgt

aattgtaactgctcatgcatttgttataattttctttatagtaatacctattataattgg

agggtttggaaattgattagttcctttaatattaggagcccctgatatagcattccctcg

aataaataatataagtttctgattactccctccttcattaacccttttattagttagaag

tatagttgaaaatggagcagggactggttgaactgtatacccccctttatcagctgctat

tgctcatggaggaggatcagttgatttagcaattttttctttacatttagctggaatttc

atcaattttaggtgctgtaaattttattacgacagtaattaatatacgatcaacaggaat

tacatttgatcgaatacctttatttgtttgagctgtagttattacagcaattttactttt

attatctttaccagttttagcaggtgcaattactatacttttaacagatcggaatctaaa

tacatcatttttcgacccagctggaggaggagaccctattttataccaacacttattt

>FIDIP1108-12|Chrysops caecutiens|COI-5P|Pending (#7798)

aacactttactttattttcggggcttgagccggaataattggaacatcattaagtatttt

aattcgtgctgaattaggtcatccaggagccttaattggtgatgatcaaatttataatgt

aattgtaactgctcatgcatttgttataattttctttatagtaatacctattataattgg

agggtttggaaattgattagttcctttaatattaggagcccctgatatagcattccctcg

aataaataatataagtttctgattactccctccttcattaacccttttattagttagaag

tatagttgaaaatggagcagggactggttgaactgtatacccccctttatcagctgctat

tgctcatggaggaggatcagttgatttagcaattttttctttacatttagctggaatttc

atcaattttaggtgctgtaaattttattacaacagtaattaatatacgatcaacaggaat

tacatttgatcgaatacctttatttgtttgagctgtagttattacagcaattttactttt

attatctttaccagttttagcgggtgcaattactatacttttaacagatcggaatctaaa

tacatcatttttcgacccagctggaggaggagaccctattttataccaacacttattt

>GBDP16768-15|Chrysops caecutiens|COI-5P|KM243493

aacactttactttattttcggggcttgagccggaataattggaacatcattaagtatttt

aattcgtgctgaattaggtcatccaggagccttaattggtgatgatcaaatttataatgt

aattgtaactgctcatgcatttgttataattttctttatagtaatacctattataattgg

agggtttggaaattgattagttcctttaatattaggagcycctgatatagcattccctcg

aataaataatataagtttctgattactccctccttcattaacccttttattagttagaag

tatagttgaaaatggagcagggactggttgaactgtatacccccctttatcagctgctat

tgctcatggaggaggatcagttgatttagcaattttttctttacatttagctggaatttc

atcaattttaggtgctgtaaattttattacgacagtaattaatatacgatcaacaggaat

tacatttgatcgaatacctttatttgtttgagctgtagttattacagcaattttactttt

attatctttaccagttttagcaggtgcaattactatacttttaacagatcggaatctaaa

tacatcatttttcgacccagctggaggaggagaccctattttatac------------

>IBIDP376-19|Chrysops caecutiens|COI-5P|MN868849

aacactttactttattttcggagcttgagccggaataattggaacatcattaagtatttt

aattcgtgctgaactaggtcatccaggagccttaattggtgatgatcaaatttataatgt

aattgtaactgctcatgcatttgttataattttctttatagtaatacctattataattgg

aggatttgggaattgattagtacctttaatattaggagcccctgatatagcatttcctcg

aataaataatataagtttctgattattacccccttcattaacccttttattagttagaag

tatagttgaaaatggagcagggactggttgaactgtatacccccctttatcagctgctat

tgctcatggaggaggatcagttgatttagcaattttttctttacatttagctggaatttc

atcaattttaggggctgtaaattttattacaacagtaattaatatacgatcaacaggaat

tacatttgatcgtatacctttatttgtttgagctgtggttattacagcaattttactttt

attatctttaccagttttagctggtgcaattactatacttttaacagatcgaaatttaaa

tacatcatttttcgatccagccggaggaggagaccctattttataccaacacttattt

>GBMIN62194-17|Chrysops caecutiens|COI-5P|MF458653

---------------------ggcttgagccggaataattggaacatcattaagtatttt

aattcgtgctgaactaggtcatccaggagccttaattggtgatgatcaaatttataatgt

aattgtaactgctcatgcatttgttataattttctttatagtaatacctattataattgg

aggatttggaaattgattagtacctttaatattaggagcccctgatatagcattccctcg

aataaataatataagtttctgattactccctccttcattaacccttttattagttagaag

tatagttgaaaatggagcagggactggttgaactgtatacccccctttatcagctgctat

cgctcatggaggaggatcagttgatttagcaattttttctttacatttagctggaatttc

atcaattttaggagctgtaaattttattacaacagtaattaatatacgatcaacaggaat

tacatttgatcgaatacctttatttgtttgagctgtagttattacagcaattttactttt

attatctttaccagttttagcaggtgctattactatacttttaacagatcggaatttaaa

tacatcatttttcgacccagccggaggaggagatcctattttataccaacactta---

>ASIND3610-12|Chrysops alleni|COI-5P

aactctatattttattttcggagcttgagccggaataattggaacatcattaagtatttt

aattcgagctgaacttggtcatccgggagccttaattggtgatgatcaaatttataatgt

aattgtaactgctcatgcatttgttataattttctttatagttatacctattataattgg

aggatttggaaattgattggttcctttaatacttggggcccctgatatagcattccctcg

aataaataatataagtttttgattacttcctccatcattaacccttttattagtaagaag

tatagtt-----------------------------------------------------

------------------------------------------------------------

------------------------------------------------------------

------------------------------------------------------------

------------------------------------------------------------

----------------------------------------------------------

>ACT057-07|Chrysops frigidus|COI-5P|KM285482

----------------------------------------ggaacatccttaagtatttt

aattcgtgctgaactaggtcacccaggagctttaattggagatgatcaaatttataatgt

aattgtaactgctcatgcatttgttataattttctttatagttatacctattataattgg

aggatttggaaattgattagtaccattaatattaggagcacctgatatagcttttcctcg

aataaataatataagtttttgattacttcctccttcattaacacttttattagttagtag

tatagttgaaaatggagctggaactggttgaactgtttaccccccattatctgctgctat

tgctcatggtggaggatcagtagatttagctattttttctttacacttagccggaatttc

atcaattttaggggctgtaaattttattacaacagtaattaatatacgatcaacaggaat

tacatttgatcgaatacctttatttgtttgagctgtagtaattacagctattcttcttct

attatctttacctgtcttagctggagcaattactatattattaacagatcgaaacttaaa

tacttcatttttcgacccagcaggaggaggagatcctattttataccaacacttattt

>JSDIQ820-10|Chrysops frigidus|COI-5P|KR658615

-----------------------------------taattggaacatccttaagtatttt

aattcgtgctgaactaggtcacccaggagctttaattggagatgatcaaatttataatgt

aattgtaactgctcatgcatttgttataattttctttatagttatacctattataattgg

aggatttggaaattgattagtaccattaatattaggagcacctgatatagcttttcctcg

aataaataatataagtttttgattacttcctccttcattaacacttttattagttagtag

tatagttgaaaatggagctggaactggttgaactgtttaccccccattatctgctgctat

tgctcatggtggaggatcagtagatttagctattttttctttacatttagccggaatttc

atcaattttaggagctgtaaattttattacaacagtaattaatatacgatcaacaggaat

tacatttgatcgaatacctttatttgtttgagctgtagtaattacagctattcttcttct

attatctttacctgtcttagctggagcaattactatattattaacagatcgaaacttaaa

tacttcatttttcgacccagcaggaggaggagatcctattttataccaacacttattt

>ACT048-07|Chrysops venus|COI-5P|KM285540

----------------------------------------ggaacatctttaagtatttt

aattcgtgctgaactaggtcacccaggagctttaattggagatgatcaaatttataatgt

aattgtaactgctcatgcatttgttataattttctttatagttatacctattataattgg

aggatttgggaattgattagtgccattaatattaggagcccctgatatagcttttcctcg

aataaataatataagtttttgattacttcctccttcattgacacttttattagttagtag

tatagttgaaaatggagctggaactggttgaactgtttaccccccattatcggctgctat

tgctcatggtggaggatcagttgatttagctattttttctttacatttagccggaatttc

atcaattttaggagctgtaaattttattacaacagtaattaatatacgatcaacaggaat

tacatttgatcgaatacctttatttgtttgagctgtagtaattacagctattcttcttct

attatctttacctgtcttagctggagcaattactatattattaacagatcgaaatttaaa

tacttcatttttcgacccagcaggaggaggagaccctattttataccaacacttattt

>ASDIP575-15|Chrysops venus|COI-5P

aacactttactttatttttggagcttgagctggaataattggaacatctttaagtatttt

aattcgtgctgaactaggtcacccaggagctttaattggagatgatcaaatttataatgt

aattgtaactgctcatgcatttgttataattttctttatagttatacctattataattgg

aggatttggaaattgattagtgccattaatattaggagcccctgatatagcttttcctcg

aataaataatataagtttttgattacttcctccttcattgacacttttattagttagtag

tatagttgaaaatggagctggaactggttgaactgtttaccccccattatctgctgctat

tgctcatggtggaggatcagttgatttagctattttttctttacatttagccggaatttc

atcaattttaggagctgtaaattttattacaacagtaattaatatacgatcaacaggaat

tacatttgatcgaatacctttatttgtttgagctgtagtaattacagctattcttcttct

attatctttacctgtcttagctggagcaattactatattattaacagatcgaaatttaaa

tacttcatttttcgacccagcaggaggaggagaccctattttataccaacatttattt

>CNPEP094-14|Chrysops|COI-5P|KR392545

aacactttactttattttcggagcttgagctggaataattggaacatccttaagtatttt

aattcgtgctgaactaggtcacccaggagctttaattggagatgatcaaatttataatgt

aattgtaactgctcatgcatttgttataattttctttatagttatacctattataattgg

aggatttggaaattgattagtgccattaatattaggagcccctgatatagcttttcctcg

aataaataatataagtttttgattacttcctccttcattgacacttttattagttagtag

tatagttgaaaatggggctggaactggttgaactgtttaccctccattatctgctgctat

tgctcatggtggaggatcagttgatttagctattttttctttacatttagccggaatttc

atcaattttaggagctgtaaattttattacaacagtaattaatatacgatcaacaggaat

tacatttgatcgaatacctttatttgtttgagctgtagtaattacagctattc-------

------------------------------------------------------------

----------------------------------------------------------

>CNPEF097-14|Chrysops|COI-5P|KR391531

aacactttactttattttcggagcttgagctggaataattggaacatccttaagtatttt

aattcgtgctgaactaggtcacccaggagctttaattggagatgatcaaatttataatgt

aattgtaactgctcatgcatttgttataattttctttatagttatacctattataattgg

aggatttggaaattgattagtaccattaatattaggagcacctgatatagcttttcctcg

aataaataatataagtttttgattacttcctccttcattaacacttttattagttagtag

tatagttgaaaatggagctggaactggttgaactgtttaccccccattatctgctgctat

tgctcatggtggaggatcagtagatttagctattttttctttacatttagccggaatttc

atcaattttaggagctgtaaattttattacaacagtaattaatatacgatcaacaggaat

tacatttgatcgaatacctttatttgtttgagctgtagtaattacagctattcttcttct

attatctttacctgtcttagctggagcaattactatattattaacagatcga--------

----------------------------------------------------------

>BBDED561-10|Chrysops frigidus|COI-5P|HM412889

aacactttactttattttcggagcttgagctggaataattggaacatccttaagtatttt

aattcgtgctgaactaggtcacccaggagctttaattggagatgatcaaatttataatgt

aattgtaactgctcatgcatttgttataattttctttatagttatacctattataattgg

aggatttggaaattgattagtaccactaatattaggagcacctgatatagcttttcctcg

aataaataatataagtttttgattacttcctccttcattaacacttttattagttagtag

tatagttgaaaatggagctggaactggttgaactgtttaccccccattatctgctgctat

tgctcatggtggaggatcagtagatttagctattttttctttacatttagccggaatttc

atcaattttaggagctgtaaattttattacaacagtaattaatatacgatcaacaggaat

tacatttgatcgaatacctttatttgtttgagctgtagtaattacagctattcttcttct

attatctttacctgtcttagctggagcaattactatattattaacagatcgaaacctaaa

tacttcatttttcgacccagcaggaggaggagatcctattttataccaacacttattt

>JWDCG837-10|Chrysops frigidus|COI-5P|JF876345

aacactttactttattttcggagcttgagctggaataattggaacatccttaagtatttt

aattcgtgctgaactaggtcacccaggagctttaattggagatgatcaaatttataatgt

aattgtaactgctcatgcatttgttataattttctttatagttatacctattataattgg

aggatttggaaattgattagtaccattaatattaggagcacctgatatagcttttcctcg

aataaataatataagtttttgattacttcctccttcattaacacttttattagttagtag

tatagttgaaaatggagctggaactggttgaactgtttaccccccattatctgctgctat

tgctcatggtggaggatcagtagatttagctattttttctttacatttagccggaatttc

atcaattttaggggctgtaaattttattacaacagtaattaatatacgatcaacaggaat

tacatttgatcgaatacctttatttgtttgagctgtagtaattacagctattcttcttct

attatctttacctgtcttagctggagcaattactatattattaacagatcgaaacttaaa

tacttcatttttcgacccagcaggaggaggagatcctattttataccaacacttattt

>JSDIQ852-11|Chrysops frigidus|COI-5P|KR665660

aacactttactttattttcggagcttgagctggaataattggaacatccttaagtatttt

aattcgtgctgaactaggtcacccaggagctttaattggagatgatcaaatttataatgt

aattgtaactgctcatgcatttgttataattttctttatagttatacctattataattgg

aggatttggaaattgactagtaccattaatattaggagcacctgatatagcttttcctcg

aataaataatataagtttttgattacttcctccttcattaacacttttattagttagtag

tatagttgaaaatggagctggaactggttgaactgtttaccccccattatctgctgctat

tgctcatggtggaggatcagtagatttagctattttttctttacatttagccggaatttc

atcaattttaggggctgtaaattttattacaacagtaattaatatacgatcaacaggaat

tacatttgatcgaatacctttatttgtttgagctgtagtaattacagctattcttcttct

attatctttacctgttttagctggagcaattactatattattaacagatcgaaacttaaa

tacttcatttttcgacccagcaggaggaggagatcctattttataccaacacttattt

>CNPAD1511-13|Chrysops|COI-5P|KR596496

aacactttactttattttcggagcttgagctggaataattggaacatccttaagtatttt

aattcgtgctgaactaggtcacccaggagctttaattggagatgatcaaatttataatgt

aattgtaactgctcatgcatttgttataattttctttatagttatacctattataattgg

aggatttggaaattgattagtaccattaatattaggagcacctgatatagcttttcctcg

aataaataatataagtttttgattgcttcctccttcattaacacttttattagttagtag

tatagttgaaaatggagctggaactggttgaactgtttaccccccattatctgctgctat

tgctcatggtggaggatcagtagatttagctattttttctttacatttagccggaatctc

atcaattttaggagctgtaaattttattacaacagtaattaatatacgatcaacaggaat

tacatttgatcgaatacctttatttgtttgagctgtagtaattacagctattcttcttct

attatctttacctgtcttagctggagcaattactatattattaacagatcgaaacttaaa

tacttcatttttcgacccagcaggagg-------------------------------

>CNPEP102-14|Chrysops|COI-5P|KR395254

aacactttactttattttcggagcttgagctggaataattggaacatccttaagtatttt

aattcgtgctgaactaggtcacccaggagctttaattggagatgatcaaatttataatgt

aattgtaactgctcatgcatttgttataattttctttatagttatacctattataattgg

aggatttggaaattgattagtaccattaatattaggagcacctgatatagcttttcctcg

aataaataatataagtttttgattacttcctccttcattaacacttttattagtcagtag

tatagttgaaaatggagctggaactggttgaactgtttaccccccattatctgctgctat

tgctcatggtggaggatcagtagatttagctattttttctttacatttagccggaatttc

atcaattttaggagctgtaaattttattacaacagtaattaatatacgatcaacaggaat

tacatttgatcgaatacctttatttgtttgagctgtagtaattacagctattcttcttct

attatctttacctgtcttagctggagcaattactatatt---------------------

----------------------------------------------------------

>SSPAA3146-13|Chrysops|COI-5P|KM908628

aacactttactttattttcggagcttgagctggaataattggaacatccttaagtatttt

aattcgtgctgaactaggtcacccaggagctttaattggagatgatcaaatttataatgt

aattgtaactgctcatgcatttgttataattttctttatagttatacctattataattgg

aggatttggaaattgattagtaccattaatattaggagcacctgatatagcttttcctcg

aataaataatataagtttttgattacttcctccttcattaacacttttattagttagtag

tatagttgaaaatggagctggaactggttgaactgtttaccccccattatctgctgctat

tgctcatggtggaggatcagtagatttagctattttttctttacatttagccggaatttc

atcaattttaggggctgtaaattttattacaacagtaattaatatacgatcaacaggaat

tacatttgatcgaatacctttatttgtttgagctgtagtaattacagctattcttcttct

attatctttacctgtcttagctggagcaattactatattattaacagatcgaaacttaaa

tacttcatttttcgacccagcaggaggagg----------------------------

>CNMIG871-14|Chrysops|COI-5P|KR389961

-acactttactttattttcggagcttgagctggaataattggaacatccttaagtatttt

aattcgtgctgaactaggtcacccaggagctttaattggagatgatcaaatttataatgt

aattgtaactgctcatgcatttgttataattttctttatagttatacctattataattgg

aggatttggaaattgattagtgccattaatattaggagcacctgatatagcttttcctcg

aataaataatataagtttttgattacttcctccttcattaacacttttattagttagtag

tatagttgaaaatggagctggaactggttgaactgtttaccccccattatctgctgctat

tgctcacggtggaggatcagtagatttagctattttttctttacatttagccggaatttc

atcaattttaggggctgtaaattttattacaacagtaattaatatacgatcaacaggaat

tacatttgatcgaatacctttatttgtttgagctgtagtaattacagctattcttcttct

attatctttacctgtcttagctgga-----------------------------------

----------------------------------------------------------

>SSGBA5205-14|Chrysops|COI-5P|MF829217

-acactttactttattttcggggcttgagctggaataattggaacatccttaagtatttt

aattcgtgctgaactaggtcacccaggagctttaattggagatgatcaaatttataatgt

aattgtaactgctcatgcatttgttataattttctttatagttatacctattataattgg

aggatttggaaattgattagtgccattaatattaggagcccctgatatagcttttcctcg

aataaataatataagtttttgattacttcctccttcattgacacttttattagttagtag

tatagttgaaaatggagctggaactggttgaactgtttaccctccattatctgctgctat

tgctcatggtggaggatcagtagatttagctattttttctttacatttagccggaatttc

atcaattttaggagctgtaaattttattacaacagtaattaatatacgatcaacaggaat

tacatttgatcgaatacctttatttgtttgagctgtagtaattacagctattcttcttct

attatctttacctgtcttagctggagcaattactata-----------------------

----------------------------------------------------------

>CNPEP1421-14|Chrysops|COI-5P|KR388754

----------------------gcttgagctggaataattggannntccttaagtatttt

aattcgtgctgaactaggtcacccaggagctttaattggagatgatcaaatttataatgt

aattgtaactgctcatgcatttgttataattttctttatagttatacctattataattgg

aggatttggaaattgattagtaccactaatattaggagcacctgatatagcttttcctcg

aataaataatataagtttttgattacttcctccttcattaacacttttattagttagtag

tatagttgaaaatggagctggaactggttgaactgtttaccccccattatctgctgctat

tgctcatggtggaggatcagtagatttagctattttttctttacatttagccggaatttc

atcaattttaggagctgtaaattttattacaacagtaattaatatacgatcaacaggaat

tacatttgatcgaatacctttatttgtttgagctgtagtaattacagctattcttcttct

attatctttacctg----------------------------------------------

----------------------------------------------------------

>CNFNE2857-14|Chrysops|COI-5P|KR394708

--------actttattttcggagcttgagctggaataattggaacatccttaagtatttt

aattcgtgctgaactaggtcacccaggagctttaattggagatgatcaaatttataatgt

aattgtaactgctcatgcatttgttataattttctttatagttatacctattataattgg

aggatttggaaattgattagtgccattaatattaggagcccctgatatagcttttcctcg

aataaataatataagtttttgattacttcctccttcattgacacttttattagttagtag

tatagttgaaaatggggctggaactggttgaactgtttaccctccattatctgctgctat

tgctcatggtggaggatcagttgatttagctattttttctttacatttagccggaatttc

atcaattttaggagctgtaaattttattacaacagtaattaatatacgatcaacaggaat

tacatttgatcgaatacctttatttgtttgagctgtagtaattacagctattcttcttct

attatctttacctgtcttagctggagcaatta----------------------------

----------------------------------------------------------

>FIDIP976-12|Chrysops viduatus|COI-5P|Pending (#7798)

aacactttactttattttcggagcttgagctggaataattggaacatctttaagtatttt

aattcgtgctgaactaggccatccaggagctttaattggagatgatcaaatttataatgt

aattgtaactgctcatgcatttgttataattttcttcatagttatacctattataattgg

aggatttggaaattgattagttccattaatattaggagcccctgatatagcctttcctcg

aataaataatataagtttttgattactacctccttcattaacacttttattagttagtag

tatagttgaaaatggggctggaactggttgaactgtatacccaccattatctgctgctat

tgctcatggaggtggatcagttgatttagcaattttctctttacatttagctggaatttc

atcaattttaggggctgtaaattttattacaacagtaattaatatacgatcaacaggaat

tacatttgaccgaatacctttatttgtttgagctgtagtaattacagcaattcttctttt

actatctttaccggttttagctggagctattactatactactaacagatcgaaatttaaa

tacatcattctttgacccagcaggaggaggtgatcctattttatatcaacacttattt

>IBIDP079-19|Chrysops viduatus|COI-5P|MN868882

aacactttactttattttcggagcttgagctggaataattggaacatctttaagtatttt

aattcgtgctgaactaggtcatccaggagctttaattggagatgatcaaatttataatgt

aattgtaactgctcatgcatttgttataattttcttcatagtcatacctattataattgg

aggatttgggaattgattagttccattaatattaggagcccctgatatagcctttcctcg

aataaataatataagtttttgattactgcctccttcattaacacttttattagttagtag

tatagttgaaaatggggctggaactggttgaactgtatacccaccattatctgctgctat

tgctcatggaggtggatcagttgatttagcaattttctctttacatttagctggaatttc

atcaattttaggagctgtaaattttattacaacagtaattaatatacggtcaacaggaat

cacatttgaccgaatacctttatttgtttgagctgtagtaattacagcaattcttctttt

attatctttaccagttttagctggagctattactatactactaacagatcgaaatttaaa

tacatcattctttgacccagcaggaggaggtgatcctattttatatcaacacttattt

>CROBB286_SK-2_Chrysops viduatus

aacactttactttattttcggagcttgagctggaataattggaacatctttaagtatttt

aattcgtgctgaactaggtcatccaggagctttaattggagatgatcaaatttataatgt

aattgtaactgctcatgcatttgttataattttcttcatagtcatacctattataattgg

aggatttggaaattgattagttccattaatattaggagcccctgatatagcctttcctcg

aataaataatataagtttttgattactgcctccttcattaacacttttattagttagtag

tatagttgaaaatggggctggaactggttgaactgtatacccaccattatctgctgctat

tgctcatggaggtggatcagttgatttagcaattttctctttacatttagctggaatttc

atcaattttaggagctgtaaattttattacaacagtaattaatatacgatcaacaggaat

cacatttgaccgaatacctttatttgtttgagctgtagtaattacagcaattcttctttt

attatctttaccagttttagctggagctattactatactactaacagatcgaaatttaaa

tacatcattctttgatccagcaggaggaggtgatcctattttatatcaacacttattt

>FIDIP1091-12|Chrysops relictus|COI-5P|Pending (#7798)

aacactttactttattttcggagcatgagccggaataattggaacttctttaagtatttt

aattcgtgctgaactaggacaccctggagctttaatcggagatgatcaaatttataatgt

aattgtaactgctcatgcatttgttataattttctttatagttatacctattataattgg

aggatttggaaattgattagtaccattaatattaggagctcctgatatagctttccctcg

aataaataatataagtttttgattacttcctccttcattaacacttttattagttagtag

tatagttgaaaatggggctgggactggttgaactgtttaccctccgctatctgctgctat

tgcacatggaggaggatcagttgatttagcaattttctctttacatttagctggaatttc

atcaattttaggagctgtaaattttattacaacagtaattaatatacgatcaacaggaat

tacatttgaccgaatacctttatttgtatgatctgtagttattacagcaattcttctttt

attatctttacctgttttagccggagctattactatattattaacagatcgaaacttaaa

tacatcattttttgaccctgcgggaggaggtgacccaattttataccaacatttattt

>FIDIP527-11|Chrysops relictus|COI-5P|Pending (#7799)

aacactttactttattttcggagcatgagccggaataattggaacttctttaagtatttt

aattcgtgctgaactaggacaccctggagctttaatcggagatgatcaaatttataatgt

aattgtaactgctcatgcatttgttataattttctttatagttatacctattataattgg

aggatttggaaattgattagtaccattaatattaggagctcctgatatagctttccctcg

aataaataatataagtttttgattacttcctccttcattaacacttttattagttagtag

tatagttgaaaatggggctgggactggttgaactgtttaccctccactatctgctgctat

tgcacatggaggaggatcagttgatttagcaattttctctttacatttagctggaatttc

gtcaattttaggggctgtaaattttattacaacagtaattaatatacgatcaacaggaat

tacatttgaccgaatacctttatttgtatgatctgtagttattacagcaattcttctttt

attatctttacctgttttagccggagctattactatattattaacagatcgaaacttaaa

tacatcattttttgaccctgcgggaggaggtgacccaattttataccaacatttattt

>CROBB285_SK-1_Chrysops relictus

aacactttactttattttcggagcatgagccggaataattggaacttctttaagtatttt

aattcgtgctgaactaggacatcctggagctttaatcggagatgatcaaatttataatgt

aattgtaactgctcatgcatttgttataattttctttatagttatacctattataattgg

aggatttggaaattgattagtaccattaatattaggagctcctgatatagctttccctcg

aataaataatataagtttttgattacttcctccttcattaacacttttattagttagtag

tatagttgaaaatggggctggaactggttgaactgtttaccctccactatctgctgctat

tgcacatggaggaggatcagttgatttagcaattttctctttacatttagctggaatttc

atcaattttaggggctgtaaattttattacaacagtaattaatatacgatcaacaggaat

tacatttgaccgaatacctttatttgtatgatctgtagttattacagcaattcttctttt

attatctttacctgttttagctggagctattactatattattaacagatcgaaacttaaa

tacatcattttttgaccctgcaggagggggtgacccaattttataccaacatttattt

>JWDCJ1399-11|Chrysops nigripes|COI-5P|JN302981

aacactttactttattttcggagcttgagctggaataattggaacatcattaagtatttt

aattcgtgctgaactaggtcatccaggagctttaattggtgatgatcaaatttataatgt

aattgtaactgctcatgcatttgttataattttctttatagtaatacctattataattgg

aggatttggaaattgattagttccattaatattaggagctcctgatatagctttccctcg

aataaataatataagtttttgattacttcctccttcattaacccttttattagttagtag

aatagttgaaaatggagctggaactggttgaactgtttaccctccattatcagctgctat

tgcacatggtggaggatcagttgatttagctattttttcattacatttagctggaatttc

atcaattttaggagctgtaaattttatcacaacagtaattaatatacgatcaacaggaat

tacatttgatcgaatacctttatttgtttgatctgtaataattacagcaattcttctttt

attatctttaccagttttagctggagctattactatattattaacagatcgaaatttaaa

tacatcattctttgatccagctggaggaggtgatccaattctatatcaacatttattt

>JWDCB043-10|Chrysops nigripes|COI-5P|HM861011

aacactttactttattttcggagcttgagctggaataattggaacatcattaagtatttt

aattcgtgctgaactaggtcatccaggagctttaattgatgatgatcaaatttataatgt

aattgtaactgctcatgcatttgttataattttctttatagtaatacctattataattgg

aggatttggaaattgattagttccattaatattaggagctcctgatatagctttccctcg

aataaataatataagtttttgattacttcctccttcattaacccttttattagttagtag

aatagttgaaaatggagctggaactggttgaactgtttaccctccattatcagctgctat

tgcacatggtggaggatcagttgatttagctattttttcattacatttagctggaatttc

atcaattttaggagctgtaaattttatcacaacagtaattaatatacgatcaacaggaat

tacatttgatcgaatacctttatttgtttgatctgtaataattacagcaattcttctttt

attatctttaccagttttagctggagctattactatattattaacagatcgaaatttaaa

tacatcattctttgatccagctggaggaggtgatccaattctatatcaacatttattt

>JWDCB038-10|Chrysops nigripes|COI-5P|HM861006

aacactttactttattttcggagcttgagctggaataattggaacatcattaagtatttt

aattcgtgctgaactaggtcatccaggagctttaattggtgatgatcaaatttataatgt

aattgtaactgctcatgcatttgttataattttctttatagtaatacctattataattgg

aggatttggaaattgattagttccattaatattaggagctcctgatatagctttccctcg

aataaataatataagtttttgattacttcctccttcattaacccttttattagttagtag

aatagttgaaaatggaactggaactggttgaactgtttaccctccattatcagctgctat

tgcacatggtggaggatcagttgatttagctattttttcattacatttagctggaatttc

atcaattttaggagctgtaaattttatcacaacagtaattaatatacgatcaacaggaat

tacatttgatcgaatacctttatttgtttgatctgtaataattacagcaattcttctttt

attatctttaccagttttagctggagctattactatattattaacagatcgaaatttaaa

tacatcattctttgatccagctggaggaggtgatccaattctatatcaacatttattt

>JWDCI074-10|Chrysops nigripes|COI-5P|JF877186

aacactttactttattttcggagcttgagctggaataattggaacatcattaagtatttt

aattcgtgctgaactaggtcatccaggagctttaattggtgatgatcaaatttataatgt

aattgtaactgctcatgcatttgttataattttctttatagtaatacctattataattgg

aggatttggaaattgattagttccattaatattaggagctcctgatatagctttccctcg

aataaataatataagtttttgattacttcctccttcattaacccttttattagttagtag

aatagttgaaaatggagctggaactggttgaactgtttaccctccattatcagctgctat

tgcacacggtgggggatcagttgatttagctattttttcattacatttagctggaatttc

atcaattttaggagctgtaaattttatcacaacagtaattaatatacgatcaacaggaat

tacatttgatcgaatacctttatttgtttgatctgtaataattacagcaattcttctttt

attatctttaccagttttagctggagctattactatattattaacagatcgaaatttaaa

tacatcattctttgatccagctggaggaggtgatccaattctatatcaacatttattt

>JWDCJ155-11|Chrysops nigripes|COI-5P|JN303092

aacactttactttattttcggagcttgagctggaataattggaacatcattaagtatttt

aattcgtgctgaactaggtcatccaggagctttaattggtgatgatcaaatttataatgt

aattgtaactgctcatgcatttgttataattttctttatagtaatacctattataattgg

aggatttggaaattgattagttccattaatattaggagctcctgatatagctttccctcg

aataaataatataagtttttgattacttcctccttcattaacccttttattagttagtag

aatagttgaaaatggagctggaactggttgaactgtttaccctccattatcagctgctat

tgcacacggtggaggatcagttgatttagctattttttcattacatttagctggaatttc

atcaattttaggagctgtaaattttatcacaacagtaattaatatacgatcaacaggaat

tacatttgatcgaatacctttatttgtttgatctgtaataattacagcaattcttctttt

attatctttaccagttttagctggagctattactatattattaacagatcgaaatttaaa

tacctcattctttgatccagctggaggaggtgatccaattctatatcaacatttattt

>JWDCI411-10|Chrysops nigripes|COI-5P|KR664677

aacactttactttattttcggtgcttgagctggaataattggaacatcattaagtatttt

aattcgtgctgaactaggtcatccaggagctttaattggtgatgatcaaatttataatgt

aattgtaactgctcatgcatttgttataattttctttatagtaatacctattataattgg

aggatttggaaattgattagttccattaatattaggagctcctgatatagctttccctcg

aataaataatataagtttttgattacttcctccttcattaacccttttattagttagtag

aatagttgaaaatggagctggaactggttgaactgtttaccctccattatcagctactat

tgcacacggtgggggatcagttgatttagctattttttcattacatttagctggaatttc

atcaattttaggagctgtaaattttatcacaacagtaattaatatacgatcaacaggaat

tacatttgatcgaatacctttatttgtttgatctgtaataattacagcaattcttctttt

attatctttaccagttttagctggagctattactatattattaacagatcgaaatttaaa

tacatcattctttgatccagctggaggaggtgatccaattctatatcaacatttattt

>MHTAB089-07|Chrysops mitis|COI-5P|KM285510

aacactttactttattttcggagcttgagctggaataattggaacatcattaagtatttt

aattcgtgctgaactaggtcatccaggagctttaattggtgatgatcaaatttataatgt

aattgtaactgctcatgcatttgttataattttctttatagtaatacctattataattgg

aggatttggaaattgattagttccattaatattaggagctcctgatatagctttccctcg

aataaataatataagtttttgattacttcctccttcattaacccttttattagttagtag

aatagttgaaaatggagctggaactggttgaactgtttaccctccattatcagctgctat

tgcacacggtgggggatcagttgatttagctattttttcattacatttagctggaatttc

atcaattttaggagctgtaaattttatcacaacagtaattaatatacgatcaacaggaat

tacatttgatcgaatacctttatttgtttgatctgtaataattacagcaattcttctttt

attatctttaccagttttagctggagctattactatattattaacagatcgaaatttaaa

tacatcattctttgatccagctggaggaggtgatccaattctatatcaacatttatt-

>JWDCL403-11|Chrysops nigripes|COI-5P|KR671190

aacactttactttattttcggagcttgagctggaataattggaacatcattaagtatttt

aattcgtgctgaactaggtcatccaggagctttaattggtgatgatcaaatttataatgt

aattgtaactgctcatgcatttgttataattttctttatagtaatacctattataattgg

aggatttggaaattgattagttccattaatattaggagctcctgatatagctttccctcg

aataaataatataagtttttgattacttcctccttcattaacccttttattagttagtag

aatagttgaaaatggggctggaactggttgaactgtttaccccccattatcagctgctat

tgcacatggtggaggatcagttgatttagctattttttcattacatttagctggaatttc

atcaattttaggagctgtaaattttatcacaacagtaattaatatacgatcaacaggaat

tacatttgatcgaatacctttatttgtctgatctgtagtaattacagcaattcttctttt

attatctttaccagttttagccggagctattactatattactaacagatcgaaatttaaa

tacatcattctttgatccagctggaggaggtgatccaattctatatcaacatttattt

>JWDCF916-10|Chrysops nigripes|COI-5P|JF875673

aacactttactttattttcggagcttgagctggaataattggaacatcattaagtatttt

aattcgtgctgaactaggtcatccaggagctttaattggtgatgatcaaatttataatgt

aattgtaactgctcatgcatttgttataattttctttatagtaatacctattataattgg

aggatttggaaattgattagttccattaatattaggggctcctgatatagctttccctcg

aataaataatataagtttttgattacttcctccttcattaacccttttattagttagtag

aatagttgaaaatggggctggaactggttgaactgtttaccccccattatcagctgctat

tgcacatggtggaggatcagttgatttagctattttttcattacatttagctggaatttc

atcaattttaggagctgtaaattttatcacaacagtaattaatatacgatcaacaggaat

tacatttgatcgaatacctttatttgtctgatctgtagtaattacagcaattcttctttt

attatctttaccagttttagccggagctattactatattattaacagatcgaaatttaaa

tacatcattctttgatccagctggaggaggtgatccaattctatatcaacatttattt

>DIPFI008-12|Chrysops|COI-5P

aacactttactttattttcggagcttgagctggaataattggaacatcattaagtatttt

aattcgtgctgaactaggtcatccaggagctttaattggtgatgatcaaatttataatgt

aattgtaactgctcatgcatttgttataattttctttatagtaatacctattataattgg

gggatttggaaattgattagttccattaatattaggagctcctgatatagctttccctcg

aataaataatataagtttttgattacttcctccttcattaacccttttattagttagtag

aatagttgaaaatggagctggaactggttgaactgtttaccctccattatcagctgctat

tgcacatggtggaggatcagttgatttagctattttttcattacatttagctggaatttc

atcaattttaggagctgtaaattttatcacaacagtaattaatatacgatcaacaggaat

tacatttgatcgaatacctttatttgtctgatctgtagtaattacagcaattcttctttt

attatctttaccagttttagctggagctattactatattattaacagatcgaaatttaaa

tacatcattctttgacccagctggaggaggtgacccaattctataccaacatttattt

>FIDIP409-11|Chrysops nigripes|COI-5P|Pending (#7798)

aacactttactttattttcggagcttgagctggaataattggaacatcattaagtatttt

aattcgtgctgaactaggtcatccaggagctttaattggtgatgatcaaatttataatgt

aattgtaactgctcatgcatttgttataattttctttatagtaatacctattataattgg

gggatttggaaattgattagttccattaatattaggagctcctgatatagctttccctcg

aataaataatataagtttttgattacttcctccttcattaacccttttattagttagtag

aatagttgaaaatggggctggaactggttgaactgtttaccctccattatcagctgctat

tgcacatggtggagggtcagttgatttagctattttttcattacatctagctggaatttc

atcaattttaggagctgtaaattttatcacaacagtaattaatatacgatcaacaggaat

tacatttgatcgaatacctttatttgtctgatctgtagtaattacagcaattcttctttt

attatctttaccagttttagccggagctattactatattattaacagatcgaaatttaaa

tacgtcattctttgacccagctggaggaggtgacccaattctataccaacatttattt

>FIDIP2951-12|Chrysops sepulcralis|COI-5P|Pending (#7799)

aacactttactttattttcggagcttgagctggaataattggaacatcattaagtatttt

aattcgtgctgaactaggtcatccaggagctttaattggtgatgatcaaatttataatgt

aattgtaactgctcatgcatttgttataattttctttatagtaatacctattataattgg

gggatttggaaattgattagttccattaatattaggagctcctgatatagctttccctcg

aataaataatataagtttttgattacttcctccttcattaacccttttattagttagtag

aatagttgaaaatggggctggaactggttgaactgtttaccctccattatcagctgctat

tgcacatggtggaggatcagttgatttagctattttttcattacatctagctggaatttc

atcaattttaggagctgtaaattttatcacaacagtaattaatatacgatcaacaggaat

tacatttgatcgaatacctttatttgtctgatctgtagtaattacagcaattcttctttt

attatctttaccagttttagccggagctattactatattattaacagatcgaaatttaaa

tacgtcatt-------------------------------------------------

>CNWAE7215-14|Chrysops|COI-5P|KR635972

-------------attttcggagcttgagctggaataattggaacatcattaagtatttt

aattcgtgctgaactaggtcatccaggagctttaattggtgatgatcaaatttataatgt

aattgtaactgctcatgcatttgttataattttctttatagtaatacctattataattgg

aggatttggaaattgattagttccattaatattaggagctcctgatatagctttccctcg

aataaataatataagtttttgattacttcctccttcattaacccttttattagttagtag

aatagttgaaaatggggctggaactggttgaactgtttaccccccattatcagctgctat

tgcacatggtggaggatcagttgatttagctattttttcattacatttagctggaatttc

atcaattttaggagctgtaaattttatcacaacagtaattaatatacgatcaacaggaat

tacatttgatcgaatacctttatttgtctgatctgtagtaattacagcaattcttctttt

attatctttaccagttttagccggagctattactatattattaacagat-----------

----------------------------------------------------------

>SSGBB911-14|Chrysops sordidus|COI-5P|KR525268

-acactttactttatttttggagcttgagctggaataattggaacatcattaagtatttt

aattcgtgctgaactaggtcatccaggagctttaattggtgatgatcaaatttataatgt

aattgtaactgctcatgcatttgttataattttctttatagttatacctattataattgg

tggatttggaaattgattagttccattaatattaggagcccctgatatggcattccctcg

aataaataatataagtttttgattattacctccttcattaaccttgttattagtcagtag

tatagttgaaaatggggctggaactggttgaactgtttaccctccattatcagctgctat

tgcacatggaggaagatcagttgatttagcaattttttcattgcatttagctggaatttc

atcaattttaggagctgtaaattttattacaacagtaattaatatacgatcaacaggaat

tacatttgatcgaatacccttatttgtttgagctgtagttattacagcaattcttctttt

attatctttacctgtattagctggagctattactatattattaacagatcga--------

----------------------------------------------------------

>SSGBB004-14|Chrysops sordidus|COI-5P|KR514749

-acactttactttatttttggagcttgagctggaataattggaacatcattaagtatttt

aattcgtgctgaactaggtcatccaggagctttaattggtgatgatcaaatttataatgt

aattgtaactgctcatgcatttgttataattttctttatagttatacctattataattgg

tggatttggaaattgattagttccattaatattaggagcccctgatatggcattccctcg

aataaataatataagtttttgattattacctccttcattaaccttgttattagtcagtag

tatagttgaaaatggggctggaactggttgaactgtttaccctccattatcagctgctat

tgcacatggaggaagatcagttgatttagcaattttttcattgcatttagctggaatttc

atcaattttaggagctgtaaattttattacaacagtaattaatatacgatcaacaggaat

tacatttgatcgaatacccttatttgtttgagctgtagttattacagcaattcttctttt

attatctttacctgtattagctggagctattactatattattaacagat-----------

----------------------------------------------------------

>SSGBB940-14|Chrysops sordidus|COI-5P|KR516558

-acactttactttatttttggagcttgagctggaataattggaacatcattaagtatttt

aattcgtgctgaactaggtcatccaggagctttaattggtgatgatcaaatttataatgt

aattgtaactgctcatgcatttgttataattttctttatagttatacctattataattgg

tggatttggaaattgattagttccattaatattaggagcccctgatatggcattccctcg

aataaataatataagtttttgattattacctccttcattaaccttgttattagtcagtag

tatagttgaaaatggggctggaactggttgaactgtttaccctccattatcagctgctat

tgcacatggaggaagatcagttgatttagcaattttttcattgcatttagctggaatttc

atcaattttaggagctgtaaattttattacaacagtaattaatatacgatcaacaggaat

tacatttgatcgaatacccttatttgtttgagctgtagttattacagcaattcttctttt

attatctttacctgtattagctggagctattactata-----------------------

----------------------------------------------------------

>ACT061-07|Chrysops dawsoni|COI-5P|KM285450

----------------------------------------ggaacatcactaagtatttt

aattcgtgctgaactaggacacccaggagctttaattggtgatgatcaaatctataatgt

aatcgttactgctcatgcattcgttataattttctttatagttatacctattataatcgg

aggatttggaaattgacttgtaccactaatattaggagcacctgatatagcattccctcg

aataaataatataagtttttgattacttcctccttcattaacccttttattagttagaag

tatagttgaaaacggagctggaactggttgaactgtataccccccattatctgcagctat

tgctcatggaggaggttcagtcgatctagcaattttctctttacatttagctggaatttc

ttcaattttaggagctgtaaattttattacaacagtaattaatatacgatcaacaggaat

cacatttgatcgaatacctctatttgtttgagctgtggtaattacagcaattcttctatt

attatctttacctgttttagctggagctattactatattattaacagatcgaaatttaaa

tacatcattctttgatccagcaggaggaggtgatcctattttatatcaacacctattt

>TTDFW137-08|Chrysops dawsoni|COI-5P|KM571734

aacactttattttatttttggggcttgagctggaataattggaacatcactaagtatttt

aattcgtgctgaactaggacacccaggagctttaattggtgatgatcaaatctataatgt

aatcgttactgctcatgcattcgttataattttctttatagttatacctattataatcgg

aggatttggaaattgacttgtaccactaatattaggagcacctgatatagcattccctcg

aataaataatataagtttttgattacttcctccttcattaacccttttattagttagaag

tatagttgaaaacggagctggaactggttgaactgtataccccccattatctgcagctat

tgctcatggaggaggttcagtcgatctagcaattttctctttacatttagctggaatttc

ttcaattttaggagctgtaaattttattacaacagtaattaatatacgatcaacaggaat

cacatttgatcgaatacctctatttgtttgagctgtagtaattacagcaattcttctact

attatctttacctgttttagctggagctattactatattattaacagatcgaaatttaaa

tacatcattctttgatccagcaggaggaggtgatcctattttatatcaacacctattt

>SSPAC11220-13|Chrysops|COI-5P|KM640362

aacactttattttatttttggggcttgagctggaataattggaacatcactaagtatttt

aattcgtgctgaactaggacacccaggagctttaattggtgatgatcaaatctataatgt

aatcgttactgctcatgcattcgttataattttctttatagttatacctattataatcgg

aggatttggaaattgacttgtaccactaatattaggagcacctgatatagcatttcctcg

aataaataatataagtttttgattacttcctccttcattaacccttttattagttagaag

tatagttgaaaacggggctggaactggttgaactgtataccccccattatctgcagctat

tgctcatggaggaggttcagtcgatctagcaattttctctttacatttagctggaatttc

ttcaattttaggagctgtaaattttattacaacagtaattaatatacgatcaacaggaat

cacatttgatcgaatacctctatttgtttgagctgtggtaattacagcaattcttctatt

attatctttacctgttttagctggagctattactatattattaacagat-----------

----------------------------------------------------------

>MHTAB185-09|Chrysops dawsoni|COI-5P|KM285447

----------------------------------------ggaacatcactaagtatttt

aattcgtgctgaactaggacatccaggagctttaattggtgatgatcaaatttataatgt

aatcgttactgctcatgcattcgtaataattttctttatagttatacctattataattgg

aggatttggaaattgacttgtaccattaatattaggggcacctgatatagcattccctcg

aataaataatataagtttttgattacttcccccttcattaacacttttattagttagaag

tatagttgaaaacggagctggaactggttgaactgtataccccccactatctgcagctat

tgctcatggaggaggttcagtcgacctagcaattttctctttacatttagctggaatttc

ttcaattttaggagctgtaaattttattacaacagtaattaatatacgatcaacaggaat

tacatttgatcgaatacctttatttgtttgagctgtagtaattacagcaattcttctatt

attatctttacctgttttagccggagctattactatattattaacagatcgaaatttaaa

tacatcattctttgatccagcaggaggaggtgaccctattttataccaacacctgttt

>CNPKF2747-14|Chrysops|COI-5P|KR383629

-----tttatt---tatttggggcttgagctggaataattggaacatcactaagtatttt

aattcgtgctgaactaggacatccaggagctttaattggtgatgatcaaatttataatgt

aatcgttactgctcatgcattcgtaataattttctttatagttatacctattataattgg

aggatttggaaattgacttgtaccattaatattaggagcacctgatatagcattccctcg

aataaataatataagtttttgattacttcccccttcattaacacttttattagttagaag

tatagttgaaaacggagctggaactggttgaactgtataccccccactatctgcagctat

tgctcatggaggaggttcagtcgacctagcaattttctctttacatttagctggagtttc

ttcaattttaggagctgtaaattttattacaacagtaattaatatacgatcaacaggaat

tacatttgatcgaatacctttatttgtttgagctgtagtaattacagcaattcttctatt

attatctttacctgttttagccggagctattactatattattaacagatcgaaatttaaa

tacatcattctttgatccagcaggaggaggtgaccctattttataccaacacctattt

>ASDIP904-15|Chrysops cuclux|COI-5P

aacactttattttatttttggggcttgagctggaataattggaacatcactaagtatttt

aattcgtgctgaactagggcatccaggagctttaattggtgatgatcaaatttataatgt

aatcgttactgctcatgcattcgtaataattttctttatagttatacctattataattgg

aggatttggaaattgacttgtaccattaatattaggagcacctgatatagcattccctcg

aataaataatataagtttttgattacttcctccttcattaacacttttattagttagaag

tatagttgaaaatggagctggaactggttgaactgtatatcccccactatctgcagctat

tgctcatggaggaggttcagtcgacctagcaattttctctttacatttagctggaatttc

ttcaattttaggagctgtaaattttattacaacagtaattaatatacgatcaacaggaat

cacatttgatcgaatacctttatttgtttgagctgtagtaattacagcaattcttctatt

attatctttacctgttttagccggagctattactatattattaacagatcgaaatttaaa

tacatcattctttgatccagcaggaggaggtgaccctattttataccaacacctattt

>SSKJB1124-14|Chrysops carbonarius|COI-5P|MF830618

-------------atttttggggcttgagctggaataattggaacatcactaagtatttt

aattcgtgctgaactaggacacccaggagctttaattggtgatgatcaaatttataatgt

aatcgttactgctcatgcattcgttataattttctttatagttatacctattataattgg

aggatttggaaattgacttgtaccattaatattaggagcacctgatatagcattccctcg

aataaataatataagtttttgattacttcccccttcattaacgcttttattagttagaag

tatagttgaaaatggagctggaactggttgaactgtatacccaccattatctgcagctat

tgctcatggaggaggttcagttgatctagcaattttctctttacatctagctggaatttc

ctcaattttaggggctgtaaattttattacaacagtaattaatatacgatcaacaggaat

tacatttgatcgaatacccctatttgtttgagctgtagtaattacagcaattcttctatt

attatctttacctgttttagctggagctattactata-----------------------

----------------------------------------------------------

>SSKJB1117-14|Chrysops carbonarius|COI-5P|MF833189

-------------atttttggggcttgagctggaataattggaacatcactaagtatttt

aattcgtgctgaactaggacacccaggagctttaattggtgatgatcaaatttataatgt

aatcgttactgctcatgcattcgttataattttctttatagttatacctattataattgg

aggatttggaaattgacttgtaccattaatattaggagcacctgatatagcattccctcg

aataaataatataagtttttgattacttcccccttcattaacgcttttattagttagaag

tatagttgaaaatggagctggaactggttgaactgtatacccaccattatctgcagctat

tgctcatggaggaggttcagttgatctagcaattttctctttacatctagctggaatttc

ctcaattttaggggctgtaaattttattacaacagtaattaatatacgatcaacaggaat

tacatttgatcgaatacccctatttgtttgagctgtagtaattacagcaattcttctatt

attatcttta--------------------------------------------------

----------------------------------------------------------

>SSKJA002-14|Chrysops carbonarius|COI-5P|MF834270

-------------atttttggggcttgagctggaataattggaacatcactaagtatttt

aattcgtgctgaactaggacacccaggagctttaattggtgatgatcaaatttataatgt

aatcgttactgctcatgcattcgttataattttctttatagttatacctattataattgg

aggatttggaaattgacttgtaccattaatattaggagcacctgatatagcattccctcg

aataaataatataagtttttgattacttcccccttcattaacgcttttattagttagaag

tatagttgaaaatggagctggaactggttgaactgtatacccaccattatctgcagctat

tgctcatggaggaggttcagttgatctagcaattttctctttacatctagctggaatttc

ctcaattttaggggctgtaaattttattacaacagtaattaatatacgatcaacaggaat

tacatttgatcgaatacccctatttgtttgagctgtagtaattacagcaattcttctatt

attatctttacctgttttagctggagctattactatactattaacagatcgaaatttaaa

tacatcattctttgacccagcaggaggaggtgatccaattttataccaacatttattt

>SSKJB1154-14|Chrysops carbonarius|COI-5P|MF830996

-------------atttttggggcttgagctggaataattggaacatcactaagtatttt

aattcgtgctgaactaggacacccaggagctttaattggtgatgatcaaatttataatgt

aatcgttactgctcatgcattcgttataattttctttatagttatacctattataattgg

aggatttggaaattgacttgtaccattaatattaggagcacctgatatagcattccctcg

aataaataatataagtttttgattacttcccccttcattaacgcttttattagttagaag

tatagttgaaaatggagctggaactggttgaactgtatatccaccattatctgcagctat

tgctcatggaggaggttcagttgatctagcaattttctctttacatctagctggaatttc

ctcaattttaggggctgtaaattttattacaacagtaattaatatacgatcaacaggaat

tacatttgatcgaatacccctatttgtttgagctgtagtaattacagcaattcttctatt

attatctttacctgttttagctggagctattactatactattaacagatcga--------

----------------------------------------------------------

>GMDAP866-12|Chrysops carbonarius|COI-5P|KR640359

aacactttattttatttttggggcttgagctggaataattggaacatcactaagtatttt

aattcgtgctgaactaggacacccaggagctttaattggtgatgatcaaatttataatgt

aatcgttactgctcatgcattcgttataattttctttatagttatacctattataattgg

aggatttggaaattgacttgtaccattaatattaggagcacctgatatagcattccctcg

aataaataatataagtttttgattacttcctccttcattaacgcttttattagttagaag

tatagttgaaaatggagctggaactggttgaactgtatacccaccattatctgcagctat

tgctcatggaggaggttcagttgatctagcaattttctctttacatctagctggaatttc

ctcaattttaggggctgtaaattttattacaacagtaattaatatacgatcaacaggaat

tacatttgatcgaatacctctatttgtttgagctgtagtaattacagcaattcttctatt

attatctttacctgttttagctggagctattactatattattaacagatcgaaatttaaa

tacatcattctttgacccagcaggaggaggtgacccaa--------------------

>SSKJB314-14|Chrysops carbonarius|COI-5P|KR519930

aacactttattttatttttgnggcttgagctggaataattggaacatcactaagtatttt

aattcgtgctgaactaggacacccaggagctttaattggtgatgatcaaatttataatgt

aatcgttactgctcatgcattcgttataattttctttatagttatacctattataattgg

aggatttggaaattgacttgtaccattaatattaggagcacctgatatagcattccctcg

aataaataatataagtttttgattacttcccccttcattaacgcttttattagttagaag

tatagttgaaaatggagctggaactggttgaactgtatacccaccattatctgcagctat

tgctcatggaggaggttcagttgatctagcaattttctctttacatctagctggaatttc

ctcaattttaggggctgtaaattttattacaacagtaattaatatacgatcaacaggaat

tacatttgatcgaatacccctatttgtttgagctgtagtaattacagcaattcttctatt

attatcttta--------------------------------------------------

----------------------------------------------------------

>BBDEC331-09|Chrysops ater|COI-5P|KR431273

aacactttattttatttttggggcttgagctggaataattggaacatcactaagtatttt

aattcgtgctgaactaggtcacccaggagctttaattggtgatgatcaaatttataatgt

aatcgttactgctcatgcattcgttataattttctttatagttatacctattataattgg

gggatttgggaactgacttgtaccattaatattaggagcacctgatatagcattccctcg

aataaataatataagtttttgattactacctccttcattaacccttttattagttagaag

tatagttgaaaacggggctggaactggttgaactgtttaccctccactatctgcagctat

tgcacatggaggaggttccgttgatttagcaattttctctttacatttagctggaatttc

ttcaattttaggagctgtaaattttattacaacagtaattaatatacgatcaacaggaat

tacatttgatcgaataccattatttgtttgagctgtagttattacagcaattcttctttt

attatctttacctgttttagctggagctattactatattattaacagaccgaaatttaaa

tacatcattctttgacccagcaggaggaggtgacccaattttataccaacatttattt

>SSGBB929-14|Chrysops ater|COI-5P|KR522704

-acactttattttatttttggggcttgagctggaataattggaacatcactaagtatttt

aattcgtgctgaactaggtcacccaggagctttaattggtgatgatcaaatttataatgt

aatcgttactgctcatgcattcgttataattttctttatagttatacctattataattgg

gggatttgggaactgacttgtaccattaatattaggagcacctgatatagcattccctcg

aataaataatataagtttttgattactacctccttcattaacccttttattagttagaag

tatagttgaaaacggggctggaactggttgaactgtttaccctccactatctgcagctat

tgcacatggaggaggttccgttgatttagcaattttctctttacatttagctggaatttc

ttcaattttaggagctgtaaattttattacaacagtaattaatatacgatcaacaggaat

tacatttgatcgaataccattatttgtttgagctgtagttattacagcaattcttctttt

attatctttacctgttttagctgga-----------------------------------

----------------------------------------------------------

>SSGBC109-14|Chrysops ater|COI-5P|MF835292

-------------atttttggggcttgagctggaataattggaacatcactaagtatttt

aattcgtgctgaactaggtcacccaggagctttaattggtgatgatcaaatttataatgt

aatcgttactgctcatgcattcgttataattttctttatagttatacctattataattgg

gggatttgggaactgacttgtaccattaatattaggagcacctgatatagcattccctcg

aataaataatataagtttttgattactacctccttcattaacccttttattagttagaag

tatagttgaaaacggggctggaactggttgaactatttaccctccactatctgcagctat

tgcacatggaggaggttccgttgatttagcaattttctctttacatttagctggaatttc

ttcaattttaggagctgtaaattttattacaacagtaattaatatacgatcaacaggaat

tacatttgatcgaataccattatttgtttgagctgtagttattacagcaattcttctttt

attatctttacctgttttagctggagctattactatattattaacagaccga--------

----------------------------------------------------------

>SSGBC105-14|Chrysops ater|COI-5P|MF837801

-------------atttttggggcttgagctggaataattggaacatcactaagtatttt

aattcgtgctgaactaggtcacccaggagctttaattggtgatgatcaaatttataatgt

aatcgttactgctcatgcattcgttataattttctttatagttatacctattataattgg

gggatttgggaactgacttgtaccattaatattaggagcacctgatatagcattccctcg

aataaataatataagtttttgattactacctccttcattaacccttttattagttagaag

tatagttgaaaacggggctggaactggttgaactatttaccctccactatctgcagctat

tgcacatggaggaggttccgttgatttagcaattttctctttacatttagctggaatttc

ttcaattttaggagctgtaaattttattacaacagtaattaatatacgatcaacaggaat

tacatttgatcgaataccattatttgtttgagctgtagttattacagcaattcttctttt

attatctttacctgttttagctggagctattactatattattaaca--------------

----------------------------------------------------------

>SSGBB4350-14|Chrysops ater|COI-5P|KR525881

----------tttatttttggggcttgagctggaataattggaacatcactaagtatttt

aattcgtgctgaactaggtcacccaggagctttaattggtgatgatcaaatttataatgt

aatcgttactgctcatgcattcgttataattttctttatagttatacctattataattgg

gggatttgggaactgacttgtaccattaatattaggagcacctgatatagcattccctcg

aataaataatataagtttttgattactacctccttcattaacccttttattagttagaag

tatagttgaaaacggggctggaactggttgaactgtttaccctccactatctgcagctat

tgcacatggaggaggttccgttgatttagcaattttctctttacatttagctggaatttc

ttcaattttaggagctgtaaattttattacaacagtaattaatatacgatcaacaggaat

tacatttgatcgaataccattatttgtttgagctgtagttattacagcaattcttctttt

attatctttacctgttttagctggagctattactata-----------------------

----------------------------------------------------------

>CNWBB166-13|Chrysops ater|COI-5P|KP041319

-acactttattttatttttggggcttgagctggaataattggaacatcactaagtatttt

aattcgtgctgaactaggtcacccaggagctttaattggtgatgatcaaatttataatgt

aatcgttactgctcatgcattcgttataattttctttatagttatacctattataattgg

aggatttggaaattgacttgtaccattaatattaggagctcctgatatagcattccctcg

aataaataatataagtttttgattactacccccttcattaacccttttattagttagaag

tatagttgaaaatggggctggaactggttgaactgtttaccccccattatctgcagctat

tgctcatggaggaggttccgttgatttagcaattttctctttacatttagctggaatttc

ttcaattttaggagctgtaaattttattacaacagtaattaatatacgatcaacaggaat

cacatttgatcgaataccattatttgtttgagctgtagttattacagcaattcttctttt

attatctttacctgttttagctggagctattactatattattaacagaccga--------

----------------------------------------------------------

>SSPAB231-13|Chrysops ater|COI-5P|KM913376

aacactttattttatttttggggcttgagctggaataattggaacatcactaagtatttt

aattcgtgctgaactaggtcacccaggagctttaattggtgatgatcaaatttataatgt

aatcgttactgctcatgcattcgttataattttctttatagttatacctattataattgg

gggatttggaaattgacttgtaccattaatattaggagctcctgatatagcattccctcg

aataaataatataagtttttgattactacccccttcattaacccttttattagttagaag

tatagttgaaaatggggctggaactggttgaactgtttaccctccattatctgcagctat

tgctcatggaggaggttccgttgatttagcaattttctctttacatttagctggaatttc

ttcaattttaggagctgtaaattttattacaacagtaattaatatacgatcaacaggaat

cacatttgatcgaataccattatttgtttgagctgtagttattacagcaattcttctttt

attatctttacctgttttagctggagctattactatattattaacagac-----------

----------------------------------------------------------

>CNPKF2740-14|Chrysops ater|COI-5P|KR383749

aacactttattttatttttggggcttgagctggaataattggaacatcactaagtatttt

aattcgtgctgaactaggtcacccaggagctttaattggtgatgatcaaatttataatgt

aatcgttactgctcatgcattcgttataattttctttatagttatacctattataattgg

gggatttggaaactgacttgtaccattaatattaggagcacctgatatagcattccctcg

aataaataatataagtttttgattactacccccttcattaacccttttattagttagaag

tatagttgaaaacggagctggaactggttgaactgtttaccccccactatctgcagctat

tgcacatggaggaggttccgttgatttagcaattttctccttacatttagctggaatttc

atcaattttaggagctgtaaattttattacaacagtaattaatatacgatcaacaggaat

tacatttgatcgaataccattatttgtttgagctgtagttattacagcaattc-------

------------------------------------------------------------

----------------------------------------------------------

>CNPKF2707-14|Chrysops ater|COI-5P|KR398611

---------------ttttggggcttgagctggaataattggaacatcactaagtatttt

aattcgtgctgaactaggtcacccaggagctttaattggtgatgatcaaatttataatgt

aatcgttactgctcatgcattcgttataattttctttatagttatacctattataattgg

gggatttggaaactgacttgtaccattaatattaggagcacctgatatagcattccctcg

aataaataatataagtttttgattactacccccttcattaacccttttattagttagaag

tatagttgaaaacggagctggaactggttgaactgtttaccccccactatctgcagctat

tgcacatggaggaggttccgttgatttagcaattttctccttacatttagctggaatttc

atcaattttaggagctgtaaattttattacaacagtaattaatatacgatcaacaggaat

tacatttgatcgaataccattatttgtttgagctgtagttattacagcaattcttctttt

actatctttacctgttttagctggagc---------------------------------

----------------------------------------------------------

>JSDIQ468-10|Chrysops ater|COI-5P|JN302205

---------------------------------aataattggaacatcactaagtatttt

aattcgtgctgaactaggtcacccaggagctttaattggtgatgatcaaatttataatgt

aatcgttactgctcatgcattcgttataattttctttatagttatacctattataattgg

gggatttggaaactgacttgtaccattaatattaggagcacctgatatagcattccctcg

aataaataatataagtttttgattactacccccttcattaacccttttattagttagaag

tatagttgaaaacggagctggaactggttgaactgtttaccccccactatctgcagctat

tgcacatggaggaggttccgttgatttagcaattttctccttacatttagctggaatttc

atcaattttaggagctgtaaattttattacaacagtaattaatatacgatcaacaggaat

tacatttgatcgaataccattatttgtttgagctgtagttattacagcaattcttctttt

actatctttacctgttttagctggagctattactatattattaacagaccgaaatttaaa

tacatcattctttgacccagcaggaggaggtgacccaattttataccaacatttattt

>ACT069-07|Chrysops ater|COI-5P|KM285439

----------------------------------------ggaacatcactaagtatttt

aattcgtgctgaactaggtcacccaggagctttaattggtgatgatcaaatttataatgt

aatcgttactgctcatgcattcgttataattttctttatagttatacctattataattgg

gggatttggaaattgacttgtaccattaatattaggagctcctgatatagcattccctcg

aataaataatawaagtttttgattactacctccttcattaacccttttattagttagaag

tatagttgaaaatggagctggaactggttgaactgtttaccccccattatctgcagctat

tgctcatggaggaggttccgttgatttagcaattttctctttacatttagctggaatttc

ttcaattttaggagctgtaaattttattacaacagtaattaatatacgatcaacaggaat

cacatttgatcgaataccattatttgtttgagctgtagttattacagcaattcttctttt

attatctttacctgttttagctggagctattactatattattaacagaccgaaatttaaa

tacatcattctttgacccagcaggaggaggtgatccaattttatatcaacatttattt

>ACT067-07|Chrysops ater|COI-5P|KM285437

----------------------------------------ggaacatcactaagtatttt

aattcgtgctgaactaggtcacccaggagctttaattggtgatgatcaaatttataatgt

aatcgttactgctcatgcattcgttataattttctttatagttatacctattataattgg

aggatttggaaattgacttgtaccattaatattaggagctcctgatatagcattccctcg

aataaataatataagtttttgattactacccccttcattaacccttttattagttagaag

tatagttgaaaatggggctggaactggttgaactgtttaccccccattatctgcagctat

tgctcatggaggaggttccgttgatttagcaattttctctttacatttagctggaatttc

ttcaattttaggagctgtaaattttattacaacagtaattaatatacgatcaacaggaat

cacatttgatcgaataccattatttgtttgagctgtagttattacagcaattcttctttt

attatctttacctgttttagctggagctattactatattattaacagaccgaaatttaaa

tacatcattctttgacccagcaggaggaggtgatccaattttatatcaacatttattt

>ASDIP295-15|Chrysops excitans|COI-5P

aacactttactttatttttggggcttgagctggaataattggaacatcattaagtatttt

aattcgtgctgaactaggtcacccaggagctttaattggtgatgaccaaatttataatgt

aattgtaactgctcatgcatttattataattttctttatagttatacctattataattgg

aggattcggaaattgacttgtaccattaatattaggagctcctgatatagcattccctcg

aataaataatataagtttttgattactccctccttcattaacacttttattagttagaag

tatagttgaaaatggagctggaacaggatgaactgtatatcccccattatctgcagctat

tgctcatggcggaggatcagtcgatctagcaattttctctttacatttagctggaatttc

ctcaattttaggtgccgtaaattttattacaacagtaattaatatacgatcaacaggaat

tacattcgatcgaatacctttatttgtttgagctgtagttattacagcaattcttctatt

attatctttacctgttttagccggggctattactatactattaacagaccgaaatttaaa

tacatctttttttgacccagcaggaggaggtgatccaattttatatcaacacttattt

>BBDCP614-10|Chrysops|COI-5P|JF868976

aacactttactttatttttggggcttgagctggaataattggaacatcattaagtatttt

aattcgtgctgaactaggtcacccaggagctttaattggtgatgaccaaatttataatgt

aattgtaactgctcatgcatttattataattttctttatagttatacctattataattgg

aggattcggaaattgacttgtaccattaatattaggagctcctgatatagcattccctcg

aataaataatataagtttttgattactccctccttcattaacacttttattagttagaag

tatagttgaaaatggagctggaacaggatgaactgtatatcccccattatctgcaactat

tgctcatggcggaggatcagtcgatctagcaattttctctttacatttagctggaatttc

ctcaattttaggtgccgtaaattttattacaacagtaattaatatacgatcaacaggaat

tacattcgatcgaatacctttatttgtttgagctgtagttattacagcaattcttctatt

attatctttacctgttttagccggggctattactatactattaacagaccgaaatttaaa

tacatctttttttgacccagcaggaggaggtgatccaattttatatcaacacttattt

>ASDIP305-15|Chrysops excitans|COI-5P

aacactttactttatttttggggcttgagctggaataattggaacatcattaagtatttt

aattcgtgctgaactaggtcacccaggagctttaattggtgatgaccaaatttataatgt

aattgtaactgctcatgcatttattataattttctttatagttatacctattataattgg

aggattcggaaattgacttgtaccattaatattaggagctcctgatatagcattccctcg

aataaataatataagtttttgattactccctccttcattaacacttttattagttagaag

tatagttgaaaatggagctggaacaggatgaactgtatatcccccattatctgcagctat

tgctcatggcggaggatcagtcgatctagcaattttctctttacatttagctggaatttc

ctcaattttaggcgccgtaaattttattacaacagtaattaatatacgatcaacaggaat

tacattcgatcgaatacctttatttgtttgagctgtagttattacagcaattcttctatt

attatctttacctgttttagccggggctattactatactattaacagaccgaaatttaaa

tacatctttttttgacccagcaggaggaggtgatccaattttatatcaacacttattt

>TTDFW139-08|Chrysops excitans|COI-5P|KM569988

aacactttactttatttttggggcttgagctggaataattggaacatcattaagtatttt

aattcgtgctgaactaggtcacccaggagctttaattggtgatgaccaaatttataatgt

aattgtaactgctcatgcatttattataattttctttatagttatacctattataattgg

aggattcggaaattgacttgtaccattaatattaggagctcctgatatagcattccctcg

aataaataatataagtttttgattacttcctccttcattaacacttttattagttagaag

tataattgaaaatggagctggaacaggatgaactgtatatcccccattatctgcagctat

tgctcatggtggaggatcagtcgatctagcaattttctctttacatttagctggaatttc

ctcaattttaggtgccgtaaattttattacaacagtaattaatatacgatcaacaggaat

tacattcgatcgaatacctttatttgtttgagctgtagttattacagcaattcttctatt

attatctttacctgttttagccggggctattactatactattaacagaccgaaatttaaa

tacatctttttttgacccagcaggaggaggtgatccaattttatatcaacacttattt

>BBDCP615-10|Chrysops excitans|COI-5P|JF868977

aacactttactttatttttggggcttgagctggaataattggaacatcattaagtatttt

aattcgtgctgaactaggtcacccaggagctttaattggtgatgaccaaatttataatgt

aattgtaactgctcatgcatttattataattttctttatagttatacctattataattgg

aggattcggaaattgacttgtaccattaatattaggagctcctgatatagcattccctcg

aataaataatataagtttttgattactccctccttcattaacacttttattagttagaag

tatagttgaaaatggagctggaacaggatgaactgtatatcccccattatctgcagctat

tgcccatggtggaggatcagtcgatctagcaattttctctttacatttagctggaatttc

ctcaattttaggtgccgtaaattttattacaacagtaattaatatacgatcaacaggaat

tacattcgatcgaatacctttatttgtttgagctgtagttattacagcaattcttctatt

attatctttacctgttttagccggggctattactatactattaacagaccgaaatttaaa

tacatctttttttgacccagcaggaggaggtgatccaattttataccaacacttattt

>ASDIP906-15|Chrysops excitans|COI-5P

aacactttactttatttttggggcttgagctggaataattggaacatcattaagtatttt

aattcgtgctgaactaggtcacccaggagctttaattggtgatgatcaaatttataatgt

aattgtaactgctcatgcatttattataattttctttatagttatacctattataattgg

aggattcggaaattgacttgtaccattaatattaggagctcctgatatagcattccctcg

aataaataatataagtttttgattactccctccttcattaacacttttattagttagaag

tatagttgaaaatggagctggaacaggatgaactgtatatcccccattatctgcagctat

tgcccatggtggaggatcagtcgatctagcaattttctctttacatttagctggaatttc

ctcaattttaggtgccgtaaattttattacaacagtaattaatatacgatcaacaggaat

tacattcgatcgaatacctttatttgtttgagctgtagttattacagcaattcttctatt

attatctttacctgttttagccggggctattactatactattaacagaccgaaatttaaa

tacatctttttttgacccagcaggaggaggtgatccaattttataccaacacttattt

>UAMIC956-13|Chrysops excitans|COI-5P|KU874615

aacactttactttatttttggggcttgagctggaataattggaacatcattaagtatttt

aattcgtgctgaactaggtcacccaggagctttaattggtgatgatcaaatttataatgt

aattgtaactgctcatgcatttattataattttctttatagttatacctattataattgg

aggattcggaaattgacttgtaccattaatattaggagctcctgatatagcattccctcg

aataaataatataagtttttgattactccctccttcattaacacttttattagttagaag

tatagttgaaaatggagctggaacaggatgaactgtatatcccccattatctgcagctat

tgcccatggtggaggatcagtcgatctagcaattttctctttacatttagctggaatttc

ctcaattttaggtgccgtaaattttattacaacagtaattaatatacgatcaacaggaat

tacattcgatcgaatacctttatttgtttgagctgtagttattacagcaattcttctatt

attatctttacctgttttagctggggctattactatactattaacagaccgaaatttaaa

tacatctttttttgacccagcaggaggaggtgatccaattttataccaacacttattt

>ASDIP740-15|Chrysops excitans|COI-5P

aacactttactttatttttggggcttgagctggaataattggaacatcattaagtatttt

aattcgtgctgaactaggtcacccaggagctttaattggtgatgaccaaatttataatgt

aattgtaactgctcatgcatttattataattttctttatagttatacctattataattgg

aggattcggaaattgacttgtaccattaatattaggagctcctgatatagcattccctcg

aataaataatataagtttttgattactccctccttcattaacacttttattagttagaag

tatagttgaaaatggagctggaacaggatgaactgtatatcccccattatctgcagctat

tgcccatggtggagggtcagtcgatctagcaattttctctttacatttagctggaatttc

ctcaattttaggtgccgtaaattttattacaacagtaattaatatacgatcaacaggaat

tacattcgatcgaatacctttatttgtttgagctgtagttattacagcaattcttctatt

attatctttacctgttttagccggggctattactatactattaacagaccgaaatttaaa

tacatctttttttgacccagcaggaggaggtgatcc----------------------

>BBDEC330-09|Chrysops excitans|COI-5P|KR437255

aacactttactttatttttggggcttgagctggaataattggaacatcattaagtatttt

aattcgtgctgaactaggtcacccaggagctttaattggtgatgatcaaatttataatgt

aattgtaactgctcatgcatttattataattttctttatagttatacctattataattgg

aggattcggaaattgacttgtaccattaatattaggagctcctgatatagcattccctcg

aataaataatataagtttttgattactccctccttcattaacacttttattagttagaag

tatagttgaaaatggagctggaacaggttgaactgtataccccccattatctgcagctat

tgcccatggtggaagatcagtcgatctagcaattttctctttacatttagctggaatttc

ctcaattttaggtgccgtaaattttattacaacagtaattaatatacgatcaacaggaat

tacattcgatcgaatacctttatttgtttgagctgtagttattacagcaattcttctatt

attatctttacctgttttagccggggctattactatactattaacagaccgaaatttaaa

tacatctttttttgacccagcaggaggaggtgatc-----------------------

>CNPKG1439-14|Chrysops|COI-5P|KR385795

aacactttactttatttttggggcttgagctggaataattggaacatcattaagtatttt

aattcgtgctgaactaggtcacccaggagctttaattggtgatgaccaaatttataatgt

aattgtaactgctcatgcatttattataattttctttatagttatacctattataattgg

aggattcggaaattgacttgtaccattaatattaggagctcctgatatagcattccctcg

aataaataatataagtttttgattactccctccttcattaacacttttattagttagaag

tatagttgaaaatggagctggaacaggatgaactgtatatcccccattatctgcagctat

tgctcatggtggaggatcagtcgatctagcaattttctctttacatttagctggaatttc

ctcaattttaggtgccgtaaattttattacaacagtaattaatatacgatcaacaggaat

tacattcgatcgaatacctttatttgtttgagctgtagttattacagcaattcttctatt

attatctttacctgttttagccggggctattactatactattaacagaccg---------

----------------------------------------------------------

>ASDIP912-15|Chrysops excitans|COI-5P

aacactttactttatttttggggcttgagctggaataattggaacatcattaagtatttt

aattcgtgctgaactaggtcacccaggagctttaattggtgatgaccaaatttataatgt

aattgtaactgctcatgcatttattataattttctttatagttatacctattataattgg

gggattcggaaattgacttgtaccattaatattaggagctcctgatatagcattccctcg

aataaataatataagtttttgattactccctccttcattaacacttttattagttagaag

tatagttgaaaatggagctggaacaggatgaactgtataccccccattatctgcagctat

tgctcatggtggaggatcagtcgatctagcaattttctctttacatttagctggaatttc

ctcaattttaggtgccgtaaattttattacaacagtaattaatatacgatcaacaggaat

tacattcgatcgaatacctttatttgtttgagctgtagttattacagcaattcttctatt

attatctttacctgttttagccggggctattactatactattaacagaccgaaatttaaa

tacatctttttttgacccagcaggag--------------------------------

>CNPKG1425-14|Chrysops|COI-5P|KR395221

aacactttactttatttttggggcttgagctggaataattggaacatcattaagtatttt

aattcgtgctgaactaggtcacccaggagctttaattggtgatgaccaaatttataatgt

aattgtaactgctcatgcatttattataattttctttatagttatacctattataattgg

aggattcggaaattgacttgtaccattaatattaggagctcctgatatagcattccctcg

aataaataatataagtttttgattactccctccttcattaacacttttattagttagaag

tatagttgaaaatggagctggaacaggatgaactgtatatcccccattatctgcagctat

tgctcatggtggaggatcagtcgatctagcaattttctctttacatttagctggaatttc

ctcaattttaggtgccgtaaattttattacaacagtaattaatatacgatcaacaggaat

tacattcgatcgaatacctttatttgtttgagctgtagttattacagcaattcttctatt

attatctttacctgttttagccggggctatt-----------------------------

----------------------------------------------------------

>SSPAA2950-13|Chrysops|COI-5P|KM901804

aacactttactttatttttggggcttgagctggaataattggaacatcattaagtatttt

aattcgtgctgaactaggtcacccaggagctttaattggtgatgaccaaatttataatgt

aattgtaactgctcatgcatttattataattttctttatagttatacctattataattgg

aggattcggaaattgacttgtaccattaatattaggagctcctgatatagcattccctcg

aataaataatataagtttttgattactccctccttcattaacacttttattagttagaag

tatagttgaaaacggagctggaacaggatgaactgtatatcccccattatctgcagctat

tgcccatggtggaggatcagtcgatctagcaattttctctttacatttagctggaatttc

ctcaattttaggtgccgtaaattttattacaacagtaattaatatacgatcaacaggaat

tacattcgatcgaatacctttatttgtttgagctgtagttattacagcaattc-------

------------------------------------------------------------

----------------------------------------------------------

>SSPAC11172-13|Chrysops|COI-5P|KM637563

aacactttactttatttttggggcttgagctggaataattggaacatcattaagtatttt

aattcgtgctgaactaggtcacccaggagctttaattggtgatgaccaaatttataatgt

aattgtaactgctcatgcatttattataattttctttatagttatacctattataattgg

aggattcggaaattgacttgtaccattaatattaggggctcctgatatagcattccctcg

aataaataatataagtttttgattactccctccttcattaacacttttattagttagaag

tatagttgaaaacggagctggaacaggatgaactgtatatcccccattatctgcagctat

tgcccatggtggaggatcagtcgatctagcaattttctctttacatttagctggaatttc

ctcaattttaggtgccgtaaattttattacaacagtaattaatatacgatcaacaggaat

tacattcgatcgaatacctttatttgtttgagctgtagttattacagcaattc-------

------------------------------------------------------------

----------------------------------------------------------

>SSPAC11176-13|Chrysops|COI-5P|KM632464

aacactttactttatttttggggcttgagctggaataattggaacatcattaagtatttt

aattcgtgctgaactaggtcacccaggagctttaattggcgatgaccaaatttataatgt

aattgtaactgctcatgcatttattataattttctttatagttatacctattataattgg

aggattcggaaattgacttgtaccattaatattaggagctcctgatatagcattccctcg

aataaataatataagtttttgattactccctccttcattaacacttttattagttagaag

tatagttgaaaatggagctggaacaggatgaactgtatatcccccattatctgcagctat

tgctcatggtggaggatcagtcgatctagcaattttctctttacatttagctggaatttc

ctcaattttaggtgccgtaaattttattacaacagtaattaatatacgatcaacaggaat

tacattcgatcgaatacctttatttgtttgagctgtagttattacagcaattc-------

------------------------------------------------------------

----------------------------------------------------------

>SSPAA2932-13|Chrysops excitans|COI-5P|MG476212

aacactttactttatttttggggcttgagctggaataatnggaacatcattaagtatttt

aattcgtgctgaactagntcacccaggagctttaattggtgatgaccaaatttataatgt

aattgtaactgctcatgcatttattataattttctttatagttatacctattataattgg

aggattcggaaattgacttgtaccattaatattaggagctcctgatatagcattccctcg

aataaataatataagtttttgattactccctccttcattaacacttttattagttagaag

tatagttgaaaacggagctggaacaggatgaactgtatatcccccattatctgcagctat

tgcccatggtggaggatcagtcgatctagcaattttctctttacatttagctggaatttc

ctcaattttaggtgccgtaaattttattacaacagtaattaatatacgatcaacaggaat

tacattcgatcgaatacctttatttgtttgagctgtagtt--------------------

------------------------------------------------------------

----------------------------------------------------------

>SSPAA2917-13|Chrysops excitans|COI-5P|MG475677

aacactttactttatttttggggcttgagctggaataattggaacatcattaagtatttt

aattcgtgctgaactagntcacccaggagctttaattggtgatgaccaaatttataatgt

aattgtaactgctcatgcatttattataattttctttatagttatacctattataattgg

aggattcggaaattgacttgtaccattaatattaggagctcctgatatagcattccctcg

aataaataatataagtttttgattactccctccttcattaacacttttattagttagaag

tatagttgaaaacggagctggaacaggatgaactgtatatcccccattatctgcagctat

tgcccatggtggaggatcagtcgatctagcaattttctctttacatttagctggaatttc

ctcaattttaggtgccgtaaattttattacaacagtaattaatatacgatcaacaggaat

tacattcgatcgaatacctttatttgtttgagctgtagtt--------------------

------------------------------------------------------------

----------------------------------------------------------

>CNPKH740-14|Chrysops excitans|COI-5P|MG472526

-acactttactttatttttggggcttgagctggaataattggaacatcattaagtatttt

aattcgtgctgaactaggtcacccaggagctttaattggtgatgaccaaatttataatgt

aattgtaactgctcatgcatttattataattttctttatagttatacctattataattgg

aggattcggaaattgacttgtaccattaatattaggagctcctgatatagcattccctcg

aataaataatataagtttttgattactccctccttcattaacacttttattagttagaag

tatagttgaaaatggagctggaacaggatgaactgtatatcccccattatctgcagctat

tgctcatggtggaggatcagtcgatctagcaattttctctttacatttagctggaatttc

ctcaattttaggtgccgtaaattttattacaacagtaattaatatacgatcaacaggaat

tacattcgatcgaatncctttatttgtttgagctgtagttatt-----------------

------------------------------------------------------------

----------------------------------------------------------

>SSPAB1642-13|Chrysops|COI-5P|KM911208

-acactttactttattttnggggcttgagctggaataattngaacatcattaagtatttt

aattcgtgctgaactaggtcacccaggagctttaattggtgatgaccaaatttataatgt

aattgtaactgctcatgcatttattataattttctttatagttatacctattataattgg

aggattcgggaattgacttgtaccattaatattaggagctcctgatatagcattccctcg

aataaataatataagtttttgattactccctccttcattaacacttttattagttagaag

tatagttgaaaatggagctggaacaggatgaactgtatatcccccattatctgcagctat

tgcccatggtggaggatcagtcgatctagcaattttctctttacatttagctggaatttc

ctcaattttaggtgccgtaaattttattacaacagtaattaatatacgatcaacaggaat

tacattcgatcgaatacctttatttgtttgagctgtagttattacagcaattcttctatt

attatctttacctgttttagcc--------------------------------------

----------------------------------------------------------

>CNMIE1008-14|Chrysops|COI-5P|KR387629

-------------------------tgagctggaataattggaacatcattaagtatttt

aattcgtgctgaactaggtcacccaggagctttaattggtgatgatcaaatttataatgt

aattgtaactgctcatgcatttattataattttctttatagttatacctattataattgg

aggattcggaaattgacttgtaccattaatattaggagctcctgatatagcattccctcg

aataaataatataagtttttgattactccctccttcattaacacttttattagttagaag

tatagttgaaaatggagctggaacaggttgaactgtataccccccattatctgcagctat

tgcccatggtggaggatcagtcgatctagcaattttctctttacatttagctgggatttc

ctcaattttaggtgccgtaaattttattacaacagtaattaatatacgatcaacaggaat

tacattcgatcgaatacctttatttgtttgagctgtagttattacagcaattcttctatt

attatctttacctgttttagccggg-----------------------------------

----------------------------------------------------------

>SSGBB2132-14|Chrysops|COI-5P|KR520239

----------------------------------------------------agtatttt

aattcgtgctgaactaggtcacccaggagctttaattggtgatgatcaaatttataatgt

aattgtaactgctcatgcatttattataattttctttatagttatacctattataattgg

aggattcggaaattgacttgtaccattaatattaggagctcctgatatagcattccctcg

aataaataatataagtttttgattactccctccttcattaacacttttattagttagaag

tatagttgaaaatggagctggaacaggttgaactgtataccccccattatctgcagctat

tgcccatggtggaggatcagtcgatctagcaattttctctttacatttagctgggatttc

ctcaattttaggtgccgtaaattttattacaacagtaattaatatacgatcaacaggaat

tacattcgatcgaatacctttatttgtttgagctgtagttattacagcaattcttctatt

attatctttacctgttttagccggg-----------------------------------

----------------------------------------------------------

>SSPAB1633-13|Chrysops|COI-5P|KM907983

-------------------------------ggaataattggaacatcattaagtatttt

aattcgtgctgaactaggtcacccaggagctttaattggtgatgaccaaatttataatgt

aattgtaactgctcatgcatttattataattttctttatagttatacctattataattgg

aggattcggaaattgacttgtaccattaatattaggagctcctgatatagcattccctcg

aataaataatataagtttttgattactccctccttcattaacacttttattagttagaag

tatagttgaaaatggagctggaacaggatgaactgtatatcccccattatctgcagctat

tgctcatggtggaggatcagtcgatctagcaattttctctttacatttagctggaatttc

ctcaattttaggtgccgtaaattttattacaacagtaattaatatacgatcaacaggaat

tacattcgatcgaatacctttatttgtttgagctgtagttattacagcaattcttctatt

attatctttacctgttttagccggggctattactata-----------------------

----------------------------------------------------------

>SSPAA2990-13|Chrysops excitans|COI-5P|MG477222

--------actttatttttggggcttgagctggaataattggaacatcattaagtatttt

aattcgtgctgaactaggtcacccaggagctttaattggtgatgaccaaatttataatgt

aattgtaactgctcatgcatttattataattttctttatagttatacctattataattgg

aggattcggaaattgacttgtaccattaatattaggagctcctgatatagcattccctcg

aataaataatataagtttttgattactccctccttcattaacacttttattagttagaag

tatagttgaaaatggagctggaacaggatgaactgtatatcccccattatctgcagctat

tgcccatggtggaggatcagtcgatctagcaattttctctttacatttagctggaatttc

ctcaattttaggtgccgtaaattttattacaacagtaattaatatacgatcaacaggaat

tacattcgatcgaatacctttatttgtttgagctgtagtt--------------------

------------------------------------------------------------

----------------------------------------------------------

>MHTAB182-09|Chrysops excitans|COI-5P|KM285460

----------------------------------------ggaacatcattaagtatttt

aattcgtgctgaactaggtcacccaggagctttaattggtgatgatcaaatttataatgt

aattgtaactgctcatgcatttattataattttctttatagttatacctattataattgg

aggattcggaaattgacttgtaccattaatattaggagctcctgatatagcattccctcg

aataaataatataagtttttgattactccctccttcattaacacttttattagttagaag

tatagttgaaaatggagctggaacaggttgaactgtataccccccattatctgcagctat

tgcccatggtggaggatcagtcgatctagcaattttctctttacatttagctgggatttc

ctcaattttaggtgccgtaaattttattacaacagtaattaatatacgatcaacaggaat

tacattcgatcgaatacctttatttgtttgagctgtagttattacagcaattcttctatt

attatctttacctgttttagccggggctattactatactattaacagaccgaaatttaaa

tacatctttttttgacccagcaggaggaggtgatccaattttataccaacacttattt

>MHTAB205-09|Chrysops excitans|COI-5P|KM285451

----------------------------------------ggaacatcattaagtatttt

aattcgtgctgaactaggtcacccaggagctttaattggtgatgatcaaatttataatgt

aattgtaactgcccatgcatttattataattttctttatagttatacctattataattgg

aggattcggaaattgacttgtaccattaatattaggagctcctgatatagcattccctcg

aataaataatataagtttttgattactccctccttcattaacacttttattagttagaag

tatagttgaaaatggagctggaacaggttgaactgtataccccccattatctgcagctat

tgcccatggtggaggatcagtcgatctagcaattttctctttacatttagctgggatttc

ctcaattttaggtgccgtaaattttattacaacagtaattaatatacgatcaacaggaat

tacattcgatcgaatacctttatttgtttgagctgtagttattacagcaattcttctatt

attatctttacctgttttagccggggctattactatactattaacagaccgaaatttaaa

tacatctttttttgacccagcaggaggaggtgatccaattttataccaacacttattt

>MHTAB177-09|Chrysops excitans|COI-5P|KM285474

----------------------------------------ggaacatcattaagtatttt

aattcgtgctgaactaggtcacccaggagctttaattggtgatgaccaaatttataatgt

aattgtaactgctcatgcatttattataattttctttatagttatacctattataattgg

aggattcggaaattgacttgtaccattaatattaggagctcctgatatagcattccctcg

aataaataatataagtttttgattactccctccttcattaacacttttattagttagaag

tatagttgaaaatggagctggaacaggatgaactgtatatcccccattatctgcagctat

tgcccatggtggagggtcagtcgatctagcaattttctctttacatttagctggaatttc

ctcaattttaggtgccgtaaattttattacaacagtaattaatatacgatcaacaggaat

tacattcgatcgaatacctttatttgtttgagctgtagttattacagcaattcttctatt

attatctttacctgttttagccggggctattactatactattaacagaccgaaatttaaa

tacatctttttttgacccagcaggaggaggtgatccaattttataccaacacttattt

>SSKJC627-15|Chrysops|COI-5P|MF830763

-acactttactttatttttggggcttgagctggaataattggaacatcattaagtatntt

aattcgtgctgaactaggtcatccaggagctttaattggtgatgatcaaatttataacgt

aatngtaactgctcatgcatttattataattttctttatagttatacctattataattgg

gggattcggaaattgacttgtaccattaatattaggagctcctgatatagcattccctcg

aataaataatataagtttttgattactccctccttcattaacacttttattagttagaag

tatagttgaaaacggagctggaacaggttgaactgtataccccccattatctgcagctat

tgcccatggcggaggatcagtcgatctagcaattttctctttacatttagctggaatttc

ctcaattttaggggccgtaaattttattacaacagtaattaatatacgatcaacaggaat

tacattcgatcgaatacctttattcgtttgagctgtagttattacagcaattcttctatt

attatctttacctgttttagcnggagctattactatattattaacagaccgaaatttaaa

tacatct---------------------------------------------------

>SSPAC599-13|Chrysops|COI-5P|KM635471

-acactttactttatttttggggcttgagctggaataattggaacatcattaagtatttt

aattcgtgctgaactaggtcacccaggagctttaattggtgatgatcaaatttataacgt

aattgtaactgctcatgcatttgttataattttctttatagttatacctattataattgg

aggattcggaaattgacttgttccattaatattaggagctcctgatatagcattccctcg

aataaataatataagtttttgattactcccaccttcattaacacttttattagttagaag

tatagttgaaaacggggctggaacaggttgaactgtataccccccattatctgcagctat

tgctcatggtggaggatcagtcgatctagcaattttctctttacatctagctggaatttc

ctcaattttaggagccataaattttattacaacagtaattaatatacgatcaacaggaat

tacattcgatcgaatacctttatttgtttgagctgtagttattacagcaattcttctatt

attatctttacctgttttagctggggctattactatattactaacagac-----------

----------------------------------------------------------

>SSPAC613-13|Chrysops|COI-5P|KM639672

-acactttactttatttttggggcttgagctggaataattggaacatcattaagtatttt

aattcgtgctgaactaggtcacccaggagctttaattggtgatgatcaaatttataacgt

aattgtaactgctcatgcatttgttataattttctttatagttatacctattataattgg

gggattcggaaattgacttgttccattaatattaggagcccctgatatagcattccctcg

aataaataatataagtttttgattactcccaccttcattaacacttttattagttagaag

tatagttgaaaacggggctggaacaggttgaactgtataccccccattatctgcagctat

tgcccatggtggaggatcagtcgatctagcaattttctctttacatttagctggaatttc

ctcaattttaggagccgtaaattttattacaacagtaattaatatacgatcaacaggaat

tacattcgatcgaatacctttatttgtttgagctgtagttattacagcaattctgctatt

attatctttacctgttttagctggggctattactatattactaacagac-----------

----------------------------------------------------------

>CNPAD1108-13|Chrysops|COI-5P|KR594474

----------------------------------------------------agtatttt

aattcgtgctgaactaggtcacccaggagctttaattggtgatgatcaaatttataacgt

aattgtaactgctcatgcatttgttataattttctttatagttatacctattataattgg

aggattcggaaattgacttgttccattaatattaggagctcctgatatagcattccctcg

aataaataatataagtttttgattactcccaccttcattaacacttttattagttagaag

tatagttgaaaacggggctggaacaggttgaactgtataccccccattatctgcagctat

tgctcatggtggaggatcagtcgatctagcaattttctctttacatctagctggaatttc

ctcaattttaggagccgtaaattttattacaacagtaattaatatacgatcaacaggaat

tacattcgatcgaatacctttatttgtttgagctgtagttattacagcaattcttctatt

attatctttacctgttttagctggggctattactatattactaacagac-----------

----------------------------------------------------------

>SSPAC611-13|Chrysops|COI-5P|KM645025

-acactttactttatttttggggcttgagctggaataattggaacatcattaagtatttt

aattcgtgctgaactaggtcacccaggagctttaattggtgatgatcaaatttataacgt

aattgtaactgctcatgcatttgttataattttctttatagttatacctattataattgg

agggttcggaaattgacttgttccattaatattaggagctcctgatatagcattccctcg

aataaataatataagtttttgattactcccgccttcattaacacttttattagttagaag

tatagttgaaaatggggctggaacaggttgaactgtataccccccattatctgcagctat

tgcccatggtggaggatcagtcgatctagcaattttctctttacatttagctggaatttc

ctcaattttaggagccgtaaattttattacaacagtaattaatatacgatcaacaggaat

tacattcgatcgaatacctttatttgtttgagctgtagttattacagcaattcttctatt

attatctttacctgttttagctggggctattactatattactaacagaccga--------

----------------------------------------------------------

>SSPAC610-13|Chrysops|COI-5P|KM647309

-acactttactttatttttggggcttgagctggaataattggaacatcattaagtatttt

aattcgtgctgaactaggtcacccaggagctttaattggtgatgatcaaatttataacgt

aattgtaactgctcatgcatttgttataattttctttatagttatacctattataattgg

ggggttcggaaattgacttgttccattaatattaggagctcctgatatagcattccctcg

aataaataatataagtttttgattactcccgccttcattaacacttttattagttagaag

tatagttgaaaatggggctggaacaggttgaactgtataccccccattatctgcagctat

tgcccatggtggaggatcagtcgatctagcaattttctctttacatttagctggaatttc

ctcaattttaggagccgtaaattttattacaacagtaattaatatacgatcaacaggaat

tacattcgatcgaatacctttatttgtttgagctgtagttattacagcaattcttctatt

attatctttacctgttttagctggggctattactata-----------------------

----------------------------------------------------------

>SSKUB213-15|Chrysops|COI-5P|MF838080

-acactttactttatttttggggcttgagctggaataattggaacatcattaagtatttt

aattcgtgctgaactaggtcacccaggagctttaattggtgatgatcaaatttataacgt

aattgtaactgctcatgcatttgttataattttctttatagttatacctattataattgg

aggattcggaaattgacttgtaccattaatattaggagctcctgatatagcattccctcg

aataaataatataagtttttgattactcccgccttcattaacacttttattagttagaag

tatagttgaaaacggagctggaacaggttgaactgtataccccccattatctgcagctat

tgctcatggtggaggatcagtcgatctagcaattttctctttacatttagctggaatctc

ctcaattttaggagccgtaaattttattacaacagtaattaatatacgatcaacaggaat

tacattcgatcgaatacctttatttgtttgagctgtagttattacagcaattcttctatt

attatctttacctgttttagctggggctattactatattacta-----------------

----------------------------------------------------------

>TTDBW224-09|Chrysops mitis|COI-5P|MG169200

-acactttactttatttttggggcttgagctggaataattggaacatcattaagtatttt

aattcgtgctgaactaggtcacccaggagctttaattggtgatgatcaaatttataacgt

aattgtaactgctcatgcatttgttataattttctttatagttatacctattataattgg

aggattcggaaattgacttgtaccattaatattaggagctcctgatatagcattccctcg

aataaataatataagtttttgattactcccgccttcattaacacttttattagttagaag

tatagttgaaaacggagctggaacaggttgaactgtataccccccattatctgcagctat

tgctcatggtggaggatcagtcgatctagcaattttctctttacatttagctggaatctc

ctcaattttaggagccgtaaattttattacaac---------------------------

------------------------------------------------------------

------------------------------------------------------------

----------------------------------------------------------

>SSKJB1155-14|Chrysops|COI-5P|MF835256

-acactttactttatttttggggcttgagctggaataattggaacatcattaagtatttt

aattcgtgctgaactaggtcacccaggagctttaattggtgatgatcaaatttataacgt

aattgtaactgctcatgcatttgttataattttctttatagttatacctattataattgg

aggattcggaaattgacttgtaccattaatattaggagctcctgatatagcattccctcg

aataaataatataagtttttgattactcccaccttcattaacacttttattagttagaag

tatagttgaaaacggagctggaacaggttgaactgtataccccccattatctgcagctat

tgctcatggtggaggatcagtcgatctagcaattttctctttacatttagctggaatttc

ctcaattttaggagccgtaaattttattacaacagtaattaatatacgatcaacaggaat

tacattcgatcgaatacctttatttgtttgagctgtagttattacagcaattcttctatt

attatctttacctgttttagctggggctattactata-----------------------

----------------------------------------------------------

>SSKJC2180-15|Chrysops|COI-5P|MF830748

-acactttactttatttttngggcttgagctggaataattggaacatcattaagtatttt

aattcgtgctgaactaggtcacccaggagctttaattggtgatgatcaaatttataacgt

aattgtaactgctcatgcatttgttataattttctttatagttatacctattataattgg

aggattcgggaattgacttgtaccattaatattaggagctcctgatatagcattccctcg

aataaataatataagtttttgattactcccaccttcattaacacttttattagttagaag

tatagttgaaaacggagctggaacaggttgaactgtataccccccattatctgcagctat

tgctcatggtggaggatcagtcgatctagcaattttctctttacatttagctggaatttc

ctcaattttaggagccgtaaattttattacaacagtaattaatatacgatcaacaggaat

tacattcgatcgaatacctttatttgtttgagctgtagttattacagcaattcttctatt

attatctttacctgttttagctggggctattactatattactaacagatcgaaatttaaa

tacatct---------------------------------------------------

>SSPAB1644-13|Chrysops|COI-5P|KM898023

----------------------------------------------------------tt

aattcgtgctgaactaggtcacccaggagctttaattggtgatgatcaaatttataacgt

aattgtaactgctcatgcatttgttataattttctttatagttatacctattataattgg

aggattcggaaattgacttgttccattaatattaggagctcctgatatagcattccctcg

aataaataatataagtttttgattacttccgccttcattaacacttttattagttagaag

tatagttgaaaacggggctggaacaggttgaactgtataccccccattatctgcagctat

tgcccatggtggaggatcagtcgatctagcaattttctctttacatttagctggaatttc

ctcaattttaggagccgtaaattttattacaacagtaattaatatacgatcaacaggaat

tacattcgatcgaatacctttatttgtttgagctgtagttattacagcaattcttctatt

attatctttacctgttttagctggggctattactata-----------------------

----------------------------------------------------------

>SSPAB277-13|Chrysops|COI-5P|KM906690

aacactttactttatttttggggcttgagctggaataattggaacatcattaagtatttt

aattcgtgctgaactaggtcacccaggagctttaattggtgatgatcaaatttataacgt

aattgtaactgctcatgcatttgttataattttctttatagttatacctattataattgg

gggattcggaaattgacttgttccattaatattaggagcccctgatatagcattccctcg

aataaataatataagtttttgattactcccaccttcattaacacttttattagttagaag

tatagttgaaaacggggctggaacaggttgaactgtataccccccattatctgcagctat

tgcccatggtggaggatcagtcgatctagcaattttctctttacatttagctggaatttc

ctcaattttaggagccgtaaattttattacaacagtaattaatatacgatcaacaggaat

tacattcgatcgaatacctttatttgtttgagctgtagttattacagcaattctgctatt

attatctttacctgttttagctggggctattactatattactaacagaccga--------

----------------------------------------------------------

>SSPAB270-13|Chrysops|COI-5P|KM912344

aacactttactttatttttggggcttgagctggaataattggaacatcattaagtatttt

aattcgtgctgaactaggtcacccaggagctttaattggtgatgatcaaatttataacgt

aattgtaactgctcatgcatttgttataattttctttatagttatacctattataattgg

gggattcggaaattgacttgttccattaatattaggagcccctgatatagcattccctcg

aataaataatataagtttttgattactcccaccttcattaacacttttattagttagaag

tatagttgaaaacggggctggaacaggttgaactgtataccccccattatctgcagctat

tgcccatggtggaggatcagtcgatctagcaattttctctttacatttagctggaatttc

ctcaattttaggagccgtaaattttattacaacagtaattaatatacgatcaacaggaat

tacattcgatcgaatacctttatttgtttgagctgtagttattacagcaattctgctatt

attatctttacctgttttagctggggctattactatattactaacagaccg---------

----------------------------------------------------------

>SSPAB288-13|Chrysops|COI-5P|KM908959

aacactttactttatttttggggcttgagctggaataattggaacatcattaagtatttt

aattcgtgctgaactaggtcacccaggagctttaattggtgatgatcaaatttataacgt

aattgtaactgctcatgcatttgttataattttctttatagttatacctattataattgg

gggattcggaaattgacttgttccattaatattaggagcccctgatatagcattccctcg

aataaataatataagtttttgattactcccaccttcattaacacttttattagttagaag

tatagttgaaaacggggctggaacaggttgaactgtataccccccattatctgcagctat

tgcccatggtggaggatcagtcgatctagcaattttctctttacatttagctggaatttc

ctcaattttaggagccgtaaattttattacaacagtaattaatatacgatcnncnngaat

tacattcgatcgaatacctttatttgtttgagctgtagttattacagcaattctgctatt

attatctttacctgttttagctggggctattactatattactaacagacc----------

----------------------------------------------------------

>SSPAC6892-13|Chrysops|COI-5P|KM649627

aacactttactttatttttggggcttgagctggaataattggaacatcattaagtatttt

aattcgtgctgaactaggtcacccaggagctttaattggtgatgatcaaatttataacgt

aattgtaactgctcatgcatttgttataattttctttatagttatacctattataattgg

aggattcggaaattgacttgttccattaatattaggagctcctgatatagcattccctcg

aataaataatataagtttttgattactcccaccttcattaacacttttattagttagaag

tatagttgaaaacggggctggaacaggttgaactgtataccccccattatctgcagctat

tgctcatggtggaggatcagtcgatctagcaattttctctttacatctagctggaatttc

ctcaattttaggagccgtaaattttattacaacagtaattaatatacgatcancnngaat

tacattcgatcgaatacctttatttgtttgagctgtagttattacagcaattcttctatt

attatctttacctgttttagctggggctattactata-----------------------

----------------------------------------------------------

>CNGBL031-14|Chrysops|COI-5P|KR390359

aacactttactttatttttggggcttgagctggaataattggaacatcattaagtatttt

aattcgtgctgaactaggtcacccaggagctttaattggtgatgatcaaatttataacgt

aattgtaactgctcatgcatttgttataattttctttatagttatacctattataattgg

aggattcggaaattgacttgttccattaatattaggagctcctgatatagcattccctcg

aataaataatataagtttttgattactcccaccttcattaacacttttattagttagaag

tatagttgaaaacggggctggaacaggttgaactgtataccccccattatctgcagctat

tgcccatggtggaggatcagtcgatctagcaattttctctttacatttagctggaatttc

ctcaattttaggagccgtaaattttattacaacagtaattaatatacgatcaacaggaat

tacattcgatcgaatacctttatttgtttgagctgtagttattacagcaattcttctatt

attatctttacctgttttagctggggctattactata-----------------------

----------------------------------------------------------

>SSPAB218-13|Chrysops|COI-5P|KM918726

aacactttactttatttttggggcttgagctggaataattggaacatcattaagtatttt

aattcgtgctgaactaggtcacccaggagctttaattggtgatgatcaaatttataacgt

aattgtaactgctcatgcatttgttataattttctttatagttatacctattataattgg

aggattcggaaattgacttgttccattaatattaggagctcctgatatagcattccctcg

aataaataatataagtttttgattactcccaccttcattaacacttttattagttagaag

tatagttgaaaacggggctggaacaggttgaactgtataccccccattatctgcagctat

tgctcatggtggaggatcagtcgatctagcaattttctctttacatctagctggaatttc

ctcaattttaggagccgtaaattttattacaacagtaattaatatacgatcaacaggaat

tacattcgatcgaatacctttatttgtttgagctgtagttattacagcaattcttctatt

attatctttacctgttttagctggggctattactatattactaacagaccg---------

----------------------------------------------------------

>JWDCI820-11|Chrysops mitis|COI-5P|JN302656

aacactttactttatttttggggcttgagctggaataattggaacatcattaagtatttt

aattcgtgctgaactaggtcacccaggagctttaattggcgatgatcaaatttataacgt

aattgtaactgctcatgcatttgttataattttctttatagttatacctattataattgg

aggattcggaaattgacttgttccattaatattaggagctcctgatatagcattccctcg

aataaataatataagtttttgattactcccaccttcattaacacttttattagttagaag

tatagttgaaaacggggctggaacaggttgaactgtataccccccattatcggcagctat

tgctcatggtggaggatcagtcgatctagcaattttctctttacatttagctggaatttc

ctcaattttaggagccgtaaattttattacaacagtaattaatatacgatcaacaggaat

tacattcgatcgaatacctttatttgtttgagctgtagttattacagcaattcttctatt

attatctttacctgttttagctggggctattactatattactaacagaccgaaatttaaa

tacatctttttttgacccagcaggaggaggtgacccaattttataccaacacttattt

>SSPAB269-13|Chrysops|COI-5P|KM908476

aacactttactttatttttggggcttgagctggaataattggaacatcattaagtatttt

aattcgtgctgaactaggtcacccaggagctttaattggtgatgatcaaatttataacgt

aattgtaactgctcatgcatttgttataattttctttatagttatacctattataattgg

aggattcggaaattgacttgttccattaatattaggagctcctgatatagcattccctcg

aataaataatataagtttttgattactcccaccttcattaacacttttattagttagaag

tatagttgaaaacggggctggaacaggttgaactgtataccccccattatctgcagctat

tgctcatggtggaggatcagtcgatctagcaattttctctttacatctagctggaatttc

ctcaattttaggggccgtaaattttattacaacagtaattaatatacgatcaacaggaat

tacattcgatcgaatacctttatttgtttgagctgtagttattacagcaattcttctatt

attatctttacctgttttagctggggctattactatattactaacagaccga--------

----------------------------------------------------------

>SSPAC11188-13|Chrysops|COI-5P|KM636345

aacactttactttatttttggggcttgagctggaataattggaacatcattaagtatttt

aattcgtgctgaactaggtcacccaggagctttaattggtgatgatcaaatttataacgt

aattgtaactgctcatgcatttgttataattttctttatagttatacctattataattgg

aggattcggaaattgacttgttccattaatattaggagctcctgatatagcattccctcg

aataaataatataagtttttgattactcccaccttcattaacacttttattagttagaag

tatagttgaaaacggggctggaacaggttgaactgtataccccccattatctgcggctat

tgctcatggtggaggatcagtcgatctagcaattttctctttacatctagctggaatttc

ctcaattttaggagccgtaaattttattacaacagtaattaatatacgatcaacaggaat

tacattcgatcgaatacctttatttgtttgagctgtagttattacagcaattcttctatt

attatctttacctg----------------------------------------------

----------------------------------------------------------

>SSPAB275-13|Chrysops|COI-5P|MG473334

-acactttactttatttttggggcttgagctggaataattggaacatcattaagtatttt

aattcgtgctgaactaggtcacccaggagctttaattggtgatgatcaaatttataacgt

aattgtaactgctcatgcatttgttataattttctttatagttatacctattataattgg

gggattcggaaattgacttgttccattaatattaggagcccctgatatagcattccctcg

aataaataatataagtttttgattactcccaccttcattaacacttttattagttagaag

tatagttgaaaacggggctggaacaggttgaactgtataccccccattatctgcagctat

tgcccatagtggaggatcagtcgatctagcaattttctctttacatttagctggaatttc

ctcaattttaggagccgtaaattttattacaacagtaattaatatacgatca--------

------------------------------------------------------------

------------------------------------------------------------

----------------------------------------------------------

>SSPAC596-13|Chrysops|COI-5P|KM643647

-acactttactttatttttggggcttgagctggaataattggaacatcattaagtatttt

aattcgtgctgaactaggtcacccaggagctttaattggcgatgatcaaatttataacgt

aattgtaactgctcatgcatttgttataattttctttatagttatacctattataattgg

aggattcggaaattgacttgttccattaatattaggagcccctgatatagcattccctcg

aataaataatataagtttttgattactcccgccttcattaacacttttattagttagaag

tatagttgaaaacggggctggaacaggttgaactgtataccccccattatcggcggctat

tgctcatggtggaggatcagtcgatctagcaattttctctttacatttagctggaatttc

ctcaattttaggagccgtaaattttattacaacagtaattaatatacgatcaacaggaat

tacattcgatcgaatacctttatttgtttgagctgtagttattacagcaattcttctatt

attatctttacctgttttagctggggctattactatatta--------------------

----------------------------------------------------------

>SSPAB9650-13|Chrysops|COI-5P|KM627847

--------actttatttttggggcttgagcnggaataatnggaacatcattaagtatttt

aattcgtgctgaactaggtcacccaggagctttaattggtgatgatcaaatttataacgt

aattgtaactgctcatgcatttgttataattttctttatagttatacctattataattgg

aggattcggaaattgacttgttccattaatattaggagctcctgatatagcattccctcg

aataaataatataagtttttgattactcccaccttcattaacacttttattagttagaag

tatagttgaaaacggggctggaacaggttgaactgtataccccccattatctgcagctat

tgctcatggtggaggatcagtcgatctagcaattttctctttacatctagctggaatttc

ctcaattttaggagccgtaaattttattacaacagtaattaatatacgatcaacaggaat

tacattcgatcgaatacctttatttgtttgagctgtagttattacagcaattcttctatt

attatctttacctgttttagctggggctatta----------------------------

----------------------------------------------------------

>GMOLF053-19|Chrysops|COI-5P

---actttactttatttttggggcttgagctggaataattggaacatcattaagtatttt

aattcgtgctgaactaggtcacccaggagctttaattggcgatgatcaaatttataacgt

aattgtaactgctcatgcatttgttataattttctttatagttatacctattataattgg

aggattcggaaattgacttgtaccattaatattaggagctcctgatatagcattccctcg

aataaataatataagtttttgattactcccgccttcattaacacttttattagttagaag

tatagttgaaaacggagctggaacaggttgaactgtataccccccattatctgcagctat

tgctcatggtggaggatcagtcgatctagcaattttctctttacatttagctggaatctc

ctcaattttaggagccgtaaattttattacaacagtaattaatatacgatcaacaggaat

tacattcgatcgaatacctttatttgtttgagctgtagttattacagcaattcttctatt

attatctttacctgttttagctggagctattactatattactaacagaccgaaatttaaa

tacatctttttttgacccagcaggaggaggtgacccaattttataccaacacttat--

>MHTAB111-09|Chrysops mitis|COI-5P|KM285506

------------------------------------------------------------

------------------------------------------------------------

---------------------------------------------------------tgg

agnannaggaaattgacttgtnccattaatattaggagctcctgatatagcattccctcg

aataaataatataagtttttgattactcccaccttcattaacacttttattagttagaag

tatagttgaaaacggagctggaacaggttgaactgtataccccccattatctgcagctat

tgcccatggtggaggatcagtcgatctagcaattttctctttacatttagctggaatttc

ctcaattttaggagccgtaaattttattacaacagtaattaatatacgatcaacaggaat

tacattcgatcgaatacctttatttgtttgagctgtagttattacagcaattcttctatt

attatctttacctgttttagctggggctattactatattactaacagaccgaaatttaaa

tacatctttttttgacccagcaggaggaggtgacccaattttataccaacacttattt

>MHTAB112-09|Chrysops mitis|COI-5P|KM285508

------------------------------------------------------------

------------------------------------------------------------

------------------------------------------------------------

-----------------ttgtnccattaatattaggagctcctgatatagcattccctcg

aataaataatataagtttttgattactcccaccttcattaacacttttattagttagaag

tatagttgaaaacggrgctggaacaggttgaactgtataccccccattatctgcagctat

tgcycatggkggaggatcagtcgatctagcaattttctctttacatttagctggaatttc

ctcaattttaggagccgtaaattttattacaacagtaattaatatacgatcaacaggaat

tacattcgatcgaatacctttatttgtttgagctgtaattattacagcaattcttctatt

attatctttacctgttttagctggggctattactatattactaacagaccgaaatttaaa

tacatctttttttgacccagcaggaggaggtgacccaattttataccaacacttattt

>ACT054-07|Chrysops mitis|COI-5P|KM285507

----------------------------------------ggaacatcattaagtatttt

aattcgtgctgaactaggtcacccaggagctttaattggggatgatcaaatttataacgt

aattgtaactgctcatgcatttgttataattttctttatagttatacctattataattgg

agggttcggaaattgacttgttccattaatattaggagctcctgatatagcattccctcg

aataaataatataagtttttgattactcccaccttcattaacacttttattagttagaag

tatagttgaaaatggggctggaacaggttgaactgtataccccccattatctgcagctat

tgcccatggtggaggatcagtcgatctagcaattttctctttacatttagctggaatttc

ctcaattttaggagccgtaaattttattacaacagtaattaatatacgatcaacaggaat

tacattcgatcgaatacctttatttgtttgagctgtagttattacagcaattcttctatt

attatctttacctgttttagctggggctattactatattactaacagaccgaaatttaaa

tacatctttttttgacccagcaggaggaggtgacccaattttataccaacacttattt

>TTDBW261-09|Chrysops|COI-5P|HQ981269

--------------------------------------ttggaacatcattaagtatttt

aattcgtgctgaactaggtcacccaggagctttaattggtgatgatcaaatttataacgt

aattgtaactgctcatgcatttgttataattttctttatagttatacctattataattgg

agggttcggaaattgacttgttccattaatattaggagctcctgatatagcattccctcg

cataaataatataagtttttgattactcccaccttcattaacacttttattagttagaag

tatagttgaaaacggggctggaacaggttgaactgtataccccccattatctgcagctat

tgctcatggtggaggatcagtcgatctagcaattttctctttacatttagctggaatttc

ctcaattttaggggccgtaaattttattacaacagtaattaatatacgatcaacaggaat

tacattcgatcgaatacctttatttgtttgagctgtagttattacagcaattcttctact

attatctttacctgttttagctggggctattactatattactaacagaccgaaatttaaa

tacatctttttttgacccagcaggaggaggtgacccaattttataccaacacttattt

>SSKJC617-15|Chrysops|COI-5P|MF837231

----------------------------------------------------------tt

aattcgtgctgaactaggtcatccaggagctttaattggtgatgatcaaatttataacgt

aattgtaactgctcatgcatttattataattttctttatagttatacctattataattgg

gngattcggaaattgacttgtaccattaatattaggagctcctgatatagcattccctcg

aataaataatataagtttttgattactccctccttcattaacacttttattagttagaag

tatagttgaaaacggagctggaacaggttgaactgtataccccccattatctgcagctat

tgcccatggcggaggatcagtcgatctagcaattttctctttacatttagctggaatttc

ctcaattttaggggccgtaaattttattacaacagtaattaatatacgatcaacaggaat

tacattcgatcgaatacctttattcgtttgagctgtagttattacagcaattcttctatt

attatctttacctgttttagctggagctattactatattattaacagaccgaaatttaaa

tacatctttt------------------------------------------------

>SSPAA3283-13|Chrysops|COI-5P|MG475848

------------------------------------------------------------

------------------------------------------------------------

--------------------------------------tagttatacctattataaatgg

aggatttggaaattgacttgtaccattaatattaggaggtcctgatatagcattccctcg

aataaataatataagtttttgattactccctccttcattaacacttttattagttagaag

tatagttgaaaatggagntggaacaggatgaactgtatatcccccattatttgcagntat

tgcccatggtggaggatcagtcgatctagcaattttctctttacatttagctggaatttc

ctcaattttaggtgccgtaaattttattacaacagtaattaatatacgatcaacaggaat

tacattcgatcgaatacctttatttgtttgagctgtagttattacagcaattcttctatt

attatctttacctgttttagccgggg----------------------------------

----------------------------------------------------------

>SSPAA3332-13|Chrysops|COI-5P|KM910412

------------------------------------------------------------

-------------------------ggagctttaattggtgatgatcaaatttataacgt

aattgtaactgctcatgcatttattataattttctttatagttatacctattataattgg

aggattcggaaattgacttgtaccattaatattaggagctcctgatatagcatttcctcg

aataaataatataagtttttgattactccctccttcattaacacttttattagttagaag

tatagttgaaaacggagctgggacaggttgaactgtataccccccattatctgcagctat

tgcccatggcggaggatcagtcgatctagcaattttctctttacatttagctggaatttc

ctcaattttaggagccgtaaattttattacaacagtaattaatatacgatcaacaggaat

tacattcgatcgaatacctttatttgtttgagctgtagttattacagcaattcttctatt

attatctttacctgttttagctggagctattactatattattaacagaccgaaatttaaa

tacatctttttttgacccagcaggaggaggt---------------------------

>CNNHE1715-14|Chrysops|COI-5P|KR505638

-------------atttttggggcttgagctggaataattggaacatcattaagtatttt

aattcgtgctgaactaggtcacccaggagctttaattggggatgatcaaatttataacgt

aattgtaactgctcatgcatttgttataattttctttatagttatacctattataattgg

gggattcggaaattgacttgttccattaatattaggagctcctgatatagcattccctcg

aataaataatataagtttttgattactcccaccttcattaacacttttattagttagaag

tatagttgaaaacggggctggaacaggttgaactgtataccccccattatctgcagctat

tgcccatggtggaggatcagtcgatctagcaattttctctttacatttagctggaatttc

ctcaattttaggggccgtaaattttattacaacagtaattaatatacgatnn---ngaat

tacattcgatcgaatacctttatttgtttgagctgtagttattacagcaattcttctatt

attatctttacctgttttagctggggntattactatattactaaca--------------

----------------------------------------------------------

>CNPKF2751-14|Chrysops|COI-5P|MG474333

aacactttactttatttttggggcttgagctggaataattggaacatcattaagtatttt

aattcgtgctgaactaggtcacccaggagctttaattggtgatgatcaaatctataacgt

aattgtaactgctcatgcatttgttataattttctttatagttatacctattataattgg

gggattcggaaattgacttgtaccattaatattaggagcccctgatatagcattccctcg

aataaataatataagtttttgattactcccaccttcattaacacttttattagttagaag

tatagttgaaaacggagctggaacaggttgaactgtataccccccattatctgcagctat

tgctcatggtggaggatcagtcgatctagcaattttctctttacatttagctggaatctc

ctcaattttaggagccgtaaattttattacaacagtaattaatatacgat----------

------------------------------------------------------------

------------------------------------------------------------

----------------------------------------------------------

>ASDIP742-15|Chrysops excitans|COI-5P

aacactttactttatttttggggcttgagctggaataattggaacatcattaagtatttt

aattcgtgctgaactaggtcacccaggagctttaattggtgatgaccaaatttataatgt

aattgtaactgctcatgcatttattataattttctttatagttatacctattataattgg

aggattcggaaattgacttgtaccattaatattaggagctcctgatatagcattccctcg

aataaataatataagtttttgattactccctccttcattaacacttttattagttagaag

tatagttgaaaatggagctggaacaggatgaactgtataccccccattatctgcagctat

tgcccatggtggaggatcagtcgatttagcaattttctctttacatttagctggaatttc

ctcaattttaggtgccgtaaattttattacaacagtaattaatatacgatcaacaggaat

tacattcgatcgaatacctttatttgtttgagctggagttattacagcaattcttcctat

tatatctttacctgttttagccggggctattactat------------------------

----------------------------------------------------------

>MOBIL1431-16|Chrysops|COI-5P

------------------------------------------------------------

------------------------------------------------------------

----------------------------------------------------------gg

aggattcggaaattgacttgtaccattaatattaggagctcctgatatagcattccctcg

aataaataatataagtttttgattactcccgccttcattaacacttttattagttagaag

tatagttgaaaacggagctggaacaggttgaacngtataccccccattatctgcagctat

tgctcatggtggaggatcagtcgatctagcaattttctctttacatttagctggaatctc

ctcaattttaggagccgtaaattttattacaacagtaattaatatacgatc---------

------------------------------------------------------------

------------------------------------------------------------

----------------------------------------------------------

>SSPAA108-13|Chrysops|COI-5P|KM907073

aacactttactttattttttgggcttgagctggaataattggaacatcattaagtatttt

aattcgtgctgaactaggccacccaggcgctttaattggtgatgatcaaatttataacgt

aattgtaactgctcangcatttattataattttctttatagttatacctattataattgg

aggattgggaaattgacttgtaccattaatattaggagctcccgatatagcatttcctcg

aataaataatataagtttttgattactccctccttcattaacacttttattagttagaag

tatagttgaaaacggagctgggacaggttgaactgtataccccccattatctgcagctat

tgcccatggcggaggatcagtcgatctagcaattttctctttacatttagctggaatttc

ttcaattttaggagccgtaaattttattacaacagtaattaatatacgatcaacaggaat

tacattcgatcgaatacctttatttgtttgagctgtagttattacagcaattcttctatt

attatctttacctgttttagctg-------------------------------------

----------------------------------------------------------

>BBDCP620-10|Chrysops|COI-5P|JF868982

aacactttactttatttttggggcttgggctggaataattggaacatcactaagtatttt

aattcgtgctgaactaggtcacccaggagctttaattggtgatgatcaaatttataatgt

aattgtaactgctcatgcattcgttataattttctttatagttatacctattataattgg

aggattcggaaattgacttgtaccactaatattaggagcacctgatatagcattccctcg

aataaataatataagtttttgattacttcctccttcattaacacttttattagttagaag

catagttgaaaacggagctggaactggatgaacggtatatcctccattatctgcagcaat

tgcccatggagggggatcagttgatctggcaattttttctttacatttagcaggaatctc

atcaattttaggagctgtaaattttatcacaacagtaattaatatacgatcaacaggaat

tacattcgatcgaatacctttatttgtttgagctgtagttattacagcaattcttctttt

attatctttacctgttttagctggagctattactatattactaacagaccgaaatttaaa

tacatctttctttgatccagcaggaggaggagatccaattttataccaacacttattt

>BBDCP619-10|Chrysops|COI-5P|JF868981

aacactttactttatttttggggcttgggctggaataattggaacatcactaagtatttt

aattcgtgctgaactaggccacccaggagctttaattggtgatgatcaaatttataatgt

aattgtaactgctcatgcattcgttataattttctttatagttatacctattataattgg

aggattcggaaattgacttgtaccactaatattaggagcacctgatatagcattccctcg

aataaataatataagtttttgattacttcctccttcattaacacttttattagttagaag

catagttgaaaacggagctggaactggatgaacggtatatcctccattatctgcagcaat

tgcccatggagggggatcagttgatctggcaattttttctttacatttagcaggaatctc

atcaattttaggagctgtaaattttatcacaacagtaattaatatacgatcaacaggaat

tacattcgatcgaatacctttatttgtttgagctgtagttattacagcaattcttctttt

attatctttacctgttttagctggagctattactatattactaacagaccgaaatttaaa

tacatctttctttgatccagcaggaggaggagatccaattttataccaacacttattt

>MHTAB114-09|Chrysops cincticornis|COI-5P|KM285446

----------------------------------------ggaacatcattaagtatttt

aattcgtgctgaattaggtcatccaggtgctttaattggtgatgatcaaatttataatgt

aattgtaactgctcatgcatttgttataattttctttatagttatacctattataattgg

gggattcggaaattgacttgttccattaatgttaggagctcctgacatagcattccctcg

aataaataatataagtttttgactacttcctccttcattaacacttttgttagttagaag

tatagttgaaaatggggctggaactggttgaactgtatacccaccgttatctgcagctat

tgctcatggaggaggatcagttgatttagcaattttttctttacatttagctggaatttc

atcaattctaggagctgtaaattttattacaacagtaattaatatacgatcaacaggaat

tacatttgatcgaatacccttatttgtctgagctgtagttattacagcaattcttctttt

attatctctacctgttttagcaggagccattactatattactaacagatcgaaatctaaa

tacatctttctttgatccagcaggaggaggtgacccaattttataccaacatttattt

>OPPEE392-17|Chrysops cincticornis|COI-5P

aacactttactttatttttggggcttgggctggaataattggaacatcattaagtatttt

aattcgtgctgaattaggtcatccaggtgctttaattggtgatgatcaaatttataatgt

aattgtaactgctcatgcatttgttataattttctttatagttatacctattataattgg

gggattcggaaattgacttgttccattaatgttaggagctcctgacatagcattccctcg

aataaataatataagtttttgactacttcctccttcattaacacttttattagttagaag

tatagttgaaaatggggctggaactggttgaactgtatacccaccgttatctgcagctat

tgctcatggaggaggatcagttgatttagcaattttttctctacatttagctggaatttc

atcaattctaggagctgtaaattttattacaacagtaattaatatacgatcaacaggaat

tacatttgatcgaatacccttatttgtctgagctgtagttattacagcaattcttctttt

attatctctacctgttttagcaggagccattactatattactaacagatcgaaatctaaa

tacatctttctttgatccagcaggaggaggtgatccaattttatatcaacatttattt

>OPPEQ219-17|Chrysops cincticornis|COI-5P

aacactttactttatttttggggcttgagctggaataattggaacatcattaagtatttt

aattcgtgctgaattaggtcatccaggtgctttaattggtgatgatcaaatttataatgt

aattgtaactgctcatgcatttgttataattttctttatagttatacctattataattgg

gggattcggaaattgacttgttccattaatgttaggagctcctgacatagcattccctcg

aataaataatataagtttttgactacttcccccttcattaacacttttattagttagaag

tatagttgaaaatggagctggaactggttgaactgtatacccaccgttatctgcagctat

tgctcatggaggaggatcagttgatttagcaattttttctttacatttagctggaatttc

atcaattctaggagctgtaaattttattacaacagtaattaatatacgatcaacaggaat

tacatttgatcgaatacccttatttgtctgagctgtagttattacagcaattcttctttt

attatctctacctgttttagcaggagccattactatattactaacagatcgaaatctaaa

tacatctttctttgatccagcaggaggaggtgatccaattttatatcaacatttattt

>OPPOC092-17|Chrysops cincticornis|COI-5P

aacactttactttatttttggggcttgagctggaataattggaacatcattaagtatttt

aattcgtgctgaattaggtcatccaggtgctttaattggtgacgatcaaatttataatgt

aattgtaactgctcatgcatttgttataattttctttatagttatacctattataattgg

gggattcggaaattgacttgttccattaatgttaggagctcctgacatagcattccctcg

aataaataatataagtttttgactacttcctccttcattaacacttttgttagttagaag

tatagttgaaaatggggctggaactggttgaactgtatacccaccgttatctgcagctat

tgctcatggaggaggatcagttgatttagcaattttttctttacatttagctggaatttc

atcaattctaggagctgtaaattttattacaacagtaattaatatacgatcaacaggaat

tacatttgatcgaatacccttatttgtctgagctgtagttattacagcaattcttctttt

attatctctacctgttttagcaggagccattactatattactaacagatcgaaatctaaa

tacatctttctttgatccagcaggaggaggtgacccaattttataccaacatttattt

>ASDIP735-15|Chrysops cincticornis|COI-5P

aacactttactttatttttggggcttgagctggaataattggaacatcattaagtatttt

aattcgtgctgaattaggtcatccaggtgctttaattggtgatgatcaaatttataatgt

aattgtaactgctcatgcatttgttataattttctttatagttatacctattataattgg

gggattcggaaattgacttgttccattaatgttaggagctcctgacatagcattccctcg

aataaataatataagtttttgactacttcctccttcattaacacttttgttagttagaag

tatagttgaaaatggggctggaactggttgaactgtatacccaccgttatctgcagctat

tgctcatggaggaggatcagttgatttagcaattttttctttacatttagctggaatttc

atcaattctaggagctgtaaattttattacaacagtaattaatatacgatcaacaggaat

tacatttgatcgaatacccttatttgtctgagctgtagttattacagcaattcttctttt

attatctctacctgttttagcaggagccattactatattactaacagatcgaaatctaaa

tacatctttctttgatccagcaggaggaggtgaccca---------------------

>RRSSA228-15|Chrysops cincticornis|COI-5P|MG168005

----------tttatttttggggcttgagctggaataattggaacatcattaagtatttt

aattcgtgctgaattaggtcatccaggtgctttaattggtgatgatcaaatttataatgt

aattgtaactgctcatgcatttgttataattttctttatagttatacctattataattgg

gggattcggaaattgacttgttccattaatgttaggagctcctgacatagcattccctcg

aataaataatataagtttttgactacttcctccttcattaacacttttattagttagaag

tatagttgaaaatggggctggaactggttgaactgtatacccaccattatctgcagctat

tgctcatggaggaggatcagttgatttagcaattttttctctacatttagctggaatttc

atcaattctaggagctgtaaattttattacaacagtaattaatatacgatcaacaggaat

tacatttgatcgaatacccttatttgtctgagctgtagttattacagcaattcttctttt

attatctctacctgttttagcaggagccattactatattacta-----------------

----------------------------------------------------------

>CROBB287_SK-3_Chrysops parallelogrammus

aacactttactttattttcggagcttgagccggaataattggaacatcattaagtatttt

aattcgagctgaactagggcatccaggagccttaattggtgatgatcaaatttataatgt

aattgtaactgctcatgcatttgttataattttctttatagttatacctattataattgg

tggatttgggaattgattggtaccattaatattaggagcccctgatatagcctttcctcg

aataaataatataagtttttgattactacctccttcattaacccttttattagttagcag

tatagttgaaaatggggctggaacaggttgaactgtttaccctccattatcagcagctat

tgctcatggaggtggatctgtagatttagcaattttttcattacatttagccggaatttc

ttcaattttaggggctgtaaattttattactacagtaattaatatacgatcaacaggaat

tacatttgatcgaatacctttatttgtatgagctgtagtaattacagctattcttctttt

attatcattacctgtcttagcaggagctattactatactattaactgatcgaaatttaaa

tacttcattctttgatccagccggaggaggtgacccaattttataccaacacttattt

>GMMDL224-15|Chrysops madagascarensis|COI-5P

aactttatactttatttttggtgcttgagccggaataattggaacatcattaagtatttt

aattcgagctgaattaggtcatccaggagctttaattggtgatgatcaaatttataatgt

aattgtaactgctcatgcatttgttataattttctttatagttatacctattataattgg

aggatttggaaattgattagttcctttaatattaggagctcctgatatagcatttccccg

aataaataatataagtttttgattattacctccttcattaacattattattagttagtag

aatagtagaaaatggagctggaacaggatgaactgtttatccaccactatcagcagctat

tgctcatggaggaggatcagttgatttagctattttctctcttcatttagctggaatctc

ttcaattttaggagctgtaaattttattactacagtaattaatatacgatcaactggaat

tacatttgatcgaataccattatttgtttgagctgtagtaattacagcaattcttctttt

attatctttacctgttttagctggtgctattactatattattaacagatcgaaatttaaa

tacatcattttttgatccagcaggagg-------------------------------

>GMMDA071-15|Chrysops madagascarensis|COI-5P

----------tttatttttggtgcttgagccggaataattggaacatcattaagtatttt

aattcgagctgaattaggtcatccaggagctttaattggtgatgatcaaatttataatgt

aattgtaactgctcatgcatttgttataattttctttatagttatacctattataattgg

aggatttggtaattgattagttcctttaatattaggagctcctgatatagcatttcctcg

aataaataatataagtttttgattattacctccttcattaacattattattagttagtag

aatagtagaaaatggagctggaacaggatgaactgtatacccaccactatcagcagctat

tgctcatggaggaggatcagttgatttagctattttttctcttcatttagctggaatctc

ttcaattttaggtgctgtaaattttattactacagtaattaatatacgatcaactggaat

tacatttgatcgaataccattatttgtctgagctgtagtaattacagcaattcttctttt

attatctttacctgttttagctggtgctattactatattattaacagat-----------

----------------------------------------------------------

>GMMDF172-15|Chrysops madagascarensis|COI-5P

-actttatactttatttttggtgcttgagccggaataattggaacatcattaagtatttt

aattcgagctgaattaggtcatccaggagctttaattggtgatgatcaaatttataatgt

aattgtaacagctcatgcatttgttataattttctttatagttatacctattataattgg

aggatttggaaattgattagttcctttaatattaggagctcctgatatagcatttcctcg

aataaataatataagtttttgattattacctccttcattaacattattattagttagtag

aatagtagaaaatggagctggaacaggatgaactgtatacccacccctatcagcagctat

tgctcatggaggaggatcagttgatttagcaattttttctcttcatttagctggaatttc

ttcaattttaggtgctgtaaattttattactacagtaattaatatgcgatcaactggaat

tacatttgatcgaataccattatttgtttgagctgtagtaattacagcaattcttctttt

attatctttacctgttttagctggtgctattactatattattaacagatcga--------

----------------------------------------------------------

>ASIND3651-12|Chrysops variegatus|COI-5P

aactctatattttattttcggggcttgagccggaataattggaacatctttaagtatttt

aattcgtgctgaactaggtcacccaggagcattaattggggatgatcaaatttataatgt

aattgtaacagctcatgcatttgttataattttctttatagtaatacctattataattgg

aggatttggtaattgattagttcctttaatattaggagcacctgatatagcattccctcg

aataaataatataagtttttgattattacctccctcattaacactattattagttagaag

tatagttgaaaatggagctggaacaggttgaactgtttacccaccattatctgctgctat

tgcacatggtggaggatcagttgatttagcaattttttctttacacttagctggaatttc

atcaattttaggagctgtaaattttattacaacagtaattaatatacgatcaacaggtat

tacatttgatcgaatacccttatttgtttgagcagtagttattactgctattcttctttt

attatcattacctgtcttagcaggagctattactatattattaactgatcgaaatttaaa

tacttctttctttgatccagcaggaggtggagatccaattttataccaacatttattt

>ASIND3649-12|Chrysops variegatus|COI-5P

aaccctatattttattttcggggcttgagccggaataattggaacatctttaagtatttt

aattcgtgctgaactaggtcacccaggggcattaattggggatgatcaaatttataatgt

aattgtaacagctcatgcatttgttataattttctttatagtaatacctattataattgg

aggatttggtaattgattagttcctttaatattaggagcacctgatatagcattccctcg

aataaataatataagtttttgattattacctccttcattaacactattattagttagaag

tatagttgaaaatggagctggaacaggttgaactgtttacccaccattatctgctgctat

tgcacatggtggaggatcagttgatttagcaattttttctttacacttagctggaatttc

atcaattttaggggctgtaaattttattacaacagtaattaatatacgatcaacaggtat

tacatttgatcgaatacccttatttgtttgagcagtagttattactgctattcttctttt

attatcattacctgtcttagcaggagctattactatattattaactgatcgaaatttaaa

tacttctttctttgatccagcaggaggtggagatccaattttataccaacatttattt

>ASIND3658-12|Chrysops variegatus|COI-5P

aaccctatattttattttcggggcttgagccggaataattggaacatctttaagtatttt

aattcgtgctgaactaggtcacccaggggcattaattggggatgatcaaatttataatgt

aattgtaacagctcatgcatttgttataattttctttatagtaatacctattataattgg

aggatttggtaattgattagttcctttaatattaggagcacctgatatagcattccctcg

aataaataatataagtttttgattattacctccttcattaacactattattagttagaag

tatagttgaaaatggagctggaacaggttgaactgtttacccaccattatctgctgctat

tgcacatggtggaggatcagttgatttagcaattttttctttacacttagctggaatttc

atcaattttaggggctgtaaattttattacaacagtaattaatatacgatcaacaggtat

tacatttgatcgaatacccttatttgtttgagcagtagttattactgctattcttctttt

attatcattacctgtcttagcaggagctattactatattattaactgatcgaaatttaaa

tacttctttctttgatccagcaggaggtggagatcca---------------------

>ASIND3655-12|Chrysops variegatus|COI-5P

aactctatattttattttcggggcttgagccggaataattggaacatctttaagtatttt

aattcgtgctgaactaggtcacccaggggcattaattggggatgatcaaatttataatgt

aattgtaacagctcatgcatttgttataattttctttatagtaatacctattataattgg

aggatttggtaattgattagttcctttaatattaggagcacctgatatagcattccctcg

aataaataatataagtttttgattattacctccttcattaacactattattagttagaag

tatagttgaaaatggagctggaacaggttgaactgtttacccaccattatctgctgctat

tgcacatggnggaggatcagttgatttagcaattttttctttacacttagctggaatttc

atcaattttaggggctgtaaattttattacaacagtaattaatatacgatcaacaggtat

tacatttgatcgaatacccttatttgtttgagcagtagttattactgctattcttctttt

attatcattacctgtcttagcaggagctattactatattattaactgatcgaaatttaaa

tacttctttctttga-------------------------------------------

>GMCWW074-14|Chrysops|COI-5P

-accctatattttattttcggggcttgagccggaataattggaacatctttaagtatttt

aattcgtgctgaactaggtcacccaggggcattaattggggatgatcaaatttataatgt

aattgtaacagctcatgcatttgttataattttctttatagtaatacctattataattgg

aggatttggtaattgattagttcctttaatattaggagcacctgatatagcattccctcg

aataaataatataagtttttgattattacctccttcattaacactattattagttagaag

tatagttgaaaatggagctggaacaggttgaactgtttacccaccattatctgctgctat

tgcacatggtggaggatcagttgatttagcaattttttctttacacttagctggaatttc

atcaattttaggggctgtaaattttattacaacagtaattaatatacgatcaacaggtat

tacatttgatcgaatacccttatttgtttgagcagtagttattactgctattcttctttt

attatcattacctgtcttagcaggagctattactatattattaactgatcgaaat-----

----------------------------------------------------------

>GBDP15882-15|Chrysops sp. 20 Peru|COI-5P|KM243517

--------------tttttggagcttgagccggaataattggaacttcattaagaatttt

aattcgagctgaacttggtcacccaggagccttaattggtgatgaccaaatttataatgt

aattgtaactgctcatgcatttgtaataattttctttatagttatacctattataattgg

aggatttggaaattgattagttcctttaatactaggagcccctgatatagcattcccacg

aataaataatataagtttttgattattacccccttcattaactcttttattagtaagtag

tatagttgaaaatggggctggaactggatgaacagtatacccaccattatcagccgctat

tgctcatggaggaggctcagttgacttagcaattttctctttacatttagctggaatttc

ttcaattttaggagctgtaaattttattacaacagttattaatatacgatctacaggaat

tacatttgaccgaatacctttatttgtctgagctgttgtaattactgcaattcttctttt

attatcattacctgtattagctggagctattactatattattaactgatcgaaacttaaa

tacttcattttttgatccggcaggaggcggagacccaattttata-------------

>GBDP25067-19|Chrysops silvifacies|COI-5P|KT225292

aactctttactttatttttggagcttgagccggaataattggaacatctttaagtatttt

aattcgtgctgaactaggtcatcctggagctttaattggtgatgatcaaatttataatgt

aattgtaactgctcatgcatttattataattttttttatagttatacctattataattgg

aggattcggaaattgactagtcccattaatactcggtgcacctgatatagctttccctcg

aataaataacataagtttttgaatacttcctccttcattgactcttttattagttagaag

aatagttgaaaacggggctggaacaggttgaactgtttatcctccgttatcagcatctat

tgctcatagtgggggatctgtcgatttagcaattttttctttacatttagctggaatttc

ttctattttaggagctgtaaattttattactacaattattaatatacgatcaactggaat

tacgtttgatcgaatacctttatttgtttgagctgtaataattacagctattcttctttt

attatctcttccagttttagccggagctattacaatattattaactgatcgaaatttaaa

tacatcattttttgacccagcaggaggaggtgatccaattttatatcaacatttattt

>GBMNB24918-20|Chrysops pellucidus|COI-5P|MT116984

----------------------------------ataattggaacttcattaagtatttt

aattcgagctgaattaggtcatccaggagctttaattggagatgaccaaatttataatgt

aattgttactgctcatgcatttgtaataattttctttatagttataccaattataattgg

aggatttggaaattgattagtaccattaatattaggagcccctgatatagcttttcctcg

aataaataatatgagtttttgacttttacccccttcattaactttactattagtaagtag

tatagttgaaaatggagctggaacaggatgaactgtttatcccccattatctgctgcaat

tgcccatggaggaggatcagttgatttagctattttttctttacatttagctggaatttc

ttcaattttaggagctgtaaattttattactacagtaattaatatacgatcaactggaat

tacatttgatcgaataccactatttgtttgagcagtagttattacagctattcttctttt

attatcactccctgttttagcaggagctattactatattattaactgatcgaaatttaaa

tacatcattttttgatcctgctggaggaggagaccctattttatatcaacatttattt

>GBMNA24634-19|Chrysops silaceus|COI-5P|MK396277

-------tattttattttcggagcatgagctggaataattggaacttcattaagtatttt

aattcgagctgaattaggtcatccaggagctttaattggagatgaccaaatttataatgt

aattgtaactgcccatgcatttgttataattttctttatagttatacccattataattgg

aggatttggtaattgattagttccattaatattaggagctcctgatatagcatttcctcg

aataaataatataagtttttgactattacctccctcattgacattattattagttagaag

tatagtcgaaaatggagctggaactggatgaactgtatatccccctctatcagcagctat

tgcccatggtggaggttcagttgatttagcaattttttctttacatctagctggaatttc

atcaattttaggagctgtaaattttattacaacagttattaatatacgatcaactggaat

tacatttgatcgaataccattatttgtttgatctgtagtaattacagcaattcttctttt

attatctcttcctgtattagcaggagctattactatattactaactgaccgaaatttaaa

tacatcattttttgatcctgctggaggaggagatccaattttataccaacatttattt

>GBMNA24624-19|Chrysops silaceus|COI-5P|MK396276

-------tattttattttcggagcatgagctggaataattggaacttcattaagtatttt

aattcgagctgaattaggtcatccaggagctttaattggagatgatcaaatttataatgt

aattgtaactgcccatgcatttgttataattttctttatagttatacccattataattgg

aggatttggtaattgattagttccattaatattaggagctcctgatatagcatttcctcg

aataaataatataagtttttgattattacctccctcattgacattattattagttagaag

tatagtcgaaaatggagctggaactggatgaactgtatatccccctctatcagcagctat

tgcccatggtggaggttcagttgatttagcaattttttctttacatctagctggaatttc

atcaattttaggagctgtaaattttattacaacagttattaatatacgatcaactggaat

tacatttgatcgaataccattatttgtttgatctgtagtaattacagcaattcttctttt

attatctcttcctgtattagcaggagctattactatattactaactgaccgaaatttaaa

tacatcattttttgatcctgctggaggaggagatccaattttatatcaacatttattt

>GBMNA24705-19|Chrysops silaceus|COI-5P|MK396278

-------tattttattttcggagcatgagctggaataattggaacttcattaagtatttt

aattcgagctgaattaggtcatccaggagctttaattggagatgaccaaatttataatgt

aattgtaactgcccatgcatttgttataattttctttatagttatacccattataattgg

aggatttggtaattgattagttccattaatattaggagctcctgatatagcatttcctcg

aataaataatataagtttttgattattacctccctcattaacattattattagttagaag

tatagtcgaaaatggagctggaactggatgaactgtatatccccctctatcagcagctat

tgcccatggtggaggttcagttgatttagcaattttttctttacatctagctggaatttc

atcaattttaggagctgtaaattttattacaacagttattaatatacgatcaactggaat

tacatttgatcgaataccattatttgtttgatctgtagtaattacagcaattcttctttt

attatctcttcctgtattagcaggagctattactatgttactaactgaccgaaatttaaa

tacatcattttttgatcctgctggaggaggagatccaattttatatcaacatttattt

>GBMNA24669-19|Chrysops dimidiatus|COI-5P|MK396265

-------tattttattttcggagcatgagctggaataattggaacttcactaagtatttt

aattcgagctgaattaggtcatccaggagctttaattggagatgaccaaatttataatgt

aattgtaactgctcatgcatttgttataattttctttatagtaatacctattataattgg

aggatttggaaattgacttgtaccattaatattaggagcccctgatatagcatttcctcg

aataaataatataagtttttgacttttacctccttcattaacattattattagtgagaag

tatagtagaaaatggagctggaactggatgaactgtttatcctcctctttcagcagctat

tgctcatggtggtggttcagttgatttagcaattttttctttacatttagctggaatttc

atcaattttaggagctgttaattttattacaacagtaattaatatacgatcaactggtat

tacatttgatcgaataccattatttgtttgagctgtagtaattacagcaattttactttt

attatctcttcctgtattagcaggagctattactatattattaactgatcgaaatttaaa

tacatcattttttgatcctgctggaggaggagacccaatcttatatcaacatttattt

>GBMNA24637-19|Chrysops longicornis|COI-5P|MK396271

-------tattttattttcggagcttgagccggaataattggaacatctttaagaatttt

aattcgtgctgaattaggtcatccaggagccttaattggagatgaccaaatttataatgt

tattgttactgctcatgcatttattataattttctttatagtaatacctattataattgg

aggatttggaaattgacttgtaccattaatattaggagctcctgatatagcatttcctcg

aataaataatataagtttttgacttcttcccccatcattaactttactattagtaagtag

tatagttgaaaatggagctggaacaggatgaactgtctatccacccttatcagcagcaat

tgctcatggaggaggatcagttgatttagctattttctcattacatttagctggaatttc

atcaattttaggagctgtaaattttattactacagttattaatatgcgatcaactggaat

tacatttgatcgaataccattatttgtttgagctgtcgttattactgctattcttctttt

attatctttaccagttttagctggagctattacaatattattaacagatcgaaatttaaa

tacttcattctttgacccagctggaggaggtgatcctattttataccaacatttattt

>GBMNA24638-19|Chrysops longicornis|COI-5P|MK396272

-------tattttattttcggagcttgagccggaataattggaacatctttaagaatttt

aattcgtgctgaattaggtcatccaggagccttaattggagatgaccaaatttataatgt

tattgttactgctcatgcatttattataattttctttatagtaatacctattataattgg

aggatttggaaattgacttgtaccattaatattaggagctcctgatatagcatttcctcg

aataaataatataagtttttgacttcttcccccatcattaactttactattagtaagtag

tatagttgaaaatggggctggaacaggatgaactgtctatccacccttatcagcagcaat

tgctcatggaggaggatcagttgatttagctattttctcattacatttagctggaatttc

atcaattttaggagctgtaaattttattactacagttattaatatgcgatcaactggaat

tacatttgatcgaataccattatttgtttgagctgtcgttattactgctattcttctttt

attatctttaccagttttagctggagctattacaatattattaacagatcgaaatttaaa

tacttcattctttgacccagctggaggaggtgatcctattttatatcaacatttattt

>GBMNA24702-19|Chrysops longicornis|COI-5P|MK396275

-------tattttattttcggagcttgagccggaataattggaacatctttaagaatttt

aattcgtgctgaattaggtcatccaggagccttaattggagatgaccaaatttataatgt

tattgttactgctcatgcatttattataattttctttatagtaatacctattataattgg

aggatttggaaattgacttgtaccattaatattaggagctcctgatatagcttttcctcg

aataaataatataagtttttgacttcttcctccatcattaactttactattagtaagtag

tatagttgaaaatggagctggaacaggatgaactgtttatccacccttatcagcagcaat

tgctcatggaggaggatcagttgatttagctattttctcattacatttagctggaatttc

atcaattttaggagctgtaaattttattactacagttattaatatacgatcaactggaat

tacatttgatcgaataccattatttgtttgagctgtcgttattactgctattctcctttt

attatctttaccagttttagctggagctattacaatattattaacagatcgaaatttaaa

tacttcattctttgatccagccggaggaggtgatcctattctataccaacatttattt

>GBMNA24706-19|Chrysops longicornis|COI-5P|MK396270

-------tattttattttcggagcttgagccggaataattggaacatctttaagaatttt

aattcgtgctgaattagggcatccaggagccttaattggtgatgatcaaatttataatgt

tattgttactgctcatgcatttattataattttctttatagtaatacctattataattgg

aggatttggaaattgacttgtaccattaatattaggagcccctgatatagcatttcctcg

aataaataatataagtttttgacttcttcctccatcactaactttactattagtaagtag

tataattgaaaacggagctggaacaggatgaactgtttatccacccttatcagcagcaat

tgctcatggaggaggatcagttgatttagctattttttcattacatttagctggaatttc

atcaattttaggagctgtaaattttattactacagttattaatatacgatcaactggaat

tacatttgatcgaataccattatttgtttgagctgtagttattactgctattcttctttt

attatctttacccgttttagctggagctattacaatattattaacagatcgaaatttaaa

tacttctttctttgatccagctggaggaggtgaccctattttatatcaacacttattt

>GBMNC485-20|Chrysops distinctipennis|COI-5P|MW013791

aactctatactttattttcggggcttgggccggaataattggaacatccttaagaattct

aattcgtgcagaattaggccatccaggagccttaattggagatgaccaaatttataatgt

tattgttactgctcatgcatttgttataattttctttatagtaatacctattataattgg

tggatttggaaattggcttgtaccattaatattaggagcccctgatatagcattccctcg

aataaataatataagtttttggctacttcctccatcgttaaccctattattagtaagtag

aatagttgaaaacggagctggaacaggatggactgtatacccaccactatcagcagcaat

tgctcatggagggggatcagttgatttagctattttttcacttcatttagcaggaatttc

atcaattctaggggctgtaaattttattacaacagttattaatatacgatcaactggaat

tacatttgaccgaatacccttatttgtttgggcagtagttattactgctattcttcttct

tttatctttacctgttttagctggagctattacaatattattaactgatcgaaacttaaa

tacttcattttttgacccagcaggaggaggtgaccctattttataccaacatttatt-

>GBMNC487-20|Chrysops distinctipennis|COI-5P|MW013789

aactctatactttattttcggagcttgggccggaataattggaacatccttaagaattct

aattcgtgcagaattaggtcatccaggagccttaattggagatgatcaaatttataatgt

tattgttactgctcatgcatttgttataattttctttatagtaatacctattataattgg

tggatttggaaattggcttgtaccattaatattaggagcccctgatatagcattccctcg

aataaataatataagtttttggctactccctccatcattaaccctattattagtaagtag

aatagttgaaaatggagctggaacaggatggactgtatacccaccactatcagcagcaat

tgctcatggagggggatcagttgatttagctattttttcacttcatttagcaggaatttc

atcaattctaggagctgtaaattttattacaacagttattaatatacgatcaactggaat

tacatttgaccgaatacctttatttgtttgggcagtagttattactgctattcttcttct

tttatctttacctgttttagctggagctattacaatattattaactgatcgaaacttaaa

tacttcattttttgacccagcaggaggaggtgaccctattttatatcaacatttatt-

>GBMNC488-20|Chrysops distinctipennis|COI-5P|MW013788

aactctatactttattttcggagcttgggccggaataattggaacatccttaagaattct

aattcgtgcagaattaggtcatccaggagccttaattggagatgaccaaatttataatgt

tattgttactgctcatgcatttgttataattttctttatagtaatacctattataattgg

tggatttggaaattggcttgtaccattaatattaggagcccctgatatagcatttcctcg

aataaataatataagtttttggctactccctccatcattaaccctattattagtaagtag

aatagttgaaaacggagctggaacaggatggactgtatacccaccactatcagcagcaat

tgctcatggagggggatcagttgatttagctattttttcacttcatttagcaggaatttc

atcaattctaggagctgtaaattttattacaacagttattaatatacgatcaactggaat

tacatttgaccgaatacctttatttgtttgggcagtagttattactgctattcttcttct

tttatctttacctgttttagctggtgctattacaatattattaaccgatcgaaacttaaa

tacttcattttttgacccagcaggaggaggtgatcctattttataccaacatttatt-

>KDIPT2586-13|Chrysops distinctipennis|COI-5P|KX946518

aactctatactttattttcggagcttgagccggaataattggaacatccttaagaattct

aattcgtgcagaattaggtcatccaggagccttaattggagatgatcaaatttataatgt

tattgttactgctcatgcatttgttataattttctttatagtaatacctattataatcgg

tggatttggaaattgacttgtaccattaatattgggagcccctgatatagcattccctcg

aataaataatataagtttttgacttcttcctccatcattaaccctattattagtaagtag

aatagttgaaaacggagctggaacaggatgaactgtatacccaccgctatcagcagcaat

tgctcatggaggaggatcagttgatttagctattttttcacttcatttagcaggaatttc

atcaattctaggagctgtaaattttattacaacagttattaatatacgatcaactggaat

tacatttgatcgaatacctttatttgtttgagctgtagttattactgctattcttcttct

tttatctttacctgttttagctggagctattacaatattattaactgatcgaaacttaaa

tacttcattttttgacccagcaggagggggagatcctattctataccaacatttattt

>KMPWN157-19|Chrysops|COI-5P

--ctctatattttattttcggggcttgagccggaataattggaacatccttaagaatttt

aattcgtgcagaattaggtcatccaggagctttaattggagatgatcaaatttataatgt

tattgttactgctcatgcatttgttataattttctttatagtaatacctattataattgg

aggatttggaaattgacttgtaccattaatattaggagcccctgatatagcattccctcg

aataaataatataagtttttgacttcttcctccatcattaactctattattagtaagtag

aatagttgaaaatggggctggaacaggatgaactgtatacccacccctatcagcagcaat

cgctcatggaggaggatcagttgatttagctattttttcacttcatttagcaggaatttc

atcaattttaggggctgtaaattttattacaacagttattaatatgcgatcaactggaat

tacatttgatcgaatacctttatttgtttgagctgtagttattacagctattctccttct

tttatctttacccgttttagctggagctattacaatattattaactgatcgaaacttaaa

tacttcattttttgacccagcaggaggaggtgacccaattttatatcaacacctatt-

>GBDP27285-19|Chrysops flavocinctus|COI-5P|MH998226

------------------------------------------aacatccttaagtatttt

aattcgagctgaactaggtcatccaggagctttaattggtgatgatcaaatttataatgt

aattgtaactgctcatgcatttgtaataattttctttatagttataccaattataattgg

agggtttggaaattgattagtaccattaatattaggagcccctgatatagcatttcctcg

aataaataatataagtttttgacttttacctccttctttaactttattattagttagaag

tatagttgaaaatggagctggaactggttgaactgtttaccctcctttatcagctgcaat

tgctcatggaggaggatcagttgatttagctattttttctcttcatttagccggaatttc

atcaattttaggagctgttaattttattacaactgttattaatatacgatcaactggaat

tacatttgatcgaatacctctatttgtatgagctgttgtaattacagccattcttctttt

attatcacttcctgttttagctggggctattacaatattattaacagatcgaaatttaaa

tacatcattttttgatccagctggaggaggagat------------------------

>GBMND40250-21|Chrysops dispar|COI-5P|MT083918

----------------------------------ataattggaacttctttaagtatttt

aattcgagctgaattaggtcatccaggagctttaattggcgatgaccaaatttataatgt

tattgtaactgctcatgcatttgtaataattttctttatagttataccaattataattgg

aggatttggtaattgacttgtaccattaatattaggagctcctgatatagcatttcctcg

aataaataatataagtttttgattacttcctccatcattaactcttttattagtaagtag

tatagttgaaaatggggctggaacaggatgaactgtatacccaccattatcagcagcaat

tgctcatggagggggttctgttgatttagctatcttttccttacatctagctgggatttc

atcaattttaggagctgtaaattttattacaacagtaattaatatacgatcaactggaat

tacatttgatcgaatacctttatttgtttgagctgttgttattactgcaattcttctttt

attatctcttcctgttttagctggagctattacaatattattaacagatcgaaatttaaa

tacatctttcttcgatcctgcaggaggaggagatccaattttatatcaaca-------

>GBDP16721-15|Chrysops dispar|COI-5P|KM111683

----------------------------------ataattggaacttctttaagtatttt

aattcgagctgaattaggccatccagcagccttaattggtgatgatcaaatttataatgt

tattgtaactgctcatgcatttgtaataattttctttatagttataccaattataattgg

aggatttggtaattgacttgtaccattaatattaggagcacctgatatagcatttcctcg

aataaataatataagtttttgattacttcctccatcattaactcttttattagtaagtag

tatagttgaaaatggagctggaacaggatgaacagtatacccaccattatcagcagcaat

tgctcatggaggaggttctgttgatttagcaattttttcattacatctagctggaatttc

atcaattttaggagctgtaaattttattacaacagtaattaatatacgatcaactggaat

tacatttgatcgaatacccttatttgtttgagccgttgttattactgcaattcttctttt

attatctttacctgttttagcaggagcaatttcaaaattattaacagatcgaaatttaaa

tacatctttctttgatcctgcaggaggaggagacccaatt------------------

>GBDP16724-15|Chrysops dispar|COI-5P|KM111679

----------------------------------ataattggaacttctttaagtatttt

aattcgagctgaattaggccatccaggagccttaattggtgatgatcaaatttataatgt

tattgtaactgctcatgcatttgtaataattttctttatagttataccaattataattgg

aggatttggtaattgacttgtaccattaatattaggagcacctgatatagcatttcctcg

aataaataatataagtttttgattacttcctccatcattaactcttttattagtaagtag

tatagttgaaaatggagctggaacaggatgaacagtatacccaccattatcagcagcaat

tgctcatggaggaggttctgttgatttagcaattttttcattacatctagctggaatttc

atcaattttaggagctgtaaattttattacaacagtaattaatatacgatcaactggaat

tacatttgatcgaatacccttatttgtttgagccgttgttattactgcaattcttctttt

attatctcttcccgttttagctggagcaattacaatattattaacagatcgaaatttaaa

tacatctttctttgatcctgcaggaggaggagatcca---------------------

>GBDP16728-15|Chrysops dispar|COI-5P|KM111675

----------------------------------ataattggaacttctttaagtatttt

aattcgagctgaactaggtcatccaggagctttaattggtgatgatcaaatttataatgt

tattgtaactgctcatgcatttgtaataattttctttatagtaataccaattataattgg

aggatttggtaattgacttgtaccattaatattaggagcccctgatatagcatttcctcg

aataaataatataagtttttgattacttcctccatcattaacccttttattagtaagcag

tatagttgaaaatggagctggaacaggatgaactgtatatccaccattatcagcagcaat

tgctcatggagtgggttctgttgatttagccattttttctttacaccttgctggaatttc

atcaattttaggagctgtaaattttattacaacagtaattaatatacgatcaactggaat

tacatttgatcgaataccattatttgtctgagctgttgttattactgcaattcttctttt

attatctcttcctgttttagccggagctattacaatactattaacagatcgaaatttaaa

tacatctttctttgatcctgcaggtggaggagagcc----------------------

>GBDP16729-15|Chrysops dispar|COI-5P|KM111674

----------------------------------ataattggaacttctttaagtatttt

aattcgagctgaactaggtcatccaggagctttaattggtgatgatcaaatttataatgt

tattgtaactgctcatgcatttgtaataattttctttatagtaataccaattataattgg

aggatttggtaattgacttgtaccattaatattaggagcccctgatatagcatttcctcg

aataaataatataagtttttgattacttcccccatcattaacccttttattagtaagcag

tatagttgaaaatggagctggaacaggatgaactgtatatccaccattatcagcagcaat

tgctcatggagggggttctgttgatttagccattttttctttacaccttgctggaatttc

atcaattttaggagctgtaaattttattacaacagtaattaatatacgatcaactggaat

tacatttgatcgaataccattatttgtctgagctgttgttattactgcaattcttctttt

attatctcttcctgttttagccggagctattacaatactattaacagatcgaaatttaaa

tacatctttctttgatcctgcaggtggaggagatccaatt------------------

>GBMIN38548-13|Chrysops coquillett|COI-5P|DQ983512

------------------------------------------------------------

------------------------------------------------------------

------------------------------------------------tattataattgg

aggatttggaaattgattagttccattaatattaggagctcctgatatagcattcccccg

aataaataatataagtttttgactacttcctccttcattaactcttttattagttagaag

tatagttgaaaacggagctggaactggatgaacagtataccccccattatcagctgctat

tgctcatggtggaggatcagttgatctagcaattttctctcttcatttagctggaatttc

atcaattttaggggctgtaaattttattactacagtaattaatatacgatcaacaggaat

tacatttgaccgaataccattatttgtttgagctgttgttattactgctattcttttatt

attatcattacctgttttagctggagctattactatattattaactgatcgaaatttaaa

tacatcattttttgacccagctggaggaggagaccctattttataccaacatttattt

>SSWLD3935-13|Silvius gigantulus|COI-5P|KM648312

aacactttattttattttcggagcatgagctggaataattggaacttcattaagaatttt

aattcgagctgaacttggtcatcctggatctttaattggtgatgatcaaatttataatgt

aattgtaacagctcatgcttttgttataattttctttatagtaatacctattataattgg

aggatttggaaactgattagtacctttaatattaggagctcccgatatagcatttccacg

aataaataatataagattttgacttcttcctccatctttaacattattattagtaagtag

catagttgaaaatggagctggaacaggatgaactgtttatccaccattatcttctacaat

tgctcatagaggaggatcagttgatttagcaattttttcattgcatttagcaggaatttc

atctattttaggagctgtaaattttattacaacagtaattaatatacgatctacaggtat

tacttttgatcgaatacctttatttgtttgatctgttgtaattacagctattttactttt

attatctttaccagttcttgctggagctattactatattattaacag-------------

----------------------------------------------------------

>CROBB315_SK-31_Silvius alpinus

aacactttattttatttttggagcttgagccggaataattggaacttcattaagaatttt

aattcgagctgaacttggtcatccaggagctttaattggagatgatcaaatttataatgt

aattgtaacagctcatgcttttgttataattttctttatagttatacctattataattgg

aggatttggaaattgattagtacctttaatattgggagctcctgatatagcattccctcg

aataaacaatataagtttttgacttcttcctccatctttaacattattattagtaagtag

tatagttgaaaatggagctggaacaggatgaactgtatatccacctttatcatctacaat

tgctcatagtggtggatcagttgacttagcaattttttcattacacttagctggaatctc

atctattttaggagctgtaaattttattacaacagtaattaatatgcgatctacaggtat

tacttttgatcgaatacctttatttgtttgatctgttgtaattacagctattttactttt

attatctttaccagttcttgctggagctattactatattattaacagatcgaaatttaaa

tacttcattttttgatccagcaggtggaggagatccaattctatatcaacatttattt

>KDIPT2564-13|Chrysops brucei|COI-5P|KX946516

tacattatattttatttttggagcatgagccggaataattggtacatctttaagaatttt

aattcgagctgaattagggcatccaggttcattaattggagacgatcaaatttataatgt

aattgtaacagcacatgcttttgttataattttctttatagtaatacctattataattgg

aggatttggaaattgattagttcctttaatattaggagctcctgatatggcctttccccg

aataaataatataagtttttgattattacccccgtcattgactcttttattagccagtag

tatagtagaaaatggagctgggactggatgaacagtttacccaccactatctgctgctat

tgctcatggaggaggatcagttgatttagctattttctctcttcatttagccggaatttc

ttctattttaggggctgttaattttattactactgtaattaatatacgatccacaggaat

tacatttgatcgaataccactatttgtttgagctgtagtaattactgcaattcttttatt

attatctttaccagtattagccggagctattacaatacttttaacagatcgaaatttaaa

tacatcattttttgacccagcaggtggaggagacccaattttatatcaacatttattt

>ASIND3603-12|Silvius tanycerus|COI-5P

aactctttattttattttcggagcttgagctggaataattggaacctcattaagaatttt

aattcgagctgaattaggtcatcctggagcattaattggagatgatcaaatttataatgt

aattgtaactgcccatgcttttgtaataattttctttatagttataccaattataattgg

tggattcggaaattgattagtacctttaatattaggggctcctgatatagcattcccacg

gataaataatataagtttttgactattacccccatctttaacattattattagttagaag

tatagttgaaaatggggctggtactggatgaactgtctaccctcccttatctgcagctat

tgctcatggaggaggatctgttgatttagccattttctctttacacctagctggaatttc

ttctattttaggagcagtaaactttattaccactgttattaatatacgatcagtaggaat

tacttttgatcgtatacctttatttgtctgagctgtggtaattactgcagtattattatt

attatctctcccagttctagccggagctattaccatattattaacagatcgaaatttaaa

tacttcattttttgatcctgcgggaggaggagacccaattttataccaacatttattt

>ASIND3605-12|Silvius tanycerus|COI-5P

aactctttattttattttcggagcttgagctggaataattggaacctcattaagaatttt

aattcgagctgaattaggacatcctggagcattaattggagatgatcaaatttataatgt

aattgtaactgcccatgcttttgtaataattttctttatagttataccaattataattgg

tggattcggaaactgattagtacctttaatattaggagcccctgatatagcattcccacg

aataaataatataagtttttgactattacccccatctttaacgttattgttagttagaag

tatagttgaaaatggggctggtactggatgaactgtttatcctcctttatctgcggctat

tgctcatggagggggatctgttgatttagccattttctctttacacttagctggaatttc

ttctattttaggggcagtaaactttattaccactgttattaatatacgatcagtaggaat

tacttttgatcgtatgcctttatttgtctgagctgtagtaattactgcagtattattatt

attatctctcccagttctagccggagctattaccatattattaacagatc----------

----------------------------------------------------------

>ASIND3607-12|Silvius tanycerus|COI-5P

aactctttattttattttcggagcttgagctggaatagttggaacttcattaagaatttt

aattcgagctgaattaggtcaccctggagcattaattggagatgatcaaatttataatgt

aattgtaactgctcatgcctttgtaataattttctttatagttataccaatcataattgg

tggattcggaaattgattagtacctttaatattaggagcccctgatatagcatttccacg

aataaataatataagtttttgattattacctccatctttaacattattattagttagaag

tatagttgaaaatggagctggtactgggtgaactgtttatccccccttatctgcagctat

tgctcatggaggagggtctgttgatttagctattttttctttacatctagctggaatttc

ttcaattttaggggcagtaaactttatcactactgttattaatatgcgatcagtaggaat

tacttttgatcgtatacctttatttgtttgagctgtagtaattactgcagtattattatt

attatctctcccagttctagccggagctattactatactattaacagatcgaaatctaaa

tacttcattttttgaccctgcaggagggggagacccaattttatatcaacatttattt

>GBDP15907-15|Silvius quadrivittatus|COI-5P|KM243548

---------------tttcggagcttgagctgggataattggaacttccctaagaatttt

aattcgtgctgaattaggtcatcctggggctttaattggtgatgatcaaatttataatgt

aattgtaactgctcatgcttttgttataattttctttatagttatgccaattataattgg

agggtttggaaattgattagttcctttaatattaggagcccctgatatagcattccctcg

tataaataatataagtttttgacttttaccgccatctttaacactattattagtcagtag

tatagtggaaaacggggctggaactggatgaactgtttatcccccattatctgctgctat

tgcccatggaggaggatcagttgatttagccattttttctctacatttagctggaatttc

atctattttaggagctgtaaattttattactactgtaattaatatacgatcaacaggaat

tacttttgatcgtataccattatttgtatgagctgtagttattactgctgtactattatt

actttcattaccagttttagctggagcaattacaatattattaacagatcgaaatttaaa

tacatcattttttgacccagctggaggaggagatcctattttataccaacatttattt

>GBDP15874-15|Silvius variegatus|COI-5P|KM243506

----------------ttcggagcttgagctggaataattggaacttcattaagaattct

tattcgggctgaattaggtcatcctggagcattaattggtgatgatcaaatttataatgt

aattgtaacagctcatgcctttgttataattttttttatagtaatacctattataattgg

aggatttggaaattgattagtccctttaatactgggagcccctgatatagcattccctcg

aataaataatataagattttgattacttcccccctctttaactttactgttagtaagtag

aatagttgaaaacggagctggaactgggtgaacagtttacccccctctatcagcagctat

tgctcatggaggaggatcagttgacttagctattttttctcttcatttagctggaatttc

ttctattttaggagccgttaattttattactacagtaattaatatacgatctagaggaat

tacttttgatcgaatacctttatttgtatgagcagttattattacagctattttactatt

attatcattacctgttcttgctggggcaattactatattactaacagaccgaaatttaaa

tacttcattttttgatcctgccggaggaggagaccc----------------------

>GBMIN38555-13|Silvius gigantulus|COI-5P|DQ983531

------------------------------------------------------------

------------------------------------------------------------

------------------------------------------------------------

----tttggaaattgattagtacctttaatattaggagcccctgatatagcatttccacg

aataaataatataagattttgacttcttcctccatctttaacattattattagtaagcag

tatagttgaaaatggagctggaacaggatgaactgtttatccaccattatcttctacaat

tgctcatagaggaggatcagttgatttagcaattttttcactacatttagctggaatttc

atctattttaggagctgtaaattttattacaacagtaattaatatacgatctacaggtat

tacttttgatcgaatacctttatttgtttgatctgttgtaattacagctattttactttt

attatctttaccagttcttgctggagctattactatattattaacagatcgaaatttaaa

tacttcattttttgacccagctggaggaggagatccaattttatatcaacatttattt

>AMTPD3344-15|Chrysopilus nubecula|COI-5P

-actttatattttatttttggggcatgggcaggaatagtaggtacttcacttagtatact

aattcgagcagaactaggtcatcctggagctttaattggtgatgatcaaatttataatgt

aatcgttaccgctcatgcttttgtaataattttctttatagtaatgcctattataatcgg

aggatttggtaattgattagtcccattaatattgggggctcctgatatagcatttccacg

aataaataatataagattctgattattacccccctctttaactttattgttagcaagtag

tatagtagaaaatggggcaggtactggatgaactgtctaccctcccctttcagctggaat

tgcccacgctggagcttctgtagatctcgctattttttctttacatcttgcaggagtctc

ctcaattttaggtgccgtaaattttattacaacagtaattaatatacgaacaacgggtat

tacttttgaccgaatgccattatttgtttgagccgtagtaattacagcaattttattact

actatccttaccagtattagccggagctattacaatattattaacagat-----------

----------------------------------------------------------

>AMTPD3492-15|Symphoromyia crassicornis|COI-5P

----------ttatttttgggggcatgagcaggtatagttggtacttctctaagaatact

aattcgagctgaactaggccacccaggagccttaattggtgatgatcaaatctataatgt

aattgttacagctcatgcttttgtaataattttttttatagtaatacctattataattgg

aggatttggtaattgattagtcccattaatattaggagccccagatatagcatttccacg

aataaataatataagattctgattactgcccccatctttaactctattattagcaagaag

catagtagaaaatggagcaggaacaggatgaacagtatatccccccctatcggccagaat

tgcacatgctggagcttctgttgatttagctattttttcattgcatttagccggagtttc

ttctattttaggggctgtaaattttattacaacagttattaatatacgatcaacaggaat

ttcgtttgatcgaatacctctatttgtatgagcggttgtaattactgctattcttttatt

actatcactaccagtattagcaggagctattacaatactattaacagat-----------

----------------------------------------------------------

>GMFIE626-12|Rhagio maculatus|COI-5P

aacattatattttatttttggggcttgagctggaatagtaggaacatctttaagtatatt

aattcgagctgaattaggacacccaggagccttaattggtgatgatcaaatttataatgt

aattgttactgctcatgcctttgtaataattttctttatagtaatgcctattataattgg

aggatttggaaattgattagtacctttaatattaggggctccagatatagccttcccccg

aataaataatataagattttgattattacctccctcattaactttacttttatctagtag

tatagtagaaaatggagcaggtactggatgaacagtttaccctccgctttctgcctctat

tgcccatggaggggcctctgttgatttagccattttctcccttcatttagctggtatttc

ttctattttaggggcagtaaattttattaccacagtaattaatatacgatcaacaggaat

tacatttgatcgaatacccctatttgtatgagctgttgtaattactgctattcttttatt

attatccttacctgtattagcaggtgcaattactatattattaacagatcgaaatctaaa

tacatcgttctttgaccctgctggtgggggagaccctattctataccaacatttattt

2. HAEMATOPOTINI AND HEPTATOMINI

>ACT059-07|Haematopota americana|COI-5P|KM285558

----------------------------------------ggaacttcattaagtattctaattcgagctgaattgggacatccaggatcattaattggtgatgatcaaatttataatgtaattgtaacagcacatgcttttgtaataattttttttatggtaatacctattataattggaggatttggaaattgattagttccattaatattaggagctcctgatatrgcatttcctcgaataaataatataagtttttgacttttacccccatcattgactcttttattagcaagtagtatagtagaaaatggagctggaactggttgaacagtttaccccccattatcagcagcaattgcccatggaggaggatcagtagatttagcaattttttctttacatcttgcaggaatttcttctattttaggagctgttaatttcattactactgtaattaatatacgatcaactggaattacatttgatcgaatacctttatttgtatgagctgtagtaattactgccattcttttattattatctttaccagtattagcaggtgctattactatacttttaacagaccgaaatttaaatacctctttttttgacccagcaggaggaggtgatccaattttataccaacatttattt

>GBDP15888-15|Haematopota singularis|COI-5P|KM243524

--------attttatttttggggcatgagccggaataattggaacttcattaagtattctaattcgagctgaattaggacatccagggtctttaattggtgatgaccaaatttataatgtaattgtaacagcacatgcttttgtaataattttctttatagtaatacctattataattggaggatttggaaattgattagttccactaatattaggagctcctgatatagcatttcctcgaataaataatataagattttgacttttacccccctcattaactcttttattagcaagtagtatagtagaaaatggagctggtactggttgaacagtttaccccccattatcagcagctattgctcatggaggaggatcagtagatttagcaattttttctttacatttagcaggaatttcttctattttaggagctgtaaattttattactactgtaattaatatacgatcaacaggaattacatttgatcgaatacctctatttgtatgagctgtagtaattactgctattcttttattattatctttaccagtattagctggtgcaattactatacttttaacagatcgaaatttaaatacatcattttttgatccagcaggaggaggagatcctattttatatc-----------

>GBDP26294-19|Haematopota singularis|COI-5P|MF144226

aacattatattttatttttggggcatgagccggaataattggaacttcattaagtattctaattcgagctgaattaggacatccaggatctttaattggtgatgaccaaatttataatgtaattgtaacagcacatgcttttgtaataattttctttatagtaatacctattataattggaggatttggaaattgattagttccactaatattaggagctcctgatatagcatttcctcgaataaataatataagattttgacttttacccccctcattaactcttttattagcaagtagtatagtagaaaatggagctggtactggttgaacagtttatcccccattatcagcagctattgctcatggaggaggatcagtagatttagcaattttttctttacatttagcaggaatttcttctattttaggagctgtaaattttattactactgtaattaatatacgatcaacaggaattacatttgatcgaatacctctatttgtatgagctgtagtaattactgctattcttttattattatctttaccagtattagctggtgcaattactatacttttaacagatcgaaatttaaatacatcattttttgatccagcaggaggaggagatcctattttatatcaacacttattt

>GBDP26295-19|Haematopota singularis|COI-5P|MF144227

aacattatattttatttttggggcatgagccggaataattggaacttcattaagtattctaattcgagctgaattaggacatccagggtctttaattggtgatgaccaaatttataatgtaattgtaacagcacatgcttttgtaataattttctttatagtaatacctattataattggaggatttggaaattgattagttccactaatattaggagctcctgatatagcatttcctcgaataaataatataagattttgacttttacccccttcattaactcttttattagcaagtagtatagtagaaaatggagctggtactggttgaacagtttatcccccattatcagcagctattgctcatggaggaggatcagtagatttagcaattttttctttacatttagcaggaatttcttctattttaggagctgtaaattttattactactgtaattaatatacgatcaacaggaattacatttgatcgaatacctctatttgtatgagctgtagtaattactgctattcttttattattatctttaccagtattagctggtgcaattactatacttttaacagatcgaaatttaaatacatcattttttgatccagcaggaggaggagatcctattttatatcaacacttattt

>GBDP26278-19|Haematopota cilipes|COI-5P|MF144210

aacattatattttatttttggggcatgagccggaataattggaacttcattaagtattttaattcgagccgaattaggacacccaggatctttaattggtgatgatcaaatttataatgtaattgtaacagcccatgcttttgtaataattttctttatagtaatacctattataattgggggatttggtaattgattagttccattaatattaggagctcctgatatagcatttcctcgaataaataatataagattttgacttttacctccttcattaactctcttattagcaagtagtatagtagaaaatggagctggtactggttgaacagtttaccccccattatcagcagctattgcccatggaggaggatcagttgatttagctattttttctttacatcttgcaggaatttcttctattctaggagctgtaaattttattactactgtaattaatatacgatcaacaggaattacatttgatcgaatacctttatttgtatgagctgtagtaattactgctattcttttattattatctttaccagtattagctggtgctattacaatacttttaacagatcgaaatttaaatacatcattttttgatcctgcaggaggaggagaccctattttataccaacatttattt

>GBDP26277-19|Haematopota cilipes|COI-5P|MF144209

aacattatattttatttttggggcatgagccggaataattggaacttcattaagtattttaattcgagccgaattaggacacccaggatctttaattggtgatgatcaaatttataatgtaattgtaacagcccatgcttttgtaataattttctttatagtaatacctattataattgggggatttggtaattgattagttccattaatattaggagctcctgatatagcatttcctcgaataaataatataagattttgacttttacctccttcattaactctcttattagcaagtagtatagtagaaaatggagctggtactggttgaacagtttaccccccattatcagcagctattgcccatggaggaggatcagttgatttagctattttttctttacatcttgcaggaatttcttctattctaggagctgtaaattttattactactgtaattaatatacgatcaacaggaattacatttgatcgaatacctttatttgtatgagctgtagtaattactgctattcttttattattatctttaccagtattagctggtgctattacaatacttttaacagatcgaaatttaaatacatcattttttgatcctgcaggaggaggagaccctattttataccaacatttattt

>GBDP26281-19|Haematopota glenni|COI-5P|MF144213

aacattatattttatttttggtgcttgagcaggaataattggaacatcattaagaattctaattcgagctgaattaggacatccaggatccttaattggtgatgatcaaatttataatgtaattgtaacagcccatgcttttgtaataattttctttatagtaatacctattataattggaggatttggaaattgattagttccattaatattaggagctcctgatatagcatttcctcgaataaataatataagattttgacttttacctccatcattaactctcttattagcaagtagtatagtagaaaatggagctggtactggttgaacagtttatccaccattatcagcagctattgctcatggaggaggatcagtagatttagcaattttttctttacatcttgcaggaatttcttctattttaggagctgtaaattttattactactgtaattaatatacgatcaacaggaattacatttgaccgaatacccttatttgtatgagctgtagtaattactgctattcttttattattatctttacccgtattagcaggtgctattactatacttttaacagatcgaaatttaaatgcatcattttttgatcctgcaggaggaggagatcctattttacaccaacatttattt

>GBDP26282-19|Haematopota glenni|COI-5P|MF144214

aacattatattttatttttggtgcttgagcaggaataattggaacatcattaagaattctaattcgagctgaattaggacatccaggatccttaattggtgatgatcaaatttataatgtaattgtaacagcccatgcttttgtaataattttctttatagtaatacctattataattggaggatttggaaattgattagttccattaatattaggagctcctgatatagcatttcctcgaataaataatataagattttgacttttacctccatcattaactctcttattagcaagtagtatagtagaaaatggagctggtactggttgaacagtttatccaccattatcagcagctattgctcatggaggaggatcagtagatttagcaattttttctttacatcttgcaggaatttcttctattttaggagctgtaaattttattactactgtaattaatatacgatcaacaggaattacatttgaccgaatacccttatttgtatgagctgtagtaattactgctattcttttattattatctttacccgtattggcaggtgctattactatacttttaacagatcgaaatttaaatgcatcattttttgatcctgcaggaggaggagatcctattttataccaacatttattt

>GBDP26303-19|Haematopota glenni|COI-5P|MF144235

aacattatattttatttttggtgcttgagcaggaataattgggacatcattaagaattctaattcgagctgaattagggcatccaggatccttaattggagatgatcaaatttataatgtaattgtaacagcccatgcttttgtaataattttctttatagtaatacctattataattggaggatttggaaattgattagttccattaatattaggagctcctgatatagcatttcctcgaataaataatataagattttgacttttacctccatcattaactctcttattagcaagtagtatagtagaaaatggggctggtactggttgaacagtttacccaccattatcagcagctattgctcatggaggaggatcagtagatttagcaattttttctttacatcttgcaggaatttcttctattttaggggctgtaaattttattactactgtaattaatatacgatcaacaggaattacattcgaccgaatacccttatttgtatgagctgtagtaattactgctattcttttattattatctttacctgtattggcaggtgctattactatacttttaacagatcgaaatttaaatacatcattttttgaccctgcgggaggaggagaccctattttataccaacatttattt

>GBDP26304-19|Haematopota glenni|COI-5P|MF144236

aacattatattttatttttggtgcttgagcaggaataattgggacatcattaagaattctaattcgagctgaattaggacatccaggatccttaattggagatgatcaaatttataatgtaattgtaacagcccatgcttttgtaataattttctttatagtaatacctattataattggaggatttggaaattgattagttccattaatattaggagctcctgatatagcatttcctcgaataaataatataagattttgacttttacctccatcattaactctcttattagcaagtagtatagtagaaaatggagctggtactggttgaacagtttacccaccattatcagcagctattgctcatggaggaggatcagtagatttagcaattttttctttacatcttgcaggaatttcttctattttaggagctgtaaattttattactactgtaattaatatacgatcaacaggaattacattcgaccgaatacccttatttgtatgagctgtagtaattactgctattcttttattattatctttacctgtattggcaggtgctattactatacttttaacagatcgaaatttaaatacatcattttttgatcctgcaggaggaggagatcctattttataccaacatttattt

>GBDP26275-19|Haematopota cilipes|COI-5P|MF144207

aacattatattttatttttggtgcttgagccggaataattggaacttcattaagtattttaattcgtgctgaattagggcatccaggatctttaattggtgatgatcaaatttataatgtaattgtaacagcacatgcttttgtaataattttctttatagtaatacctattataattggaggatttggaaattgattagttccattaatattaggagctcctgatatagcatttcctcgaataaataatataagattttgacttttacctccatcattaactcttttattagcaagtagtatagtagaaaacggagctggtactggttgaacagtttatcccccattatcagcagctattgctcacggaggaggatcagtagatttagcaattttttctttacatcttgcaggaatttcatctattttaggagctgtaaattttattactactgtaattaatatacgatcaacaggaattacatttgatcgaatacctttatttgtatgagctgtagtaattactgctattcttttattactatctttaccggtattagcaggtgctattactatacttttaacagatcgaaatttaaatacatcattttttgacccagctggaggaggagaccctattttataccaacatttattt

>GBDP26276-19|Haematopota cilipes|COI-5P|MF144208

aacattatattttatttttggtgcttgagccggaataatcggaacttcattaagtattttaattcgtgctgaattaggacatccaggatctttaattggtgatgatcaaatttataatgtaattgtaacagcacatgcttttgtaataattttctttatagtaatacctattataattggaggatttggaaattgattagttccattaatattaggagctcctgatatggcatttcctcgaataaataatataagattttgacttttacctccatcattaactcttttattagcaagtagtatagtagaaaatggagctggtactggttgaacagtttatcccccattatcagcagctattgctcacggaggaggatcagtagatttagcaattttttctttacatcttgcaggaatttcatctattttaggagctgtaaattttattactactgtaattaatatacgatcaacaggaattacatttgatcgaatacctttatttgtatgagctgtagtaattactgctattcttttattactatctttaccggtattagcaggagctattactatacttttaacagatcgaaatttaaatacatcattttttgatccagccggaggaggagatcctattttataccaacatttattt

>GBDP26293-19|Haematopota procyon|COI-5P|MF144225

aacattatattttatttttggtgcatgagctggaataattgggacctcattaagtatcctaattcgagctgaattaggacacccaggatctttaattggtgatgaccaaatttataatgtaattgtaacagcacatgcctttgtaataattttctttatagtaatacctatcataattggagggtttggaaattgattagtcccattaatgttaggagctcctgatatagcatttcctcgaataaataatataagattttgacttttacctccatcattaactcttttattaacaagtagtatagtagaaaatggagctggaacaggttgaacagtttaccccccattatcagcagctattgctcatggaggaggatcagtagatttagcaattttttctctacatcttgcaggaatctcttctattttaggggctgtaaattttattactactgtaattaatatacgatcaacaggaattacatttgatcgaatacctttatttgtatgagcagtagtgattactgctattcttttattattatctttaccagtattggcaggtgctattactatactcttaacagatcgaaatttaaatacttctttttttgatccggcaggaggaggagatcctattttataccaacatttattt

>GBDP26297-19|Haematopota procyon|COI-5P|MF144229

aacattatattttatttttggtgcatgagctggaataattggaacctcattaagtatcctaattcgagctgaattaggacacccaggatctttaattggtgatgaccaaatttataatgtaattgtaacagcacatgcttttgtaataattttctttatagtaatacctattataattggagggtttggaaattgattagtcccattaatgttaggagctcctgatatagcatttcctcgaataaataatataagattttgacttttacctccatcattaactcttttattagcaagtagtatagtagaaaatggagctggaacaggttgaacagtttaccccccattatcagcagctattgctcatggaggaggatcagtagatttagcaattttttctctacatcttgcaggaatctcttctattttaggggctgtaaattttattactactgtaattaatatacgatcaacaggaattacatttgatcgaatacctttatttgtatgagcagtagtgattactgctatccttttattattatctttaccagtattggcaggtgctattactatactcttaacagatcgaaatttaaatacttctttttttgatccggcaggaggaggagatcctattttataccaacatttattt

>GBDP26296-19|Haematopota procyon|COI-5P|MF144228

aacattatattttatttttggtgcatgagctggaataattggaacctcattaagtatcctaattcgagctgaattaggacacccaggatctttaattggtgatgaccaaatttataatgtaattgtaacagcacatgcttttgtaataattttctttatagtaatacctattataattggagggtttggaaattgattagtcccattaatgttaggagctcctgatatagcatttcctcgaataaataatataagattttgacttttacctccatcattaactcttttattagcaagtagtatagtagaaaatggagctggaacaggttgaacagtttaccccccattatcagcagctattgctcatggaggaggatcagtagatttagcaattttttctctacatcttgcaggaatctcttctattttaggggctgtaaattttattactactgtaattaatatacgatcaacaggaattacatttgatcgaatacctttatttgtatgagcagtagtgattactgctattcttttattattatctttaccagtattggcaggtgctattactatactcttaacagatcgaaatttaaatacttctttttttgatccggcaggaggaggagatcctattttataccaacatttattt

>GBDP26292-19|Haematopota procyon|COI-5P|MF144224

aacattatattttatttttggtgcatgagctggaataattgggacctcattaagtatcctaattcgagctgaattaggacacccaggatctttaattggtgatgaccaaatttataatgtaattgtaacagcacatgcctttgtaataattttctttatagtaatacctatcataattggagggtttggaaattgattagtcccattaatgttaggagctcctgatatagcatttcctcgaataaataatataagattttgacttttacctccatcattaactcttttattagcaagtagtatagtagaaaatggagttggaacaggttgaacagtttaccccccattatcagcagctattgctcatggaggaggatcagtagatttagcaattttttctctacatcttgcaggaatctcttctattttaggggctgtaaattttattactactgtaattaatatacgatcaacaggaattacatttgatcgaatacctttatttgtatgagcagtagtgattactgctattcttttattattatctttaccagtattggcaggtgctattactatactcttaacagatcgaaatttaaatacttctttttttgatccggcaggaggaggagatcatcttttataccaacatttattt

>GBDP26286-19|Haematopota tenasserimi|COI-5P|MF144218

aacattatattttatttttggggcatgagccggaataattgggacttcattatgtattctaattcgagctgaattagggcacccaggatctttaattggtgatgatcaaatttataatgtaattgtaacagcacatgcatttgtaataattttctttatagtaatacctattataattgggggatttggtaattgattagtcccattaatattaggagcccctgatatagcatttcctcgaataaataatataagattttgacttttacctccatcattaactcttttattagcaagtagtatagtagaaaatggggctggtacaggttgaacagtttaccctcctttatcagcagctattgctcatggaggtggatcagtagatttagcaattttttctttacaccttgcaggaatttcttctattttaggagctgtaaattttattactactgtaattaatatacgatcaacaggaattacatttgatcgaatacctttatttgtatgagctgtagtaattactgctattcttttattattatctttaccagtattagcaggagctattactatacttttaacagatcgaaatttaaatacttccttctttgaccctgcaggaggaggagaccctattttatatcaacatttattt

>GBDP26285-19|Haematopota tenasserimi|COI-5P|MF144217

aacattatattttatttttggggcatgagccggaataattggaacttcattaagtattctaattcgagctgaattaggacacccaggatccttaattggtgatgatcaaatttataatgtaattgtaacagcacatgcatttgtaataattttctttatagtaatacctattataattggagggtttggtaattgattagtcccattaatattaggagcccctgacatagcatttcctcgaataaataatataagattttgacttttacccccatcattaactcttttattagcaagtagtatagtagaaaatggagctggtacaggttgaacaatttatccccctttatcagcagctattgctcatgggggtggatcagtagatttagcaattttttctttacaccttgcaggaatttcttctattttaggagctgtaaattttattactactgtaattaatatacgatcaacaggaattacatttgatcgaatacctttattcgtatgagctgtagtaattactgctattcttttattattatctttaccagtattagcaggagctattactatacttttaacagatcgaaatttaaatacctccttctttgaccctgcaggaggaggagatcctattttatatcaacatttattt

>GBDP15900-15|Haematopota howarthi|COI-5P|KM243539

---------ttttatttttggtgcatgagccggaataattggaacttcattaagtattttaattcgagctgaattaggacacccgggttctttaattggtgatgaccaaatttataatgtaattgtaacagcacatgcttttgtaataattttctttatagttatacctattataattggaggattcggaaattgattagttccattaatattaggagctcctgatatagcttttcctcgaataaataatataagattttgacttttacctccatcattaactcttttattagcaagtagtatagtagaaaatggagcgggtactggttgaacagtttatcccccgttatcagcagctattgctcatggaggaggatcagtagatttagcaattttttctttacatcttgcaggtatttcttctattttaggagctgtaaattttattactactgtaattaatatacgatcaacaggaattacctttgatcgaatacctttatttgtttgagctgtagtaattacagcaattcttttattattatctttaccagtattagcgggagctattactatactcttaacagatcgaaatttaaatacttcattttttgacccagcaggaggaggagatcctattttata-------------

>GBDP26287-19|Haematopota howarthi|COI-5P|MF144219

aacattatattttatttttggtgcatgagccggaataattggaacttcattaagtattttaattcgagctgaattagggcacccgggttctttaattggtgatgaccaaatttataatgtaattgtaacagcacatgcttttgtaataattttctttatagttatacctattataattggaggatttggaaattgattagttccattaatattaggagctcctgatatagcttttcctcgaataaataatataagattttgacttttaccaccatcattaactcttttattagcaagtagtatagtagaaaatggggctggtactggttgaacagtttaccccccattatcagcagctatcgctcatggagggggatcagtagatttagcaattttttctttacatcttgcaggaatttcttctattttaggagctgtaaattttattactactgtaattaatatacgatcaacaggaattacctttgatcgaatacctttatttgtttgagctgtagtaattacagcaattcttttattattatctttaccagtattagctggagctattactatactcttaacagatcgaaatttaaatacttcattttttgacccagcaggaggaggagaccctattttataccaacacttattt

>GMCHE016-14|Haematopota|COI-5P

-acattatactttatttttggggcatgagccggaataattggaacttcattaagtattttaattcgagcagaattaggacacccaggatctttaattggtgatgaccaaatttataatgtaattgtaacagcacatgcttttgtaataattttctttatagttatacctattataattgggggatttggaaattgattagttccattaatattgggggctcctgatatagcattccctcgaataaataatataagtttttgacttttacccccatcattaactcttttattagcaagtagtatagtagaaaatggagctggtacaggttgaacagtttatccccccttatcagctgctattgcccatggaggaggatcagtagatttagcaattttttctttacatttagcaggaatttcttctattttaggagctgttaattttattactactgtaattaatatacgatcaacaggaattacatttgaccgaatacctttatttgtgtgagcagtagtaattactgctattcttttattattatctttaccagtattagcagga---------------------------------------------------------------------------------------------

>GMCHE018-14|Haematopota|COI-5P

-acattatactttatttttggggcatgagccggaataattggaacttcattaagtattttaattcgagcagaattaggacacccaggatctttaattggtgatgaccaaatttataatgtaattgtaacagcacatgcttttgtaataattttctttatagttatacctattataattgggggatttggaaattgattagttccattaatattgggggctcctgatatagcattccctcgaataaataatataagtttttgacttttacccccatcattaactcttttattagcaagtagtatagtagaaaatggggctggtacaggttgaacagtttatccccccttatcagctgctattgcccatggaggaggatcagtagatttagcaattttttctttacatttagcaggaatttcttctattttaggagctgttaattttattactactgtaattaatatacgatcaacaggaattacatttgaccgaatacctttatttgtgtgagcagtagtaattactgctattcttttattattatctttaccagtattagcagga---------------------------------------------------------------------------------------------

>GMCHE025-14|Haematopota|COI-5P

-acattatactttatttttggggcatgagccggaataattggaacttcattaagtattttaattcgagcagaattaggacacccaggatctttaattggtgatgaccaaatttataatgtaattgtaacagcacatgcttttgtaataattttctttatagttatacctattataattgggggatttggaaattgattagttccattaatgttgggggctcctgatatagcattccctcgaataaataatataagtttttgacttttacccccatcattaactcttttattagcaagtagtatagtagaaaatggggctggtacaggttgaacagtttatccccccttatcagctgctattgcccatggaggaggatcagtagatttagcaattttttctttacatttagcaggaatttcttctattttaggagctgttaattttattactactgtaattaatatacgatcaacaggaattacatttgaccgaatacctttatttgtgtgagcagtagtaattactgctattcttttattattatctttaccagtattagcagga---------------------------------------------------------------------------------------------

>GMCHE017-14|Haematopota|COI-5P

-acattatactttatttttggggcatgagccggaataattggaacttcattaagtattttaattcgagcagaattaggacacccaggatctttaattggtgatgaccaaatttataatgtaattgtaacagcacatgcttttgtaataattttctttatagttatacctattataattgggggatttggaaattgattagttccattaatattgggggctcctgatatagcatttcctcgaataaataatataagtttttgacttttacccccatcattaactcttttattagcaagtagtatagtagaaaatggggctggtacaggttgaacagtttatccccccttatcagctgctattgcccatggaggaggatcagtagatttagcaattttttctttacatttagcaggaatttcttctattttaggagctgttaattttattactactgtaattaatatacgatcaacaggaattacatttgaccgaatacctttatttgtgtgagcagtagtaattactgctattcttttattattatctttaccagtattagcagga---------------------------------------------------------------------------------------------

>GMCHE027-14|Haematopota|COI-5P

-acattatactttatttttggggcatgagccggaataattggaacttcattaagtattttaattcgagcagaattaggacacccaggatctttaattggtgatgaccaaatttataatgtaattgtaacagcacatgcttttgtaataattttctttatagttatacctattataattggaggatttggaaattgattagttccattaatattgggagctcctgatatagcattccctcgaataaataatataagtttttgacttttacccccatcattgactcttttattagcaagtagtatagtagaaaatggggctggtacaggttgaacagtttatccccccttatcagctgctattgcccatggaggaggatcagtagatttagcaattttttctttacatttagcaggaatttcttctattttaggagctgttaattttattactactgtaattaatatacgatcaacaggaattacatttgaccgaatacctttatttgtgtgagcagtagtaattactgctattcttttattattatctttaccagtattagcaggg---------------------------------------------------------------------------------------------

>GMCHE028-14|Haematopota|COI-5P

-acattatactttatttttggggcatgagccggaataattggaacttcattaagtattttaattcgagcagaattagggcacccaggatctttaattggtgatgaccaaatttataatgtaattgtaacagcacatgcttttgtaataattttctttatagttatacctattataattgggggatttggaaattgattagttccattaatattgggagctcctgatatagcattccctcgaataaataatataagtttttgacttttacccccatcattgactcttttattagcaagtagtatagtagaaaatggggctggtacaggttgaacagtttatccccccttatcagctgctattgcccatggaggaggatcagtagatttagcaattttttctttacatttagcaggaatttcttctattttaggagctgttaattttattactactgtaattaatatacgatcaacaggaattacatttgaccgaatacctttatttgtgtgagcagtagtaattactgctattcttttattattatctttaccagtattagcaggg---------------------------------------------------------------------------------------------

>GMCHE024-14|Haematopota|COI-5P

-acattatactttatttttggggcatgagccggaataattggaacttcattaagtattttaattcgagcagaattaggacacccaggatctttaattggtgatgaccaaatttataatgtaattgtaacagcacatgcttttgtaataattttctttatagttatacctattataattgggggatttggaaattgattagttccattaatattgggagctcctgatatagcattccctcgaataaataatataagtttttgacttttacccccatcattgactcttttattagcaagtagtatagtagaaaatggggctggtacaggttgaacagtttatccccccttatcagctgctattgcccatggaggaggatcagtagatttagcaattttttctttacatttagcaggaatttcttctattttaggagctgttaattttattactactgtaattaatatacgatcaacaggaattacatttgaccgaatacctttatttgtgtgagcagtagtaattactgctattcttttattattatctttaccagtattagcaggggctattactatacttttaacagat---------------------------------------------------------------------

>GMCHE023-14|Haematopota|COI-5P

-acattatactttatttttggggcatgagccggaataattggaacttcattaagtattttaattcgagcagaattaggacacccaggatctttaattggtgatgaccaaatttataatgtaattgtaacagcacatgcttttgtaataattttctttatagttatacctattataattgggggatttggaaattgattagttccattaatattgggagctcctgatatagcattccctcgaataaataatataagtttttgacttttacccccatcattgactcttttattagcaagtagtatagtagaaaatggggctggtacaggttgaacagtttatccccccttatcagctgctattgcccatggaggaggatcagtagatttagcaattttttctttacatttagcaggaatttcttctattttaggagctgttaattttattactactgtaattaatatacgatcaacaggaattacatttgaccgaatacctttatttgtgtgagcagtagtaattactgctattcttttattattatctttaccagtattagcaggggctattactatacttttaacagat---------------------------------------------------------------------

>GMCHE030-14|Haematopota|COI-5P

-acattatactttatttttggggcatgagccggaataattggaacttcattaagtattttaattcgagcagaattaggacacccaggatctttaattggtgatgaccaaatttataatgtaattgtaacagcacatgcttttgtaataattttctttatagttatacctattataattgggggatttggaaattgattagttccattaatattgggggctcctgatatagcattccctcgaataaataatataagtttttgacttttacccccatcattaactcttttattagcaagtagtatagtagaaaatggggctggtacaggttgaacagtttatccccccttatcagctgctattgcccatggaggaggatcagtagatttagcaattttttctttacatttagcaggaatttcttctattttaggagctgttaattttattactactgtaattaatatacgatcaacaggaattacatttgaccgaatacctttatttgtgtgagcagtagtaattactgctattcttttattattatctttaccagtattagcaggagctattactatacttttaacagat---------------------------------------------------------------------

>GMCHE020-14|Haematopota|COI-5P

-acattatactttatttttggggcatgagccggaataattggaacttcattaagtattttaattcgagcagaattaggacacccaggatctttaattggtgatgaccaaatttataatgtaattgtaacagcacatgcttttgtaataattttctttatagttatacctattataattgggggatttggaaattgattagttccattaatattgggggctcctgatatagcatttcctcgaataaataatataagtttttgacttttacccccatcattgactcttttattagcaagtagtatagtagaaaatggggctggtacaggttgaacagtttatccccccttatcagctgctattgcccatggagggggatcagtagatttagcaattttttctttacatttagcaggaatttcttctattttaggagctgttaattttattactactgtaattaatatacgatcaacaggaattacatttgaccgaatacctttatttgtgtgagcagtagtaattactgctattcttttattattatctttaccagtattagcaggggctattactatacttttaacagat---------------------------------------------------------------------

>GMCHE022-14|Haematopota|COI-5P

-acattatactttatttttggggcatgagccggaataattggaacttcattaagtattttaattcgagcagaattaggacacccaggatctttaattggtgatgaccaaatttataatgtaattgtaacagcacatgcttttgtaataattttctttatagttatacctattataattgggggatttggaaattgattagttccattaatattgggggctcctgatatagcattccctcgaataaataatataagtttttgacttttacccccatcattaactcttttattagcaagtagtatagtagaaaatggggctggtacaggttgaacagtttatccccccttatcagctgctattgcccatggaggaggatcagtagatttagcaattttttctttacatttagcaggaatttcttctattttaggagctgttaattttattactactgtaattaatatacgatcaacaggaattacatttgaccgaatacctttatttgtgtgagcagtagtaattactgctattcttttattattatctttaccagtattagcaggagctattactatacttttaacagatcgaaat---------------------------------------------------------------

>GMCHE029-14|Haematopota|COI-5P

-acattatactttatttttggggcatgagccggaataattggaacttcattaagtattttaattcgagcagaattaggacacccaggatctttaattggtgatgaccaaatttataatgtaattgtaacagcacatgcttttgtaataattttctttatagttatacctattataattgggggatttggaaattgattagttccattaatgttgggagctcctgatatagcattccctcgaataaataatataagtttttgacttttacccccatcattaactcttttattagcaagtagtatagtagaaaatggggctggtacaggttgaacagtttatccccccttatcagctgctattgcccatggaggaggatcagtagatttagcaattttttctttacatttagcaggaatttcttctattttaggagctgttaattttattactactgtaattaatatacgatcaacaggaattacatttgaccgaatacctctatttgtgtgagcagtagtaattactgctattcttttattattatctttaccagtattagca------------------------------------------------------------------------------------------------

>GMCHE019-14|Haematopota|COI-5P

-acattatactttatttttggggcatgagccggaataattggaacttcattaagtattttaattcgagcagaattaggacacccaggatctttaattggtgatgaccaaatttataatgtaattgtaacagcacatgcttttgtaataattttctttatagttatacctattataattgggggatttggaaattgattagttccattaatattgggagctcctgatatagcattccctcgaataaataatataagtttttgacttttacccccatcattgactcttttattagcaagtagtatagtagaaaatggggctggtacaggttgaacagtttatccccccttatcagctgctattgcccatggaggaggatcagtagatttagcaattttttctttacatttagcaggaatttcttctattttaggagctgttaattttattactactgtaattaatatacgatcaacaggaattacatttgaccgaatacctttatttgtgtgagcagtagtaattactgctattcttttattattatctttaccagtattagca------------------------------------------------------------------------------------------------

>GMCHE026-14|Haematopota|COI-5P

----------------------gcatgagccggaataattggaacttcattaagtattttaattcgagcagaattaggacacccaggatctttaattggtgatgaccaaatttataatgtaattgtaacagcacatgcttttgtaataattttctttatagttatacctattataattgggggatttggaaattgattagttccattaatattgggagctcctgatatagcattccctcgaataaataatataagtttttgacttttacccccatcattgactcttttattagcaagtagtatagtagaaaatggggctggtacaggttgaacagtttatccccccttatcagctgctattgcccatggaggaggatcagtagatttagcaattttttctttacatttagcaggaatttcttctattttaggagctgttaattttattactactgtaattaatatacgatcaacaggaattacatttgaccgaatacctttatttgtgtgagcagtagtaattactgctattcttttattattatctttaccagtattagcaggg---------------------------------------------------------------------------------------------

>GMCHE021-14|Haematopota|COI-5P

aacattatactttatttttggggcatgagccggaataattggaacttcattaagtattttaattcgagcagaattaggacacccaggatctttaattggtgatgaccaaatttataatgtaattgtaacagcacatgcttttgtaataattttctttatagttatacctattataattgggggatttggaaattgattagttccattaatattgggagctcctgatatagcattccctcgaataaataatataagcttttgacttttacccccatcattgactcttttattagcaagtagtatagtagaaaatggggctggtacaggttgaacagtttatccccccttatcagctgctattgcccatggaggaggatcagtagatttagcaattttttctttacatttagcaggaatttcttctattttaggagctgttaattttattactactgtaattaatatacgatcaacaggaattacatttgaccgaatacctttatttgtgtgagcagtagtaattactgctattcttttattattatctttaccagtattagcaggggctattactatacttttaacagat---------------------------------------------------------------------

>GMCHM005-14|Haematopota|COI-5P

aacattatactttatttttggggcatgagccggaataattggaacttcattaagtattttaattcgagcagaattaggacacccaggatctttaattggtgatgaccaaatttataatgtaattgtaacagcacatgcttttgtaataattttctttatagttatacctattataattgggggatttggaaattgattagttccattaatattgggagctcctgatatagcattccctcgaataaataatataagtttttgacttttacccccatcattgactcttttattagcaagtagtatagtagaaaatggggctggtacaggttgaacagtttatccccccttatcagctgctattgcccatggaggaggatcagtagatttagcaattttttctttacatttagcaggaatttcttctattttaggagctgttaattttattactactgtaattaatatacgatcaacaggaattacatttgaccgaatacctttatttgtgtgagcagtagtaattactgctattcttttattattatctttaccagtattagcaggggctattactatacttttaacagatcgaaatttaaatacttcattttttgacccagcaggaggaggagatcctattttatatcaacatctattt

>GMCHE031-14|Haematopota|COI-5P

----------tttatttttggggcatgagccggaataattggaacttcattaagtattttaattcgagcagaattaggacacccaggatctttaattggtgatgaccaaatttataatgtaattgtaacagcacatgcttttgtaataattttctttatagttatacctattataattggaggatttggaaattgattagttccattaatattgggagctcctgatatagcattccctcgaataaataatataagtttttgacttttacccccatcattgactcttttattagcaagtagtatagtagaaaatggggctggtacaggttgaacagtttatccccccttatcagctgctattgcccatggaggaggatcagtagatttagcaattttttctttacatttagcaggaatttcttctattttaggagctgttaattttattactactgtaattaatatacgatcaacaggaattacatttgaccgaatacctttatttgtgtgagcagtagtaattactgctattcttttattattatctttaccagtattagcaggggctattactatacttttaacagat---------------------------------------------------------------------

>GMCHO132-14|Haematopota|COI-5P

-------------------ggggcatgagccggaataattggaacttcattaagtattttaattcgagcagaattaggacacccaggatctttaattggtgatgaccaaatttataatgtaattgtaacagcacatgcttttgtaataattttctttatagttatacctattataattgggggatttggaaattgattagttccattaatattgggggctcctgatatagcattccctcgaataaataatataagtttttgacttttacccccatcattaactcttttattagcaagtagtatagtagaaaatggggctggtacaggttgaacagtttatccccccttatcagctgctattgcccatggaggaggatcagtagatttagcaattttttctttacatttagcaggaatttcttctattttaggagctgttaattttattactactgtaattaatatacgatcaacaggaattacatttgaccgaatacctttatttgtgtgagcagtagtaattactgctattcttttattattatctttaccagtattagcaggagctattactatacttttaacagat---------------------------------------------------------------------

>GBMNB24963-20|Haematopota brevis|COI-5P|MT138909

--------------------------------------ttggtacctcattaagtatcttaattcgagctgaattaggacatccaggatctttaattggtgatgatcaaatttataatgtaattgtaacagcacatgcttttgtaataattttctttatagtaatacctattataattggaggatttggtaattgattagttccactaatattaggagcccctgatatagcatttcctcgaataaataatataagattttgacttttacctccatcattgactcttttattagcaagtagtatagtagaaaatggggctggtactggttgaacagtttaccctccattatcagcagctattgctcatggaggaggatcagtagatttagcaattttttctttacatcttgcaggaatttcttctattttaggagctgtaaattttattactactgtaattaatatacgatcaacaggtattacatttgatcgaatacctttatttgtatgagctgtagtaattactgctattcttctattattatctttaccagtattagctggagctattactatacttttaacagatcgaaatttaaatacctcattttttgacccagctggaggaggagaccctattttataccaacatt-----

>GBMND40251-21|Haematopota|COI-5P|MT083917

--------------------------------------ttggaacttcattaagtattttaattcgagctgaattagggcatccaggatctttaattggtgatgatcaaatttataatgtaattgtaacagcacatgcctttgtaataattttcttcatagtaatacctattataattgggggatttggtaactgattagttcccctaatattaggagcccccgatatagcatttccccgaataaataatataagattttgacttttacccccatcattaactctattattagcaagtagtatagtagaaaatggagccggtactggttgaacagtttacccaccactatcagcagctattgctcatggaggaggatcagtagatttagcaattttttctttacaccttgctggaatttcttctattttaggagctgtaaattttattactactgtaattaatatacgatcaacaggaattacatttgaccgaatacctttatttgtatgagcagtagtaattactgctattcttttattattatctttaccagtattagctggagctattactatacttttaacggatcggaatttaaatacatcattttttgacccagcagggggaggagaccctattttatatcaacacttattt

>GBDP26273-19|Haematopota pachycera|COI-5P|MF144205

aacattatattttatttttggggcatgagctggaataattggaacctcattaagtattttaattcgagctgaattaggacatccaggatctctaattggtgatgaccaaatctataatgtaattgtaacagcgcatgcttttgtaataattttctttatagtaatacctattataattgggggattcggaaactgactagttccactaatattaggagctcctgatatggcattccctcgaataaataatataagattttgacttttacccccatcattaactcttttattagccagtagtatagtagaaaatggggcaggtactggttgaacagtttatcccccattatcggcagctattgctcatggagggggatcagtagatttagcaattttttctttacaccttgcgggagtttcctctattttaggggctgtaaattttattaccactgttattaatatacgatcaacaggtattacatttgatcgaatacctttatttgtatgagctgtagtaattacagctattcttttattattatctttaccggtattggctgttgctattactatacttttaacagatcgaaatttaaatacttcattttttgatcctgcggggggaggagacccaattctataccaacatttattt

>GBDP26274-19|Haematopota pachycera|COI-5P|MF144206

aacattatattttatttttggggcatgagctggaataattggaacctcattaagtattttaattcgagctgaattaggacatccaggatctctaattggtgatgaccaaatctataatgtaattgtaacagcgcatgcttttgtaataattttctttatagtaatacctattataattgggggattcggaaactgactagttccactaatattaggagctcctgatatggcattccctcgaataaataatataagattttgacttttacccccatcattaactcttttattagccagtagtatagtagaaaatggggcaggtactggttgaacagtttatcccccattatcggcagctattgctcatggagggggatcagtagatttagcaattttttctttacaccttgcgggagtttcctctattttaggggctgtaaattttattaccactgttattaatatacgatcaacaggtattacatttgatcgaatacctttatttgtatgagctgtagtaattacagctattcttttattattatctttaccggtattggctggtgctattactatacttttaacagatcgaaatttaaatacttcattttttgatcctgcggggggaggagacccaattctataccaacatttattt

>GBDP26298-19|Haematopota pachycera|COI-5P|MF144230

aacattatattttatttttggggcatgagctggaataattggaacctcattaagtattttaattcgagctgaattaggacatccaggatctctaattggtgatgaccaaatctataatgtaattgtaacagcgcatgcttttgtaataattttctttatagtaatacctattataattgggggattcggaaactgactagttccactaatattaggagctcctgatatggcattccctcgaataaataatataagattttgacttttacccccatcattaactcttttattagctagtagtatagtagaaaatggggcaggtactggttgaacagtttatcccccattatcggcagctattgctcatggagggggatcagtagatttagcaattttttctttacaccttgcgggagtttcctctattttaggggctgtaaattttattaccactgttattaatatacgatcaacaggtattacatttgatcgaatacctttatttgtatgagctgtagtaattacagctattcttttattattatctttaccggtattggctggtgctattactatacttttaacagatcgaaatttaaatacttcattttttgatcctgcggggggaggagacccaattctataccaacatttattt

>GBDP26299-19|Haematopota pachycera|COI-5P|MF144231

aacattatattttatttttggggcatgagctggaataattggaacctcattaagtattttaattcgagctgaattaggacatccaggatctctaattggtgatgaccaaatctataatgtaattgtaacagcgcatgcttttgtaataattttctttatagtaatacctattataattgggggattcggaaactgactagttccactaatattaggagctcctgatatagcattccctcgaataaataatataagattttgacttttacccccatcattaactcttttattagctagtagtatagtagaaaatggggcaggtactggttgaacagtttaccccccgttatcggcagctattgctcatggagggggatcagtagatttagcaattttttctttacaccttgcgggagtttcctctattttaggagctgtaaattttattaccactgttattaatatacgatcaacaggtattacatttgatcgaatacctttatttgtatgggctgtagtaattacagctattcttttattattatctttaccagtattggctggtgctattactatacttttaacagatcgaaatttaaatacttcattttttgatcctgcggggggaggagacccaattctataccaacatttattt

>GBDP15872-15|Haematopota pachycera|COI-5P|KM243504

-------ttttttatttttggggcatgagctggaataattggaacctcactaagtattttaattcgagctgaattaggacacccggggtctttaattggtgatgaccaaatttataatgtaattgtaacagcacatgcttttgtaataattttctttatagtaatacctattataattgggggattcggaaactgactagttccactaatattaggagctcctgatatagcattccctcgaataaataatataagattttgacttttacccccatcattaactcttttattagctagtagtatagtagaaaatggggcaggtactggttgaacagtttaccccccgttatcggcagctattgctcatggagggggatcagtagatctagcaattttttctttacatcttgcaggagtttcctctattttaggagctgtaaattttattactactgttattaatatacgatcaacaggtattacgtttgatcgaatgcctttatttgtatgagctgtagtaattacagctattcttttattattatctttaccagtattggcaggtgctattactatacttttaacagatcgaaatttaaatacttcattttttgaccctgcggggggaggggacccaattctatacca----------

>GBDP26301-19|Haematopota pachycera|COI-5P|MF144233

aacattatattttatttttggggcatgagctggaataattgggacttcattaagtattttaattcgagctgaattaggacatcctgggtctttaattggtgatgaccaaatttataatgtaattgtaacagcacatgcttttgtaataattttctttatagtaatacccattataattgggggatttggaaattgattagttccattaatattaggggctcctgatatggcatttcctcgaataaataatataagattttgacttttacccccatcattgactcttttattagctagtagtatagtagaaaatggggcaggtactggttgaacagtttaccctccattatcagcagctattgctcatgggggaggatcagtagatttagcaattttttctctacatcttgcaggagtttcttctattttaggagctgtaaattttattactactgttattaatatacgatcaacaggtattacatttgaccgaatacctttatttgtatgagctgtagtaattacagctattcttttattattatctctaccagtattggctggtgctattactatacttttaacagatcgaaatttaaatacttcattttttgaccctgcaggaggaggagacccaattctatatcaacatttattt

>GBDP26300-19|Haematopota pachycera|COI-5P|MF144232

aacattatattttatttttggagcatgagctggaataattgggacttcattaagtattttaattcgagctgaattagggcaccctggatctttaattggtgatgaccaaatttataatgtaattgtaacagcacatgcttttgtaataattttctttatagtaatacctattataattggaggatttggaaattgattagttccattaatactaggagctcctgatatagcatttcctcgaataaataatataagattttgacttttacccccatcattaactcttttattagctagtagcatagtagaaaatggggcgggtactggttgaacagtttaccccccgttatcagcggctattgcccatggaggaggatcagtagatttagcaattttttctctacatcttgcgggagtttcttctattctcggagctgtaaattttattactactgttatcaatatacgatcaacaggtattacatttgaccgaatgcctttatttgtatgagctgtagtaattacagctattcttttattattatctttaccggtattagctggtgctattactatacttttaaccgatcgaaatttaaatacttcattttttgatcctgcaggaggaggagacccaattttataccaacacttattt

>GBDP26270-19|Haematopota pachycera|COI-5P|MF078332

aacattatattttatttttggagcatgagccggaataattgggacttcattaagtattttaattcgagctgaattaggacacccgggatctttaattggtgatgatcaaatttataatgtaattgtaacagcacatgcttttgtaataattttctttatagtaatacctattataattggaggatttggaaattgattagttccattaatattaggggctcctgatatagcatttcctcgaataaataatataagattttgacttttacctccttcattgactcttttattagctagtagtatagtagaaaatggggctggtactggttgaacagtttaccctccattatctgcagctattgctcatggagggggatcagtagatttagcaattttttctcttcatcttgcaggaatttcttctattttaggggctgtaaattttattactactgttattaatatacgatcaacaggtattacatttgatcgaatacctttatttgtatgagctgtagtaattacagccattttactattattatctttaccagtattagcaggagctattacaatacttttaacagatcgaaatttaaatacctcattttttgaccccgctggaggaggagaccctattctatatcaacatttattt

>GBDP26279-19|Haematopota javana|COI-5P|MF144211

aacattatattttatttttggggcatgagcaggaataattggaacttcattaagtattctaattcgagctgaattaggacatccaggatctttaattggtgatgaccaaatttataatgtaattgtaacagcacacgcttttgtaataattttctttatagtaatacctattataattggaggatttggaaattgactagtacctttaatattaggagctcctgatatagcatttcctcgaataaataatataagattttgacttttacctccatcattaactcttttattagcaagcagtatagtagaaaatggagctggtactggttgaacagtataccctcctttatcagccgctattgctcatggaggaggatcagtagatttagcaattttttctttacatctagcaggaatttgttctattctaggagctgttaattttattacaactgtaattaatatacgatcaacaggaattacatttgatcgaatacctttatttgtgtgagcagtagtaattactgctattttattattattatctttaccagtattagcgggagctattactatattattaacagatcgaaatttaaatacttcattctttgacccagcaggaggaggagatcctattttatatcaacatttattt

>GBDP26280-19|Haematopota javana|COI-5P|MF144212

aacattatattttatttttggggcatgagcaggaataattggaacttcattaagtattctaattcgagctgaattaggacatccaggatctttaattggtgatgaccaaatttataatgtaattgtaacagcacacgcttttgtaataattttctttatagtaatacctattataattggaggatttggaaattgactagtacctttaatattaggagctcctgatataacatttcctcgaataaataatataagattttgacttttacctccatcattaactcttttattagcaagcagtatagtagaaaatggagctggtactggttgaacagtataccctcctttatcagccgctattgctcatggaggaggatcagtagacttagcaattttttctttacatcttgcaggaatttgttctattctaggagctgttaattttattacaactgtaattaatatacgatcaacaggaattacatttgatcgaatacctttatttgtgtgagcagtagtaattactgctattttattattattatctttaccagtattagcgggagctattactatattattaacagatcgaaatttaaatacttcattctttgacccagcaggaggaggagatcctattttatatcaacatttattt

>GBDP26305-19|Haematopota javana|COI-5P|MF144237

aacattatattttatttttggggcatgagctggaataattggaacttcattaagtattctaattcgagctgaattaggacacccaggatctttaattggtgatgaccaaatttataatgtaattgtaacagcacacgcttttgtaataattttctttatagtaatacctattataattggaggatttggaaattgactagtacctttaatattaggagcccctgatatagcatttcctcgaataaataatataagattttgacttttacccccatcattaactcttttattagcaagtagtatagtagaaaatggagctggtactggttgaacagtataccctcctttatcagctgctattgcccatggagggggatccgtagatttagcaattttttctttacatctagcaggaatttcttctattctaggggctgttaattttattacaactgtaattaatatacgatcaacaggaattacatttgaccgaatacctttatttgtatgagcagtagtgattactgctattttattattattatctttaccagtattagcaggagctattactatattattaacagatcgaaatttaaatacttcattctttgatccagcaggaggaggagatcctattttataccaacatttattt

>GBMNB24960-20|Haematopota javana|COI-5P|MT132390

--------------------------------------ttgggacttcattaagtattttaattcgagctgaattaggacatcctggatctttaattggagatgaccaaatttataatgtaattgtaacagcacacgcttttgtaataattttctttatagtaatacctattataattggaggatttggtaattgactagtacctttaatattaggagctcctgatatagcatttcctcgaataaataatataagattttgacttttacccccctcattaactcttttattagcaagtagtatagtagaaaatggggccggtactggttgaacagtataccccccattatcagctgctattgctcatgggggaggatcagtagatttagcaattttttctttacatcttgcaggaatttcttctattttaggggctgttaattttattacaactgtaattaatatacgatcaacaggaattacatttgatcgaatacctttatttgtatgagctgtagtaattactgctattttattattattatctttaccagtattagccggagctattactatattattaacagatcgaaatttaaatacttcattttttgatccagcaggaggaggagatcctattttataccaacatttatt-

>GBDP15887-15|Haematopota latifascia|COI-5P|KM243523

--------attttatttttggggcatgagctggaataattggaacttcattaagtattttaattcgagctgaattagggcatccaggatctttaattggtgatgaccaaatttataatgtaattgtaacagcacatgcttttgtaataattttttttatagtaatgcctattataattggaggatttggaaattgattagttccattaatattaggagctcctgatatagccttccctcgaataaataatataagtttttgacttttacctccttcattaactcttttattagcaagtagtatagtagaaaatggagctggtactggttgaacagtttatccaccattatcagctgctattgctcatggtggaggatcagtagacttagcaattttttctttacatcttgcaggaatttcttctattttaggggctgttaactttattactactgtaattaatatacgatcgacaggaattacatttgatcgaatacctttatttgtatgagcagtagtaattactgccattttattattattatctttaccagtattagcaggagctattactatacttttaacagatcgaaatttaaatacttcattctttgaccctgcaggaggaggggaccctattttata-------------

>GBDP26272-19|Haematopota lata|COI-5P|MF144204

aacattatattttatttttggagcatgagctggaataattgggacttcattaagtattctaattcgagctgaattaggacatcctgggtctttaattggtgatgaccaaatttataacgtaattgtaactgcacatgcatttgtaataattttctttatagtaatacctattataattggaggatttggaaattgattagttccattaatattaggagctcctgatatagctttccctcgaataaataatataagtttttgacttttacccccatcattaactcttttattagcaagtagtatagtagaaaatggagcgggtacaggttgaacagtttaccccccattatcagctgctattgctcatggaggaggatcagtagatttagcaattttttctttacatcttgcaggaatttcttctattttaggagctgttaattttattactactgtaattaatatacgatcaacaggaattacatttgatcgaatacctctatttgtatgagcagtagtaattactgctattttactattattatctttaccagtattagcgggagctattactatacttttaactgaccgaaacttaaatacctcattttttgacccagcaggaggaggggaccctatcctatatcaacatttattt

>GBDP26290-19|Haematopota biorbis|COI-5P|MF144222

aacattatattttatttttggagcatgagctggaataattgggacttcattaagtattctaattcgagctgaattaggacatcctgggtctttaattggtgatgaccaaatttataacgtaattgtaactgcacatgcatttgtaataattttctttatagtaatacctattataattggaggatttggaaattgattagttccattaatattaggagctcctgatatagctttccctcgaataaataatataagtttttgacttttacccccatcattaactcttttattagcaagtagtatagtagaaaatggagcgggtacaggttgaacagtttaccccccattatcagctgctattgctcatggaggaggatcagtagatttagcaattttttctttacatcttgcaggaatttcttctattttaggagctgttaattttattactactgtaattaatatacgatcaacaggaattacatttgatcgaatacctctatttgtatgagcagtagtaattactgctattttactattattatctttaccagtattagcgggagctattactatacttttaactgaccgaaacttaaatacctcattttttgacccagcaggaggaggggaccctatcctatatcaacatttattt

>GBDP26291-19|Haematopota pallida|COI-5P|MF144223

aacattatattttatctttggagcatgagccggaataattggaacttcattaagtatcttaattcgagctgaattaggacatccaggatctttaattggtgatgatcaaatttataatgtaattgtaacagcacatgcctttgtaataattttctttatagtaatacctattataattggaggatttgggaattgattagtaccattaatattaggagctcctgatatagccttccctcgaataaataatataagtttttgacttttacccccttcattaactcttttattagcaagtagtatagtagaaaatggagcaggtactggttgaacagtttaccctccattatctgctgctattgcccatggaggaggatcagtagacttagcaattttttctttacatcttgcaggaatttcttctattttaggggcagttaattttattactactgtaattaatatacgatcaacaggaattacttttgatcgaatacctttatttgtatgagcagtagtaattactgctattttattattattatctttaccagtattagcaggagctattactatacttttaacagatcgaaatttaaatacttctttttttgatccagcaggaggaggagatcctattttatatcaacatttattt

>GBDP26283-19|Haematopota gracilicornis|COI-5P|MF144215

aacattatattttatttttggagcatgagccggaataattggaacctcattaagtattttaattcgagctgaattaggacacccgggatcattaattggtgatgatcaaatttataacgtaattgtaacagcacatgcttttgtaataattttctttatagtaatacctattataattggaggatttggaaattgattagttccattaatactaggagctcctgatatagcatttcctcgaataaataatataagtttttggcttttacctccatcattgactcttttattagcaagtagtatagtagaaaatggagcaggtactggttgaacagtttaccctcctctatcagcagctattgcccatggtggtggttcagtagatttagcaattttttctttacatcttgcaggaatttcttctattttaggagctgtaaattttattactactgtaattaacatacgttcaacaggaattacattcgaccggatacccctatttgtatgagcggtagtgattactgctattctcttattactatctttaccagtattggctggtgcaattactatacttttaacagatcgaaatttaaatacttcattttttgacccagcaggaggaggagatcctatcttataccaacatttattt

>GBDP26302-19|Haematopota gracilicornis|COI-5P|MF144234

aacattatattttatttttggagcatgagccggaataattggaacctcattaagtattttaattcgagctgaattaggacacccgggatcattaattggtgatgatcaaatttataacgtaattgtaacagcacatgcttttgtaataattttctttatagtaatacctattataattggaggatttggaaattgattagttccattaatactaggagctcctgatatagcatttcctcgaataaataatataagtttttggcttttacctccatcattgactcttttattagcaagtagtatagtagaaaatggagcaggtactggttgaacagtttaccctcctctatcagcagctattgcccatggtggtggttcagtagatttagcaattttttctttacatcttgcaggaatttcttctattttaggagctgtaaattttattactactgtaattaacatacgttcaacaggaattacattcgaccggatacccctatttgtatgagcggtagtgattactgctattctcttattactatctttaccagtattggctggtgcaattactatacttttaacagatcgaaatttaaatacttcattttttgacccagcaggaggaggagatcctatcttataccaacatttattt

>GBDP26284-19|Haematopota gracilicornis|COI-5P|MF144216

aacattatattttatttttggagcatgagccggaataattggaacctcattaagtattttaattcgagctgaattaggacacccgggatcattaattggtgatgatcaaatttataacgtaattgtaacagcacatgcttttgtaataattttctttatagtaatacctattataattggaggatttggaaattgattagttccattaatactaggagctcctgatatagcatttcctcgaataaataatataagtttttgacttttacctccatcattgactcttttattagcaagtagtatagtagaaaatggagcaggtactggttgaacagtttaccctcctctatcagcagctattgctcatggtggtggttcagtagatttagcaattttttctttacatcttgcaggaatttcttctattttaggagctgtaaattttattactactgtaattaacatacgttcaacaggaattacattcgaccggatacccctatttgtatgagcggtagtgattactgctattcttttattactatctttaccagtattggctggtgcaattactatacttttaacagatcgaaatttaaatacttcattttttgacccagcaggaggaggagatcctatcttataccaacatttattt

>AMTPD3586-15|Haematopota pluvialis|COI-5P

-------------------------------------------------------attttaattcgagctgaattaggacacccgggatctttaattggtgatgaccaaatttataatgtaattgtaacagcacatgcttttgtaataattttctttatagttatacctattataattggaggatttggaaattgattagtaccattaatattaggagctcctgatatagcatttcctcgaataaataatataagtttttgacttttacccccatcattgacccttttattagcaagtagtatagtagaaaatggggctggaactggttgaacagtttaccccccattatcagctgcaattgcccacggagggggatctgtagatttagcaattttttctttacatcttgcaggaatttcttctatcttaggggcagttaattttattactactgtaattaatatacgatcaacaggaattacatttgatcgaatacctttatttgtttgagcagtagtaattacagctattcttttattactatctttacctgtattagcaggtgcaattactatacttttaacagat---------------------------------------------------------------------

>DIPFI011-12|Haematopota pluvialis|COI-5P

aacattatactttatttttggggcatgagccggaataattggaacctcgctaagtattttaattcgagctgaattaggacacccgggatctttaattagtgatgaccaaatttataatgtaattgtaacagcacatgcttttgtaataattttctttatagttatacctattataattggaggatttggaaattgattagtaccattaatattaggagctcctgatatagcatttcctcgaataaataatataagtttttgacttttacccccatcattgacccttttattagcaagtagtatagtagaaaatggggctggaactggttgaacagtttaccccccattatcagctgcaattgcccacggaggaggatctgtagatttagcaattttttctttacatcttgcaggaatttcttctattttaggggcagttaattttattactactgtaattaatatacgatcaacaggaattacatttgatcgaatacctttatttgtttgagcagtagtaattacagctattcttttattattatctttacctgtattagcgggtgcaattactatacttttaacagatcgaaatttaaatacatccttttttgaccctgctggaggaggagacccaattttatatcaacatttattt

>DIPFI010-12|Haematopota pluvialis|COI-5P

aacattatactttatttttggggcatgagccggaataattggaacctcgctaagtattttaattcgagctgaattaggacacccgggatctttaattggtgatgaccaaatttataatgtaattgtaacagcacatgcttttgtaataattttctttatagttatacctattataattggaggatttggaaattgattagtaccattaatattaggagctcctgatatagcatttcctcgaataaataatataagtttttgacttttacccccatcattgacccttttattagcaagtagtatagtagaaaatggggctggaactggttgaacagtttaccccccattatcagctgcaattgcccacggaggaggatctgtagatttagcaattttttctttacatcttgcaggaatttcttctattttaggggcagttaattttattactactgtaattaatatacgatcaacaggaattacatttgatcgaatacctttatttgtttgagcagtagtaattacagctattcttttattattatctttacctgtattagcgggtgcaattactatacttttaacagatcgaaatttaaatacatccttttttgaccctgctggaggaggagacccaattttatatcaacatttattt

>FIDIP325-11|Haematopota pluvialis|COI-5P|Pending (#7798)

aacattatactttatttttggggcatgagccggaataattggaacctcgctaagtattttaattcgagctgaattaggacacccgggatctttaattggtgatgaccaaatttataatgtaattgtaacagcacatgcttttgtaataattttctttatagttatacctattataattggaggatttggaaattgattagtaccattaatattaggagctcctgatatagcatttcctcgaataaataatataagtttttgacttttacccccatcattgacccttttattagcaagtagtatagtagaaaatggggctggaactggttgaacagtttaccccccattatcagctgcaattgcccacggaggaggatctgtagatttagcaattttttctttacatcttgcaggaatttcttctattttaggggcagttaattttattactactgtaattaatatacgatcaacaggaattacatttgatcgaatacctttatttgtttgagcagtagtaattacagctattcttttattattatctttacctgtattagcgggtgcaattactatacttttaacagatcgaaatttaaatacatccttttttgaccctgctggaggaggagacccaattttatatcaacatttattt

>TIPNO311-19|Haematopota sp.|COI-5P

aacattatactttatttttggggcatgagccggaataattggaacctcgctaagtattttaattcgagctgaattaggacacccgggatctttaattggtgatgaccaaatttataatgtaattgtaacagcacatgcttttgtaataattttctttatagttatacctattataattggaggatttggaaattgattagtaccattaatattaggagctcctgatatagcatttcctcgaataaataatataagtttttgacttttacccccatcattgacccttttattagcaagtagtatagtagaaaatggggctggaactggttgaacagtttaccccccattatcagctgcaattgcccacggaggaggatctgtagatttagcaattttttctttacatcttgcaggaatttcttctattttaggggcagttaattttattactactgtaattaatatacgatcaacaggaattacatttgatcgaatacctttatttgtttgagcagtagtaattacagctattcttttattattatctttacctgtattagcaggtgcaattactatacttttaacagatcgaaatttaaatacatccttttttgaccctgctggaggaggagacccaattttatatcaacatttattt

>FIDIP2662-12|Haematopota pluvialis|COI-5P|Pending (#7799)

aacattatactttatttttggggcatgagccggaataattggaacctcgctaagtattttaattcgagctgaattaggacacccgggatctttaattggtgatgaccaaatttataatgtaattgtaacagcacatgcttttgtaataattttctttatagttatacctattataattggaggatttggaaattgattagtaccattaatattaggagctcctgatatagcatttcctcgaataaataatataagtttttgacttttacccccatcattgacccttttattagcaagtagtatagtagaaaatggggctggaactggttgaacagtttaccccccattatcagctgcaattgcccacggagggggatctgtagatttagcaattttttctttacatcttgcaggaatttcttctattttaggggcagttaattttattactactgtaattaatatacgatcaacaggaattacatttgatcgaatacctttatttgtttgagcagtagtaattacagctattcttttattattatctttacctgtattagcgggtgcaattactatacttttaacagatcgaaatttaaatacatccttttttgaccctgctggaggaggagacccaattttatatcaacatttattt

>FIDIP3315-12|Haematopota pluvialis|COI-5P|Pending (#7799)

aacattatactttatttttggggcatgagccggaataattggaacctcgctaagtattttaattcgagctgaattaggacacccgggatctttaattggtgatgaccaaatttataatgtaattgtaacagcacatgcttttgtaataattttctttatagttatacctattataattggaggatttggaaattgattagtaccattaatattaggagctcctgatatagcatttcctcgaataaataatataagtttttgacttttacccccatcattgacccttttattagcaagtagtatagtagaaaatggggctggaactggttgaacagtttaccccccattatcagctgcaattgcccacggagggggatctgtagatttagcaattttttctttacatcttgcaggaatttcttctattttaggggcagttaattttattactactgtaattaatatacgatcaacaggaattacatttgatcgaatacctttatttgtttgagcagtagtaattacagctattcttttattattatctttacctgtattagcaggtgcaattactatacttttaacagatcgaaatttaaatacatccttttttgaccctgctggaggaggagacccaattttatatcaacatttattt

>TIPNO304-19|Haematopota cf. pluvialis|COI-5P

aacattatactttatttttggggcatgagccggaataattggaacctcgctaagtattttaattcgagctgaattaggacacccgggatctttaattggtgatgaccaaatttataatgtaattgtaacagcacatgcttttgtaataattttctttatagttatacctattataattggaggatttggaaattgattagtaccattaatattaggagctcctgatatagcatttcctcgaataaataatataagtttttgacttttacctccatcattgacccttttattagcaagtagtatagtagaaaatggggctggaactggttgaacagtttaccccccattatcagctgcaattgcccacggaggaggatctgtagatttagcaattttttctttacatcttgcaggaatttcttctattttaggggcagttaattttattactactgtaattaatatacgatcaacaggaattacatttgatcgaatacctttatttgtttgagcagtagtaattacagctattcttttattattatctttacctgtattagcgggtgcaattactatacttttaacagatcgaaatttaaatacatccttttttgaccctgctggaggaggagacccaattttatatcaacatttattt

>TIPNO379-19|Haematopota cf. crassicornis|COI-5P

aacattatactttatttttggggcatgagccggaataattggaacctcgctaagtattttaattcgagctgaattaggacacccgggatctttaattggtgatgaccaaatttataatgtaattgtaacagcacatgcttttgtaataattttctttatagttatacctattataattggaggatttggaaattgattagtaccattaatattaggagctcctgatatagcatttcctcgaataaataatataagtttttgacttttacccccatcattgacccttttattagtaagtagtatagtagaaaatggggctggaactggttgaacagtttaccccccattatcagctgcaattgcccacggaggaggatctgtagatttagcaattttttctttacatcttgcaggaatttcttctattttaggggcagttaattttattactactgtaattaatatacgatcaacaggaattacatttgatcgaatacctttatttgtttgagcagtagtaattacagctattcttttattattatctttacctgtattagcgggtgcaattactatacttttaacagatcgaaatttaaatacatccttttttgaccctgctggaggaggagacccaattttatatcaacatttattt

>CROBB316_SK-32_Haematopota pluvialis

aacattatactttatttttggggcatgagccggaataattggaacctcgctaagtattttaattcgagctgaattaggacacccgggatctttaattggtgatgaccaaatttataatgtaattgtaacagcacatgcttttgtaataattttctttatagttatacctattataattgggggatttggaaattgattagtaccattaatattaggagctcctgatatagcatttcctcgaataaataatataagtttttgacttttacccccatcattgacccttttattagcaagtagtatagtagaaaatggggctggaactggttgaacagtttaccccccattatcagctgcaattgcccatggagggggatctgtagatttagcaattttttctttacatcttgcaggaatttcttctattttaggggcagttaattttattactactgtaattaatatacgatcaacaggaattacatttgatcgaatacctttatttgtttgagcagtagtaattacagctattcttttattactatctttacctgtattagcaggtgcaattactatacttttaacagatcgaaatttaaatacatccttttttgaccctgctggaggaggggacccaattttatatcaacatttattt

>FIDIP3316-12|Haematopota pluvialis|COI-5P|Pending (#7799)

--------------------------------------------------taagtattttaattcgagctgaattaggacacccgggatctttaattggtgatgaccaaatttataatgtaattgtaacagcacatgcttttgtaataattttctttatagttatacctattataattggaggatttggaaattgattagtaccattaatattaggagctcctgatatagcatttcctcgaataaataatataagtttttgacttttacccccatcattgacccttttattagcaagtagtatagtagaaaatggggctggaactggttgaacagtttaccccccattatcagctgcaattgcccacggaggnggatctgtagatttagcaattttttctttacatcttgcaggaatttcttctattttaggggcagttaattttattactactgtaattaatatacgatcaacaggaattacatttgatcgaatacctttatttgtttgagcagtagtaattacagctattcttttattantatctttacctgtattagcaggtgcaattactatacttttaacagatcgaaatttaaatacatccttttttgacc-----------------------------------------

>GBDP13565-13|Haematopota pluvialis|COI-5P|KC192969

aacattatactttatttttggggcatgagccggaataattggaacctcgctaagtattttaattcgagctgaattaggacacccgggatctttaattggtgatgaccaaatttataatgtaattgtaacagcacatgcttttgtaataattttctttatagttatacctattataattggaggatttggaaattgattagtaccattaatattaggagctcctgatatagcatttcctcgaataaataatataagtttttgacttttacccccatcattgacccttttattagcaagtagtatagtagaaaatggggctggaactggttgaacagtttaccccccattatcagctgcaattgcccatggagggggatctgtagatttagcaattttttctttacatcttgcaggaatttcttctatgttaggggcagttaattttattactactgtaattaatatacgatcaacaggaattacatttgatcgaatacctttatttgtttgagcagtagtaattacagctattcttttattactatctttacctgtattagcaggtgcaattactatacttttaacagatcgaaatttaaatacatccttttttgaccctgctggaggaggggacccaattttatatcaacatttattt

>CROBB317_SK-33_Haematopota subcylindrica

aacattatactttatttttggagcatgagccggaataattggaacctcattaagtattttaattcgagctgaattaggacacccggggtctttaattggagatgaccaaatttataatgtaattgtaacagcacatgcttttgtaataattttctttatagttatacctattataattggaggatttggaaattgattagtcccattaatattaggggctcctgatatagcatttcctcgaataaataatataagtttttgacttttacctccatcattgactcttttattagcaagtagtatagtagaaaatggggctggaactggttgaacagtttacccaccattatcagctgcaattgcccatggaggaggatctgtagatttagcaattttttctttacatcttgcaggaatttcttctattttaggggcagttaattttattactactgtaattaatatacgatcaacaggaattacatttgatcgaatacctttatttgtttgagcagtagtaattacagctattcttttattattatctttacctgtattagctggtgcaattactatacttttaacagatcgaaatttaaatacatccttttttgatcctgctggaggaggagatccaattctatatcaacatttattt

>AMTPD3601-15|Haematopota pluvialis|COI-5P

----------------tttggggcatgagccggaataat---nnnctcgctaagtattttaattcgagctgaattaggacacccgggatctttaattggcgatgaccaaatttataatgtaattgtaacagcacatgcttttgtaataattttctttatagttatacctattataattggaggatttggaaattgattagtaccattaatattaggagctcctgatatagcatttcctcgaataaataatataagtttttgacttttacccccatcattggcccttttattagcaagtagtatagtagaaaatggggctggaactggttgaacagtttaccccccattatcagctgcaattgcccacggaggaggatctgtagatttagcaattttttctttacaccttgcaggaatttcttctattttaggggcagttaattttattactactgtaattaatatacgatcaacaggaattacatttgatcgaatacctttatttgtttgagcagtagtaattacagctattcttttattattatctttacctgtattagcaggt---------------------------------------------------------------------------------------------

>GMGMK860-14|Haematopota pluvialis|COI-5P

-acattatactttatttttggggcatgagccggaataattggaacctcgctaagtattttaattcgagctgaattaggacacccgggatctttaattggtgatgaccaaatttataatgtaattgtaacagcacatgcttttgtaataattttctttatagttatacctattataattggaggatttggaaattgattagtaccattaatattaggagctcctgatatagcatttcctcgaataaataatataagtttttgactcttacccccatcattgacccttttattagcaagtagtatagtagaaaatggggctggaactggttgaacagtttaccccccattatcagctgcaattgcccacggagggggatctgtagatttagcaattttttctttacatcttgcaggaatttcttctattttaggggcagttaattttattactactgtaattaatatacgatcaacaggaattacatttgatcgaatacctttatttgtttgagcagtagtaattacagctattcttttattattatctttacctgtattagcgggt---------------------------------------------------------------------------------------------

>CROBB320_SK-36_Haematopota scutellata

aacattatattttatttttggggcatgagccggaataattggaacttcattaagtattttaattcgagctgaattaggacacccaggatctttaattggagatgaccaaatttataatgtaattgtaacagcacatgcttttgtaataattttctttatagttatacctattataattggaggatttggaaattgattagtcccattaatattaggagcccctgatatagcatttcctcgaataaataatataagtttttgacttttacccccatcattgactcttttattagcaagtagtatagtagaaaatggggctggaactggttgaacagtttatccaccattatcagctgcaattgcccatggaggaggatcagtagatctagcaattttctctttacatcttgcaggaatttcttctattttaggagcagttaattttattactactgtaattaatatacgatcaacaggaattacatttgatcgaatacctttatttgtatgagctgtagtaattacagctattctcttattattatctttaccagtattagctggagctattactatacttttaacagaccgaaatctaaatacatctttttttgacccggctggaggaggagacccaattttatatcaacatttattt

>IBIDP166-19|Haematopota ocelligera|COI-5P|MN868869

aacattatattttatttttggagcatgagccggaataattggaacttcattaagtattttaattcgagcagaattaggacacccaggatctttaattggagatgaccaaatttataatgtaattgtaactgcacatgcttttgtaataattttctttatagttatacctattataattggaggatttggaaattgattagtcccattaatattaggagcccctgatatagcatttcctcgaataaataatataagtttttgacttttacctccatcattaactcttttattagcaagttctatagtagaaaacggagctggaactggttgaacagtataccccccattatcagctgcaattgcccatggaggtggatcagttgatttagcaattttttctttacatcttgcaggaatttcttctattttaggagcagttaattttattactactgtaattaatatacgatcaacaggaattacatttgaccgaataccattatttgtgtgagctgtagtaattacagctattcttttattattatctttaccagtattagctggtgctattactatacttttaacagatcgaaatttaaatacatctttttttgaccctgctggaggaggagaccctattttataccaacatttattt

>GBDP26268-19|Haematopota pandazisi|COI-5P|LT903698

----------------------------------------------------------------------------------ccagggtctttaattggagatgatcaaatttataatgtaattgtaacagcgcatgcttttgtaataattttctttatagttatacctattataattggagggtttggaaattgattagttccattaatattaggagctcctgatatagcctttcctcgaataaataatataagtttttgacttttacccccttcattgacccttttattagcaagtagtatagtagaaaatggagctggaactggttgaacagtttaccccccattatcagctgcaattgctcatgggggaggatcagtagatttagcaattttttctttacaccttgcaggaatttcgtctattcttggagcagttaattttattactactgtaattaatatacgatcaacaggaattacctttgatcgaatacctttatttgtatgagcagtagtaattacagctattcttttattattatctttacctgtattagcaggagcaatcactatactttta---------------------------------------------------------------------------

>GBDP26267-19|Haematopota pandazisi|COI-5P|LT903697

----------------------------------------------------------------------------------ccagggtctttaattggagatgatcaaatttataatgtaattgtaacagcgcatgcttttgtaataattttctttatagttatacctattataattggagggtttggaaattgattagttccattaatattaggagctcctgatatagcctttcctcgaataaataatataagtttttgacttttacccccttcattgacccttttattagcaagtagtatagtagaaaatggagctggaactggttgaacagtttaccccccattatcagctgcaattgctcatgggggaggatcagtagatttagcaattttttctttacaccttgcaggaatttcgtctattcttggagcagttaattttattactactgtaattaatatacgatcaacaggaattacctttgatcgaatacctttatttgtatgagcagtagtaattacagctattcttttattattatctttacctgtattagcgggagcaatcactatactttta---------------------------------------------------------------------------

>GBDP26269-19|Haematopota pandazisi|COI-5P|LT903699

----------------------------------------------------------------------------------cccgggtctttaattggagatgatcaaatttataatgtaattgtaacagcgcatgcttttgtaataattttctttatagttatacctattataattggagggtttggaaattgattagttccattaatattaggagctcctgatatagcctttcctcgaataaataatataagtttttgacttttacccccttcattgacccttttattagcaagtagtatagtagaaaatggagctggaactggttgaacagtttaccccccattatcagctgcaattgctcatgggggaggatcagtagatttagcaattttttctttacaccttgcaggaatttcgtctattcttggagcagttaattttattactactgtaattaatatacgatcaacaggaattacctttgatcgaatacctttatttgtatgagcagtagtaattacagctattcttttattattatctttacctgtattagcgggagcaatcactatactttta---------------------------------------------------------------------------

>GMGMB035-14|Haematopota italica|COI-5P

----------------------------------------------------------------------------------ccaggatctttaattggagatgaccaaatttataatgtaattgtaacagcacatgcttttgtaataattttctttatagttatacctattataattggaggatttggaaattgattagtnccattaatattaggagctcctgatatagcattccctcgaataaataatataagtttttgacttttacccccttcattgactcttttattagcaagtagtatagtagaaaatggggctggaactggttgaacagtttaccccccattatcagctgcaattgcncatggaggaggatcagtagatttagcaattttttctttacacctngcaggaatttcttctattcttggagcagttaattttattactactgtaattaatatacgatcaacaggaattacntttgatcgaatacctttatttgtatgagcagtagtaattacagctattcttttattattatctttacccgtattngcaggagcaatcactatactttta---------------------------------------------------------------------------

>CROBB319_SK-35_Haematopota italica

aacattatattttatttttggtgcatgagccggaataattggaacctccttaagtattctaattcgagctgaattaggacatccaggatctttaattggagatgaccaaatttataatgtaattgtaacagcacatgcttttgtaataattttctttatagttatacctattataattggaggatttggaaattgattagttccattaatattaggagctcctgatatagcattccctcgaataaataatataagtttttgacttttacccccttcattgactcttttattagcaagtagtatagtagaaaatggggctggaactggttgaacagtttaccccccattatcagctgcaattgcccatggaggaggatcagtagatttagcaattttttctttacaccttgcaggaatttcttctattcttggagcagttaattttattactactgtaattaatatacgatcaacaggaattacctttgatcgaatacctttatttgtatgagcagtagtaattacagctattcttttattattatctttacccgtattagcaggagcaatcactatacttttaacagaccgaaatttaaatacttctttttttgaccctgctggaggaggagaccctattttataccaacatctattt

>CROBB318_SK-34_Haematopota pandazisi

aacattatattttatttttggggcatgagccggaataatcggaacttcgttgagtattctaattcgagctgaattaggacatccaggatctttaattggagatgaccaaatttataatgtaattgtaacagcacatgcttttgtaataattttctttatagttatacctattataattggaggatttggaaattgattagttccattaatactaggagctcctgatatagcattccctcgaataaataatataagtttttgacttttacctccttcattgacccttttattagcaagtagtatagtagaaaatggagctgggactggttgaacagtttacccaccattatcggctgcaattgcccatggaggaggatcagtagatttagcaattttttctttacaccttgccggaatttcttctattcttggggcagttaactttattactactgtaattaatatacgatcaacagggattacttttgaccgaatacctctatttgtttgagcagtagtaattactgctattcttttattattatctttaccagtattagctggagcaattactatgcttttaacagatcgaaatttaaatacctccttttttgaccctgctggaggaggagaccctattttatatcaacatctattt

>GBMNC8971-20|Haematopota sp. EMU57|COI-5P|MT231188

aacattatattttatttttggggcatgggctggaataattggaacttcattaagtatcttaattcgagctgaattaggtcatccaggatctttaattggagatgaccaaatttataatgtaattgtaacagcacatgcatttgtaataattttcttcatagttatacctattataattggtggatttggaaattgattagtcccattaatattaggggctcctgatatagcatttcctcgtataaataatataagtttttgacttttacccccatcattaactcttttattaacaagtagtatagtagaaaatggggctggaactggttgaacagtttacccgccattatctgctgcaattgctcatggaggaggatcagtagatttagcaattttttctttacatcttgcaggaatttcttcaattttaggggctgttaattttattactactgtaattaatatacgatcaacaggaattacatttgatcgaatacctttatttgtatgagctgtagtaattacagctattcttttattattatctttaccagtattagctggtgctattactatacttctaacagaccgaaatctaaatacttctttttttgaccctgccggagggggtgatcctatcttatatcaacatttattt

>KDIPT2466-13|Haematopota fenestralis|COI-5P|KX946525

aacattatattttatttttggggcctgagctggaataattggaacttcattaagtatcttaattcgagctgaattaggtcatccagggtctttaattggtgatgaccaaatttataatgtaattgtaacagcacatgcatttgtaataattttctttatagttatacctattataattggtggatttggaaattgattagttccattaatattaggggctcctgatatagcatttcctcgaataaataatataagattttgacttttacctccatcattaactcttttattagcaagtagtatagtagaaaatggagccggaactggttgaacagtttatccaccattatcagctgcaattgctcatggagggggatcagttgatttagcaattttttctttacatcttgcaggaatttcctcaattttaggagctgttaattttattactactgtaattaatatacgatcaacaggaattacatttgatcgaatacctttatttgtatgagctgtagtaattacagctattcttttattgttatctttaccagtattagctggtgctattactatacttttaacagatcgaaacttaaatacttctttttttgatcctgcaggaggaggagatcctattttatatcaacatttattt

>KDIPT2491-13|Haematopota fenestralis|COI-5P|KX946528

aacattatattttatttttggggcctgagctggaataattggaacttcattaagtatcttaattcgagctgaattaggtcatccagggtctttaattggtgatgaccaaatttataatgtaattgtaacagcacatgcatttgtaataattttctttatagttatacctattataattggtggatttggaaattgattagttccattaatattaggggctcctgatatagcatttcctcgaataaataatataagattttgacttttacctccatcattaactcttttattagcaagtagtatagtagaaaatggagccggaactggttgaacagtttatccaccattatcagctgcaattgctcatggagggggatcagttgatttagcaattttttctttacatcttgcaggaatttcctcaattttaggagctgttaattttattactactgtaattaatatacgatcaacaggaattacatttgatcgaatacctttatttgtatgagctgtagtaattacagctattcttttattgttatctttaccagtattagctggtgctattactatacttttaacagatcgaaacttaaatacttcttttttcgatcctgcaggaggaggagatcctattttatatcaacatttattt

>KDIPT2468-13|Haematopota fenestralis|COI-5P|KX946527

aacattatattttatttttggggcctgagctggaataattggaacttcattaagtatcttaattcgagctgaattaggtcatccaggatctttaattggtgatgaccaaatttataatgtaattgtaacagcacatgcatttgtaataattttctttatagttatacctattataattggtggatttgggaattgattagttccattaatattaggggctcctgatatagcatttcctcgaataaataatataagattttgacttttacctccatcattaactcttttattagcaagtagtatagtagaaaatggagccggaactggttgaacagtttatccaccattatcagctgcaattgctcatggaggaggatcagttgatttagcaattttttctttacatcttgcaggaatttcctcaattttaggagctgttaattttattactactgtaattaatatacgatcaacaggaattacatttgatcgaatacctttatttgtatgagctgtagtaattacagctattcttttattgttatctttaccagtattagctggtgctattactatacttttaacagatcgaaacttaaatacttcttttttcgatcctgcaggaggaggagatcctattttatatcaacatttattt

>KDIPT2492-13|Haematopota fenestralis|COI-5P|KX946524

aacattatattttatttttggggcctgagctggaataattggaacttcattaagtatcttaattcgagctgaattaggtcatccaggatctttaattggtgatgaccaaatttataatgtaattgtaacagcacatgcatttgtaataattttctttatagttatacctattataattggtggatttgggaattgattagttccattaatattaggggctcctgatatagcatttcctcgaataaataatataagattttgacttttacctccatcattaactcttttattagcaagtagtatagtagaaaatggagccggaactggttgaacagtttatccaccattatcagctgcaattgctcatggaggaggatcagttgatttagcaattttttctttacatcttgcaggaatttcctcaattttaggagctgttaattttattactactgtaattaatatacgatcaacaggaattacatttgatcgaatacctttatttgtatgagctgtagtaattacagctattcttttattgttatctttaccagtattagctggtgctattactatacttttaacagatcgaaacttaaatacttcttttttcgatcctgcaggaggaggagatcccattttatatcaacatttattt

>KDIPT2469-13|Haematopota fenestralis|COI-5P|KX946526

aacattatattttatttttggggcctgagctggaataattggaacttcattaagtatcttaattcgagctgaattaggtcatccaggatctttaattggtgatgaccaaatttataatgtaattgtaacagcacatgcatttgtaataattttctttatagttatacctattataattggtggatttggaaattgattagttccattaatattaggggctcctgatatagcatttcctcgaataaataatataagattttgacttttacctccatcattaactcttttattagcaagtagtatagtagaaaatggggccggaactggttgaacagtttatccaccattatcagctgcaattgctcatggaggaggatcagttgatttagcaattttttctttacatcttgcaggaatttcctcaattttaggagctgttaattttattactactgtaattaatatacgatcaacaggaattacatttgatcgaatacctttatttgtatgagctgtagtaattacagctattcttttattgttatctttaccagtattagctggtgctattactatacttttaacagatcgaaacttaaatacttcttttttcgatcctgcaggaggaggag--------------------------

>GBDP27947-19|Haematopota pluvialis|COI-5P|MF706417

----------------------------------------------------------------------------------------------------------------------------------------------------------------------------------------------------------------------------------------ttcccacgaataaataatataagtttttgacttttacctccatcattgacccttttattagcaagtagtatagtagaaaatggggctggaactggttgaacagtttaccccccattatcagctgcaattgcccacggaggaggatctgtagatttagcaattttttctttacatcttgcaggaatttcttctattttaggggcagttaattttattactactgtaattaatatacgatcaacaggaattacatttgatcgaatacctttatttgtttgagcagtagtaattacagctattcttttattattatctttacctgtattagcgggtgcaattactatacttttaacagatcgaaatttaaatacatccttttttgaccctgctggaggagga---------------------------

>KDIPT2467-13|Haematopota duttoni|COI-5P|KX946520

aacattatattttattttaggggcatgagccggaataattggaacttcattaagtattttaattcgagctgaattaggtcatcctggatccttaattggtgatgatcaaatttataatgtaattgtaacagcacatgcatttgtaataattttctttatagttatacctattataattggaggatttggaaattgattagttccattaatacttggagctcctgatatagcatttcctcgaataaataatataagtttttgacttttacccccctcattaactcttttattagcaagtagtatagtagaaaatggggctgggactggttgaacagtttatcctccattatctgctgcaattgcccatggagggggatcagtagatttagcaattttttctttacatcttgcaggaatttcttcaattttaggagctgttaattttattactactgtaattaacatacgatcaacaggaattacatttgatcgaatacctttatttgtatgagctgtagtaattacagctattctattattattatctttaccagtattggctggagctattactatacttttaacagatcgaaatctaaatacctctttttttgatccagcaggagggggagatccaattttataccaacatttattt

>KDIPT2439-13|Haematopota duttoni|COI-5P|KX946523

aacattatattttattttaggggcatgagccggaataattggaacttcattaagtattttaattcgagctgaattaggtcatcctggatccttaattggtgatgatcaaatttataatgtaattgtaacagcacatgcatttgtaataattttctttatagttatacctattataattggaggatttggaaattgattagttccattaatacttggagctcctgatatagcatttcctcgaataaataatataagtttttgacttttacccccctcattaactcttttattagcaagtagtatagtagaaaatggggctgggactggttgaacagtttatcctccattatctgctgcaattgcccatggagggggatcagtagatttagcaattttttctttacatcttgcaggaatttcttcaattttaggagctgttaattttattactactgtaattaacatacgatcaacaggaattacatttgatcgaatacctttatttgtatgagctgtagtaattacagctattctattattattatctttaccagtattggctggagctattactatacttttaacagatcgaaatctaaatacctctttttttgatccagcaggagggggagatccaattttataccaacatttattt

>KDIPT2435-13|Haematopota duttoni|COI-5P|KX946522

aacattatattttattttaggagcatgagccggaataattggaacttcattaagtattttaattcgagctgaattaggtcatcctggatccttaattggtgatgatcaaatttataatgtaattgtaacagcacatgcatttgtaataattttctttatagttatacccattataattggaggatttggaaattgattagttccattaatacttggagctcctgatatagcatttcctcgaataaataatataagtttttgacttttacccccctcattaactcttttattagcaagtagtatagtagaaaatggagctgggaccggttgaacagtttatcctccattatctgctgcaattgctcatggagggggatcagtagatttagcaattttttctttacatcttgcaggaatttcttcaattttaggagctgttaattttattactactgtaattaacatacgatcaacaggaattacatttgatcgaatacctttatttgtatgagctgtagtaattacagctattctattattattatctttaccagtattggctggagctattactatacttttaacagatcgaaatctaaatacctctttttttgatccagcgggagggggagatccaattttataccaacatttattt

>KDIPT2438-13|Haematopota duttoni|COI-5P|KX946521

aacattatattttattttaggggcatgagccggaataattggaacttcattaagtattttaattcgagctgaattaggtcatcctggatccttaattggtgatgatcaaatttataatgtaattgtaacagcacatgcatttgtaataattttctttatagttatacctattataattggaggatttggaaattgattagttccattaatacttggagctcctgatatagcatttcctcgaataaataatataagtttttgacttttacccccctcattaactcttttattagcaagtagtatagtagaaaatggagctgggaccggttgaacagtttatcctccattatctgctgcaattgctcatggagggggatcagtagatttagcaattttttctttacaccttgcaggaatttcttcaattttaggagctgttaattttattactactgtaattaacatacgatcaacaggaattacatttgatcgaatacctttatttgtatgagctgtagtaattacagctattctattattattatctttaccagtattggctggagctattactatacttttaacagatcgaaatctaaatacctctttttttgatccagcgggagggg-----------------------------

>CROBB326_SK-42_Haematopota grandis

aacattatactttatttttggggcatgagccggaataattggaacttccttaagtattctaattcgagctgaattaggacatccaggatctttaattggagatgaccaaatttataatgtaattgtaacagcacatgcttttgtaataattttctttatagttatacctattataattggaggatttggaaattgattagttccattaatattaggagctcctgatatagcatttcctcgaataaataatataagtttttgacttttaccaccatcattgactcttttattagcaagtagtatagtggaaaacggagctggaactggttgaacagtttacccaccattatcagctgcaatcgctcatggaggaggatctgtagatttagcaattttttcacttcacttagcaggaatttcttctattttaggagcagttaattttattactactgttattaatatacgatcaacaggaattacatttgaccgaatacctttatttgtatgagccgtagtaattactgctattttattattattatccttaccagtattagctggtgcaattactatacttttaacagatcgaaatttaaatacttcattctttgatcctgctggaggaggagacccaattttataccaacatttattt

>GBDP26307-19|Haematopota whartoni|COI-5P|MF144239

aactttatattttatttttggagcatgagccggaataattggaacctcattgagtattttaattcgagctgaattaggacatccaggatcattaattggtgatgaccaaatttataatgtaattgtaacagctcatgcatttgtaataattttctttatagtaatacccattataattggaggatttggtaattgattagttccattaatattaggagctcctgatatagcatttcctcgaataaataatataagtttttgacttttacctccttcattaactcttttattagctagtagtatagtagaaaatggagctggaactggatgaacagtttatcctcccttatctgctgcaattgctcatggaggaggatctgttgatttagctattttttctttacaccttgcaggaatttcttctattttaggagctgttaattttattactactgtaattaatatacgatcaacaggaattacttttgatcgaatacctttatttgtgtgagctgtagtaattacagctattcttttattattatcattaccagtactagctggagctattactatacttttaacagatcgaaacctaaatacttctttttttgacccagcaggaggaggagatccaattttataccaacatttattt

>GBDP26288-19|Haematopota whartoni|COI-5P|MF144220

aactttatattttatttttggagcatgagccggaataattggaacctcattgagtattttaattcgagctgaattaggacatccaggatcattaattggtgatgaccaaatttataatgtaattgtaacagctcatgcatttgtaataattttctttatagtaatacccattataattggaggatttggtaattgattagttccattaatattaggagctcctgatatagcatttcctcgaataaataatataagtttttgacttttacctccttcattaactcttttattagctagtagtatagtagaaaatggagctggaactggatgaacagtttatcctcccttatctgctgcaattgctcatggaggaggatctgttgatttagctattttttctttacaccttgcaggaatttcttctattttaggagctgttaattttattactactgtaattaatatacgatcaacaggaattacttttgatcgaatacctttatttgtgtgagctgtagtaattacagctattcttttattattatcattaccagtactagctggagctattactatacttttaacagatcgaaacctaaatacttctttttttgacccagcaggaggaggagatccaattttataccaacatttattt

>GBDP26306-19|Haematopota whartoni|COI-5P|MF144238

aactttatattttatttttggagcatgagccggaataattggaacctcattgagtattttaattcgagctgaattaggacatccaggatcattaattggtgatgaccaaatttataatgtaattgtaacagctcatgcatttgtaataattttctttatagtaatacccattataattggaggatttggtaattgattagttccattaatattaggagctcctgatatagcatttcctcgaataaataatataagtttttgacttttacctccttcattaactcttttattagctagtagtatagtagaaaatggagctggaactggatgaacagtttatcctcccttatctgctgcaattgctcatggagggggatctgttgatttagctattttttctttacaccttgcaggaatttcttctattttaggagctgttaattttattactactgtaattaatatacgatcaacaggaattacttttgatcgaatacctttatttgtgtgagctgtagtaattacagctattcttttattattatcattaccagtactagctggagctattactatacttttaacagatcgaaacctaaatacttctttttttgacccagcaggaggaggagatccaattttataccaacatttattt

>GBDP15902-15|Haematopota sp. RSA 2000|COI-5P|KM243541

aacattatattttatttttggtgcatgagccggaataattggaacctcattaagtattttaattcgagctgaattagggcacccaggatcattaattggagatgaccaaatttataatgtaattgtaacagcacatgcttttgtaataattttctttatagtaatacctattataattggaggatttggaaattgattagttccattaatattaggagctcctgatatagctttccctcgaataaataatataagtttttgacttttacctccatcattaactcttttattagccagtagtatagtagaaaatggagctggaactggttgaacagtttaccccccattatcagctgcaattgcccatggaggaggatcagttgatttagcaattttttctttacatcttgcaggaatttcttctattctaggagcagttaattttattactactgtaattaatatacgatcaacaggaattacatttgatcgaatacctctatttgtatgagctgttgtaattactgctgtacttttattattatctttaccagtattagcaggagctattactatattattaacagatcgaaatttaaatacttctttttttgacccagcaggaggaggagatcctattttatatcaacatttattt

>GBDP15901-15|Haematopota rara|COI-5P|KM243540

---------tttcatttttggtgcatgagccggaataattggaacctcattaagtattttaattcgagccgaattaggacatccaggatcattaattggagatgaccaaatttataatgtaattgtaacagcacatgcttttgtaataattttctttatagtaatacctattataattggaggatttggaaattgattagttccattaatattaggagctcccgatatagcttttcctcgaataaataatataagattttgacttttacccccatcattgactcttttattagcaagtagtatagtagaaaacggagctggaactggttgaacagtttaccctccattatcagcagcaatcgcccatgggggaggttcagtagacttagcaatcttttctttacatcttgcaggaatttcttctattttaggagcagttaattttattactactgtaattaatatacgatcagctggaattacttttgatcgaatacctttatttgtatgagctgtagtaattactgccattcttttattattatctttaccagtattagcaggggctattactatattattaacagatcgaaatttaaatacttctttttttgaccctgcaggagggggagaccccattttataccaacatttattt

>GBMNA24644-19|Haematopota brucei|COI-5P|MK396286

-------tattttatttttggagcatgagccggaataattggaacttcattaagtatcttaattcgagctgaattaggacacccaggatcactaattggagatgaccaaatttataatgtaattgttacagcacatgcttttgtaataattttctttatagttatacctattataattggaggatttggaaattgattagttccattaatattaggagctcctgatatagcatttcctcgaataaataatataagtttttgacttttacctccatcattaactcttttattagctagtagtatagtagaaaatggagctggaactggttgaacagtttaccccccactatcagctgctattgcccatggaggtggatcagttgatttagcaattttttcattacaccttgcaggaatttcttctattttaggtgcagttaattttattactactgtaattaatatacgatcaacaggaattacctttgaccgaatacctttatttgtgtgagcagtagtaattactgctattcttttattattatctctaccagtattagctggagctattactatacttttaactgatcgaaatttaaatacatcattttttgacccagcaggaggaggagacccaattctctaccaacatttattt

>GBMNA24643-19|Haematopota brucei|COI-5P|MK396285

-------tattttatttttggagcatgagccggaataattggaacttcattaagtatcttaattcgagctgaattaggacacccaggatccctaattggagatgaccaaatttataatgtaattgttacagcacatgcttttgtaataattttctttatagttatacctattataattggaggatttggaaattgattagttccattaatattaggagctcctgatatagcatttcctcgaataaataatataagtttttgacttttacctccatcattaactcttttattagctagtagtatagtagaaaatggagctggaactggttgaacagtttaccccccactatcagctgctattgcccatggaggtggatcagttgatttagcaattttttcattacaccttgcaggaatttcttctattttaggtgcagttaattttattactactgtaattaatatacgatcaacaggaattacctttgaccgaatacctttatttgtgtgagcagtagtaattactgctattcttttattattatctctaccagtattagctggagctattactatacttttaactgatcgaaatttaaatacatcattttttgacccagcaggaggaggagacccaattctctaccaacatttattt

>GBMNA24712-19|Haematopota brucei|COI-5P|MK396284

-------tattttatttttggggcatgagccggaataattggaacctcattaagtatcttaattcgagctgaattaggacacccaggagcactaattggagatgaccaaatttataatgtaattgttacagcacatgcttttgtaataattttctttatagtaatacctattataattggaggatttggaaattgattagttccattaatattaggagctcctgatatagcattccctcgaataaataatataagtttttgacttttacccccatcattaacccttttattagctagtagtatagtagaaaatggggctggaactggttgaacagtttaccctccactatcagctgctattgcccatggaggaggatcagttgatttagcaattttttcattacacctggcaggaatttcttctattttaggagcagttaattttattactactgtaattaatatacgatcaacaggaattacctttgatcgaatacctctatttgtgtgatcagtagtaattactgctattctcttattattatccttaccagtactagctggagctattactatacttttaactgatcgaaacttaaatacatcattttttgacccagcagggggaggagaccctattctgtaccaacatttattt

>KMPDA141-19|Haematopota|COI-5P

---attatattttatctttggagcatgagccggaataattggaacttcattaagtattttaattcgagctgaattagggcatccaggatccctaattggagatgaccaaatttataatgtaattgttacagcacatgcttttgtaataattttctttatagtaatacccattataattggaggatttggaaattgattagttccactaatattaggggctcctgatatagcatttcctcgaataaataatataagtttttgattattacccccatcattaactcttttattagctagtagtatagtagaaaatggagctggaactggttgaacagtttaccccccattatcagctgctattgcacatggaggaggatcagttgatttagcaattttttcactacatcttgcaggaatttcttctattttaggtgcagttaattttattactactgtaattaatatacgatcaacaggaattacatttgaccgaatacctttatttgtatgagcagtagtaattacagctattcttttattattatcattaccagtattagcaggagctattactatacttttaactgatcgaaatttaaatacatcattttttgacccagcaggaggaggagacccaattctatatcaacatttattt

>KMPDF1895-19|Haematopota|COI-5P

---attatattttatctttggagcatgagccggaataattggaacttcattaagtattttaattcgagctgaattagggcatccaggatccctaattggagatgaccaaatttataatgtaattgttacagcacatgcttttgtaataattttctttatagtaatacccattataattggaggatttggaaattgattagttccactaatattaggggctcctgatatagcatttcctcgaataaataatataagtttttgattattacccccatcattaactcttttattagctagtagtatagtagaaaatggagctggaactggttgaacagtttaccccccattatcagctgctattgcacatggaggaggatcagttgatttagcaattttttcactacatcttgcaggaatttcttctattttaggtgcagttaattttattactactgtaattaatatacgatcaacaggaattacatttgaccgaatacctttatttgtatgagcagtagtaattacagctattcttttattattatcattaccagtattagcaggagctattactatacttttaactgatcgaaatttaaatacatcattttttgacccagcaggaggaggagatccaattctatatcaacatttatt-

>KMPDA127-19|Haematopota|COI-5P

---attatattttatctttggagcatgagccggaataattggaacttcattaagtattttaattcgagctgaattagggcatccaggatccctaattggagatgaccaaatttataatgtaattgttacagcacatgcttttgtaataattttctttatagtaatacccattataattggaggatttggaaattgattagttccactaatattaggggctcctgatatagcatttcctcgaataaataatataagtttttgattattacccccatcattaactcttttattagctagtagtatagtagaaaatggagctggaactggttgaacagtttaccccccattatcagctgctattgcacatggaggaggatcagttgatttagcaattttttcactacatcttgcaggaatttcttctattttaggtgcagttaattttattactactgtaattaatatacgatcaacaggaattacatttgaccgaatacctttatttgtatgagcagtagtaattacagctattcttttattattatcattaccagtattagcaggagctattactatacttttaactgatcgaaatttaaatacatcattttttgacccagcaggaggaggagatccaattctatatcaacatttatt-

>KMPDI147-19|Haematopota|COI-5P

---attatattttatctttggagcatgagccggaataattggaacttcattaagtattttaattcgagctgaattagggcatccaggatccctaattggagatgaccaaatttataatgtaattgttacagcacatgcttttgtaataattttctttatagtaatacccattataattggaggatttggaaattgattagttccactaatattaggggctcctgatatagcatttcctcgaataaataatataagtttttgattattacccccatcattgactcttttattagctagtagtatagtagaaaatggagctggaactggttgaacagtttaccccccattatcagctgctattgcacatggaggaggatcagttgatttagcaattttttcactacatcttgcaggaatttcttctattttaggtgcagttaattttattactactgtaattaatatacgatcaacaggaattacatttgaccgaatacctttatttgtatgagcagtagtaattacagctattcttttattattatcattaccagtattagcaggagctattactatacttttaactgatcgaaatttaaatacatcattttttgacccagcaggaggaggagatccaattctatatcaacatttatt-

>KMPMO064-19|Haematopota|COI-5P

---attatattttatctttggagcatgagccggaataattggaacttcattaagtattttaattcgagctgaattagggcatccaggatccctaattggagatgaccaaatttataatgtaattgttacagcacatgcttttgtaataattttctttatagtaatacccattataattggaggatttggaaattgattagttccactaatattaggggctcctgatatagcatttcctcgaataaataatataagtttttgactattacccccatcattaactcttttattagctagtagtatagtagaaaatggagctggaactggttgaacagtttaccccccattatcagctgctattgcacatggaggaggatcagttgatttagcaattttttcactacatcttgcaggaatttcttctattttaggtgcagttaattttattactactgtaattaatatacgatcaacaggaattacatttgaccgaatacctttatttgtatgagcagtagtaattacagctattcttttattattatcattaccagtattagcaggagctattactatacttttaactgatcgaaatttaaatacatcattttttgacccagcaggaggaggagatccaattctatatcaacatttatt-

>KMPDJ183-19|Haematopota|COI-5P

---attatattttatctttggagcatgagccggaataattggaacttcattaagtattttaattcgagctgaattagggcatccaggatccctaattggagatgatcaaatttataatgtaattgttacagcacatgcttttgtaataattttctttatagtaatacccattataattggaggatttggaaattgattagttccactaatattaggggctcctgatatagcatttcctcgaataaataatataagtttttgattattacccccatcattaactcttttattagctagtagtatagtagaaaatggagctggaactggttgaacagtttaccccccattatcagctgctattgcacatggaggaggatcagttgatttagcaattttttcactacatcttgcaggaatttcttctattttaggtgcagttaattttattactactgtaattaatatacgatcaacaggaattacatttgaccgaatacctttatttgtatgagcagtagtaattacagctattcttttattattatcattaccagtattagcaggagctattactatacttttaactgatcgaaatttaaatacatcattttttgacccagcaggaggaggagacccaattctatatcaacatttat--

>KMPDB057-19|Haematopota|COI-5P

---attatattttatctttggagcatgagccggaataattggaacttcattaagtattttaattcgagctgaattagggcatccaggatccctaattggagatgaccaaatttataatgtaattgttacagcacatgcttttgtaataattttctttatagtaatacccattataattggaggatttggaaattgattagttccactaatattaggggctcctgatatagcatttcctcgaataaataatataagtttttgattattacccccatcattaactcttttattagctagtagtatagtagaaaatggagctggaactggttgaacagtttaccccccattatcagctgctattgcacatggaggaggatcagttgatttagcaattttttcactacatcttgcaggaatttcttctattttaggtgcagttaattttattactactgtaattaatatacgatcaacaggaattacatttgaccgaatacctttatttgtatgagcagtagtaattacagctattcttttattattatcattaccagtattagcaggagctattactatacttttaactgatcgaaatttaaatacatcattttttgacccagcaggaggaggagatccaattctatatcaacatttat--

>KMPGU028-19|Haematopota|COI-5P

---attatattttatctttggagcatgagccggaataattggaacttcattaagtattttaattcgagctgaattagggcatccaggatccctaattggagatgaccaaatttataatgtaattgttacagcacatgcttttgtaataattttctttatagtaatacccattataattggaggatttggaaattgattagttccactaatattaggagctcctgatatagcatttcctcgaataaataatataagtttttgactattacccccatcattaactcttttattagctagtagtatagtagaaaatggagctggaactggttgaacagtttaccccccattatcagctgctattgcacatggaggaggatcagttgatttagcaattttttcactacatcttgcaggaatttcttctattttaggtgcagttaattttattactactgtaattaatatacgatcaacaggaattacatttgaccgaatacctttatttgtatgagcagtagtaattacagctattcttttattattatcattaccagtattagcaggagctattactatacttttaactgatcgaaatttaaatacatcattttttgacccagcaggaggaggagatccaattctatatcaacatttat--

>KMPMO069-19|Haematopota|COI-5P

--cattatattttatctttggggcatgagccggaataattggaacttcattaagtattttaattcgagctgaattagggcatccaggatccctaattggagatgaccaaatttataatgtaattgttacagcacatgcttttgtaataattttctttatagtaatacccattataattggaggatttggaaattgattagttccactaatattaggggctcctgatatagcatttcctcgaataaataatataagtttttgattattacccccatcattaactcttttattagctagtagtatagtagaaaatggagctggaactggttgaacagtttaccccccattatcagctgctattgcacatggaggaggatcagttgatttagcaattttttcactacatcttgcaggaatttcttctattttaggtgcagttaattttattactactgtaattaatatacgatcaacaggaattacatttgaccgaatacctttatttgtatgagcagtagtaattacagctattcttttattattatcattaccagtattagcaggagctattactatacttttaactgatcgaaatttaaatacatcattttttgacccagcaggaggaggagatccaattctatatcaacatttat--

>KMPMO086-19|Haematopota|COI-5P

--cattatattttatctttggagcatgagccggaataattggaacttcattaagtattttaattcgagctgaattagggcatccaggatccctaattggagatgaccaaatttataatgtaattgttacagcacatgcttttgtaataattttctttatagtaatacccattataattggaggatttggaaattgattagttccactaatattaggagctcctgatatagcatttcctcgaataaataatataagtttttgattattacccccatcattaactcttttattagctagtagtatagtagaaaatggagctggaactggttgaacagtttaccccccattatcagctgctattgcacatggaggaggatcagttgatttagcaattttttcactacatcttgcaggaatttcttctattttaggtgcagttaattttattactactgtaattaatatacgatcaacaggaattacatttgaccgaatacctttatttgtatgagcagtagtaattacagctattcttttattattatcattaccagtattagcaggagctattactatacttttaactgatcgaaatttaaatacatcattttttgacccagcaggaggaggagatccaattctatatcaacattta---

>KMPMQ416-19|Haematopota|COI-5P

----ttatattttatctttggagcatgagccggaataattggaacttcattaagtattttaattcgagctgaattagggcatccaggatccctaattggagatgaccaaatttataatgtaattgttacagcacatgcttttgtaataattttctttatagtaatacccattataattggaggatttggaaattgattagttccactaatattaggggctcctgatatagcatttcctcgaataaataatataagtttttgattattacccccatcattaactcttttattagctagtagtatagtagaaaatggagctggaactggttgaacagtttaccccccattatcagctgctattgcacatggaggaggatcagttgatttagcaattttttcactacatcttgcaggaatttcttctattttaggtgcagttaattttattactactgtaattaatatacgatcaacaggaattacatttgaccgaatacctttatttgtatgagcagtagtaattacagctattcttttattattatcattaccagtattagcaggagctattactatacttttaactgatcgaaatttaaatacatcattttttgacccagcaggaggaggagatccaattctatatcaacattta---

>KMPJQ052-19|Haematopota|COI-5P

--cattatattttatctttggagcatgagccggaataattggaacttcattaagtattttaattcgagctgaattagggcatccaggatccctaattggagatgaccaaatttataatgtaattgttacagcacatgcttttgtaataattttctttatagtaatacccattataattggaggatttggaaattgattagttccactaatattaggagctcctgatatagcatttcctcgaataaataatataagtttttgactattacccccatcattaactcttttattagctagtagtatagtagaaaatggagctggaactggttgaacagtttaccccccattatcagctgctattgcacatggaggaggatcagttgatttagcaattttttcactacatcttgcaggaatttcttctattttaggtgcagttaattttattactactgtaattaatatacgatcaacaggaattacatttgaccgaatacctttatttgtatgagcagtagtaattacagctattcttttattattatcattaccagtattagcaggagctattactatacttttaactgatcgaaatttaaatacatcattttttgacccagcaggaggaggagatccaattctatatcaacatttatt-

>KMPDA128-19|Haematopota|COI-5P

aacattatattttatctttggagcatgagccggaataattggaacttcattaagtattttaattcgagctgaattagggcatccaggatccctaattggagatgaccaaatttataatgtaattgttacagcacatgcttttgtaataattttctttatagtaatacccattataattggaggatttggaaattgattagttccactaatattaggagctcctgatatagcatttcctcgaataaataatataagtttttgactattacccccatcattaactcttttattagctagtagtatagtagaaaatggagctggaactggttgaacagtttaccccccattatcagctgctattgcacatggaggaggatcagttgatttagcaattttttcactacatcttgcaggaatttcttctattttaggtgcagttaattttattactactgtaattaatatacgatcaacaggaattacatttgaccgaatacctttatttgtatgagcagtagtaattacagctattcttttattattatcattaccagtattagcaggagctattactatacttttaactgatcgaaatttaaatacatcattttttgacccagcaggaggaggagatccaattctatatcaacatttatt-

>KMPDG126-19|Haematopota|COI-5P

---attatattttatctttggagtatgagccggaataattggaacttcattaagtattttaattcgagctgaattagggcatccaggatccctaattggagatgaccaaatttataatgtaattgttacagcacatgcttttgtaataattttctttatagtaatacccattataattggaggatttggaaattgattagttccactaatattaggggctcctgatatagcatttcctcgaataaataatataagtttttgattattacccccatcattaactcttttattagctagtagtatagtagaaaatggagctggaactggttgaacagtttaccccccattatcagctgctattgcacatggaggaggatcagttgatttagcaattttttcactacatcttgcaggaatttcctctattttaggtgcagttaattttattactactgtaattaatatacgatcaacaggaattacatttgaccgaatacctttatttgtatgagcagtagtaattacagctattcttttattattatcattaccagtattagcaggagctattactatacttttaactgatcgaaatttaaatacatcattttttgacccagcaggaggaggagatccaattcta---------------

>KMPMO085-19|Haematopota|COI-5P

----ttatattttatctttggagcatgagccggaataattggaacctcattaagtattttaattcgagctgaattagggcatccaggatccctaattggagatgaccaaatttataatgtaattgttacagcacatgcttttgtaataattttctttatagtaatacccattataattggaggatttggaaattgattagttccactaatattaggagctcctgatatagcatttcctcgaataaataatataagtttttgactattacccccatcattaactcttttattagctagtagtatagtagaaaatggagctggaactggttgaacagtttaccccccattatcagctgctattgcacatggaggaggatcagttgatttagcaattttttcactacatcttgcaggaatttcttctattttaggtgcagttaattttattactactgtaattaatatacgatcaacaggaattacatttgaccgaatacctttatttgtatgagcagtagtaattacagctattcttttattattatcattaccagtattagcaggagctattactatgcttttaactgatcgaaatttaaatacatcattttttgacccagcaggaggaggagacccaattctatatcaacatttat--

>KMPMQ459-19|Haematopota|COI-5P

----ttatattttatctttggagcatgagccggaataattggaacttcattaagtattttaattcgagctgaattagggcacccaggatccctaattggagatgaccaaatttataatgtaattgttacagcacatgcttttgtaataactttctttatagtaatacccattataattggaggatttggaaattgattagttccactaatattaggggctcctgatatagcatttcctcgaataaataatataagtttttgattattacccccatcattaactcttttattagctagtagtatagtagaaaatggagctggaactggttgaacagtttaccccccattatcagctgctattgcacatggaggaggatcagttgatttagcaattttttcactacatcttgcaggaatttcttctattttaggtgcagttaattttattactactgtaattaatatacgatcaacaggaattacatttgaccgaatacctttatttgtatgagcagtagtaattacagctattcttttattattatcattaccagtattagcaggagctattactatacttttaactgatcgaaatttaaatacatcattttctgacccagcaggaggaggagatccaattctatatcaacatttat--

>KMPMO070-19|Haematopota|COI-5P

----ttatattttatctttggagcatgagccggaataattggaacttcattaagtattttaattcgagctgaattagggcatccaggatccctaattggagatgaccaaatttataatgtaattgttacagcacatgcttttgtaataattttctttatagtaatacccattataattggaggatttggaaattgattagttccactaatattaggagctcctgatatagcatttcctcgaataaataatataagtttttgactattacccccatcattaactcttttattagctagtagtatagtagaaaatggagctggaactggttgaacagtttaccccccattatcagctgctattgcacatggaggaggatcagttgatttagcaattttttcactacatcttgcaggaatttcttctattttaggtgcagttaattttattactactgtaattaatatacgatcaacaggaattacatttgaccgaatacctttatttgtatgagcagtagtaattacagctattcttttattattatcattaccagtattagcaggagctattactatacttttaactgatcgaaatttaaatacatcattttttgacccagcaggaggaggagacccaattctatatcaacatttattt

>KMPDI224-19|Haematopota|COI-5P

-------------------------------------------------------ttcctaattcgagctgaattaggacaccctggatcattaattggagatgaccaaatttataatgtaattgtaacagcacatgcctttgtaataattttctttatagtaatacctattataattggaggatttggaaattgattagtcccattaatattaggagctcctgacatagcatttcctcgaataaataatataagtttttgacttttacccccatcattaacccttttattagctagtagtatagtagaaaatggggctggaactggttgaacagtttatccaccattatcagctgctattgctcatggaggagggtcagtagatttagcaattttttctctacaccttgcaggaatttcctctattttaggtgcagttaattttattactactgtaattaatatacgatcaacaggaattacatttgatcgaatacctctatttgtatgagcagtagtaattactgctattcttttattattatcattaccagtattagctggtgctattactatacttttaacagatcgaaatttaaatacatccttttttgacccagcaggaggaggagacccaattttataccaacactta---

>KMPMQ176-19|Haematopota|COI-5P

---------------ttttggagcatgagccggaataatcggaacttcattaagtactctaattcgagctgaattaggacatcctggatcattaattggagatgaccaaatttataatgtaattgtaacagcacatgcctttgtaataattttctttatagtaatacctattataattggaggatttggaaattgattagtcccattaatattaggagctcctgacatagcatttcctcgaataaataatataagtttttgacttttacctccatcattaacccttttattagctagtagtatagtagaaaatggagctggaactggttgaacagtttatccaccattatcagctgctattgctcatggaggggggtcagtagatttagcaattttttctctacaccttgcaggaatttcctctattttaggtgcagttaattttattactactgtaattaatatacgatcaacaggaattacatttgatcgaatacctctatttgtatgagcagtagtaattactgctattcttttattattatcattaccagtattagctggtgctattactatacttttaacagatcgaaatttaaatacatccttttttgacccagcaggaggaggagacccaattttataccaacactta---

>KMPNO1428-19|Haematopota|COI-5P

---attatattttatttttggagcatgagccggaataatcggaacttcattaagtattctaattcgagctgaattaggacatcctggatcattaattggagatgaccaaatttataatgtaattgtaacagcacatgcctttgtaataattttctttatagtaatacctattataattggaggatttggaaattgattagtcccattaatattaggagctcctgacatagcatttcctcgaataaataatataagtttttgacttttacccccatcattaacccttttattagctagtagtatagtagaaaatggggctggaactggttgaacagtttatccaccattatcagctgctattgctcatggagggggatcagtagatttagcaattttttctctacaccttgcaggaatttcctctattttaggtgcagttaattttattactactgtaattaatatacgatcaacaggaattacatttgatcgaatacctctatttgtatgagcagtagtaattactgctattcttttattattatcattaccagtattagctggtgctattactatacttttaacagatcgaaatttaaatacatccttttttgacccagcaggaggaggagacccaattttataccaacacttattt

>KMPNU1273-19|Haematopota|COI-5P

---attatattttatttttggagcatgagccggaataatcggaacttcattaagtattctaattcgagctgaattaggacatcctggatcattaattggagatgaccaaatttataatgtaattgtaacagcacatgcctttgtaataattttctttatagtaatacctattataattggaggatttggaaattgattagtcccattaatattaggagctcctgacatagcatttcctcgaataaataatataagtttttgacttttacccccatcattaacccttttattagctagtagtatagtagaaaatggggctggaactggttgaacagtttatccaccattatcagctgctattgctcatggagggggatcagtagatttagcaattttttctctacaccttgcaggaatttcctctattttaggtgcagttaattttattactactgtaattaatatacgatcaacaggaattacatttgatcgaatacctctatttgtatgagcagtagtaattactgctattcttttattattatcattaccagtattagctggtgctattactatacttttaacagatcgaaatttaaatacatccttttttgacccagcaggaggaggagacccaattttataccaacacttat--

>KMPRV079-19|Haematopota|COI-5P

---attatattttatttttggagcatgagccggaataatcggaacttcattaagtattctaattcgagctgaattaggacatcctggatcattaattggagatgaccaaatttataatgtaattgtaacagcacatgcctttgtaataattttctttatagtaatacctattataattggaggatttggaaattgattagtcccattaatattaggagctcctgacatagcatttcctcgaataaataatataagtttttgacttttacctccatcattaacccttttattagctagtagtatagtagaaaatggagctggaactggttgaacagtttatccaccattatcagctgctattgctcatggaggggggtcagtagatttagcaattttttctctacaccttgcaggaatttcctctattttaggtgcagttaattttattactactgtaattaatatacgatcaacaggaattacatttgatcgaatacctctatttgtatgagcagtagtaattactgctattcttttattattatcattaccagtattagctggtgctattactatacttttaacagatcgaaatttaaatacatccttttttgacccagcaggaggaggagacccaattttataccaacacttat--

>KMPDI203-19|Haematopota|COI-5P

---attatattttatttttggagcatgagccggaataatcggaacttcattaagtattctaattcgagctgaattagggcatcctggatcattaattggagatgaccaaatttataatgtaattgtaacagcacatgcctttgtaataattttctttatagtaatacctattataattggaggatttggaaattgattagtcccattaatattaggagctcctgacatagcattccctcgaataaataatataagtttttgacttttacctccatcattaacccttttattagctagtagtatagtagaaaatggagctggaactggttgaacagtttatccaccattatcagctgctattgctcatggaggggggtcagtagatttagcaattttttctctacaccttgcaggaatttcctctattttaggtgcagttaattttattactactgtaattaatatacgatcaacaggaattacatttgatcgaatacctctatttgtatgagcagtagtaattactgctattcttttattattatcattaccagtattagctggtgctattactatacttttaacagatcgaaatttaaatacatccttttttgacccagcaggaggaggagacccaattttataccaacacttatt-

>KMPLY019-19|Haematopota|COI-5P

--cattatattttatttttggagcatgagccggaataatcggaacttcattaagtattctaattcgagctgaattagggcatcctggatcattaattggagatgaccaaatttataatgtaattgtaacagcacatgcctttgtaataattttctttatagtaatacctattataattggaggatttggaaattgattagtcccattaatattaggagctcctgacatagcatttcctcgaataaataatataagtttttgacttttacctccatcattaacccttttattagctagtagtatagtagaaaatggagctggaactggttgaacagtttatccaccattatcagctgctattgctcatggaggggggtcagtagatttagcaattttttctctacaccttgcaggaatttcctctattttaggtgcagttaattttattactactgtaattaatatacgatcaacaggaattacatttgatcgaatacctctatttgtatgagcagtagtaattactgctattcttttattattatcattaccagtattagctggtgctattactatacttttaacagatcgaaatttaaatacatccttttttgacccagcaggaggaggagacccaattttataccaacacttatt-

>KMPXV1042-19|Haematopota|COI-5P

aacattatattttatttttggagcatgagccggaataatcggaacttcattaagtattctaattcgagctgaattagggcatcctgggtcattaattggagatgaccaaatttataatgtaattgtaacagcacatgcctttgtaataattttctttatagtaatacctattataattggaggatttggaaattgattagtcccattaatattaggagctcctgacatagcatttcctcgaataaataatataagtttttgacttttacctccatcattaacccttttattagctagtagtatagtagaaaatggagctggaactggttgaacagtttatccaccattatcagctgctattgctcatggaggggggtcagtagatttagcaattttttctctacaccttgcaggaatttcctctattttaggtgcagttaattttattactactgtaattaatatacgatcaacaggaattacatttgatcgaatacctctatttgtatgagcagtagtaattactgctattcttttattattatcattaccagtattagctggtgctattactatacttttaacagatcgaaatttaaatacatccttttttgacccagcaggaggaggagacccaattttataccaacacttat--

>KMPDM071-19|Haematopota|COI-5P

---attatattttatttttggggcatgagccggaataatcggaacttcattaagtattctaattcgagctgaattagggcatcctggatcattaattggagatgaccaaatttataatgtaattgtaacagcacatgcctttgtaataattttctttatagtaatacctattataattggaggatttggaaattgattagtcccattaatattaggagctcctgacatagcatttcctcgaataaataatataagtttttgacttttacctccatcattaacccttttattagctagtagtatagtagaaaatggggctggaactggttgaacagtttatccaccattatcagctgctattgctcatggaggggggtcagtagatttagcaattttttctctacaccttgcaggaatttcctctattttaggtgcagttaattttattactactgtaattaatatacgatcaacaggaattacatttgatcgaatacctctatttgtatgagcagtagtaattactgctattcttttattattatcattaccagtattagctggtgctattactatacttttaacagatcgaaatttaaatacatccttttttgacccagcaggaggaggagacccaattttataccaacactta---

>KMPDI211-19|Haematopota|COI-5P

----ttatattttatttttggagcatgagccggaataatcggaacttcattaagtattctaattcgagctgaattagggcatcctggatcattaattggagatgaccaaatttataatgtaattgtaacagcacatgcctttgtaataattttctttatagtaatacctattataattggaggatttggaaattgattagtcccattaatattaggagctcctgatatagcatttcctcgaataaataatataagtttttgacttttacccccatcattaacccttttattagctagtagtatagtagaaaatggggctggaactggttgaacagtttatccaccattatcagctgctattgctcatggagggggatcagtagatttagcaattttttctctacaccttgcaggaatttcctctattctaggtgcagttaattttattactactgtaattaatatacgatcaacaggaattacatttgatcgaatacctctatttgtatgagcagtagtaattactgctattcttttattattatcattaccagtattagctggtgctattactatacttttaacagatcgaaatttaaatacatccttttttgacccagcaggaggaggagacccaattttataccaacacttatt-

>KMPOX005-19|Haematopota|COI-5P

----------------tttggagcatgagccggaataatcggaacttcattaagtattctaattcgagctgaattagggcatcctggatcattaattggagatgaccaaatttataatgtaattgtaacagcacatgcctttgtaataattttctttatagtaatacctattataattggaggatttggaaattgattagtcccattaatattaggagctcctgatatagcatttcctcgaataaataatataagtttttgacttttacccccatcattaactcttttattagctagtagtatagtagaaaatggggctggaactggttgaacagtttacccaccattatcagccgctattgcccatggaggggggtcagtagatttagcaattttttctctacaccttgcaggaatttcctctattctaggtgcagttaattttattactactgtaattaatatacgatcaacaggaattacatttgatcgaatacctctatttgtatgagcagtagtaattactgctattcttttattgttatcattaccagtattagccggtgctattactatacttttaacagatcgaaatttaaatacatccttttttgacccggcaggaggaggagacccaattttataccaacacttatt-

>KMPMO111-19|Haematopota|COI-5P

aacattatattttatttttggggcatgagccggaataattggaacttcattaagtattctaattcgagctgaattaggacaccctggatcattaattggagatgaccaaatttataatgtaattgtaacagcacatgcttttgtaataattttctttatagtaatacctattataattggaggatttggaaattgattagtcccattaatattaggagctcctgatatagcatttcctcgaataaataatataagtttttgacttttacctccatcattaacccttttattagctagtagtatagtagaaaatggagctggaactggttgaacagtttacccaccattatcagctgctattgctcatggaggaggatcagtagatttagcaattttttctctacaccttgcaggaatttcttctattttaggtgcagttaattttattactactgtaattaatatacgatcaacaggaattacatttgatcgaatacctctatttgtatgagcagtagtaattactgctattcttttattactatcattaccggtattagctggtgctattactatgcttttaacagatcgaaatttaaatacatccttttttgacccagcaggaggaggggatccaattctataccaacacttat--

>KMTTI1250-19|Haematopota|COI-5P

----ttatattttatttttggggcatgagccggaataattggaacttcattaagtattctaattcgagctgaattaggacaccctggatcattaattggagatgaccaaatttataatgtaattgtaacagcacatgcttttgtaataattttctttatagtaatacctattataattggaggatttggaaattgattagtcccattaatattaggagctcctgatatagcatttcctcgaataaataatataagtttttgacttttacccccatcattaacccttttattagctagtagtatagtagaaaatggagctggaactggttgaacagtttacccaccattatcagctgctattgctcatggaggaggatcagtagatttagcaattttttctctacaccttgcaggaatttcttctattttaggtgcagttaattttattactactgtaattaatatacgatcaacaggaattacatttgatcgaatacctctatttgtatgagcagtagtaattactgctattcttttattactatcattaccggtattagctggtgctattactatgcttttaacagatcgaaatttaaatacatccttttttgacccagcaggaggaggggatccaattctataccaacacttat--

>KMPMX045-19|Haematopota|COI-5P

---cttatattttatttttggggcatgagccggaataattggaacctcattaagtattttaattcgagctgaattaggtcacccgggatcattaattggagatgaccaaatttataatgtaattgtaacagcacatgcttttgtaataattttctttatagtaatacctattataattggaggatttggaaattgactagtcccattaatattaggagcgcctgatatggcattccctcgaataaataatataagtttttgacttttacccccatcattaactcttttattagctagtagtatagtagaaaatggagctggaacaggttgaactgtttaccccccattatcagctgctattgctcatggaggaggatcagtagatttagcaattttttctttacaccttgcaggaatttcttctattttaggggcagttaattttattactactgtaattaatatacgatcaacaggaattacatttgatcgaatacctttatttgtttgagctgtagtaattactgctattcttttattattatctttaccagtattagctggtgccattactatacttttaactgatcgaaatttaaatacatccttctttgaccctgcaggaggaggagacccaattttatatcaacatttat--

>GBMNA24662-19|Haematopota albihirta|COI-5P|MK396282

-------tattttattttcggagcatgagccggaatgattggaacttcattaagtattctaattcgggctgaattaggacacccaggatcattaattggagatgaccaaatttataatgtaattgtaacagcgcatgcttttgtaataattttctttatagtaatacctattataattggaggatttggaaattgattagttccattaatattaggagcccctgacatagcatttcctcgaataaataatataagtttttgacttttacccccatcactgactcttttattagctagtagtatagtagaaaatggggctggaactggttgaacagtttatccaccattatctgctgccattgctcatggaggaggatcagtagatttagcaattttttctttacatcttgcaggaatttcctcaattttaggagcagttaattttattactactgtaattaatatacgatcaactggaattacatttgatcgaatacctttatttgtatgagctgtagtaattactgccatccttttattattatcattaccagtattagctggtgctattactatacttttaactgatcgaaatttaaatacatctttctttgacccagcaggaggaggagacccaattttatatcaacatttattt

>GBMNA24661-19|Haematopota albihirta|COI-5P|MK396281

-------tattttattttcggagcatgagccggaatgattggaacttcattaagtattctaattcgggctgaattaggacacccaggatcattaattggagatgaccaaatttataatgtaattgtaacagcgcatgcttttgtaataattttctttatagtaatacctattataattggaggatttggaaattgattagttccattaatattaggagcccctgacatagcatttcctcgaataaataatataagtttttgacttttacccccatcactgactcttttattagctagtagtatagtagaaaatggggctggaactggttgaacagtttatccaccattatctgctgccattgctcatggaggaggatcagtagatttagcaattttttctttacatcttgcaggaatttcctcaattttaggagcagttaattttattactactgtaattaatatacgatcaactggaattacatttgatcgaatacctttatttgtatgagctgtagtaattactgccatccttttattattatcattaccagtattagctggtgctattactatacttttaactgatcgaaatttaaatacatctttctttgacccagcaggaggaggagacccaattttatatcaacatttattt

>GBMNA24711-19|Haematopota ciliatipes|COI-5P|MK396288

-------tattttatttttggggcatgagccggaataatcggaacctcattaagtattttaattcgagcagaattaggacatcctgggtcattaattggtgatgaccaaatttataatgtaattgtaacagcacatgcttttgtaataattttctttatagtaatacctattataattggaggatttggaaattgattagtcccattaatattaggagctcctgatatagctttccctcgaataaataatataagtttttgacttttacctccatcattaactcttttattagctagtagtatagtagaaaatggagctggaacaggttgaacagtttaccctccattatcagctgctatcgctcatggagggggatcagtagatttagcaattttttcattacatcttgcaggaatttcttctattttaggagcagttaattttattactactgtaattaatatacgatcagcaggaattacatttgaccgaatacctttatttgtgtgagctgtagtaattactgctattcttttattattatctttaccagtattagctggtgctattactatgcttttaactgatcgaaatttaaatacatctttttttgaccccgcaggagggggagacccaattttataccaacatctattt

>GBMNA24710-19|Haematopota ciliatipes|COI-5P|MK396287

-------tattttatttttggggcatgagccggaataatcggaacctcattaagtattttaattcgagcagaattaggacatcctgggtcattaattggtgatgaccaaatttataatgtaattgtaacagcacatgcttttgtaataattttctttatagtaatacctattataattggaggatttggaaattgattagtcccattaatattaggagctcctgatatagctttccctcgaataaataatataagtttttgacttttacctccatcattaactcttttattagctagtagtatagtagaaaatggagctggaacaggttgaacagtttaccctccattatcagctgctatcgctcatggaggaggatcagtagatttagcaattttttcattacatcttgcaggaatttcttctattttaggagcagttaattttattactactgtaattaatatacgatcagcaggaattacatttgaccgaatacctttatttgtgtgagctgtagtaattactgctattcttttattattatctttaccagtattagctggtgctattactatgcttttaactgatcgaaatttaaatacatctttttttgaccctgcaggagggggagacccaattttataccaacatctattt

>GBMNA24707-19|Haematopota griseicoxa|COI-5P|MK396291

-------tattttatctttggggcatgagccggaataattggaacctcattaagtatcttaattcgagctgaattaggacatccagggtcattaattggagacgatcaaatttataatgtaattgtaacagcacatgcttttgtaataattttctttatagtaatacctattataattggaggatttggaaattgactagttccattaatattaggagcccccgatatagcattccctcgaataaataatataagtttttgacttttacctccatcattgactcttttattagctagtagtatagtagaaaatggggccggaacaggttgaacagtttatcctccattatcagctgctattgctcatggaggaggatcagtagatttagcaattttttctttacatcttgcaggaatttcctcaattttaggggcagttaattttattactactgtaattaatatacgatcaacaggaattacatttgatcgaatacctttatttgtctgggctgtagtaattactgctattctattattattatctctaccagtattagcaggagctattactatacttttaactgatcgtaatttaaatacatctttctttgaccctgcgggtggaggagacccaattttataccaacatttattt

>GBMNA24709-19|Haematopota griseicoxa|COI-5P|MK396293

-------tattttatctttggagcatgagccggaataattggaacctcattaagtatcttaattcgagctgaattaggacatccagggtcattaattggagacgatcaaatttataatgtaattgtaacagcacatgcttttgtaataattttctttatagtaatacctattataattggaggatttggaaattgactagttccattaatattaggagcccccgatatagcattccctcgaataaataatataagtttttgacttttacctccatcattgactcttttattagctagtagtatagtagaaaatggggccggaacaggttgaacagtttatcctccattatcagctgctattgctcatggaggaggatcagtagatttagcaattttttctttacatcttgcaggaatttcctcaattttaggggcagttaattttattactactgtaattaatatacgatcaacaggaattacatttgatcgaatacctttatttgtctgggctgtagtaattactgctattctattattattatctctaccagtattagcaggagctattactatacttttaactgatcgtaatttaaatacatctttctttgaccctgcgggtggaggagacccaattttataccaacatttattt

>GBMNA24708-19|Haematopota griseicoxa|COI-5P|MK396292

-------tattttatctttggagcatgagccggaataattggaacctcattaagtatcttaattcgagctgaattaggacatccggggtcattaattggagacgatcaaatttataatgtaattgtaacagcacatgcttttgtaataattttctttatagtaatacctattataattggaggatttggaaattgactagttccattaatattaggagcccccgatatagcattccctcgaataaataatataagtttttgacttttacctccatcattgactcttttattagctagtagtatagtagaaaatggggccggaacaggttgaacagtttatcctccattatcagctgctattgctcatggaggaggatcagtagatttagcaattttttctttacatcttgcaggaatttcctcaattttaggggcagttaattttattactactgtaattaatatacgatcaacaggaattacatttgatcgaatacctttatttgtctgggctgtagtaattactgctattctattattattatctctaccagtattagcaggagctattactatacttttaactgatcgtaatttaaatacatctttctttgaccctgcgggtggaggagacccaattttataccaacatttattt

>GBMNA24701-19|Haematopota guineensis|COI-5P|MK396294

aacattatattttatttttggggcatgagccggaataattggaacttcattaagtattttaattcgtgctgaattaggacaccctggatcactaattggagatgaccaaatttataatgtaattgtaacagcgcatgcttttgtaataattttctttatagtaatacctattataattggaggatttggaaattgattagttccattaatactaggagctcctgatatagcatttcctcgaataaataatataagtttttgacttttacccccatcactgactcttttattagctagtagtatagtggaaaatggagctggaacaggttgaacagtttatccaccattatcagctgctattgctcacggggggggttcagtagatttagcaattttttctttacaccttgcaggaatttcctctattttaggggctgttaattttattaccacagtaattaatatacgatcaacaggtattacatttgatcgaatacctttatttgtctgatctgtagtaattactgctattcttttattattatctttaccagtattagctggtgccattactatacttttaactgatcgaaatttaaatacatccttttttgacccagcgggagggggagatccaatcttataccaacatttattt

>GBMND40252-21|Haematopota|COI-5P|MT083915

--------------------------------------ttggaacctcattaagtatcctaattcgagctgaattaggacacccaggatcattaattggagatgaccaaatttataatgtaattgtaacagcacacgcttttgtaataattttctttatagtaatacctattataattggaggatttggaaattgattagttcctttaatattaggagctcctgatatagcatttcctcgaataaataatataagtttttgacttttacctccatcattaactcttttattagcaagtagtatagtagaaaatggagctggtactggttgaacagtttaccccccattatcagctgctattgctcatggaggaggatcagttgatttagcaattttttccttacatcttgcaggaatttcttctattttaggagctgttaattttattactactgtaattaacatacgatcgacaggaattacatttgatcgaatacctttatttgtgtgagcagtagtaattaccgctattttattattattatctttaccagtattagctggggcaattactatacttttaactgatcgaaatctaaatacctcttttttcgacccagcaggaggaggagatcctattttatatcaacatttattt

>GBMNC9030-20|Haematopota glenni|COI-5P|MK393434

----ttgatttgggtcctttgtagctgtagaggaataattgggacctcattaagtattctaattcgagctgaattaggacacccaggatcattaattggtgacgatcaaatttataatgtaattgtaacagcacatgcttttgtaataattttctttatagtaatacctattataattggaggatttggaaattgattagttccattaatattaggagctcctgatatagcatttcctcgaataaataatataagtttttgacttttacctccatcattaactcttttattagcaagtagtatagtagaaaatggagctggtactggttgaacagtttaccctcctctatcagctgctattgctcatggaggaggatcagtagatctagcaattttttctttacaccttgcaggaatttcttctattttaggagctgttaattttattactactgttattaatatacgatcaacaggaattacatttgaccgaatacctttattcgtatgagcagtagtaattactgctattcttttattattatcattaccagtattagcaggagctattactatacttttaactgatcgaaatttaaatacttcattttttgatcctgcgggaggaggagatcctattttataccaacatttattt

>GBMNA24611-19|Haematopota torquens|COI-5P|MK396280

aacattatattttatttttggagcatgagccggaataattgggacatcattaagtatcttaattcgagctgaattaggacacccaggatcactaattggtgatgatcaaatttataatgtaattgtaacagcgcatgcttttgtaataattttctttatagtaatgccgattataattggaggatttggaaattgattagttccattaatattaggagctcctgatatagcatttcctcgaataaataatataagtttttgacttttacctccatcattgactcttttattagcaagtagtatagtagaaaatggagctggaactggttgaacagtttaccccccgttatcagctgcaattgcccatggaggaggttcagttgatttagcaattttttctttacatttagcaggaatttcttcaattttaggagctgttaattttattactactgtaattaatatacgatcaacaggaattacatttgaccgaatacctttatttgtgtgatctgtagtaattactgctgtacttttacttttatctttaccagtattagcaggagctattacaatacttttaacagatcggaatttaaatacatctttttttgaccctgcgggaggaggagaccctattttataccaacatttattt

>GBMNA24685-19|Haematopota furians|COI-5P|MK396290

-------tattttatttttggagcatgagccggaataattgggacatcattaagtattctaattcgagctgaattaggacaccctgggtcactaattggtgatgatcaaatttataatgtaattgtaacagcacatgcttttgtaataattttctttatagtaatacctattataattggaggatttggaaattgattagttccattaatattaggagctcctgatatagcatttcctcgaataaataatataagtttctgacttttacctccatcattgactcttttattagcaagaagtatagtagaaaatggagctggaactggttgaacagtttaccctccattatcagctgcaattgctcatggaggaggttcagttgatttagcaattttctctttacatttagcaggaatttcttctattttaggagctgttaattttattacaactgtaattaatatacgatcaacaggaattacatttgaccgaataccattatttgtatgatctgtagtaattactgctgtacttttattattatctttaccagtattagcaggagctattacaatacttttaacagatcgaaatttaaatacatctttctttgatcctgcaggaggaggagaccctattttatatcaacatttattc

>GBMNA24683-19|Haematopota furians|COI-5P|MK396289

-------tattttatttttggagcatgagccggaataattgggacatcattaagtattctaattcgagctgaattaggacaccctgggtcactaattggtgatgatcaaatttataatgtaattgtaacagcacatgcttttgtaataattttctttatagtaatacctattataattggaggatttggaaattgattagttccattaatattaggagctcctgatatagcatttcctcgaataaataatataagtttctgacttttacctccatcattgactcttttattagcaagaagtatagtagaaaatggagctggaactggttgaacagtttaccctccattatcagctgcaattgctcatggaggaggttcagttgatttagcaattttctctttacatttagcaggaatttcttctattttaggagctgttaattttattacaactgtaattaatatacgatcaacaggaattacatttgaccgaataccattatttgtatgatctgtagtaattactgctgtacttttattattatctttaccagtattagcaggagctattacaatacttttaacagatcgaaatttaaatacatctttctttgatcctgcaggaggaggagaccctattttatatcaacatttattc

>GBMNA24670-19|Haematopota bowdeni|COI-5P|MK396283

-------tattttatttttggagcatgagccggaataatcggaacatccttaagtattttaattcgagctgaattaggacatcctggatcattaattggtgatgaccaaatttataatgtaattgtaactgcgcatgcttttgtaataatttttttcatagtaatgcctattataattggaggattcggaaattgattagttccattaatattaggggctcctgatatagcatttcctcgaataaataatataagtttctgacttttacctccatcattaactcttttattagcaagtagtatagtagaaaatggagctggaactggttgaacagtttatcccccattatcagctgcaattgcccatggaggaggttcagttgatttagcaattttttctttacatttagcaggaatttcatctattttaggagctgttaattttattactactgtaattaatatacgatcaacaggaattacatttgaccgaatacctttatttgtatgatccgtagtaattactgctattcttttacttttatctttaccagtattagcaggagctattacaatacttttaacagatcgaaatttaaatacatctttttttgacccagcaggaggaggagatcctatcttataccaacatctattt

>GBDP26289-19|Haematopota libera|COI-5P|MF144221

aacattatattttatttttggagcctgagctggaataattggaacttcattaagtattttaatttgagctgaattaggacatccaggttctttaattggagatgaccaaatttataatgtaattgttactgctcatgcttttgtaataattttctttatagtaatacctattataattggaggatttggaaattgattagttccattaatattaggagctcccgatatagcatttcctcgaataaataatataagattttgattattacctccatcattaactcttttattagcaagtagtatggtagaaaacggggcaggtacaggttgaacagtgtacccccctctatcagcagctattgctcatggaggaggttctgtagacttagcaattttttctttacatcttgcaggaatttcttcaattttaggagctgtaaattttattactactgtaattaatatacgttcaacaggaattacatttgatcgaatacctttatttgtatgagctgtagtaattacagctattcttttattattatctttaccagtattagcaggagcaattactatacttttaacagatcgaaatttaaatacttcattttttgacccagctggaggaggagatcctattttatatcaacatttattt

>GBMNB25110-20|Haematopota albimedia|COI-5P|MT193005

----------------------gcaagagctggaataattggaacctcattaagtattttaattcgagctgaattaggacacccaggttcattaattggtgatgatcaaatttataatgtaatcgtaacagcacatgcttttgtaataattttctttatagttatacctattataattggaggatttggtaattgattagttcctctaatgttaggagctcctgatatagcattccctcgaataaataatataagtttctgacttttacccccatcattaacacttttattagcaagtagtatagtagaaaatggagctggtactggttgaacagtttaccccccattatcagctgctattgcccatggaggaggatcagttgatttagcaattttttctctacaccttgcaggtatttcttcaattttaggggctgttaattttattactactgtaattaatatacgatcaacaggaattacattcgatcgaatacccttatttgtatgatcagtagtaattactgctattttattattactatctttaccagtattagcaggtgcaattactatacttttaactgatcgaaatttaaatacttccttttttgacccagcaggaggaggagatccaattttatatcaaca-------

>GBDP25185-19|Haematopota longa|COI-5P|KY555753

----------------------------------acaattggaacctcattatgtattttcattcgagctgaattaggacatccaggatctttaattggtgatgaccaaatttataatgtaattgtaactgcacatgcatttgtaataattttctttatagttatacccattataattggtggatttggaaattggttagttccattaatattaggagctcctgatatagcattcccacgaataaataatataagtttttgtcttttacctccatcattaactcttttattagcaagtagtatagtagaaaatggagctggaactggctcaacagtttacccaccattatcagctgcaattgctcatggaggaggatcagtagatttagcaattttttctttacaccttgcaggaatttcttcaattttaggggctgtaaattttattactactgtaattaatatacgatcaacaggaattacttttgatcgaatacctttatttgtattagctgtagtaattacagctattctattattgttatctttaccagtattagccggtgccattactatacttttaacagatcgaaatttaaatacttctttttttgatcctgctggaggaggagaccctattttatatcaa---------

>GBDP16696-15|Haematopota javana|COI-5P|KM111712

--------------------------------------ttggaacttcattaagtattttaattcgagctgaattaggacaccccggatcattaattggtgatgatcaaatttataatgtaattgtaacagcacatgcttttgttataattttctttatagttataccaattataattggaggatttggtaattgattagttcctttaatattaggagctcctgatatagcctttcctcgaataaataatataagtttttgattattacccccatcattaactcttttattagctagtagtatagtagaaaatggagctggaactggatgaacagtttacccaccactatcagctgctattgctcatggaggaggatcagttgatttagctattttttctcttcatttagcaggaatttcttcaattttaggggcagttaattttattactactgtaattaatatacgatctacaggaattacatttgatcgaatacccttatttgtatgagcagtagtaattactgctattttattattattatctttacctgtattagcaggtgctattacaatacttttaactgatcgaaatttaaatacatcattttttgacccagctggaggaggagacccaattttatatcaacatttattt

>GBDP15873-15|Haematopota personata|COI-5P|KM243505

aacattatattttatttttggagcatgagctggaataattggaacttcattaagtattttaattcgagctgaattaggtcatccaggatcattaattggagatgaccaaatttacaatgtaattgtaacagcacatgcttttgttataattttctttatagtaatacctattataattggaggatttggaaattgattagttccattaatattaggagctcctgatatagcatttcctcgaataaataatataagtttttgacttttacctccatcattaacccttttattagcaagtagtatagtagaaaatggagctggaactggttgaacagtttaccccccattatcagctgcaattgcccatggaggaggatcagttgttttctccattttttctttacatcttgcaggaatttcgtctattttaggaggtgttaattttattactgcagtaattaatatacgatcaacaggaattacatttgatcgaatacctttatttgtatgagctgtagtaattactgctattcttttattactatctttaccagtattagccggagctattactatacttttgactgatcgaaatttaaaatacctcttttttgaccctgcaggaggaggtgaccctattttataccaacacat----

>GBDP25184-19|Haematopota longa|COI-5P|KY555752

--------attttttttttggagcatgagctggaataattggaacctcattaagtattttaattcgagctgcattaggacatccaggatctttaattggtgttgcccaaatttatattgttattgtatctgcacatgcatttgtatttattttctttaaagttatacccattattattggtggatttggaaattgataagttccatgaatatttggagctcctgttattgcattcccacgaaaaaaaaatatatgtttttgacttttacctccatcatttactcttttatttgcaagtactatagtagaaaatggagctggaactggttcaacagtttacccaccattatcagctgcaattgctcatgggggaggatcagtagatttagcaattttttctttacaccttgcaggaatttcttcaattttaggggctgtaaattttattactactgtaattaatatacgatcaacaggaattacttttgatcgaatacctttatttgtatcagctgtagtaattacagctattctattattgttatctttaccagtattagccggtgccattactatacttttaacagatcgaaatttaaatacttctttttttgatcctgctggaggaggagaccctattttatatcaaca-------

>FIDIP284-11|Heptatoma pellucens|COI-5P|Pending (#7798)

aacattatattttattttcggggcatgagctggaataattggtacatcactaagtatactaattcgagctgaattaggccatccaggttcattaattggagatgatcaaatttacaatgtaattgtaacagcacacgctttcgtaataattttttttatagtaatacctattataattggaggatttggaaattgattagttcctttaatattaggagctcctgatatagcatttcctcgaataaataatataagtttttgactactacccccttcattaactctattattagcaagtagtatagtagaaaacggagctgggactggatgaactgtttacccacctctttcagctgcaattgctcatggaggaggatcagtagatttagcaattttttcattacatttagcaggaatttcttcaattttaggagctgttaattttattaccactgtaattaatatacgatctacaggtatttcatttgatcgaatacctttatttgtttgagcagttgtaattacagctattcttttattgttatcactaccagttttagccggagctattacaatactattaacagatcgaaatttaaatacatctttttttgacccagctggaggaggagacccaattttataccaacatttattt

>CROBB324_SK-40_Heptatoma pellucens

aacattatattttattttcggggcatgagctggaataattggtacatcactaagtatactaattcgagctgaattaggccatccaggttcattaattggagatgatcaaatttacaatgtaattgtaacagcacacgctttcgtaataattttttttatagtaatacctattataattggaggatttggaaattgactagttcctttaatattaggggctcctgatatagcatttcctcgaataaataatataagtttttgactactacccccttcattaactctattattagcaagtagtatagtagaaaacggagctggaactggatgaactgtttacccacctctttcagctgcaattgctcatggaggaggatcagtagatttagcaattttttcattacatttagcaggaatttcttcaattttaggagctgttaattttattaccactgtaattaatatacgatctacaggtatttcatttgatcgaatacctttatttgtttgagcagttgtaattacagctattcttttattgttatcactaccagttttagccggagctattacaatactattaacagatcgaaatttaaatacatctttttttgacccagctggaggaggagacccaattttataccagcatttattt

>GMFIE626-12|Rhagio maculatus|COI-5P

aacattatattttatttttggggcttgagctggaatagtaggaacatctttaagtatattaattcgagctgaattaggacacccaggagccttaattggtgatgatcaaatttataatgtaattgttactgctcatgcctttgtaataattttctttatagtaatgcctattataattggaggatttggaaattgattagtacctttaatattaggggctccagatatagccttcccccgaataaataatataagattttgattattacctccctcattaactttacttttatctagtagtatagtagaaaatggagcaggtactggatgaacagtttaccctccgctttctgcctctattgcccatggaggggcctctgttgatttagccattttctcccttcatttagctggtatttcttctattttaggggcagtaaattttattaccacagtaattaatatacgatcaacaggaattacatttgatcgaatacccctatttgtatgagctgttgtaattactgctattcttttattattatccttacctgtattagcaggtgcaattactatattattaacagatcgaaatctaaatacatcgttctttgaccctgctggtgggggagaccctattctataccaacatttattt

>AMTPD3344-15|Chrysopilus nubecula|COI-5P

-actttatattttatttttggggcatgggcaggaatagtaggtacttcacttagtatactaattcgagcagaactaggtcatcctggagctttaattggtgatgatcaaatttataatgtaatcgttaccgctcatgcttttgtaataattttctttatagtaatgcctattataatcggaggatttggtaattgattagtcccattaatattgggggctcctgatatagcatttccacgaataaataatataagattctgattattacccccctctttaactttattgttagcaagtagtatagtagaaaatggggcaggtactggatgaactgtctaccctcccctttcagctggaattgcccacgctggagcttctgtagatctcgctattttttctttacatcttgcaggagtctcctcaattttaggtgccgtaaattttattacaacagtaattaatatacgaacaacgggtattacttttgaccgaatgccattatttgtttgagccgtagtaattacagcaattttattactactatccttaccagtattagccggagctattacaatattattaacagat---------------------------------------------------------------------

>AMTPD3492-15|Symphoromyia crassicornis|COI-5P

----------ttatttttgggggcatgagcaggtatagttggtacttctctaagaatactaattcgagctgaactaggccacccaggagccttaattggtgatgatcaaatctataatgtaattgttacagctcatgcttttgtaataattttttttatagtaatacctattataattggaggatttggtaattgattagtcccattaatattaggagccccagatatagcatttccacgaataaataatataagattctgattactgcccccatctttaactctattattagcaagaagcatagtagaaaatggagcaggaacaggatgaacagtatatccccccctatcggccagaattgcacatgctggagcttctgttgatttagctattttttcattgcatttagccggagtttcttctattttaggggctgtaaattttattacaacagttattaatatacgatcaacaggaatttcgtttgatcgaatacctctatttgtatgagcggttgtaattactgctattcttttattactatcactaccagtattagcaggagctattacaatactattaacagat---------------------------------------------------------------------

>GBMIN38560-13|Haematopota sp. SIM1000.1|COI-5P|DQ983519

--------------------------------------------------------------------------------------------------------------------------------------------------------------------------------------------gaaattgattagttccattaatattaggagctcctgatatagctttccctcgaataaataatataagtttttgacttttacctccatcattaactcttttattagccagtagtatagtagaaaatggagctggaactggttgaacagtttaccccccattatcagctgcaattgcccatggaggaggatcagttgatttagcaattttttctttacatcttgcaggaatttcttctattctaggagcagttaattttattactactgtaattaatatacgatcaacaggaattacatttgatcgaatacctctatttgtatgagccgttgtaattactgctgtacttttattattatctttaccagtattagcaggagctattactatattattaacagatcgaaatttaaatacttctttttttgacccagcaggaggaggagatcctattttatatcaacatttattt

3. TABANINI AND DIACHLORINI

>CNNHA009-14|Hybomitra lurida|COI-5P|KR495919

-acattatattttattttcggggcatgagctggaataattggtacctcattaagtattttaattcgagctgaattaggacatcctggatcattaattggtgatgaccaaatttataatgtaattgtaacagcacatgcttttgtaataattttctttatagtaatacctattataattggaggatttggaaattgattagttcctttaatattaggagctcctgatatagcatttcctcgaataaataatataagtttttgattacttccgccatcattgactcttttattagccagtagtatagtagaaaatggagctggaactggatgaacagtttaccnncntctatcagctgcaattgctcatggaggaggatcagttgatttagcaattttttccctacatttagcaggaatttcatctattttaggagctgttaattttattactactgtaattaatatacgatcaacaggagtaacttttgaccgaatacctttatttgtatgagcagtagtgattacagctattcttctattattatcattaccagtattagctggagctattactatacttttaactgat---------------------------------------------------------------------

>CNPAL1056-13|Hybomitra lurida|COI-5P|KR598871

aacattatattttattttcggggcatgagctggaataattggtacctcattaagtattttaattcgagctgaattaggacatcctggatcattaattggtgatgaccaaatttataatgtaattgtaacagcacatgcttttgtaataattttctttatagtaatacctattataattggaggatttggaaattgattagttcctttaatattaggagctcctgatatagcatttcctcgaataaataatataagtttttgattacttccgccatcattgactcttttattagccagtagtatagtagaaaatggagctggaactggatgaacagtttacccccctctatcagctgcaattgctcatggaggaggatcagttgatttagcaattttttccctacatttagcaggaatttcatctattttaggagctgttaattttattactactgtaattaatatacgatcaacaggagtaacttttgaccgaatacctttatttgtatgagcagtagtgattacagctattcttctattattatcattaccagtattagctggagctattactatacttttaactgat---------------------------------------------------------------------

>CNWBE842-13|Hybomitra lurida|COI-5P|KP049157

aacattatattttattttcggggcatgagctggaataattggtacctcattaagtattttaattcgagctgaattaggacatcctggatcattaattggtgatgaccaaatttataatgtaattgtaacagcacatgcttttgtaataattttctttatagtaatacctattataattggaggatttggaaattgattagttcctttaatattaggagctcctgatatagcatttcctcgaataaataatataagtttttgattacttccgccatcattgactcttttattagccagtagtatagtagaaaatggagctggaactggatgaacagtttacccccctctatcagctgcaattgctcatggaggaggatcagttgatttagcaattttttccctacatttagcaggaatttcatctattttaggagctgttaattttattactactgtaattaatatacgatcaacaggagtaacttttgaccgaatacctttatttgtatgagcagtagtgattacagctattcttctattattatcattaccagtattagctggagctattactatacttttaaccgat---------------------------------------------------------------------

>CNWBA607-13|Hybomitra lurida|COI-5P|KP049217

aacattatattttattttcggggcatgagctggaataattggtacctcattaagtattttaattcgagctgaattaggacatcctggatcattaattggtgatgaccaaatttataatgtaattgtaacagcacatgcttttgtaataattttctttatagtaatacctattataattggaggatttggaaattgattagttcctttaatattaggagctcctgatatagcatttcctcgaataaataatataagtttttgattacttccgccatcattgactcttttattagccagtagtatagtagaaaatggagctggaactggatgaacagtttacccccctctatcagctgcaattgctcatggaggaggatcagttgatttagcaattttttccctacatttagcaggaatttcatctattttaggagctgttaattttattactactgtaattaatatacgatcaacaggagtaacttttgaccgaatacctttatttgtatgagcagtagtgattacagctattcttctattattatcattaccagtattagctggagctattactatacttttaactgatcgaaatttaaatacttccttctttgacccagcaggaggagg----------------------------

>CNWBA584-13|Hybomitra lurida|COI-5P|KP045125

aacattatattttattttcggggcatgagctggaataattggtacctcattaagtattttaattcgagctgaattaggacatcctggatcattaattggtgatgaccaaatttataatgtaattgtaacagcacatgcttttgtaataattttctttatagtaatacctattataattggaggatttggaaattgattagttcctttaatattaggagctcctgatatagcatttcctcgaataaataatataagtttttgattacttccgccatcattgactcttttattagccagtagtatagtagaaaatggagctggaactggatgaacagtttacccccctctatcagctgcaattgctcatggaggaggatcagttgatttagcaattttttccctacatttagcaggaatttcatctattttaggagctgttaattttattactactgtaattaatatacgatcaacaggagtaacttttgaccgaatacctttatttgtatgagcagtagtgattacagctattcttctattattatcattaccagtattagctggagctattactatacttttaactgatcgaaatttaaatacttccttctttgacccagcaggaggagg----------------------------

>JWDCI562-10|Hybomitra lurida|COI-5P|JF877490

aacattatattttattttcggggcatgagctggaataattggtacctcattaagtattttaattcgagctgaattaggacatcctggatcattaattggtgatgaccaaatttataatgtaattgtaacagcacatgcttttgtaataattttctttatagtaatacctattataattggaggatttggaaattgattagttcctttaatattaggagctcctgatatagcatttcctcgaataaataatataagtttttgattacttccgccatcattgactcttttattagccagtagtatagtagaaaatggagctggaactggatgaacagtttacccccctctatcagctgcaattgctcatggaggaggatcagttgatttagcaattttttccctacatttagcaggaatttcatctattttaggagctgttaattttattactactgtaattaatatacgatcaacaggagtaacttttgaccgaatacctttatttgtatgagcagtagtgattacagctattcttctattattatcattaccagtattagctggagctattactatacttttaactgatcgaaatttaaatacttccttctttgacccagcaggaggaggtgancctattttatatcaacatttattt

>JWDCC230-10|Hybomitra lurida|COI-5P|HM861444

aacattatattttattttcggggcatgagctggaataattggtacctcattaagtattttaattcgagctgaattaggacatcctggatcattaattggtgatgaccaaatttataatgtaattgtaacagcacatgcttttgtaataattttctttatagtaatacctattataattggaggatttggaaattgattagttcctttaatattaggagctcctgatatagcatttcctcgaataaataatataagtttttgattacttccgccatcattgactcttttattagccagtagtatagtagaaaatggagctggaactggatgaacagtttacccccctctatcagctgcaattgctcatggaggaggatcagttgatttagcaattttttccctacatttagcaggaatttcatctattttaggagctgttaattttattactactgtaattaatatacgatcaacaggagtaacttttgaccgaatacctttatttgtatgagcagtagtaattacagctattcttctattattatcattaccagtattagctggagctattactatacttttaaccgatcgaaatttaaatacttccttctttgacccagcaggaggaggtgatcctattttatatcaacatttattt

>CNWBE815-13|Hybomitra lurida|COI-5P|KP042174

aacattatattttattttcggggcatgagctggaataattggtacctcattaagtattttaattcgagctgaattaggacatcctggatcattaattggtgatgaccaaatttataatgtaattgtaacagcacatgcttttgtaataattttctttatagtaatacctattataattggaggatttggaaattgattagttcctttaatattaggagctcctgatatagcatttcctcgaataaataatataagtttttgattacttccgccntcattgactcttttattagccagtagtatagtagaaaatggagctggaactggatgaacagtttacccccctctatcagctgcaattgctcatggaggaggatcagttgatttagcaattttttccctacatttagcaggaatttcatctattttaggagctgttaattttattactactgtaattaatatacgatcaacaggagtaacttttgaccgaatacctttatttgtatgagcagtagtgattacagctattcttctattattatcattaccagtattagctggagctattac-------------------------------------------------------------------------------------

>JWDCC439-10|Hybomitra lurida|COI-5P|HM861634

aacattatattttattttcggggcatgagctggaataattggtacctcattaagtattttaattcgagctgaattaggacatcctggatcattaattggtgatgaccaaatttataatgtaattgtaacagcacatgcttttgtaataattttctttatagtaatacctattataattggaggattcggaaattgattagttcctttaatattaggagctcctgatatggcatttcctcgaataaataatataagtttttgattacttccaccgtcattgactcttttattagccagtagtatagtagaaaatggagctggaactggatgaacagtttacccccctctatcagctgcaattgctcatggaggaggatcagttgatttagcaattttttccctacatttagcaggaatttcatctattttaggagctgttaattttattactactgtaattaatatacgatcaacaggagtaacttttgaccgaatacctttatttgtatgagcagtagtgattacagctattcttctattattatcattaccagtattagctggagctattactatacttttaaccgatcgaaatttaaatacttccttctttgacccagcaggaggaggggatcctattttatatcaacatttattt

>JWDCF510-10|Hybomitra lurida|COI-5P|JF875305

aacattatattttattttcggggcatgagctggaataattggtacctcattaagtattttaattcgagctgaattaggacatcctggatcattaattggtgatgaccaaatttacaatgtaattgtaacagcacatgcttttgtaataattttctttatagtaatacctattataattggaggatttggaaattgattagttcctttaatattaggagctcctgatatggcatttcctcgaataaataatataagtttttgattacttccaccgtcattgactcttttattagccagtagtatagtagaaaatggagctggaactggatgaacagtttacccccctctatcagctgcaattgctcatggaggaggatcagttgatttagcaattttttccctacatttagcaggaatttcatctattttaggagctgttaattttattactactgtaattaatatacgatcaacaggagtaacttttgaccgaatacctttatttgtatgagcagtagtgattacagctattcttctattattatcattaccagtattagctggagctattactatacttttaaccgatcgaaatttaaatacttccttctttgacccagcaggaggaggggatcctattttatatcaacatttattt

>MHTAB150-09|Hybomitra lurida|COI-5P|KM285719

----------------------------------------ggtacctcattaagtattttaattcgagctgaattaggacatcctggatcattaattggtgatgaccaaatttataatgtaattgtaacagcacatgcttttgtaataattttctttatagtaatacctattataattggaggatttggaaattgattagttcctttaatattaggagctcctgatatagcatttcctcgaataaataatataagtttttgattacttccgccatcattgactcttttattagccagtagtatagtagaaaatggagctggaactggatgaacagtttacccccctctatcagctgcaattgctcatggaggaggatcagttgatttagcaattttttccctacatttagcaggaatttcatctattttaggagctgttaattttattactactgtaattaatatacgatcaacaggagtaacttttgaccgaatacctttatttgtatgagcagtagtgattacagctattcttctattattatcattaccagtattagctggagctattactatacttttaactgatcgaaatttaaatacttccttctttgacccagcaggaggaggtgatcctattttatatcaacatttattt

>ACT073-07|Hybomitra lurida|COI-5P|KM285722

----------------------------------------ggtacctcattaagtattttaattcgagctgaattaggacatcctggatcattaattggtgatgaccaaatttataatgtaattgtaacagcacatgcttttgtaataattttctttatagtaatacctattataattggaggattcggaaattgattagttcctttaatattaggagctcctgatatggcatttcctcgaataaataatataagtttttgattacttccaccatcattgactcttttattagccagtagtatagtagaaaatggagctggaactggatgaacagtttacccccctctatcagctgcaattgctcatggaggaggatcagttgatttagcaattttttccctacatttagcaggaatttcatctattttaggagctgttaattttattactactgtaattaatatacgatcaacaggagtaacttttgaccgaatacctttatttgtatgagcagtagtgattacagctattcttctattattatcattaccagtattagctggagctattactatacttttaaccgatcgaaatttaaatacttccttctttgacccagcaggaggaggggatcctattttatatcaacatttattt

>UAMIC969-13|Hybomitra lurida|COI-5P|KU875226

aacattatattttattttcggggcatgagctggaataattggtacctcattaagtattttaattcgagctgaattaggacatcctggatcattaattggtgatgaccaaatttataatgtaattgtaacagcacatgcttttgtaataattttctttatagtaatacctattataattggaggatttggaaattgattagttcctttaatattaggagctcctgatatggcatttcctcgaataaataatataagtttttgattacttccaccatcactgactcttttattagccagtagtatagtagaaaatggggctggaactggatgaacagtttacccccctctatcagctgcaattgctcatggaggaggatcagttgatttagcaattttttcnctacatttagcaggaatttcttctattttaggagctgttaattttattactactgtaattaatatacgatcaacaggagtaacttttgaccgaatacctttatttgtatgagcagtagtaattacagctattcttctattattatcattaccagtattagctggagctattactatacttttaactgatcgaaatttaaatacttcnttctttgacccagcaggaggaggagatcctattttatatcaacatttattt

>CNNHA227-14|Hybomitra lurida|COI-5P|KR502182

----------tttattttcggggcatgagctggaataattggtacctcattaagtattttaattcgagctgaattaggacatcctggatcattaattggtgatgaccaaatttataatgtaattgtaacagcacatgcttttgtaataattttctttatagtaatacctattataattggaggatttggaaattgattagttcctttaatattaggagctcctgatatagcatttcctcgaataaataatataagtttttgattacttccgccatcattgactcttttattagccagtagtatagtagaaaatggagctggaactggatgaacagtttacccccctctatcagctgcaattgctcatggaggaggatcagttgatttagcaattttttccctacatttagcaggaatttcatctattttaggagctgttaattttattactactgtaattaatatacgatcaacaggagtaacttttgaccgaatacctttatttgtatgagcagtagtgattacagctattcttctattattatcattaccagtattagctggagctattactatacttttaactgat---------------------------------------------------------------------

>CNNHA007-14|Hybomitra lurida|COI-5P|KR514248

-------------attttcggggcatgagctggaataattggtacctcattaagtattttaattcgagctgaattaggacatcctggatcattaattggtgatgaccaaatttataatgtaattgtaacagcacatgcttttgtaataattttctttatagtaatacctattataattggaggatttggaaattgattagttcctttaatattaggagctcctgatatagcatttcctcgaataaataatataagtttttgattacttccgccatcattgactcttttattagccagtagtatagtagaaaatggagctggaactggatgaacagtttacccccctctatcagctgcaattgctcatggaggaggatcagttgatttagcaattttttccctacatttagcaggaatttcatctattttaggagctgttaattttattactactgtaattaatatacgatcaacaggagtaacttttgaccgaatacctttatttgtatgagcagtagtgattacagctattcttctattattatcattaccagtattagctggagctattactatacttttaactgat---------------------------------------------------------------------

>CNNHA258-14|Hybomitra lurida|COI-5P|KR507760

-------------attttcggggcatgagctggaataattggtacctcattaagtattttaattcgagctgaattaggacatcctggatcattaattggtgatgaccaaatttataatgtaattgtaacagcacatgcttttgtaataattttctttatagtaatacctattataattggaggatttggaaattgattagttcctttaatattaggagctcctgatatagcatttcctcgaataaataatataagtttttgattacttccgccatcattgactcttttattagccagtagtatagtagaaaatggagctggaactggatgaacagtttacccccctctatcagctgcaattgctcatggaggaggatcagttgatttagcaattttttccctacatttagcaggaatttcatctattttaggagctgttaattttattactactgtaattaatatacgatcaacaggagtaacttttgaccgaatacctttatttgtatgagcagtagtgattacagctattcttctattattatcattaccagtattagctggagctattactatacttttaactgat---------------------------------------------------------------------

>SSKJB1137-14|Hybomitra lurida|COI-5P|MF836741

-------------attttcggggcatgagctggaataattggtacctcattaagtattttaattcgagctgaattaggacatcctggatcattaattggtgatgaccaaatttataatgtaattgtaacagcacatgcttttgtaataattttctttatagtaatacctattataattggaggatttggaaattgattagttcctttaatattaggagctcctgatatagcatttcctcgaataaataatataagtttttgattacttccaccgtcattgactcttttattagccagtagtatagtagaaaatggagctggaactggatgaacagtttacccccctctatcagctgcaattgctcatggaggaggatcagttgatttagcaattttttccctacatttagcaggaatttcatctattttaggagctgttaattttattactactgtaattaatatacgatcaacaggagtaacttttgaccgaatacctttatttgtatgagcagtagtgattacagctattcttctattattatcattaccagtattagctgga---------------------------------------------------------------------------------------------

>CNWBE823-13|Hybomitra lurida|COI-5P|KP042501

---------ttttattttcggggcatgagctggaataattggtacctcattaagtattttaattcgagctgaattaggacatcctggatcattaattggtgatgaccaaatttataatgtaattgtaacagcacatgcttttgtaataattttctttatagtaatacctattataattggaggatttggaaattgattagttcctttaatattaggagctcctgatatagcatttcctcgaataaataatataagtttttgattacttccgccatcattgactcttttattagccagtagtatagtagaaaatggagctggaactggatgaacagtttacccccctctatcagctgcaattgctcatggaggaggatcagttgatttagcaattttttccctacatttagcaggaatttcatctattttaggagctgttaattttattactactgtaattaatatacgatcaacaggagtaacttttgaccgaatacctttatttgtatgagcagtagtgattacagctattcttctattattatcattaccagtattagctggagctattactatacttttaactgatcga------------------------------------------------------------------

>CNWBE833-13|Hybomitra lurida|COI-5P|KP045308

aacattatattttattttcggggcatgagctggaataattggtacctcattaagtattttaattcgagctgaattaggacatcctggatcattaattggtgatgaccaaatttataatgtaattgtaacagcacatgcttttgtaataattttctttatagtaatacctattataattggaggatttggaaattgattagttcctttaatattaggagctcctgatatagcatttcctcgaataaataatataagtttttgattacttccaccgtcattgactcttttattagccagtagtatagtagaaaatggagctggaactggatgaacagtttacccccctctatcagctgcaattgctcatggaggaggatcagttgatttagcaattttttccctacatttagcaggaatttcatctattttaggagctgttaattttattactactgtaattaatatacgatcaacaggagtaacttttgaccgaatacctttatttgtatgagcagtagtgattacagctattcttctattattatcattaccagta------------------------------------------------------------------------------------------------------

>FIDIP387-11|Hybomitra bimaculata|COI-5P

aacattatattttattttcggggcatgagctggaataattggtacttcattaagtattttaattcgagctgaattaggacatcctggatcattaattggtgatgaccaaatttataatgtaattgtaacagcacatgcttttgtaataattttttttatagtaatacctattataattggaggatttggaaattgattagttcctttaatattaggagctcctgatatagcatttcctcgaataaataatataagtttttgattacttccaccatcactaactcttttattagccagtagtatagtagaaaatggagctggaactggatgaacagtttatccaccactatcagctgcaattgctcatggaggaggatcagttgatttagcaattttttctttacatttagcaggaatttcttctattttaggagctgttaattttattactactgtaattaatatacgatcaacaggagttacttttgatcgaatacctctatttgtatgagcagtagtaattactgctattcttctattattatcattaccagtattagctggagctattactatacttttaactgatcgaaatttaaatacttcattctttgacccagcaggaggaggagatcctattctatatcaacatttattt

>CROBB307_SK-23_Hybomitra bimaculata

aacattatattttattttcggggcatgagctggaataattggtacttcattaagtattttaattcgagctgaattagggcatcctggatcattaattggtgatgaccaaatttataatgtaattgtaacagcacatgcttttgtaataattttttttatagtaatacctattataattggaggatttggaaattgattagttcctttaatattaggagctcctgatatagcatttcctcgaataaataatataagtttttgattacttccaccatcactaactcttttattagccagtagtatagtagaaaatggagctggaactggatgaacagtttatccaccactatcagctgcaattgctcatggaggaggatcagttgatttagcaattttttctttacatttagcaggaatttcttctattttaggagctgttaattttattactactgtaattaatatacgatcaacaggagttacttttgatcgaatacctctatttgtatgagcagtagtaattactgctattcttctattattatcattaccagtattagctggagctattactatacttttaactgatcgaaatttaaatacttcattctttgacccagcaggaggaggagatcctattctatatcaacatttattt

>FIDIP958-12|Hybomitra bimaculata|COI-5P

aacattatattttattttcggagcatgagctggaataattggtacttcattaagtattttaattcgagctgaattagggcatcctggatcattaattggtgatgaccaaatttacaatgtaattgtaacagcacatgcttttgtaataattttttttatagtaatacctattataattggaggatttggaaattgattagttcctttaatattaggagctcctgatatagcatttcctcgaataaataatataagtttttgattacttccaccatcactaactcttttattagccagtagtatagtagaaaatggagctggaactggatgaacagtttatccaccactatcagctgcaattgctcatggaggaggatcagttgatttagcaattttttctttacatttagcaggaatttcttctattttaggagctgttaattttattactactgtaattaatatacgatcaacaggagttacttttgatcgaatacctctatttgtatgagcagtagtaattactgctattcttctattattatcattaccagtattagctggagctattactatacttttaactgatcgaaatttaaatacttcattctttgacccagcaggaggaggagatcctattctatatcaacatttattt

>TIPNO310-19|Hybomitra cf. distinguenda|COI-5P

aacattatattttattttcggggcatgagctggaataattggtacttcattaagtattttaattcgagctgaattagggcatcctggatcattaattggtgatgaccaaatttataatgtaattgtaacagcacatgcttttgtaataattttttttatagtaatacctattataattggaggatttggaaactgattagttcctttaatattaggagctcctgatatagcatttcctcgaataaataatataagtttttgattacttccaccatcactaactcttttattagccagtagtatagtagaaaatggagctggaactggatgaacagtttatccaccactatcagctgcaattgctcatggaggaggatcagttgatttagcaattttttctttacatttagcaggaatttcttctattttaggagctgttaattttattactactgtaattaatatacgaccaacaggagttacttttgatcgaatacctctatttgtatgagcagtagtaattactgctattcttctattattatcattaccagtattagctggagctattactatacttttaactgatcgaaatttaaatacttcattctttgacccagcaggaggaggagatcctattctatatcaacatttattt

>FIDIP210-11|Hybomitra bimaculata|COI-5P

aacattatattttattttcggggcatgagctggaataattggtacttcattaagtattttaattcgagctgaattagggcatcctggatctttaattggtgatgaccaaatttataatgtaattgtaacagcacatgcttttgtaataattttttttatagtaatacctattataattggaggatttggaaattgattagttcctttaatattaggagctcctgatatagcatttcctcgaataaataatataagtttttgattacttccaccatcactaactcttttattagccagtagtatagtagaaaatggagctggaactggatgaacagtttatccaccactatcagctgcaattgctcatggaggaggatcagttgatttagcaattttttctttacatttagcaggaatttcttctattttaggagctgttaattttattactactgtaattaatatacgatcaacgggagttactttcgatcgaatacctctatttgtatgagcagtagtaattactgctattcttctattattatcattaccagtattagctggagctattactatacttttaactgatcgaaatttaaatacttcattctttgacccagcaggaggaggagatcctattctatatcaacatttattt

>GMFID690-12|Hybomitra epistates|COI-5P

aacattatattttattttcggggcatgagctggaataattggtacttcattaagtattttaattcgagctgaattagggcatcctggatcattaattggtgatgaccaaatttataatgtaattgtaacagcacatgcttttgtaataattttttttatagtaatacctattataattggaggatttggaaattgattagttcctttaatattaggagctcctgatatagcatttcctcgaataaataatataagtttttgattacttccaccatcactaactcttttattagccagtagtatagtagaaaatggagctggaactggatgaacagtttatccaccactatcagctgcaattgctcatggaggaggatcagttgatttagcaattttttctttacatttagcaggaatttcttctattttaggagctgttaattttattactactgtaattaatatacgatcaacaggagttacttttgatcgaatacctctatttgtatgagcagtagtaattactgctattcttctattattatcattaccagtattagctggagctattactatacttttaactgatcgaaatttaaatacttcattctttgacccagcaggaggaggagat------------------------

>GMFIO811-13|Hybomitra epistates|COI-5P

aacattatattttattttcggggcatgagctggaataattggtacttcattaagtattttaattcgagctgaattagggcatcctggatctttaattggtgatgaccaaatttataatgtaattgtaacagcacatgcttttgtaataattttttttatagtaatacctattataattggaggatttggaaattgattagttcctttaatattaggagctcctgatatagcatttcctcgaataaataatataagtttttgattacttccaccatcactaactcttttattagccagtagtatagtagaaaatggagctggaactggatgaacagtttatccaccactatcagctgcaattgctcatggaggaggatcagttgatttagcaattttttctttacatttagcaggaatttcttctattttaggagctgttaattttattactactgtaattaatatacgatcaacaggagttacttttgatcgaatacctctatttgtatgagcagtagtaattactgctattcttctattattatcattaccagtattagctggagctattactatacttttaactgatcgaaatttaaatacttcattctttgacccagcaggaggaggag--------------------------

>NOBRA484-15|Diptera|COI-5P

aacattatattttattttcggggcgtgagctggaataattggtacttcattaagtattttaattcgagctgaattaggacatcctggatcattaattggtgatgaccaaatttacaatgtaattgtaacagcacatgcttttgtaataatttttttcatagtaatacctattataattggaggatttggaaattgattagttcctttaatattaggagctcctgatatagcattccctcgaataaataatataagtttttgattacttccgccatcattaactcttttattagccagtagtatagtagaaaatggagctggaactggatgaacagtttatccacccctatcagctgcaattgctcatggaggaggatcagttgatttagcaattttttctttacatttagcaggaatttcttctattttaggagctgttaattttattactactgtaattaatatacgatcaacaggagttacttttgaccgaatacctctatttgtatgagcagtagtaattactgctattcttctattattatcattaccagtattagctggagctattactatacttttaactgatcgaaatttaaatacttcattctttgacccagcaggaggaggagatcctattctatatcaacatttattt

>NOBRA422-15|Diptera|COI-5P

aacattatattttattttcggggcgtgagctggaataattggtacttcattaagtattttaattcgagctgaattaggacatcctggatcattaattggtgatgaccaaatttacaatgtaattgtaacagcacatgcttttgtaataatttttttcatagtaatacctattataattggaggatttggaaattgattagttcctttaatattaggagctcctgatatagcattccctcgaataaataatataagtttttgattacttccgccatcattaactcttttattagccagtagtatagtagaaaatggagctggaactggatgaacagtttatccacccctatcagctgcaattgctcatggaggaggatcagttgatttagcaattttttctttacatttagcaggaatttcttctattttaggagctgttaattttattactactgtaattaatatacgatcaacaggagttacttttgaccgaatacctctatttgtatgagcagtagtaattactgctattcttctattattatcattaccagtattagctggagctattactatacttttaactgatcgaaatttaaatacttcattctttgacccagcaggaggaggagatcctattctatatcaacatttattt

>NOBRA487-15|Diptera|COI-5P

----------------ttcggggcgtgagctggaataattggtacttcattaagtattttaattcgagctgaattaggacatcctggatcattaattggtgatgaccaaatttacaatgtaattgtaacagcacatgcttttgtaataatttttttcatagtaatacctattataattggaggatttggaaattgattagttcctttaatattaggagctcctgatatagcattccctcgaataaataatataagtttttgattacttccgccatcattaactcttttattagccagtagtatagtagaaaatggagctggaactggatgaacagtttatccacccctatcagctgcaattgctcatggaggaggatcagttgatttagcaattttttctttacatttagcaggaatttcttctattttaggagctgttaattttattactactgtaattaatatacgatcaacaggagttacttttgaccgaatacctctatttgtatgagcagtagtaattactgctattcttctattattatcattaccagtattagctggagctattactatacttttaactgatcgaaatttaaatacttcattctttgacccagcaggaggaggagatcctattctatatcaacatttattt

>CROBB308_SK-24_Hybomitra ciureai

aacattatattttattttcggggcatgagctggaataattggtacttcattaagtattttaattcgagctgaattaggacatcctggatcattaattggtgatgaccaaatttataatgtaattgtaacagcacatgcttttgtaataattttctttatagtaatacctattataattggaggatttggaaattgattagttcctttaatattaggggctcctgatatagcattccctcgaataaataatataagtttttgattacttcccccatcactaactcttttattagccagtagtatagtagaaaatggagctgggactggatgaacagtttaccctcctctatcagctgcaattgcccatggaggtggatcagttgatttagcaattttttctttacatttagcaggaatctcttctattttaggagctgttaattttattactactgtaattaatatacgatcaacaggagttacttttgatcgaatacctctatttgtatgagcagtagtaattactgctattcttttattattatcactaccagtattagctggagctattactatacttttaactgatcgaaatttaaatacttcattctttgacccagcaggaggaggagatcctatcttatatcaacatttattt

>NOBRA492-15|Diptera|COI-5P

aacattatattttattttcggagcatgagctggaataattggtacttcattaagtattttaattcgagctgaattaggacatcctggatcattaattggtgatgaccaaatttataatgtaattgtaacagcacatgcttttgtaataattttctttatagtaatacctattataattggaggatttggaaattgattagttcctttaatattaggagctcctgatatagcatttcctcgaataaataatataagtttttgattacttccgccatcactaactcttctattagccagtagtatagtagaaaatggagctggaactggatgaacagtttacccacctctatcagctgcaattgctcatggaggaggatcagttgatttagcaattttctctttacatttagcaggaatttcttctattttaggagctgttaattttattactactgttattaatatacgatcaacaggtgttacttttgaccgaatacctttatttgtatgagctgtagtaattaccgctattcttctattattgtcattaccagtattagctggagctattactatacttttaactgatcgaaatttaaatacttcattctttgacccagcaggaggaggagacccaattttatatcaacatttattt

>FIDIP2972-12|Hybomitra muehlfeldi|COI-5P

aacattatattttattttcggagcatgagctggaataattggtacttcattaagtattttaattcgagctgaattaggacatcctggatcattaattggtgatgaccaaatttataatgtaattgtaacagcacatgcttttgtaataattttctttatagtaatacctattataattggaggatttggaaattgattagttcctttaatattaggagctcctgatatagcatttcctcgaataaataatataagtttttgattacttccgccatcactaactcttctattagccagtagtatagtagaaaatggagctggaactggatgaacagtttacccacctctatcagctgcaattgctcatggaggaggatcagttgatttagcaattttctctttacatttagcaggaatttcttctattttaggagctgttaattttattactactgttattaatatacgatcaacaggtgttacttttgaccgaatacctttatttgtatgagctgtagtaattaccgctattcttctattattgtcattaccagtattagctggagctattactatacttttaactgatcgaaatttaaatacttcattctttgacccagcaggaggaggagacccaattttatatcaacatttattt

>NOBRA294-15|Diptera|COI-5P

aacattatattttattttcggggcatgagctggaataattggtacttcattaagtattttaattcgagctgaattaggacatcctggatcattaattggtgatgaccaaatttataatgtaattgtaacagcacatgcttttgtaataattttctttatagtaatacctattataattggaggatttggaaattgattagttcctttaatattaggagctcctgatatagcatttcctcgaataaataatataagtttttgattacttccgccatcactaactcttctattagccagtagtatagtagaaaatggagctggaactggatgaacagtttacccacctctatcagctgcaattgctcatggaggaggatcagttgatttagcaattttctctttacatttagcaggaatttcttctattttaggagctgttaattttattactactgttattaatatacgatcaacaggtgttacttttgaccgaatacctttatttgtatgagccgtagtaattaccgctattcttctattattgtcattaccagtattagctggagctattactatacttttaactgatcgaaatttaaatacttcattctttgacccagcaggaggaggagacccaattttatatcaacatttattt

>FIDIP308-11|Hybomitra muehlfeldi|COI-5P

aacattatattttattttcggggcatgagctggaataattggtacttcattaagtattttaattcgagctgaattaggacatcctggatcattaattggtgatgaccaaatttataatgtaattgtaacagcacatgcttttgtaataattttctttatagtaatacctattataattggaggatttggaaattgattagttcctttaatattaggagctcctgatatagcatttcctcgaataaataatataagtttttgattacttccgccatcactaactcttctattagccagtagtatagtagaaaatggagctggaactggatgaacagtttatccacctctatcagctgcaattgctcatggaggaggatcagttgatttagcaattttctctttacatttagcaggaatttcttctattttaggagctgttaattttattactactgttattaatatacgatcaacaggtgttacttttgaccgaatacctttatttgtatgagccgtagtaattaccgctattcttctattactatcattaccagtattagctggagctattactatacttttaactgatcgaaatttaaatacttcattctttgacccagcaggaggaggagacccaattttatatcaacatttattt

>FIDIP2971-12|Hybomitra muehlfeldi|COI-5P

aacattatattttattttcggggcatgagctggaataattggtacttcattaagtattttaattcgagctgaattaggacatcctggatcattaattggtgatgaccaaatttataatgtaattgtaacagcacatgcttttgtaataattttctttatagtaatacctattataattggaggatttggaaattgattagttcctttaatattaggagctcctgatatagcatttcctcgaataaataatataagtttttgattacttccgccatcactaactcttctattagccagtagtatagtagaaaatggagctggaactggatgaacagtttatccacctctatcagctgcaattgctcatggaggaggatcagttgatttagcaattttctctttacatttagcaggaatttcttctattttaggagctgttaattttattactactgttattaatatacgatcaacaggtgttacttttgaccgaatacctttatttgtatgagccgtagtaattaccgctattcttctattactatcattaccagtattagctggagctattactatacttttaactgatcgaaatttaaatacttcattctttgacccagcaggaggaggagacccaattttatatcaacatttattt

>FIDIP2679-12|Hybomitra muehlfeldi|COI-5P

aacattatattttattttcggggcatgagctggaataattggtacttcattaagtattttaattcgagctgaattaggacatcctggatcattaattggtgatgaccaaatttataatgtaattgtaacagcacatgcttttgtaataattttctttatagtaatacctattataattggaggatttggaaattgattagttcctttaatattaggagctcctgatatagcatttcctcgaataaataatataagtttttgattacttccgccatcactaactcttctattagccagtagtatagtagaaaatggagctggaactggatgaacagtttatccacctctatcagctgcaattgctcatggaggaggatcagttgatttagcaattttctctttacatttagcaggaatttcttctattttaggagctgttaattttattactactgttattaatatacgatcaacaggtgttacttttgaccgaatacctttatttgtatgagccgtagtaattaccgctattcttctattactatcattaccagtattagctggagctattactatacttttaactgatcgaaatttaaatacttcattctttgacccagcaggaggaggagacccaattttatatcaacatttattt

>CROBB310_SK-26_Hybomitra muehlfeldi

aacattatattttattttcggggcatgagctggaataattggtacttcattaagtattttaattcgagctgaattaggacatcctggatcattaattggtgatgaccaaatttataatgtaattgtaacagcacatgcttttgtaataattttctttatagtaatacctattataattggaggatttggaaattgattagttcctttaatattaggagctcctgatatagcatttcctcgaataaataatataagtttttgattacttccgccatcactaactcttctattagccagtagtatagtagaaaatggagctggaactggatgaacagtttatccacctctatcagctgcaattgctcatggaggaggatcagttgatttagcaattttctctttacatttagcaggaatttcttctattttaggagctgttaattttattactactgttattaatatacgatcaacaggtgttacttttgaccgaatacctttatttgtatgagccgtagtaattaccgctattcttctattactatcattaccagtattagctggagctattactatacttttaactgatcgaaatttaaatacttcattctttgacccagcaggaggaggagacccaattttatatcaacatttattt

>FIDIP2961-12|Hybomitra solstitialis|COI-5P

--------------------------------------ttggtacctcattaagtatnttaattcgagctgaattaggacatcctggatcattaattggtgatgaccaaatttataatgtaattgtaacagcacatgcttttgtaataattttctttatagtaatacctattataattggagggtttggaaattgattagttcctttaatattaggagctcctgatatagcattccctcgaataaataatataagtttttgattacttcccccatcactaactcttttattagccagtagtatagtagaaaatggggctgggactggatgaacagtttacccccctctatcagctgcaattgcccatggaggaggatcagttgatttagcaattttctccttacatttagcaggaatttcttctattttaggggctgttaattttattactactgtaattaatatacgatcaacaggagttacttttgancgaatacctttatttgtatgagcagtagtaattactgctattcttctattattatcattaccagtattagctggagctattactatacttttaactgatcgaaatcttaatacttcattttttga-------------------------------------------

>FIDIP2978-12|Hybomitra sp.|COI-5P

aacattatattttattttcggggcatgagctggaataattggtacctcattaagtatcttaattcgagctgaattaggacatcctggatcattaattggtgatgaccaaatttataatgtaattgtaacagcacatgcttttgtaataattttctttatagtaatacctattataattggagggtttggaaattgattagttcctttaatattaggagctcctgatatggcattccctcgaataaataatataagtttttgattacttcccccatcactaactcttttattagccagtagtatagtagaaaatggggctgggactggatgaacagtttacccccctctatcagctgcaattgcccatggaggaggatcagttgatttagcaattttttccttacatttagcaggaatttcttctattttaggggctgttaattttattactactgtaattaatatacgatcaacaggagttacttttgancgaatacctttatttgtatgagcagtagtaattactgctattcttctattattatcattaccagtattagctggagctattactatacttttaactgatcgaaatctaaatacttcattttttgacccagcaggaggagg----------------------------

>FIDIP2962-12|Hybomitra solstitialis|COI-5P

aacattatattttattttcggggcatgagctggaataattggtacctcattaagtatcttaattcgagctgaattaggacatcctggatcattaattggtgatgaccaaatttataatgtaattgtaacagcacatgcttttgtaataattttctttatagtaatacctattataattggagggtttggaaattgattagttcctttaatattaggagctcctgatatggcattccctcgaataaataatataagtttttgattacttcccccatcactaactcttttattagccagtagtatagtagaaaatggggctgggactggatgaacagtttacccccctctatcagctgcaattgcccatggaggagggtcagttgatttagcaattttttccttacatttagcaggaatttcttctattttaggggctgttaattttattactactgtaattaatatacgatcaacaggagttacttttgaccgaatacctttatttgtatgagcagtagtaattactgctattcttctattattatcattaccagtattagctggagctattactatacttttaactgatcgaaatctaaatacttcattttttgacccggcaggaggaggagatcctattttatacc-----------

>FIDIP501-11|Hybomitra solstitialis|COI-5P

aacattatattttattttcggggcatgagctggaataattggtacctcattaagtatcttaattcgagctgaattaggacatcctggatcattaattggtgatgaccaaatttataatgtaattgtaacagcacatgcttttgtaataattttctttatagtaatacctattataattggagggtttggaaattgattagttcctttaatattaggagctcctgatatggcattccctcgaataaataatataagtttttgattacttcccccatcactaactcttttattagccagtagtatagtagaaaatggggctgggactggatgaacagtttacccccctctatcagctgcaattgcccatggaggaggatcagttgatttagcaattttttccttacatttagcaggaatttcttctattttaggggctgttaattttattactactgtaattaatatacgatcaacaggagttacttttgaccgaatacctttatttgtatgagcagtagtaattactgctattcttctattattatcattaccagtattagctggagctattactatacttttaactgatcgaaatctaaatacttcattttttgacccggcaggaggaggagatcctattttataccaacacttattt

>CROBB323_SK-39_Hybomitra ukrainica

aacattatattttattttcggggcatgagctggaataattggtacctcattaagtatcttaattcgagctgaattaggacatcctggatcattaattggtgatgaccaaatttataatgtaattgtaacagcacatgcttttgtaataattttctttatagtaatacctattataattggagggtttggaaattgattagttcctttaatattaggagctcctgatatggcattccctcgaataaataatataagtttttgattacttcccccatcactaactcttttattagccagtagtatagtagaaaatggggctgggactggatgaacagtttacccccctctatcagctgcaattgcccatggaggaggatcagttgatttagcaattttttccttacatttagcaggaatttcttctattttaggggctgttaattttattactactgtaattaatatacgatcaacaggagttacttttgaccgaatacctttatttgtatgagcagtagtaattactgctattcttctattattatcattaccagtattagctggagctattactatacttttaactgatcgaaatctaaatacctcattttttgacccggcaggaggaggagatcctattttataccaacacttattt

>FIDIP2977-12|Hybomitra nitidifrons|COI-5P

--cattatattttattttcggggcatgagctggaataattggtacctcattaagtattttaattcgagctgaattaggacatcctggatcattaattggtgatgaccaaatttataacgtaattgtaacagcacatgcttttgtaataattttctttatagtaatacctatcataattggagggtttgggaattgattagttcctttaatattaggagctcctgatatagcattccctcgaataaataatataagtttttgattactcccgccatcactaactcttttattagccagtagtatagtagaaaatggagctgggactggatgaacagtttacccacctctatcagctgcaattgctcatggaggaggatcagttgatttagcaattttttctttacatttagcaggaatttcatctattttaggagctgttaatttcattactactgtaattaatatacgatcaacaggagtaacttttgatcgaatacctctatttgtatgggcagtggtaattacagctattcttctattattatcactaccagtattagccggagctattactatacttttaactgatcgaaatctaaatacttcatt-------------------------------------------------

>NOBRA305-15|Diptera|COI-5P

aacattatattttattttcggggcatgagctggaataattggtacctcattaagtattttaattcgagctgaattaggacatcctggatcattaattggtgatgaccaaatttataacgtaattgtaacagcacatgcttttgtaataattttctttatagtaatacctatcataattggagggtttgggaattgattagttcctttaatattaggagctcctgatatggcattccctcgaataaataatataagtttttgattactcccgccatcactaactcttttattagccagtagtatagtagaaaatggagctgggactggatgaacagtttacccacctctatcagctgcaattgctcatggaggaggatcagttgatttagcaattttttctttacatttagcaggaatttcatctattttaggggctgttaatttcattactactgtaattaatatacgatcaacaggagtaacttttgatcgaatacctctatttgtatgagcagtagtaattacagctattcttctattattatcactaccagtattagccggagctattactatacttttaactgatcgaaatctaaatacttcattttttgacccagcaggaggaggagatcctatcctatatcaacatttattt

>NOBRA292-15|Diptera|COI-5P

aacattatattttattttcggggcatgagctggaataattggtacctcattaagtattttaattcgagctgaattaggacatcctggatcattaattggtgatgaccaaatttataacgtaattgtaacagcacatgcttttgtaataattttctttatagtaatacctatcataattggagggtttgggaattgattagttcctttaatattaggagctcctgatatagcattccctcgaataaataatataagtttttgattactcccgccatcactaactcttttattagccagtagtatagtagaaaatggagctgggactggatgaacagtttacccacctctatcagctgcaattgctcatggaggaggatcagttgatttagcaattttttctttacatttagcaggaatttcatctattttaggagctgttaatttcattactactgtaattaatatacgatcaacaggagtaacttttgatcgaatacctctatttgtatgggcagtggtaattacagctattcttctattattatcactaccagtattagccggagctattactatacttttaactgatcgaaatctaaatacttcattttttgacccagcaggaggaggagatcctatcctatatcaacatttattt

>NOBRA306-15|Diptera|COI-5P

aacattatattttattttcggggcatgagctggaataattggtacctcattaagtattttaattcgagctgaattaggacatcctggatcattaattggtgatgaccaaatttataacgtaattgtaacagcacatgcttttgtaataattttctttatagtaatacctatcataattggagggtttgggaattgattagttcctttaatattaggagctcctgatatagcattccctcgaataaataatataagtttttgattactcccgccatcattaactcttttattagccagtagtatagtagaaaatggagctgggactggatgaacagtttacccacctctatcagctgcaattgctcatggaggaggatcagttgatttagcaattttttctttacatttagcaggaatttcatctattttaggagctgttaatttcattactactgtaattaatatacgatcaacaggagtaacttttgatcgaatacctctatttgtatgagcagtggtaattacagctattcttctattattatcactaccagtattagccggagctattactatacttttaactgatcgaaatctaaatacttcattttttgacccagcaggaggaggagatcctatcctatatcaacatttattt

>NOBRA293-15|Diptera|COI-5P

aacattatattttattttcggggcatgagctggaataattggtacctcattaagtattttaattcgagctgaattaggacatcctggatcattaattggtgatgaccaaatttataacgtaattgtaacagcacatgcttttgtaataattttctttatagtaatacctatcataattggagggtttgggaattgattagttcctttaatattaggagctcctgatatagcattccctcgaataaataatataagtttttgattactcccgccatcactaactcttttattagccagtagtatagtagaaaatggagctgggactggatgaacagtttaccctcctctatcagctgcaattgctcatggaggaggatcagttgatttagcaattttttctttacatttagcaggaatttcatctattttaggagctgttaatttcattactactgtaattaatatacgatcaacaggagtaacttttgatcgaatgcctctatttgtatgagcagtaataattacagctattcttctattattatcactaccagtattagccggagctattactatacttttaactgatcgaaatctaaatacttcattttttgacccagcaggaggaggagatcctatcctatatcaacatttattt

>CROBB309_SK-25_Hybomitra distinguenda

aacattatattttattttcggggcatgagctggaataattggtacctcattaagtattttaattcgagctgaattaggtcatcctggatcattaattggtgatgatcaaatttataatgtaattgtaacagcacatgcttttgtaataattttctttatagtaataccgattataattggagggtttggaaattgattagttcctttaatattaggagctcccgatatagcattccctcgaataaataatataagtttttgattacttccaccatcactaactcttttattagccagtagtatagtagaaaatggggctggaactggatgaacagtttatccacctctatcagctgcaattgctcatggaggaggatcagttgatttagcaattttttctttacatttagcaggaatctcttctattttaggagctgttaattttattactactgtaattaatatacgatcaacaggagttacctttgatcgaatacctttatttgtatgagcagtagtaattactgctattcttttactattatcactcccagtattagccggagctattactatacttttaactgatcgaaatttaaatacttcattctttgacccagcaggaggaggagaccctattctataccaacatttattc

>FIDIP2963-12|Hybomitra distinguenda|COI-5P

aacattatattttattttcggggcatgagctggaataattggtacctcattaagtattttaattcgagctgaattaggtcatcctggatcattaattggtgatgatcaaatttataatgtaattgtaacagcacatgcttttgtaataattttctttatagtaataccgattataattggagggtttggaaattgattagttcctttaatattaggagctcccgatatagcattccctcgaataaataatataagtttttgattacttccaccatcactaactcttttattagccagtagtatagtagaaaatggagctggaactggatgaacagtttatccacctctatcagctgcaattgctcatggaggaggatcagttgatttagcaattttttctttacatttagcaggaatctcttctattttaggagctgttaattttattactactgtaattaatatacgatcaacaggagttacctttgatcgaatacctttatttgtatgagcagtagtaattactgctattcttttattattatcactcccagtattagccggagctattactatacttttaactgatcgaaatttaaatacttcattctttgacccagcagggggaggagatcctattctataccaac--------

>CNNHC203-14|Hybomitra|COI-5P|KR514143

----------tttatttttggagcgtgagctggaataattggtacctcattaagtatcttaattcgagctgaattaggacaccctgggtcattaattggagatgaccaaatttataatgtaattgtaacagcacatgcttttgtaataattttctttatagtaatacctattataattggaggatttggaaattgattagttcctttaatattaggagcccctgatatagcattccctcgaataaataatataagtttctgattacttccaccatcattaactcttttattagccagtagcatagtagaaaatggggctggaactggttgaacagtt---ccccctctatctgctgcaattgctcatggaggaggatcagttgatttagcaattttttctttacacttagcaggaatttcttctattttaggagctgttaattttattactactgtaattaatatacgatcaacaggagtcacttttgatcgaatacctttatttgtatgagcagtagtaattacagctattcttttattattatcactaccagtattagctgga---------------------------------------------------------------------------------------------

>SSPAC1981-13|Hybomitra|COI-5P|KM643087

-acattatattttatttttggagcgtgagctggaataattggtacctcattaagtatcttaattcgagctgaattaggacaccctgggtcattaattggagatgaccaaatttataatgtaattgtaacagcacatgcttttgtaataattttctttatagtaatacctattataattggaggatttggaaattgattagttcctttaatattaggggcccctgatatagcattccctcgaataaataatataagtttctgattacttccgccatcattaactcttttattagccagtagcatagtagaaaatggggctggaactggttgaacagtttacccccctctatctgctgcaattgctcatggaggaggatcagttgatttagcaattttttctttacacttagcaggaatttcttctattttaggagctgttaattttattactactgtaattaatatacgatcaacaggagtcacttttgaccgaatacctttatttgtatgagcagtagtaattacagctattcttttattattatcactaccagtattagctggagctattactatattattaactgat---------------------------------------------------------------------

>JWDCC467-10|Hybomitra arpadi|COI-5P|HM861662

-------------------------------ggaataattggtacctcattaagtatcttaattcgagctgaattaggacaccctgggtcattaattggagatgaccaaatttataatgtaattgtaacagcacatgcttttgtaataattttctttatagtaatacctattataattggaggatttggaaattgattagttcctttaatattaggagcccctgatatagcattccctcgaataaataatataagtttctgattacttccaccatcattaactcttttgttagccagtagcatagtagaaaatggggctggaactggttgaacagtttacccccctctatctgctgcaattgctcatggaggaggatcagttgatttagcaattttttctttacacttagcaggaatttcttctattttaggagctgttaattttattactactgtaattaatatacgatcaacaggagtcacttttgatcgaatacctttatttgtatgagcagtagtaattacagctattcttttattattatcactaccagtattagctggagctattactatattattaactgatcgaaatttaaatacttcattttttgaccctgcaggaggaggagaccctattttatatcaacatttattt

>JWDCC210-10|Hybomitra arpadi|COI-5P|HM861425

aacattatattttatttttggagcgtgagctggaataattggtacctcattaagtatcttaattcgagctgaattaggacaccctgggtcattaattggagatgaccaaatttataatgtaattgtaacagcacatgcttttgtaataattttctttatagtaatacctattataattggaggatttggaaattgattagttcctttaatattaggagcccctgatatagcattccctcgaataaataatataagtttctggttacttccaccatcattaactcttttattagccagtagcatagtagaaaatggggctggaactggttgaacagtttacccccctctatctgctgcaattgctcatggaggaggatcagttgatttagcaattttttctttacacttagcaggaatttcttctattttaggagctgttaattttattactactgtaattaatatacgatcaacaggagtcacttttgatcgaatacctttatttgtatgagcagtagtaattacagctattcttttattattatcactaccagtattagctggagctattactatattattaactgatcgaaatttaaatacttcattttttgaccctgcaggaggaggagaccctattttatatcaacatttattt

>JWDCJ1191-11|Hybomitra arpadi|COI-5P|JN302844

aacattatattttatttttggagcgtgagctggaataattggtacctcattaagtatcttaattcgagctgaattaggacaccctgggtcattaattggagatgaccaaatttataatgtaattgtaacagcacatgcttttgtaataattttctttatagtaatacctattataattggaggatttggaaattgattagttcctttaatattaggagcccctgatatagcattccctcgaataaataatataagtttctgattacttccaccatcattaactcttttattagccagtagcatagtagaaaatggggctggaactggttgaacagtttacccccctctatctgctgcaattgctcatggaggaggatcagttgatttagcaattttttctttacacttagcaggaatttcttctattttaggagctgttaattttattactactgtaattaatatacgatcaacaggagtcacttttgatcgaatacctttatttgtatgagcagtagtaattacagctattcttttattattatcactaccagtattagctggagctattactatattattaactgatcgaaatttaaatacttcattttttgaccctgcaggaggaggagaccctattttatatcaacatttattt

>SAMOS080-09|Hybomitra arpadi|COI-5P|KR755266

aacattatattttatttttggagcgtgagctggaataattggtacctcattaagtatcttaattcgagctgaattaggacaccctgggtcattaattggagatgaccaaatttataatgtaattgtaacagcacatgcttttgtaataattttctttatagtaatacctattataattggaggatttggaaattgattagttcctttaatattaggagcccctgatatagcattccctcgaataaataatataagtttctgattacttccaccatcattaactcttttattagccagtagcatagtagaaaatggggctggaactggttgaacagtttacccccctctatctgctgcaattgctcatggaggaggatcagttgatttagcaattttttctttacacttagcaggaatttcttctattttaggagctgttaattttattactactgtaattaatatacgatcaacaggagtcacttttgatcgaatacctttatttgtatgagcagtagtaattacagctattcttttattattatcactaccagtattagctggagctattactatattattaactgatcgaaatttaaatacttcattttttgaccctgcaggaggaggagaccctattttatatcaacatttattt

>JWDCJ077-11|Hybomitra arpadi|COI-5P|JF877724

aacattatattttatttttggagcgtgagctggaataattggtacctcattaagtatcttaattcgagctgaattaggacaccctgggtcattaattggagatgaccaaatttataatgtaattgtaacagcacatgcttttgtaataattttctttatagtaatacctattataattggaggatttggaaattgattagttcctttaatattaggagcccctgatatagcattccctcgaataaataatataagtttctgattacttccaccatcattaactcttttattagccagtagcatagtagaaaatggggctggaactggttgaacagtttacccccctctatctgctgcaattgctcatggaggaggatcagttgatttagcaattttttctttacacttagcaggaatttcttctattttaggagctgttaattttattactactgtaattaatatacgatcaacaggagtcacttttgatcgaatacctttatttgtatgagcagtagtaattacagctattcttttattattatcactaccagtattagctggagctattactatattattaactgatcgaaatttaaatacttcattttttgaccctgcaggaggaggagaccctattttatatcaacatttattt

>SAMOS075-09|Hybomitra arpadi|COI-5P|KR742955

aacattatattttatttttggagcgtgagctggaataattggtacctcattaagtatcttaattcgagctgaattaggacaccctgggtcattaattggagatgaccaaatttataatgtaattgtaacagcacatgcttttgtaataattttctttatagtaatacctattataattggaggatttggaaattgattagttcctttaatattaggagcccctgatatagcattccctcgaataaataatataagtttctgattacttccaccatcattaactcttttattagccagtagcatagtagaaaatggggctggaactggttgaacagtttacccccctctatctgctgcaattgctcatggaggaggatcagttgatttagcaattttttctttacacttagcaggaatttcttctattttaggagctgttaattttattactactgtaattaatatacgatcaacaggagtcacttttgatcgaatacctttatttgtatgagcagtagtaattacagctattcttttattattatcactaccagtattagctggagctattactatattattaactgatcgaaatttaaatacttcattttttgaccctgcaggaggaggagaccctattttatatcaacatttattt

>JWDCJ1144-11|Hybomitra arpadi|COI-5P|JN302800

aacattatattttatttttggagcgtgagctggaataattggtacctcattaagtatcttaattcgagctgaattaggacaccctggatcattaattggagatgaccaaatttataatgtaattgtaacagcacatgcttttgtaataattttctttatagtaatacctattataattggaggatttggaaattgattagttcctttaatattaggagcccctgatatagcattccctcgaataaataatataagtttctgattacttccgccatcattaactcttttattagccagtagcatagtagaaaatggggctggaactggttgaacagtttacccccctctatctgctgcaattgctcatggaggaggatcagttgatttagcaattttttctttacacttagcaggaatttcttctattttaggagctgttaattttattactactgtaattaatatacgatcaacaggagtcacttttgatcgaatacctttatttgtatgagcagtagtaattacagctattcttttattattatcactaccagtattagctggagctattactatattattaactgatcgaaatttaaatacttcattttttgaccctgcaggaggaggagaccctattttatatcaacatttattt

>JWDCC049-10|Hybomitra arpadi|COI-5P|HQ962048

aacattatattttatttttggagcgtgagctggaataattggtacctcattaagtatcttaattcgagctgaattaggacaccctgggtcattaattggagatgaccaaatttataatgtaattgtaacagcacatgcttttgtaataattttctttatagtaatacctattataattggaggatttggaaattgattagttcctttaatattaggagcccctgatatagcattccctcgaataaataatataagtttctgattacttccgccatcattaactcttttattagccagtagcatagtagaaaatggggctggaactggttgaacagtttacccccctctatctgctgcaattgctcatggaggaggatcagttgatttagcaattttttctttacacttagcaggaatttcttctattttaggagctgttaattttattactactgtaattaatatacgatcaacaggagtcacttttgatcgaatacctttatttgtatgagcagtagtaattacagctattcttttattattatcactaccagtattagctggagctattactatattattaactgatcgaaatttaaatacttcattttttgaccctgcaggaggaggagaccctattttatatcaacatttattt

>JWDCC460-10|Hybomitra arpadi|COI-5P|HM861655

aacattatattttatttttggagcgtgagctggaataattggtacctcattaagtatcttaattcgagctgaattaggacaccctgggtcattaattggagatgaccaaatttataatgtaattgtaacagcacatgcttttgtaataattttctttatagtaatacctattataattggaggatttggaaattgattagttcctttaatattaggggcccctgatatagcattccctcgaataaataatataagtttctgattacttccgccatcattaactcttttattagccagtagcatagtagaaaatggggctggaactggttgaacagtttacccccctctatctgctgcaattgctcatggaggaggatcagttgatttagcaattttttctttacacttagcaggaatttcttctattttaggagctgttaattttattactactgtaattaatatacgatcaacaggagtcacttttgaccgaatacctttatttgtatgagcagtagtaattacagctattcttttattattatcactaccagtattagctggagctattactatattattaactgatcgaaatttaaatacttcattttttgaccctgcaggagggggagaccctattttatatcaacatttattt

>FIDIP408-11|Hybomitra arpadi|COI-5P

aacattatattttatttttggagcgtgggctggaataattggtacctcattaagtatcttaattcgagctgaattaggacaccctgggtcattaattggagatgaccaaatttataatgtaattgtaacagcacatgcttttgtaataattttctttatagtaatacctattataattggaggatttggaaattgattagttcctttaatattaggagcccctgatatagcattccctcgaataaataatataagtttctgattacttccgccatcattaactcttttattagccagtagtatagtagaaaatggggctggaactggttgaacagtttacccccctctatctgctgcaattgctcatggaggaggatcagttgatttagcaattttttctttacacttagccggaatttcttctattttaggagctgttaattttattactactgtaattaatatacgatcaacaggagtcacttttgatcgaatacctttatttgtatgagcagtagtaattacagctattcttttattattatcactaccagtattagctggagctattactatattattaactgatcgaaatttaaatacttcattttttgaccctgcaggaggaggagatcctattttatatcaacatttattt

>JWDCC468-10|Hybomitra arpadi|COI-5P|HM861663

aacattatattttatttttggagcgtgagctggaataattggtacctcattaagtatcttaattcgagctgaattaggacaccctgggtcattaattggagatgaccaaatttataatgtaattgtaacagcacatgcttttgtaataattttctttatagtaatacctattataattggaggatttggaaattgattagttcctttaatattaggagcccctgatatagcattccctcgaataaataatataagtttctgattacttccgccatcattaactcttttattagccagtagtatagtagaaaatggggctggaactggttgaacagtttacccccctctatctgctgcaattgctcatggaggaggatcagttgatttagcaattttttctttacacttagccggaatttcttctattttaggagctgttaattttattactactgtaattaatatacgatcaacaggagtcacttttgatcgaatacctttatttgtatgagcagtagtaattacagctattcttttattgttatcactaccagtattagctggagctattactatattattaactgatcgaaatttaaatacttcattttttgatcctgcaggaggaggagatcctattttatatcaacatttattt

>MHTAB004-07|Hybomitra arpadi|COI-5P|KM285587

aacattatattttatttttggagcgtgagctggaataattggtacctcattaagtatcttaattcgagctgaattaggacaccctgggtcattaattggagatgaccaaatttataatgtaattgtaacagcacatgcttttgtaataattttctttatagtaatacctattataattggaggatttggaaattgattagttcctttaatattaggggcccctgatatagcattccctcgaataaataatataagtttctgattacttccgccatcattaactcttttattagccagtagcatagtagaaaatggggctggaactggttgaacaatttacccccctctatctgctgcaattgctcatggaggaggatcagttgatttagcaattttttctttacacttagcaggaatttcttctattttaggagctgttaattttattactactgtaattaatatacgatcaacaggagtcacttttgaccgaatacctttatttgtatgagcagtagtaattacagctattcttttattattatcactaccagtattagctggagctattactatattattaactgatcgaaatttaaatacttcattttttgaccctgcaggagggggagaccctattttatatcaacatttatt-

>MHTAB011-07|Hybomitra affinis|COI-5P|KM285570

aacattatattttatttttggagcgtgagctggaataattggtacctcattaagtattttaattcgagctgaattaggacatcctggatcattaattggagatgaccaaatttataatgtaattgtaacagcacatgcttttgtaataattttttttatagtaatacctattataattgggggatttggaaattgattagttcctttaatattaggagctcctgatatagcattccctcgaataaataatataagtttctgattacttccaccatcattaactcttttattagccagtagtatagtagaaaatggggctggaactggttgaacagtttacccccctctatctgctgcaattgctcatggaggaggatcagttgatttagcaattttttctttacatttagcaggaatttcttctattttaggggctgttaattttattactactgtaattaatatacgatcaacaggagttacttttgatcgaatacctttatttgtatgagccgtagtaattacagctattcttttattattatcattaccagtattagctggagctattactatactattaactgatcgaaatttaaatacttcattctttgaccc----------------------------------------

>MHTAB010-07|Hybomitra affinis|COI-5P|KM285571

aacattatattttatttttggagcgtgagctggaataattggtacctcattaagtattttaattcgagctgaattaggacatcctggatcattaattggagatgaccaaatttataatgtaattgtaacagcacatgcttttgtaataattttttttatagtaatacctattataattggaggatttggaaattgattagttcctttaatattaggagctcctgatatagcattccctcgaataaataatataagtttctgattactyccaccatcattaactcttttattagccagtagtatagtagaaaatggggctggaactggttgaacagtttacccccctctatctgctgcaattgctcatggaggargatcagttgatttagcaattttttctttacatttagcaggaatttcttctattttaggagctgttaattttattactactgtaattaatatacgatcaacaggagttacttttgatcgaatacctttatttgtatgagccgtagtaattacagctattcttttattattatcattaccagtattagctggagctattactatactattaactgatcgaaatttaaatacttcattctttgaccctgcaggaggaggagaccctattttatatcaacatttatt-

>MHTAB003-07|Hybomitra affinis|COI-5P|KM285572

aacattatattttatttttggagcgtgagctggaataattggtacctcattaagtattttaattcgagctgaattaggacatcctggatcattaattggagatgaccaaatttataatgtaattgtaacagcacatgcttttgtaataattttttttatagtaatacctattataattgggggatttggaaattgattagttcctttaatattaggagctcctgatatagcattccctcgaataaataatataagtttctgattacttccaccatcattaactcttttattagccagtagtatagtagaaaatggggctggaactggttgaacagtttacccccctctatctgctgcaattgctcatggaggaggatcagttgatttagcaattttttctttacatttagcaggaatttcttctattttaggggctgttaattttattactactgtaattaatatacgatcaacaggagttacttttgatcgaatacctttatttgtatgagccgtagtaattacagctattcttttattattatcattaccagtattagctggagctattactatactattaactgatcgaaatttaaatacttcattctttgaccctgcaggaggaggagaccctattttatatcaacatttatt-

>MHTAB082-07|Hybomitra arpadi|COI-5P|KM285590

aacattatattttatttttggagcgtgagctggaataattggtacctcattaagtattttaattcgagctgaattaggacatcctggatcattaattggagatgaccaaatttataatgtaattgtaacagcacatgcttttgtaataattttttttatagtaatacctattataattgggggatttggaaattgattagttcctttaatattaggagctcctgatatagcattccctcgaataaataatataagtttctgattacttccaccatcattaactcttttattagccagtagtatagtagaaaatggggctggaactggttgaacagtttacccccctctatctgctgcaattgctcatggaggaggatcagttgatttagcaattttttctttacatttagcaggaatttcttctattttaggggctgttaattttattactactgtaattaatatacgatcaacaggagttacttttgatcgaatacctttatttgtatgagccgtagtaattacagctattcttttattattatcattaccagtattagctggagctattactatactattaactgatcgaaatttaaatacttcattctttgaccctgcaggaggaggagaccctattttatatcaacatttatt-

>JWDCB033-10|Hybomitra affinis|COI-5P|HM861001

aacattatattttatttttggagcgtgagctggaataattggtacctcattaagtattttaattcgagctgaattaggacatcctggatcattaattggagatgaccaaatttataatgtaattgtaacagcacatgcttttgtaataattttttttatagtaatacctattataattgggggatttggaaattgattagttcctttaatattaggggctcctgatatagcattccctcgaataaataatataagtttctgattacttccaccatcattaactcttttattagccagtagtatagtagaaaatggggctggaactggttgaacagtttacccccctctatctgctgcaattgctcatggaggaggatcagttgatttagcaattttttctttacatttagcaggaatttcttctattttaggggctgttaattttattactactgtaattaatatacgatcaacaggagttacttttgatcgaatacctttatttgtatgagccgtagtaattacagctattcttttattattatcattaccagtattagctggagctattactatactattaactgatcgaaatttaaatacttcattctttgaccctgcaggaggaggagaccctattttatatcaacatttattt

>SAMOS081-09|Hybomitra affinis|COI-5P|MG166449

aacattatattttatttttggagcgtgagctggaataattggtacctcattaagtattttaattcgagctgaattaggacatcctggatcattaattggagatgaccaaatttataatgtaattgtaacagcacatgcttttgtaataattttttttatagtaatacctattataattgggggatttggaaattgattagttcctttaatattaggagctcctgatatagcattccctcgaataaataatataagtttctgattacttccaccatcattaactcttttattagccagtagtatagtagaaaatggggctggaactggttgaacagtttacccccctctatctgctgcaattgctcatggaggaggatcagttgatttagcaattttttctttacatttagcaggaatttcttctattttaggggctgttaattttattactactgtaattaatatacgatcaacaggagttacttttgatcgaatacctttatttgtatgagccgtagtaattacagctattcttttattattatcattaccagtattagctggagctattactatactattaactgatcgaaatttaaatacttcattctttgaccctgcaggaggaggagaccctattttatatcaacatttattt

>TCAP029-06|Hybomitra arpadi|COI-5P|KM285591

aacattatattttatttttggagcgtgagctggaataattggtacctcattaagtattttaattcgagctgaattaggacatcctggatcattaattggagatgaccaaatttataatgtaattgtaacagcacatgcttttgtaataattttttttatagtaatacctattataattgggggatttggaaattgattagttcctttaatattaggagctcctgatatagcattccctcgaataaataatataagtttctgattacttccaccatcattaactcttttattagccagtagtatagtagaaaatggggctggaactggttgaacagtttacccccctctatctgctgcaattgctcatggaggaggatcagttgatttagcaattttttctttacatttagcaggaatttcttctattttaggngctgttaattttattactactgtaattaatatacgatcaacaggagttacttttgatcgaatacctttatttgtatgagccgtagtaattacagctattcttttattattatcattaccagtattagctggagctattactatactattaactgatcgaaatttaaatacttcattctttgaccctgcaggaggaggagaccctattttatatcaacatttattt

>TCAP026-06|Hybomitra affinis|COI-5P|KM285576

aacattatattttatttttggagcgtgagctggaataattggtacctcattaagtattttaattcgagctgaattaggacatcctggatcattaattggagatgaccaaatttataatgtaattgtaacagcacatgcttttgtaataattttttttatagtaatacctattataattggnggatttggaaattgattagttcctttaatattaggagcncctgatatagcattccctcgaataaataatataagtttctgattacttccaccatcattaactcttttattagccagtagtatagtagaaaatggggctggaactggttgaacagtttacccccctctatctgctgcaattgctcatggaggaggatcagttgatttagcaattttttctttacatttagcaggaatttcttctattttaggngctgttaattttattactactgtaattaatatacgatcaacaggagttacttttgatcgaatacctttatttgtatgagcngtagtaattacagctattcttttattattatcattaccagtattagctggagctattactatactattaactgatcgaaatttaaatacttcattctttgaccctgcaggaggaggagnnnnnnnnnnnnnnnnnnnnnnnnnn

>TCAP027-06|Hybomitra affinis|COI-5P|KM285561

aacattatattttatttttggagcgtgagctggaataattggtacctcattaagtattttaattcgagctgaattaggacatcctggatcattaattggagatgaccaaatttataatgtaattgtaacagcacatgcttttgtaataattttttttatagtaatacctattataattgggggatttggaaattgattagttcctttaatattaggagctcctgatatagcattccctcgaataaataatataagtttctgnttacttccaccatcattaactcttttattagccagtagtatagtagaaaatggggctggaactggttgaacagtttacccccctctatctgctgcaattgctcatggaggaggatcagttgatttagcaattttttctttacatttagcaggaatttcttctattttaggngctgttaattttattactactgtaattaatatacgatcaacaggagttacttttgatcgaatacctttatttgtatgagccgtagtaattacagctattcttttattattatcattaccagtattagctggagctattactatactattaactgatcgaaatttaaatacttcattctttgaccctgcaggaggaggagaccctattttatatcaacatttattt

>UAMIC979-13|Hybomitra arpadi|COI-5P|KU875220

aacattatattttatttttggagcgtgagctggaataattggtacctcattaagtattttaattcgagctgaattaggacatcctggatcattaattggagatgaccaaatttataatgtaattgtaacagcacatgcttttgtaataattttttttatagtaatacctattataattggaggatttggaaattgattagttcctttaatattaggagctcctgatatagcattccctcgaataaataatataagtttctgattactnccaccatcattaactcttttattagccagtagtatagtagaaaatggggctggaactggttgaacagtttacccccctctatctgctgcaattgctcatggaggaggatcagttgatttagcaattttttctttacatttagcaggaatttcttctattttaggggctgttaattttattactactgtaattaatatacgatcaacaggagttacttttgatcgaatacctttatttgtatgagccgtagtaattacagctattcttttattattatcattaccagtattagctggagctattactatactattaactgatcgaaatttaaatacttcattctttgaccctgcaggaggaggagaccctattttatatcaacatttattt

>TCAP032-06|Hybomitra affinis|COI-5P|KM285566

aacattatattttatttttggagcgtgagctggaataattggtacctcattaagtattttaattcgagctgaattaggacatcctggatcattaattggagatgaccaaatttataatgtaattgtaacagcacatgcttttgtaataattttttttatagtaatacctattataattggaggatttggaaattgattagttcctttaatattaggagctcctgatatagcattccctcgaataaataatataagtttctgattactnccaccatcattaactcttttattagccagtagtatagtagaaaatggggctggaactggttgaacagtttacccccctctatctgctgcaattgctcatggaggaggatcagttgatttagcaattttttctttacatttagcaggaatttcttctattttaggngctgttaattttattactactgtaattaatatacgatcaacaggagttacttttgatcgaatacctttatttgtatgagccgtagtaattacagctattcttttattattatcattaccagtattagctggagctattactatactattaactgatcgaaatttaaatacttcattctttgaccctgcaggaggaggagaccctattttatatcaacatttattt

>TCAP030-06|Hybomitra affinis|COI-5P|KM285564

aacattatattttatttttggagcgtgagctggaataattggtacctcattaagtattttaattcgagctgaattaggacatcctggatcattaattggagatgaccaaatttataatgtaattgtaacagcacatgcttttgtaataattttttttatagtaatacctattataattggaggatttggaaattgattagttcctttaatattaggagctcctgatatagcattccctcgaataaataatataagtttctgattactcccaccatcattaactcttttattagccagtagtatagtagaaaatggggctggaactggttgaacagtttacccccctctatctgctgcaattgctcatggaggaggatcagttgatttagcaattttttctttacatttagcaggaatttcttctattttaggngctgttaattttattactactgtaattaatatacgatcaacaggagttacttttgatcgaatacctttatttgtatgagccgtagtaattacagctattcttttattattatcattaccagtattagctggagctattactatactattaactgatcgaaatttaaatacttcattctttgaccctgcaggaggaggagaccctattttatatcaacatttattt

>SAMOS077-09|Hybomitra affinis|COI-5P|KR754372

aacattatattttatttttggagcgtgagctggaataattggtacctcattaagtattttaattcgagctgaattaggacatcctggatcattaattggagatgaccaaatttataatgtaattgtaacagcacatgcttttgtaataattttttttatagtaatacctattataattgggggatttggaaattgattagttcctttaatattaggagctcctgatatagcattccctcgaataaataatataagtttctgattacttccaccatcattaactcttttattagccagtagtatagtagaaaatggagctggaactggttgaacagtttacccccctctatctgctgcaattgctcatggaggaggatcagttgatttagcaattttttctttacatttagcaggaatttcttctattttaggggctgttaattttattactactgtaattaatatacgatcaacaggagttacttttgaccgaatacctttatttgtatgagccgtagtaattacagctattcttttattattatcattaccagtattagctggagctattactatactattaactgatcgaaatttaaatacttcattctttgaccctgcaggaggaggagaccctattttatatcaacatttattt

>JWDCD174-10|Hybomitra affinis|COI-5P|HM862220

aacattatattttatttttggagcgtgagctggaataatcggtacctcattaagtattttaattcgagctgaattagggcatcctggatcattaattggagatgaccaaatttataatgtaattgtaacagcacatgcttttgtaataattttttttatagtaatacctattataattgggggatttggaaattgattagttcctttaatattaggagctcctgatatagcattccctcgaataaataatataagtttctgattacttccaccatcattaactcttttattagccagtagtatagtagaaaatggggctggaactggttgaacagtttacccccctctatctgctgcaattgctcatggaggaggatcagttgatttagcaattttttctttacatttagcaggaatttcttctattttaggggctgttaattttattactactgtaattaatatacgatcaacaggagttacttttgatcgaatacctttatttgtatgagccgtagtaattacagctattcttttattattgtcattaccagtattagctggagctattactatactattaactgatcgaaatttaaatacttcattctttgaccctgcaggaggaggagaccctattttatatcaacatttattt

>CNPKG1445-14|Hybomitra|COI-5P|KR396570

aacattatattttatttttggagcgtgagctggaataattggtacctcattaagtattttaattcgagctgaattaggacatcctggatcattaattggagatgaccaaatttataatgtaattgtaacagcacatgcttttgtaataattttttttatagtaatacctattataattggaggatttggaaattgattagttcctttaatattaggagctcctgatatagcattccctcgaataaataatataagtttctggttactcccaccatcattaactcttttattagccagtagtatagtagaaaatggggctggaactggttgaacagtttacccccctctatctgctgcaattgctcatggaggaggatcagttgatttagcaattttttctttacatttagcaggaatttcttctattttaggggctgttaattttattactactgtaattaatatacgatcaacaggagttacttttgatcgaatacctttatttgtatgagccgtagtaattacagctattcttttattattatcattaccagtattagctggagctattactatactattaactgatcgaa-----------------------------------------------------------------

>CNKOP089-14|Hybomitra|COI-5P|KR382665

----------tttatttttggagcgtgagctggaataattggtacctcattaagtattttaattcgagctgaattaggacatcctggatcattaattggagatgaccaaatttataatgtaattgtaacagcacatgcttttgtaataattttttttatagtaatacctattataattgggggatttggaaattgattagttcctttaatattaggagctcctgatatagcattccctcgaataaataatataagtttctgattacttccaccatcattaactcttttattagccagtagtatagtagaaaatggggctggaactggttgaacagtttacccccctctatctgctgcaattgctcatggaggaggatcagttgatttagcaattttttctttacatttagcaggaatttcttctattttaggggctgttaattttattactactgtaattaatatacgatcaacaggagttacttttgatcgaatacctttatttgtatgagccgtagtaattacagctattcttttattattatcattaccagtattagctggagctattactatactattaactgat---------------------------------------------------------------------

>SSKJB1146-14|Hybomitra|COI-5P|MF832490

----------tttatttttggagcgtgagctggaataattggtacctcattaagtattttaattcgagctgaattaggacatcctggatcattaattggagatgaccaaatttataatgtaattgtaacagcacatgcttttgtaataattttttttatagtaatacctattataattgggggatttggaaattgattagttcctttaatattaggggctcctgatatagcattccctcgaataaataatataagtttctgattacttccaccatcattaactcttttattagccagtagtatagtagaaaatggggctggaactggttgaacagtttacccccctctatctgctgcaattgctcatggaggaggatcagttgatttagcaattttttctttacatttagcaggaatttcttctattttaggggctgttaattttattactactgtaattaatatacgatcaacaggagttacttttgatcgaatacctttatttgtatgagccgtagtaattacagctattcttttattattatcattaccagtattagctggagctattactata---------------------------------------------------------------------------------

>SSBAE1234-13|Hybomitra|COI-5P|KM943152

-acattatattttatttttggagcgtgagctggaataattggtacctcattaagtattttaattcgagctgaattaggacatcctggatcattaattggagatgaccaaatttataatgtaattgtaacagcacatgcttttgtaataattttttttatagtaatacctattataattgggggatttggaaattgattagttcctttaatattaggagctcctgatatagcattccctcgaataaataatataagtttctgattacttccaccatcattaactcttttattagccagtagtatagtagaaaatggggctggaactggttgaacagtttacccccctctatctgctgcaattgctcatggaggaggatcagttgatttagcaattttttctttacatttagcaggaatttcttctattttaggggctgttaattttattactactgtaattaatatacgatcaacaggagttacttttgatcgaatacctttatttgtatgagccgtagtaattacagctattcttttattattatcattaccagtattagctggagctattactatactattaactgatcga------------------------------------------------------------------

>NBPSI281-11|Diptera|COI-5P

aacattatattttatttttggagcatgagctggaataatcggtacttcattaagtattttaattcgagctgaattaggacaccctggatcattaattggggatgaccaaatttataatgtaattgtaacagcacatgcttttgtaataattttctttatagtaatacctattataattggaggatttggaaattgattagttcctttaatattaggagctcctgatatagcatttcctcgaataaataatataagtttttgattacttcccccatcattaacccttttattagccagtagtatagtagaaaatggggctggaactggttgaacagtttacccccctctatctgctgcaattgctcatggaggaggatcagttgatttagcaattttttctttacatttagcaggaatttcttctattttaggagctgttaattttattactactgtaattaatatacgatcaacaggagttacttttgatcgaatacctttatttgtatgagcagtagtaattacagctattcttttattattatcattaccagtattagctggagctattactatattattaactgatcgaaatttaaatacctcattctttgatcctgcaggaggaggagatcctattttataccaacatttattt

>NBPSI282-11|Diptera|COI-5P

aacattatattttatttttggagcatgagctggaataatcggtacttcattaagtattttaattcgagctgaattaggacaccctggatcattaattggggatgaccaaatttataatgtaattgtaacagcacatgcttttgtaataattttctttatagtaatacctattataattggaggatttggaaattgattagttcctttaatattaggagctcctgatatagcatttcctcgaataaataatataagtttttgattacttcccccatcattaacccttttattagccagtagtatagtagaaaatggggctggaactggttgaacagtttacccccctctatctgctgcaattgctcatggaggaggatcagttgatttagcaattttttctttacatttagcaggaatttcttctattttaggagctgttaattttattactactgtaattaatatacgatcaacaggagttacttttgatcgaatacctttatttgtatgagcagtagtaattacagctattcttttattattatcattaccagtattagctggagctattactatattattaactgatcgaaatttaaatacctcattctttgatcctgcaggaggaggagatcctattttataccaacatttattt

>NBPSI283-11|Diptera|COI-5P

aacattatattttatttttggagcatgagctggaataatcggtacttcattaagtattttaattcgagctgaattaggacaccctggatcattaattggggatgaccaaatttataatgtaattgtaacagcacatgcttttgtaataattttctttatagtaatacctattataattggaggatttggaaattgattagttcctttaatattaggagctcctgatatagcatttcctcgaataaataatataagtttttgattacttcccccatcattaacccttttattagccagtagtatagtagaaaatggggctggaactggttgaacagtttacccccctctatctgctgcaattgctcatggaggaggatcagttgatttagcaattttttctttacatttagcaggaatttcttctattttaggagctgttaattttattactactgtaattaatatacgatcaacaggagttacttttgatcgaatacctttatttgtatgagcagtagtaattacagctattcttttattattatcattaccagtattagctggagctattactatattattaactgatcgaaatttaaatacctcattctttgatcctgcaggaggaggagatcctattttataccaacatttattt

>CNTGC128-15|Hybomitra|COI-5P|KR979547

-------------atttttggagcatgagctggaataatcggtacttcattaagtattttaattcgagctgaattaggacaccctggatcattaattggggatgaccaaatttataatgtaattgtaacagcacatgcttttgtaataattttctttatagtaatacctattataattggaggatttggaaattgattagttcctttaatattaggagctcctgatatagcatttcctcgaataaataatataagtttttgattacttcccccatcattaacccttttattagccagtagtatagtagaaaatggggctggaactggttgaacagtttacccccctctatctgctgcaattgctcatggaggaggatcagttgatttagcaattttttctttacatttagcaggaatttcttctattttaggagctgttaattttattactactgtaattaatatacgatcaacaggagttacttttgatcgaatacctttatttgtatgagcagtagtaattacagctattcttttattattatcattaccagtattagctggagctattactata---------------------------------------------------------------------------------

>FIDIP2964-12|Hybomitra kaurii|COI-5P

aacattatattttatttttggggcatgagctggaataattggtacttcattaagtattttaattcgagctgaattagggcatcctggatcattaattggtgatgaccaaatttataatgtaattgtaacagcacatgcttttgtaataattttctttatagtaatacctattataattggaggatttggaaattgattagttcctttaatattaggagctcctgatatagcatttcctcgaataaataatataagtttttgattacttccaccatcattaacccttttattagccagtagtatagtagaaaatggagctggaactggttgaacagtttatccacctctatctgctgcaattgctcatggaggaggatcagttgatttagcaattttttcattacatttagcaggaatttcttctattttaggagctgttaattttattactactgtaattaatatacgatcaacaggaattacttttgaccgaatacctttatttgtatgagcagtagtaattacagctattcttttattattatcattaccagtattagctggagctattactatattattaactgatcgaaatttaaatacctcattttttgaccctgcaggaggaggagatcctatcttatatcaacatttattc

>FIDIP213-11|Hybomitra kaurii|COI-5P

aacattatattttatttttggggcatgagctggaataattggtacttcattaagtattttaattcgagctgaattagggcatcctggatcattaattggtgatgaccaaatttataatgtaattgtaacagcacatgcttttgtaataattttctttatagtaatacctattataattggaggatttggaaattgattagttcctttaatattaggagctcctgatatagcatttcctcgaataaataatataagtttttgattacttccaccatcattaacccttttattagccagtagtatagtagaaaatggagctggaactggttgaacagtttatccacctctatctgctgcaattgctcatggaggaggatcagttgatttagcaattttttcattacatttagcaggaatttcttctattttaggagctgttaattttattactactgtaattaatatacgatcaacaggaattacttttgaccgaatacctttatttgtatgagcagtagtaattacagctattcttttattattatcattaccagtattagctggagctattactatattattaactgatcgaaatttaaatacctcattttttgaccctgcaggaggaggagatcctatcttatatcaacatttattc

>DIPFI006-12|Hybomitra kaurii|COI-5P

aacattatattttatttttggggcatgagctggaataatcggtacttcattaagtattttaattcgagctgaattagggcatcctggatcattaattggtgatgaccaaatttataatgtaattgtaacagcacatgcttttgtaataattttctttatagtaatacctattataattggaggatttggaaattgattagttcctttaatattaggagctcctgatatagcatttcctcgaataaataatataagtttttgattacttccaccatcattaacccttttattagccagtagtatagtagaaaatggagctggaactggttgaacagtttatccacctctatctgctgcaattgctcatggaggaagatcagttgatttagcaattttttcattacatttagcaggaatttcttctattttaggagctgttaattttattactactgtaattaatatacgatcaacaggaattacttttgaccgaatacctttatttgtatgagcagtagtaattacagctattcttttattattatcattaccagtattagctggagctattactatattattaactgatcgaaatttaaatacctcattttttgaccctgcaggaggaggagatcctatcttatatcaacatttattc

>FIDIP3702-13|Hybomitra sexfasciata|COI-5P

aacattatattttatttttggggcatgagctggaataattggtacttcattaagtattttaattcgagctgaattagggcatcctggatcattaattggtgatgaccaaatttataatgtaattgtaacagcacatgcttttgtaataattttctttatagtaatacctattataattggaggatttggaaattgattagttcctttaatattaggagctcctgatatagcatttcctcgaataaataatataagtttttgattacttccaccatcattaacccttttattagccagtagtatagtagaaaatggagctggaactggttgaacagtttatccacctctatctgctgcaattgctcatggaggaggatcagttgatttagcaattttttcattacatttagcaggaatttcttctattttaggagctgttaattttattactactgtaattaatatacgatcaacaggaattacttttgaccgaatacctttatttgtatgagcagtagtaattacagctattcttttattattatcattaccagtattagctggagctattactatattattaactgatcgaaatttaaatacatcattttttgaccctgcaggaggaggagatcctatcttatatcaacatttattc

>FIDIP2960-12|Hybomitra kaurii|COI-5P

---------------ttttggggcatgagctggaataattggtacttcattaagtattttaattcgagntgaattagggcatcctggatcattaattggtgatgaccaaatttataatgtaattgtaacagcacatgcttttgtaataattttctttatagtaatacctattataattggaggatttggaaattgattagttcctttaatattaggagctcctgatatagcatttcctcgaataaataatataagtttttgattacttccaccatcattaacccttttattagccagtagtatagtagaaaatggagctggaactggttgaacagtttatccacctctatctgctgcaattgctcatggaggaggatcagttgatttagcaattttttcattacatttagcaggaatttcttctattttaggagctgttaattttattactactgtaattaatatacgatcaacaggaattacttttgaccgaatacctttatttgtatgagcagtagtaattacagctattcttttattattatcattaccagtattagctggagctattactatattattaactgatcgaaatttaaatacctcattttttgaccctgcaggaggaggagatcctatcttatatcaacatttattc

>NODIP194-13|Diptera|COI-5P

aacattatattttatttttggggcatgagctggaataattggtacttcattaagtattttaattcgagctgaattagggcatcctggatcattaattggtgatgaccaaatttataatgtaattgtaacagcacatgcttttgtaataattttctttatagtaatacctattataattggaggatttggaaattgattagttcctttaatattaggagctcctgatatagcatttcctcgaataaataatataagtttttgattacttccaccatcattaacccttttattagccagtagtatagtagaaaatggagctggaactggttgaacagtttacccccctctatctgctgcaatcgctcatggaggaggatcagttgatttagcaattttctcactacatttagcaggaatttcttctattttaggagctgttaattttattactactgtaattaatatacgatcaacaggaattacttttgatcgaatacctttatttgtatgagcagtagtaattacagctattcttttattattatcattaccagtattagctggagccattactatactcttaactgatcgaaatttaaatacctcattttttgatcctgcaggaggaggagatcctattttatatcaacatttattc

>UAMIC1190-13|Hybomitra polaris|COI-5P|KU875229

aacattatattttatttttggggcatgagctggaataattggtacttcattaagtattttaattcgagctgaattagggcatcctggatcattaattggtgatgaccaaatttataatgtaattgtaacagcacatgcttttgtaataattttctttatagtaatacctattataattggaggatttggaaattgattagttcctttaatattaggagctcctgatatagcatttcctcgaataaataatataagtttttgattacttccaccatcattaacccttttattagccagtagtatagtagaaaatggagctggaactggttgaacagtttacccccctctatctgctgcaatcgctcatggaggaggatcagttgatttagcaattttctcactacatttagcaggaatttcttctattttaggagctgttaattttattactactgtaattaatatacgatcaacaggaattacttttgatcgaatacctttatttgtatgagcagtagtaattacagctattcttttattattatcattaccagtattagctggagccattactatactcttaactgatcgaaatttaaatacctcattttttgatcctgcaggaggaggagatcctattttatatcaacatttattc

>NODIP249-13|Diptera|COI-5P

aacattatattttatttttggggcatgagctggaataatcggtacttcattaagtattttaattcgagctgaattagggcatcctggatcattaattggtgatgaccaaatttataatgtaattgtaacagcacatgcttttgtaataattttctttatagtaatacctattataattggaggatttggaaattgattagttcctttaatattaggagctcctgatatagcatttcctcgaataaataatataagtttttgactacttccaccatcattaacccttttattagccagtagtatagtagaaaatggagctggaactggttgaacagtttacccccctctatctgctgcaattgctcatggaggaggatcagttgatttagcaattttctcattacatttagcaggaatttcttctattttaggagctgttaattttattactactgtaattaatatacgatcaacaggaattacttttgaccgaatacctttatttgtatgagcagtagtaattacagctattcttttattattatcattaccagtattagctggngccattactatactcttaactgaccgaaatttaaatacctcattttttgatcctgcaggaggaggagatcctattttatatcaacatttattc

>INRMA3561-20|Hybomitra sexfasciata|COI-5P

aacattatattttatttttggggcatgagctggaataattggtacttcattaagtatttt-attcgagctgaattagggcatcctggatcattaattggtgatgaccaaatttataatgtaattgtaacagcacatgcttttgtaataattttctttatagtaatacctattataattggaggatttggaaattgattagttcctttaatattaggagctcctgatatagcatttcctcgaataaataatataagtttttgattacttccaccatcattaacccttttattagccagtagtatagtagaaaatggagctggaactggttgaacagtttacccccctctatctgctgcaatcgctcatggaggaggatcagttgatttagcaattttctcactacatttagcaggaatttcttctattttaggagctgttaattttattactactataattaatatacgatcaacaggaattacttttgatcgaatacctttatttgtatgagcagtagtaattacagctattcttttattattatcattaccagtattagctggagccattactatactcttaactgatcgaaatttaaatacctcattttttgatcctgcaggaggaggaga-------------------------

>JWDCD180-10|Hybomitra affinis|COI-5P|HM862224

---------------------------------------tggtacctcattaagtattttaattcgagctgaattaggacatcctggatcattaattggagatgaccaaatttataatgtaattgtaacagcacatgcttttgtaataattttttttatagtaatacctattataattgggggatttggaaattgattagttcctttaatattaggagctcctgatatagcattccctcgaataaataatataagtttctgattacttccaccatcattaactcttttattagccagtagtatagtagaaaatggggctggaactggttgaacagtttacccccctctatctgctgcaattgctcatggaggaggatcagttgatttagcaattttttctttacatttagcaggaatttcttctattttaggggctgttaattttattactactgtaattaatatacgatcaacaggagttacttttgatcgaatacctttatttgtatgagccgtagtaattacagctattcttttattattatcattaccagtattagctggagctattactatactattaactgatcgaaatttaaatacttcattctttgaccctgcaggaggaggagaccctattttatatcaacatttattt

>SSPAB1604-13|Hybomitra|COI-5P|KM902267

---------------------------------------------------------------------------------------------------agatgaccaaatttataatgtaattgtaacagcacatgcttttgtaataattttttttatagtaatacctattataattgggggatttggaaattgattagttcctttaatattaggagctcctgatatagcattccctcgaataaataatataagtttctgattacttccaccatcattaactcttttattagccagtagtatagtagaaaatggggctggaactggttgaacagtttacccccctctatctgctgcaattgctcatggaggaggatcagttgatttagcaattttttctttacatttagcaggaatttcttctattttaggggctgttaattttattactactgtaattaatatacgatcaacaggagttacttttgatcgaatacctttatttgtatgagccgtagtaattacagctattcttttattattatcattaccagtattagctggagctattactatactattaactgatcgaaatttaaatacttcattc------------------------------------------------

>ACT053-07|Hybomitra affinis|COI-5P|KM285577

----------------------------------------ggtacctcattaagtatcttaattcgagctgaattaggacatcctggatcattaattggagatgaccaaatttataatgtaattgtaacagcacatgcttttgtaataattttttttatagtaatacctattataattgggggatttggaaattgattagttcctttaatattaggagctcctgatatagcattccctcgaataaataatawaagtttctgattacttccaccatcattaactcttttattagccagtagtatagtagaaaatggggctggaactggttgaacagtttacccccctctatcwgctgcaattgctcatggaggaggatcagttgatttagcaattttttctttacatttagcaggaatttcttctattttaggagctgttaattttattactactgtaattaatatacgatcaacaggagttacttttgaccgawtacctttatttgtatgagcagtagtaattacagctattcttttattattatcattaccagtattagctggagctattactatactattaactgatcgaaatttaaatacttcattctttgaccctgcaggaggaggagatcctattttatatcaacatttattt

>NODIP197-13|Diptera|COI-5P

aacattatattttatttttggggcatgagctggaataattggtacttcattaagtattttaattcgagctgaattagggcatcctggatcattaattggtgatgaccaaatttataatgtaattgtaacagcacatgcttttgtaataattttctttatagtaatacctattataattggaggatttggaaattgactagttcctttaatattaggagctcctgatatagcattcccccgaataaataatataagtttttgattacttccaccatcattgactcttttattagccagtagtatagtagaaaatggggctggaactggttgaacagtttacccccctctgtctgctgcaattgcccatggaggaggatcagttgatttggcaattttttctttacatttagcaggaatttcttctattttaggagctgttaattttattactactgtaattaatatacgatcaacaggaattacttttgaccggatacctttatttgtatgagcagtagtaattacagctattcttttattattatcattaccagtattagccggagctattactatattattaactgatcgaaatttaaatacttcattctttgaccctgcaggaggtggagaccctattttataccaacatttattt

>NOBRA296-15|Diptera|COI-5P

aacattatattttatttttggggcatgagctggaataattggtacttcattaagtattttaattcgagctgaattagggcatcctggatcattaattggtgatgaccaaatttataatgtaattgtaacagcacatgcttttgtaataattttctttatagtaatacctattataattggaggatttggaaattgactagttcctttaatattaggagctcctgatatagcattcccccgaataaataatataagtttttgattacttccaccatcattgactcttttattagccagtagtatagtagaaaatggggctggaactggttgaacagtttacccccctctgtctgctgcaattgcccatggaggaggatcagttgatttggcaattttttctttacatttagcaggaatttcttctattttaggagctgttaattttattactactgtaattaatatacgatcaacaggaattacttttgaccggatacctttatttgtatgagcagtagtaattacagctattcttttattattatcattaccagtattagccggagctattactatattattaactgatcgaaatttaaatacttcattctttgaccctgcaggaggtggagaccctattttataccaacatttattt

>FIDIP234-11|Hybomitra tarandina|COI-5P

aacattatattttatttttggggcatgagctggaataattggtacttcattaagtattttaattcgagctgaattagggcatcctggatcattaattggtgatgaccaaatttataatgtaattgtaacagcacatgcttttgtaataattttctttatagtaatacctattataattggaggatttggaaattgactagttcctttaatattaggagctcctgatatagcattcccccgaataaataatataagtttttgattacttccaccatcattgactcttttattagccagtagtatagtagaaaatggggctggaactggttgaacagtttacccccctctgtctgctgcaattgcccatggaggaggatcagttgatttggcaattttttctttacatttagcaggaatttcttctattttaggagctgttaattttattactactgtaattaatatacgatcaacaggaattacttttgaccggatacctttatttgtatgagcagtagtaattacagctattcttttattattatcattaccagtattagccggagctattactatattattaactgatcgaaatttaaatacttcattctttgaccctgcaggaggtggagaccctattttataccaacatttattt

>FIDIP285-11|Hybomitra tarandina|COI-5P

aacattatattttatttttggggcatgagctggaataattggtacttcattaagtattttaattcgagctgaattagggcatcctggatcattaattggtgatgaccaaatttataatgtaattgtaacagcacatgcttttgtaataattttctttatagtaatacctattataattggaggatttggaaattgactagttcctttaatattaggagctcctgatatagcattcccccgaataaataatataagtttttgattacttccaccatcattgactcttttattagccagtagtatagtagaaaatggggctggaactggttgaacagtttacccccctctgtctgctgcaattgcccatggaggaggatcagttgatttggcaattttttctttacatttagcaggaatttcttctattttaggagctgttaattttattactactgtaattaatatacgatcaacaggaattacttttgaccggatacctttatttgtatgagcagtagtaattacagctattcttttattattatcattaccagtattagccggagctattactatattattaactgatcgaaatttaaatacttcattctttgaccctgcaggaggtggagaccctattttataccaacatttattt

>FIDIP211-11|Hybomitra tarandina|COI-5P

aacattatattttatttttggggcatgagctggaataattggtacttcattaagtattttaattcgagctgaattagggcatcctggatcattaattggtgatgaccaaatttataatgtaattgtaacagcacatgcttttgtaataattttctttatagtaatacctattataattggaggatttggaaattgactagttcctttaatattaggagctcctgatatagcattcccccgaataaataatataagtttttgattacttccaccatcattgactcttttattagccagtagtatagtagaaaatggggctggaactggttgaacagtttacccccctctgtctgctgcaattgcccatggaggaggatcagttgatttggcaattttttctttacatttagcaggaatttcttctattttaggagctgttaattttattactactgtaattaatatacgatcaacaggaattacttttgaccggatacctttatttgtatgagcagtagtaattacagctattcttttattattatcattaccagtattagccggagctattactatattattaactgatcgaaatttaaatacttcattctttgaccctgcaggaggtggagaccctattttataccaacatttattt

>NOBRA290-15|Diptera|COI-5P

aacattatattttatttttggagcatgagctggaataattggtacttcattaagtatcttaattcgagctgaattaggacatcccgggtcattgattggagatgaccaaatttataatgtaattgtaacagcacatgcttttgtaataattttctttatagtaatacctattataattggaggatttggaaattgattagttcctttaatattaggagctcctgatatagcatttcctcgaataaataatataagtttctgacttcttcctccatcattaactcttttattagccagtagtatagtagaaaatggggctggaacaggttgaacagtttacccccctctatctgctgcaattgctcatggaggaggatcagtcgatttagcaatcttttctttacatttagcaggaatttcttctattttaggagctgttaattttattactactgtaattaatatacgatcaacaggagttacttttgaccgaatacctttatttgtatgagcagtagtaattacagctattcttttattactatcactaccagtattagctggggctattactatacttttaactgatcgaaatttaaatacctcattttttgaccctgcaggaggaggagaccctattttatatcaacatctattt

>FIDIP2965-12|Hybomitra lundbecki|COI-5P

aacattatattttatttttggagcatgagctggaataattggtacttcattaagtatcttaattcgagctgaattaggacatcccggatcattgattggagatgaccaaatttataatgtaattgtaacagcacatgcttttgtaataattttctttatagtaatacctattataattggaggatttggaaattgattagttcctttaatattaggagctcctgatatagcatttcctcgaataaataatataagtttctgacttcttcctccatcattaactcttttattagccagtagtatagtagaaaatggggctggaacaggttgaacagtttacccccctctatctgctgcaattgctcatggaggaggatcagtcgatttagcaatcttttctttacatttagcaggaatttcttctattctaggagctgttaattttattactactgtaattaatatacgatcaacaggagttacttttgaccgaatacctttatttgtatgagcagtagtaattacagctattcttttattactatcactaccggtattagctggagctattactatacttttaactgatcgaaatttaaatacctcattttttgaccctgcaggaggaggagatcctattttatatcaacatctattt

>NODIP223-13|Diptera|COI-5P

aacattatattttatttttggagcatgagctggaataattggtacttcattaagtatcttaattcgagctgaattaggacatcccggatcattgattggagatgaccaaatttataatgtaattgtaacagcacatgcttttgtaataattttctttatagtaatacctattataattggaggatttggaaattgattagttcctttaatattaggagctcctgatatagcatttcctcgaataaataatataagtttctgacttcttcccccatcattaactcttttattagccagtagtatagtagaaaatggggctggaacaggttgaacagtttacccccctctatctgctgcaattgctcatggaggaggatcagtcgatttagcaatcttttctttacatttagcaggaatttcttctattttaggagctgttaattttattactactgtaattaatatacgatcaacaggagttacttttgaccgaatacctttatttgtatgagcagtagtaattacagctattcttttattactatcactaccagtattagctggagctattactatacttttaactgatcgaaatttaaatacctcattttttgaccctgcaggaggaggagatcctattttatatcaacatctattt

>FIDIP2975-12|Hybomitra lundbecki|COI-5P

aacattatattttatttttggagcatgagctggaataattggtacttcattaagtatcttaattcgagctgaattaggacatcccggatcattgattggagatgaccaaatttataatgtaattgtaacagcacatgcttttgtaataattttctttatagtaatacctattataattggaggatttggaaattgattagttcctttaatattaggagctcctgatatagcatttcctcgaataaataatataagtttctgacttcttcctccatcattaactcttttattagccagtagtatagtagaaaatggggctggaacaggttgaacagtttacccccctctatctgctgcaattgctcatggaggaggatcagtcgatttagcaatcttttctttacatttagcaggaatttcttctattttaggagctgttaattttattactactgtaattaatatacgatcaacaggagttacttttgaccgaatacctttatttgtatgagcagtagtaattacagctattcttttattactatcactaccagtattagctggagctattactatacttttaactgatcgaaatttaaatacctcattttttgaccctgcaggaggaggagatcctattttatatcaacatctattt

>DIPFI001-12|Hybomitra lundbecki|COI-5P

aacattatattttatttttggagcatgggctggaataattggtacttcattaagtatcttaattcgagctgaattaggacatcccggatcattgattggagatgaccaaatttataatgtaattgtaacagcacatgcttttgtaataattttctttatagtaatacctattataattggaggatttggaaattgattagttcctttaatattaggagctcctgatatagcatttcctcgaataaataatataagtttctgacttcttcctccatcattaactcttttattagccagtagtatagtagaaaatggggctggaacaggttgaacagtttacccccctctatctgctgcaattgctcatggaggaggatcagtcgatttagcaatcttttctttacatttagcaggaatttcttctattttaggagctgttaattttattactactgtaattaatatacgatcaacaggagttacttttgaccgaatacctttatttgtatgagcagtagtaattacagctattcttttattactatcactaccagtattagctggagctattactatacttttaactgatcgaaatttaaatacctcattttttgaccctgcaggaggaggagatcctattttatatcaacatctattt

>FIDIP2966-12|Hybomitra lundbecki|COI-5P
[truncated: 121,777 more chars]
