# Supplementary figures and images for: ﻿DNA barcoding of the horsefly fauna (Diptera, Tabanidae) of Croatia with notes on the morphology and taxonomy of selected species from Chrysopsinae and Tabaninae
Source: Zookeys. 2022 Feb 23;1087:141–61. doi: 10.3897/zookeys.1087.78707 (PMC8891235; doi:10.3897/zookeys.1087.78707)

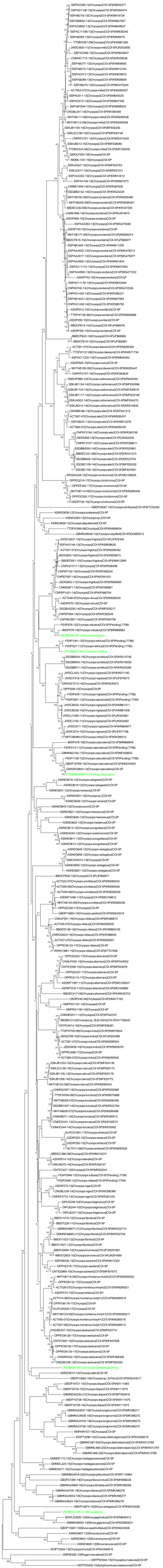

Supplement: Supplementary material 3 — Figure S1. NJ tree for the tribe Chrysopsini [file zookeys-1087-141-s003.pdf]

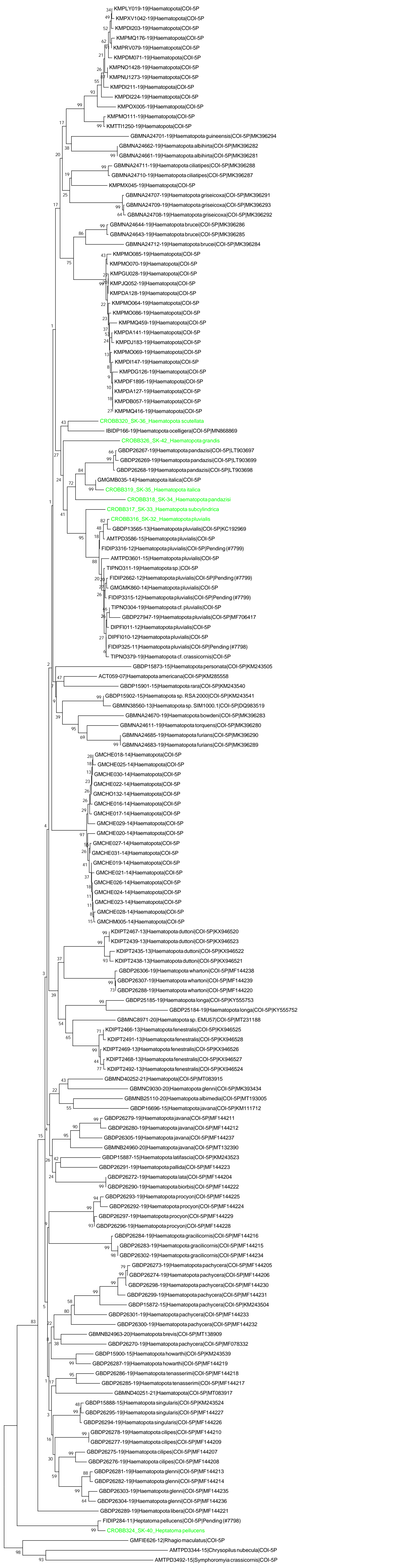

0.01

Supplement: Supplementary material 4 — Figure S2. NJ tree for the tribes Haematopotini and Heptatomini [file zookeys-1087-141-s004.pdf]

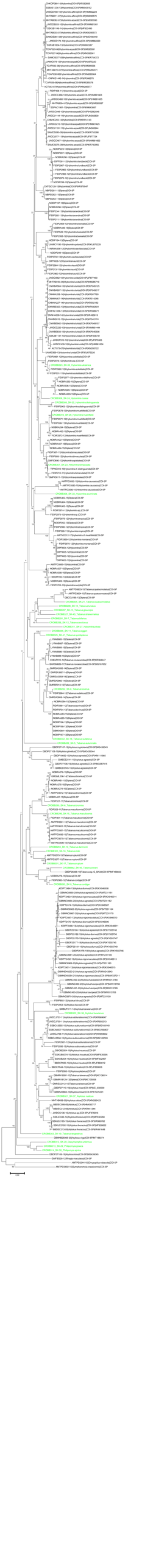

Supplement: Supplementary material 5 — Figure S3. NJ tree for the tribes Tabanini and Diachlorini [file zookeys-1087-141-s005.pdf]
